# Transcriptome Analysis of Gelatin Seed Treatment as a Biostimulant of Cucumber Plant Growth

**DOI:** 10.1155/2015/391234

**Published:** 2015-10-08

**Authors:** H. T. Wilson, K. Xu, A. G. Taylor

**Affiliations:** Cornell University School of Integrated Plant Science, New York State Agriculture Experimental Station, Geneva, NY 24456, USA

## Abstract

The beneficial effects of gelatin capsule seed treatment on enhanced plant growth and tolerance to abiotic stress have been reported in a number of crops, but the molecular mechanisms underlying such effects are poorly understood. Using mRNA sequencing based approach, transcriptomes of one- and two-week-old cucumber plants from gelatin capsule treated and nontreated seeds were characterized. The gelatin treated plants had greater total leaf area, fresh weight, frozen weight, and nitrogen content. Pairwise comparisons of the RNA-seq data identified 620 differentially expressed genes between treated and control two-week-old plants, consistent with the timing when the growth related measurements also showed the largest differences. Using weighted gene coexpression network analysis, significant coexpression gene network module of 208 of the 620 differentially expressed genes was identified, which included 16 hub genes in the blue module, a NAC transcription factor, a MYB transcription factor, an amino acid transporter, an ammonium transporter, a xenobiotic detoxifier-glutathione S-transferase, and others. Based on the putative functions of these genes, the identification of the significant WGCNA module and the hub genes provided important insights into the molecular mechanisms of gelatin seed treatment as a biostimulant to enhance plant growth.

## 1. Introduction

Seed gelatin encapsulation is a technology developed by Alliance Seed Capsule, a consortium between Coating Supply and Sakata Seed, in which raw and/or processed seeds are encapsulated in gelatin capsule (Supplementary Figure 1A, B in Supplementary Material available online at http://dx.doi.org/10.1155/2015/391234). The encapsulation provides several advantages such as improved handling and sowing, faster plant growth, precision planting, and precise seed quantities per sowing unit and also provides the capability of combining seed enhancement technologies and other chemicals or biological additives such as pesticides, fertilizers, and* Rhizobium* [1].

Plant biostimulants are a broad class of substances and microorganisms that enhance plant growth. Biostimulants have been gaining interest in sustainable agriculture because they stimulate physiological process in plants that enhances plant development and nutrient use efficiency, which reduces fertilizer consumption [2, 3]. Many biostimulants have been reported to counteract the effect of biotic and abiotic stresses, enhancing quality and crop yield [3, 4]. The European Biostimulants Industry Council (EBIC) has defined plant biostimulants as substance(s) and/or microorganisms whose function when applied to plants or the rhizosphere is to stimulate natural processes to enhance/benefit nutrient uptake, nutrient efficiency, tolerance to abiotic stress, and crop quality. Biostimulants have no direct action against pests and therefore do not fall within the regulatory framework of pesticides [5, 6]. Thus the application of gelatin as a seed treatment falls under the category of plant biostimulants as it uses animal based protein hydrolysate, a hydrolyzed form of protein, as the main component.

Protein hydrolysate and other protein-based product applications were reported to enhance plant growth and yield in field tomato [7], greenhouse tomato [8], papaya [9], maize seedlings [10, 11], spinach [2], carrot [12], and lettuce [13, 14]. A positive effect of protein hydrolysate on abiotic stress also was reported, and cucumber plants subjected to suboptimal pH level and temperature performed better with the application of humic acid containing substance LACTOFOL, with notable increase in leaf area, shoot, and root mass [15, 16]. Atonik, a synthetic biostimulant composed of nitrophenolates, have been shown to increase leaf area and root system development. Plants treated with nitrophenolates have been characterized as having greater inhibition of IAA oxidase, which ensures a higher activity of naturally synthesized auxins [17]. MEGAFOL, a biostimulant composed of a complex of vitamins, amino acids, proteins, and betaines, positively increased crop yield in sweet yellow pepper [18]. The biostimulant also provided drought stress resistance in tomato, and MEGAFOL treated plants were able to recover more quickly from drought stress. Analyzing the expression levels of drought-stress marker genes in tomato plants treated with MEGAFOL demonstrated a lower expression of drought-related genes [19]. An alfalfa plant-derived biostimulant increased maize plant biomass under salinity condition and enhanced Na^+^ accumulation and reduced K^+^ accumulation in roots and leaves [10]. Perennial ryegrass treated with an animal membrane hydrolysate, Macro-Sorb Foliar (FOLIAR), when subjected to high air temperature stress exhibited increased photochemical efficiency and membrane thermostability compared to plants without the treatment [20]. Seed priming with BABA (*β*-amino butyric acid), a nonprotein amino acid, reduced the abiotic stress in* Vigna radiate*, by enhancing photosynthesis and proline accumulation and antioxidant enzyme activities in seedlings subjected to NaCl/PEG [21].

Protein hydrolysate was shown to stimulate carbon and nitrogen metabolism and increase nitrogen assimilation in plants [22, 23]. A biostimulant containing amino acid, Aminoplant, enhanced the nitrate reductase activity in spinach [2]. NAD-dependent glutamate dehydrogenase, nitrate reductase, and malate dehydrogenase in maize had higher activities following application of animal epithelial hydrolysate [24]. Alfalfa protein hydrolysate applied to hydroponically grown maize increased the activity of malate dehydrogenase, isocitrate dehydrogenase, and citrate synthase, which are enzymes found in the TCA cycle, as well as nitrogen metabolism enzymes: nitrate reductase, nitrite reductase, glutamine, glutamine synthetase (GS), glutamine synthase, and aspartate aminotransferase [22]. Ertani et al. [23] reported that both alfalfa protein hydrolysate and animal connective tissue hydrolysate stimulated plant growth and also increased nitrate conversion into organic nitrogen by inducing nitrate reductase and GS activities. The treatments especially enhanced the GS2 isoform, which is responsible for assimilation of ammonia produced by nitrate reduction, thus confirming that protein hydrolysate enhances plant growth by upregulating nitrate assimilation [23, 25]. Similarly, Vaccaro et al. [26] demonstrated that low concentration of humic acid positively influenced nitrate metabolism by increasing the content of soluble protein and amino acid synthesis, which was stimulated by significant increase in activity and transcription of enzymes functioning in N assimilation and Krebs cycle.

Seed encapsulation using gelatin capsules is a novel approach as a seed treatment to enhance plant growth [1]. The effect of gelatin applied at time of sowing on cucumber plant increased leaf surface area and fresh and dry weight as compared to the nontreated control and increased salinity tolerance in plants treated with gelatin capsules (Wilson and Taylor, unpublished). However, the underlying molecular mechanisms of the plant growth promotion by protein hydrolysate are unknown. The objective of this study was to characterize the effect of gelatin capsules on cucumber growth and development at the transcriptome level using mRNA sequencing (RNA-seq) based genome-wide gene expression profiling approach.

## 2. Materials and Methods

### 2.1. Plant Materials and Evaluation of Growth and Nitrogen Content

Cucumber seeds “Vlaspik” (Seminis, Oxnard, CA) were planted in a controlled greenhouse maintained at 24°C/21°C temperature with 14/10-hour photoperiod at New York State Agricultural Experiment Station in Geneva, New York, in the winter of 2013. Two size #3 gelatin capsules (Capsule Line, Pompano Beach, FL) were placed adjacent to each seed in a 4-inch pot (Supplementary Figure 1A); these are referred to as treated plants. Capsules were placed adjacent to the seeds to prevent decrease in germination rate and for controlling the amount of gelatin used in the treatment. Total weight of two gelatin capsules was 93.7 mg with a nitrogen content of 14.2 mg and protein content of 80.0 mg. The nontreated, control plants did not receive gelatin capsule treatment. Thirty-two plants (16 control and 16 treated plants) were placed in the greenhouse following a completely randomized design. First (L1) and second (L2) true leaves from 8 plants in each treatment group were sampled at each of two different time points: one (T1) and two (T2) weeks after emergence. Total leaf area was measured using a CI-202 Leaf Area Meter (CID Bio-Science, Camas, WA) and fresh weight was determined. Samples were flash-frozen in liquid nitrogen and stored at −80°C. Frozen samples were weighed to determine frozen weight (for approximating dry weight). A subsample (~100 mg) of the frozen leaf tissue was placed in an oven to dry at 100°C over night and sent to the Cornell University Stable Isotope Laboratory (Ithaca, NY) for elemental analysis of N by combustion method. All sampling data were then statistically analyzed by ANOVA (*α* = 0.05) and Tukey's HSD test was then used to determine significance.

### 2.2. RNA Isolation and Strand-Specific RNA-seq Library Construction and Sequencing

Three biological replicates of each treatment of each leaf (L1 and L2) at each time point (T1 and T2) were selected for RNA-seq analysis, a total of 24 samples (two treatments, two time points, two leaves, and three biological replicates). Total RNA was isolated from ~5 g of ground leaf tissue using the Spectrum Plant Total RNA Kit (Sigma Aldrich, St. Louis, MO) following the manufacturer's protocol. The isolated RNA quantity and quality were evaluated by electrophoresis (2% agarose gel) followed by ethidium bromide staining and quantification by Nanodrop 1000 (Thermo Scientific, Waltham, MA).

A 5 *μ*g subsample of the isolated total RNA from each sample was treated with 1 *μ*L DNase I (Invitrogen, Carlsbad, CA) incubated at RT for 15 minutes followed by 70°C for 10 minutes. The DNase treated total RNA samples were used to isolate mRNA in order to construct strand-specific RNA-seq library using NEBNext Poly(A) mRNA Magnetic Isolation Module and NEBNext Ultra Directional RNA Library Prep Kit for Illumina (New England Biolabs, Ipswich, MA). Briefly, the mRNA was isolated with 14 *μ*L of NEBNext Magnetic Oligo d(T)_25_ and eluted with 16 *μ*L of Elution Buffer. Five-microliter of the isolated mRNA was fragmented in NEBNext First Strand Synthesis Buffer by heating at 94°C for 15 minutes and then used for the first-stranded cDNA synthesis by reverse transcription using random primers. The synthesized first strand cDNA was then used as a template to synthesize double stranded cDNA. The resulting double stranded cDNA was end-repaired, dA-tailed, and then ligated with NEBNext adaptor. Small size (approximately 300 bp) fragments were selected using Agencourt AMPure XP beads (Bechman Coulter, Pasadena, CA) in 0.6 volumes of ligation reaction. Another round of size selection was performed with 0.25 volumes of the cDNA library solution and then digested with 1 *μ*L of NEBNext USER enzyme at 37°C for 15 minutes. The cDNA library was enriched by PCR with NEBNext index primers following conditions, 98°C for 30 seconds; 15 cycles of 98°C for 10 seconds, 65°C for 30 seconds, and 72°C for 30 seconds; 72°C for 5 minutes, and then held at 4°C. The PCR-enriched cDNA libraries were purified by 1.4 volumes of Agencourt AMPure XP beads and eluted in 22 *μ*L Elution Buffer. The purified cDNA library was analyzed along with Quick-Load 1 kb Extended DNA Ladder (New England Biolabs, Ipswich, MA) by electrophoresis (2% agarose gel) followed by ethidium bromide staining to visualize the optimal size range of the cDNA library (250 bp–400 bp). The cDNA library of optimal sizes was quantified by Qubit 2.0 Fluorometer using the dsDNA HS Assay Kit (Invitrogen, Carlsbad, CA). Three sets of 8 multiplexed libraries (30 ng per library) were configured and sent to Cornell University Biotechnology Resource Center (Ithaca, NY) for single-end sequencing of 101 bases using three lanes (one lane per set of the multiplexed libraries) on Illumina HiSeq 2000 (Illumina, San Diego, CA).

### 2.3. Reads Processing and Data Analysis

The Illumina sequencing generated a total of 377.5 million raw reads passing the Illumina pipeline in software CASAVA v1.8 in Sanger FASTQ format for the 24 samples. On average there were 15.7 ± 6.7 million raw reads per library (Table 1). The RNA-seq data was analyzed with CLC Genomics Workbench (CLC) version 6.5 (CLCBio, Cambridge, MA). The cucumber reference genome (Chinese long, version 2.0) [27] and annotation file were downloaded from the International Cucurbit Genomic Database (http://www.icugi.org/). MapMan gene ontology of the reference transcriptome of 23,248 genes was obtained via the web-based search tool Mercator (http://mapman.gabipd.org/), which searched several databases, including Arabidopsis TAIR 10 (The Arabidopsis Information Resources), SwissProt/Uniref 90 (UniProt Reference Clusters), CDD (Conserved Doman Database, NCBI), KOG (Eukaryotic Orthologous Groups of proteins, NCBI), InterPro (EMBL-EBI), and others.

Reads mapping against the reference genome (Chinese long, version 2.0) was conducted using the following parameters. The limit for read unspecific match to the reference genome was set to 10, minimum length fraction was 0.9, and minimum similarity fraction was 0.98. Normalized read counts RPKM (reads per kilobase exon model per million mapped reads) for each gene was calculated, and genes with RPKM lower than 1.0 were considered not expressed and excluded from analysis. In order to identify differentially expressed genes (DEGs), pairwise comparisons of RPKMs were performed between control and treated plants in each leaf tissue at each time point (control T1-L1 versus treated T1-L1, control T1-L2 versus treated T1-L2, control T2-L1 versus treated T2-L1, and control T2-L2 versus treated T2-L2). The results were weighted by the *t*-type test statistics (*p* value) and FDR (false discovery rate) corrected *p* values using Baggerley's test [28]. The cutoff for defining a DEG is *P*_FDR_ < 0.05.

### 2.4. Gene Network Construction and Visualization

The coexpression gene networks were constructed using weighted gene coexpression network analysis (WGCNA) (v1.41) package in R [29] using square root normalized RPKM data from the 24 samples. A total of 620 differentially expressed genes (DEGs) were used. The adjacency matrix was created by calculating Pearson's correlation between all genes and raised to a default power of 6. The Topological Overlap Measure (TOM), which is a measure of overlap in shared neighbors, was calculated using the adjacency matrix. The dissimilarity TOM was used as input for the dendrogram, and modules were detected using the DynamicTreeCut algorithm. Modules are defined as clusters of highly interconnected genes, and genes within the same cluster have high correlation coefficients among one another [29, 30]. All modules were assigned a unique color (blue, yellow, brown, turquoise, and gray). The module eigengenes were used to represent each module, which were calculated by the first principal component, capturing the maximal amount of variation of the module. The eigengenes were then used to estimate the correlations between module eigengenes and the traits of interest: total leaf area (TLA), fresh weight (FW), frozen weight (DW), and total nitrogen percent (PercentN). The gene network of the blue module was visualized using Cytoscape (v.3.0.0), the most relevant module in explaining the trait variations, such as the effect of the treatments.

### 2.5. Expression Analysis Using qRT-PCR

Expression profile of 5 genes (Csa1G066570, Csa1G590300, Csa5G589260, Csa5G140480, and Csa5G319910) was confirmed in the 12 samples taken at time point 2 using qRT-PCR. A housekeeping gene (Csa6M484600) encoding ACTIN was used as reference. Primers were designed using BatchPrimer 3v1.0 software [31] (Table 2). Genes were selected from the 620 DEGs identified from pairwise comparisons of RPKMs between control and treated plants at each leaf tissue in each time point. The RPKM and qRT-PCR expression values were square root transformed.

The mRNA was purified from 5 *μ*g of total RNA for each of the 12 samples and reverse transcribed in a final volume of 20 *μ*L using the High Capacity cDNA Reverse Transcription Kit (Applied Biosystems, Foster City, CA), according to the manufacturer's protocol. The qRT-PCR was performed using 50 ng of cDNA in a final volume of 20 *μ*L containing 10 *μ*L of LightCycler 480 SYBR Green MasterMix (Roche Applied Science, Germany) and 2 *μ*L of 10 mmol of specific primers (Table 2). Triplicates of each sample were run in the LightCycler 480 instrument (Roche Applied Science, Germany). The total amount of cDNA was normalized by the coamplification of the ACTIN gene and by calibrating the relative expression using the relative quantification method described by Pfaffl [32, 33].

## 3. Results

### 3.1. Evaluation of Growth and Nitrogen Content

**Figure 1:**
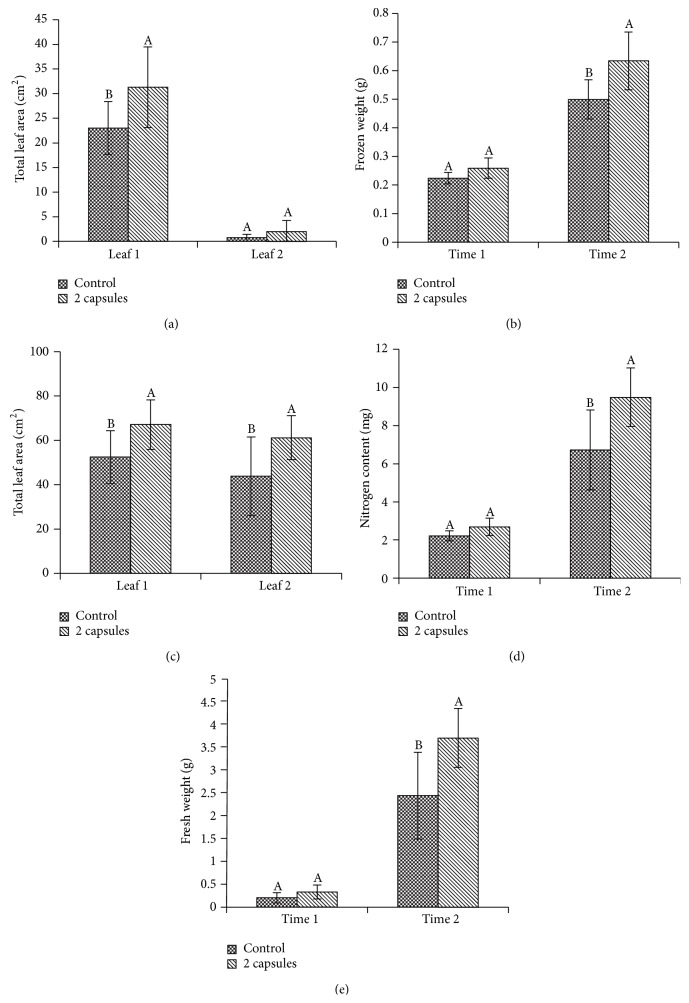
Evaluation of the effect of cucumber (“Vlaspik”) seed gelatin treatment on plant growth and nitrogen content. Data were analyzed by ANOVA (*α* = 0.05), and Tukey's HSD test was then used to compare mean separation. Letters not associated with the same letter were significantly different. Bars represent standard deviation. Legend shows control and gelatin capsule treatment (2 capsules). (a) Total leaf areas at one week after emergence (T1) between control and gelatin capsule treatment (2 capsules). First leaf (leaf 1) and second leaf (leaf 2) were harvested separately. (b) Total leaf areas at two weeks after emergence (T2) between control and gelatin capsule treatment (2 capsules). (c) Fresh weight at one week after emergence (time 1) and two weeks after emergence (time 2). (d) Frozen weight at one week after emergence (time 1) and two weeks after emergence (time 2). (e) Nitrogen contents at one week after emergence (time 1) and two weeks after emergence (time 2).

The growth measurements of the plants treated with gelatin capsules were generally greater than the control in both time points (T1 and T2) (Figures 1(a), 1(b), 1(c), and 1(d)). One week after emergence (T1), there were significant differences between the first leaf (L1) of control and that of plants treated by gelatin capsules, with a 36% increase in total leaf area (*p* = 0.0314) (Figure 1(a)). There were no significant differences in leaf area between the control and the treated plants on the second leaf (L2) (Figure 1(a)). Two weeks after emergence (T2), both L1 and L2 were significantly different between the control and treated plants (*p* = 0.0230 and *p* = 0.0292), respectively (Figure 1(b)). There was a 28% and a 40% increase in leaf area from the control compared to the treated for L1 and L2, respectively (Figure 1(b)).

There were no significant differences in fresh weight one week after emergence (T1) between the control and treated plants. However, a significant difference (*p* = 0.0076) was measured on the second week after emergence (T2) with a 52% increase in the treated plant compared to the control (Figure 1(c)).

Frozen weight also increased with the gelatin capsule treatments, with a 16% increase one week after emergence and a 28% increase two weeks after emergence. There were no significant differences in frozen weight between control and treated plants at T1, but significant differences were measured at T2 (*p* < 0.0076) (Figure 1(d)).

The nitrogen content increase was less significant with only a 4% increase in nitrogen in the treated plants compared to the control one week after emergence and a 12% increase two weeks after emergence. However, there was not a significant increase at T1, but a significant difference in nitrogen content was measured at T2 (*p* < 0.0076) (Figure 1(e)).

### 3.2. Transcriptome Analysis

RNA sequencing of the 24 samples (two treatments, two time points, two leaf types, and three biological replicates) yielded a total of 377.5 million reads, translating to a mean of 15.7 ± 6.7 million reads per sample. Mapping of the 377.5 million reads against the reference genome (Chinese long, version 2.0) indicated that 69% (260.1 million) were mapped of which 98.8% (257.0 million) were mapped to exons and 1.2% (3.1 million) to introns. In this study, 98.0% (255.0 million) of the 260.1 million mapped reads were mapped uniquely to the reference genome (Table 1). The first leaf of the control plants (L1) at T1 had a total of 14,864 expressed genes (RPKM > 1.0), whereas L1 of the treated plants at T1 had the fewest number of expressed genes in the whole group with only 13,885 expressed genes. The samples from L2 in T1 had the highest number of expressed genes, with 15,764 expressed genes in the control and 15,874 expressed genes in the treated plants. The control L1 from T2 had 14,713 expressed genes, and the treated had 14,843 expressed genes. The control L2 from T2 had 14,756 expressed genes, and the treated had 15,287 expressed genes.

### 3.3. Differentially Expressed Genes in Plant Tissue One Week after Emergence (T1)

In this study a cutoff of *p*_FDR_ < 0.05 in Baggerley's test was used to judge the significant differences in gene expression. A total of 22 genes (Supplementary Table 1) were differentially expressed between the treated and the control in the first leaf (L1) one week after emergence (T1). Out of 22 differentially expressed genes (DEG), nine genes (Csa4G622870, Csa2G258100, Csa1G049960, Csa5G623650, Csa4G124910, Csa7G398090, Csa5G171700, Csa3G872080, and Csa5G207960) were downregulated in treated samples (lower expression in the treated compared to the control), and 13 genes (Csa6G109650, Csa2G079660, Csa6G425840, Csa3G120410, Csa7G039260, Csa2G247040, Csa7G447100, Csa7G047970, Csa6G118330, Csa2G355030, Csa6G194150, Csa4G000030, and Csa3G822300) were upregulated (higher expression in the treated compared to the control). The DEGs were involved in development (Csa7G398090 and Csa4G124910), photosynthesis (Csa2G079660), hormone metabolism (Csa2G258100), cell wall degradation (Csa1G049960), signaling (Csa6G194150), and biotic stress (Csa6G425840) (Supplementary Table 1). The second leaf (L2) had 13 DEGs (Supplementary Table 1), two of which were downregulated (Csa5G161900 and Csa3G027190) and ten genes (Csa1G569290, Csa1G569270, Csa6G150530, CsaUNG029290, Csa7G009730, Csa3G047780, Csa2G024440, Csa3G128920, Csa1G172630, Csa1G540870, and Csa5G576740) were upregulated (Supplementary Table 1) in the treated plants as compared with controls. The MapMan bin classification revealed that these DEGs belonged to protein synthesis (Csa1G540870), cell wall related functions (Csa7G009730, Csa6G150530), abiotic stress (Csa1G569270, Csa1G569290), hormone metabolism (Csa2G024440), and photosynthesis (Csa5G576740) (Supplementary Table 1).

### 3.4. Differentially Expressed Genes Two Weeks after Emergence (T2)

Samples from second leaf (L2) at T2 had 588 DEGs between treatment and controls, of which 146 were downregulated in the treated, and 442 were upregulated (Supplementary Table 1) despite the fact that the first leaf (L1) revealed no DEG at the set threshold value. The MapMan bin classification revealed that the largest function categories were protein synthesis, transcription factor, transport, hormone, and secondary metabolism, as well as lipid metabolism. The list of DEGs at T2 (588 genes) was much more extensive compared to that of T1 (22 genes). Despite the limited number of DEGs at T1, there were six overlapping DEGs between T1 (L1) and T2 (L2), including Csa2G247040, Csa2G355030, Csa4G000030, Csa6G109650, Csa6G425840, and Csa7G398090 (Supplementary Table 1).

### 3.5. Identification of Coexpression Gene Modules and Network Hub Genes Highly Associated with Enhanced Growth by Gelatin Seed Treatment

**Figure 2:**
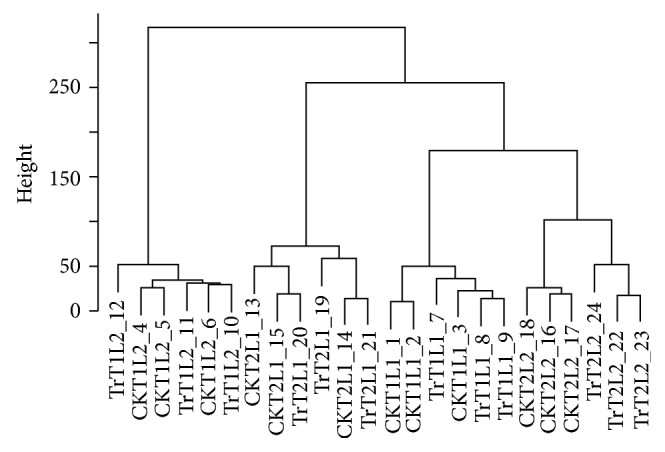
Cluster dendrogram illustrating the relationships among the leaf tissues harvested at different time points (T1/T2), leaf position (L1/L2) of control plants (CK), and plants treated with gelatin capsule (Tr), which were calculated based on the expression data of the 620 DEGs.

Weighted gene coexpression network analysis (WGCNA) of the 24 RNA-seq samples using the 620 DEGs (Supplementary Table 1) showed four clusters along the lines between two time points and two leaf positions, that is, T1-L1, T1-L2, T2-L1, and T2-L2 (Figure 2). There was no subclustering by the treatment for T1-L1, T1-L2, and T2-L1. However, for T2-L2, the subclustering is clearly separated according to the treatment, and all control samples are clustered under one branch, which are separated from the treated samples, reflecting the fact that 588 of the 620 genes were DEGs between the control and the treated in T2-L2 (Figure 2).

**Figure 3:**
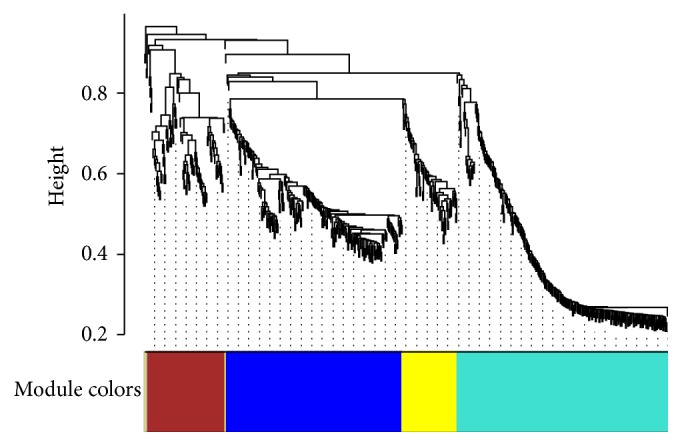
Hierarchical cluster tress showing coexpression modules identified by WGCNA. The major branches constitute 5 modules labeled by different colors (grey module is for genes unassigned). Each branch in the dendrogram constitutes a module and each leaf in the branch represents one gene.

The WGCNA analyses further identified that the 620 DEGs were clustered into five modules, which are labeled by colors: blue, yellow, brown, turquoise, and gray (Figure 3). There were 208 genes in the blue module, 90 genes in the brown module, 249 genes in the turquoise module, 66 genes in the yellow module, and 4 genes in the grey module (for unassigned genes) (Supplementary Table 1).

**Figure 4:**
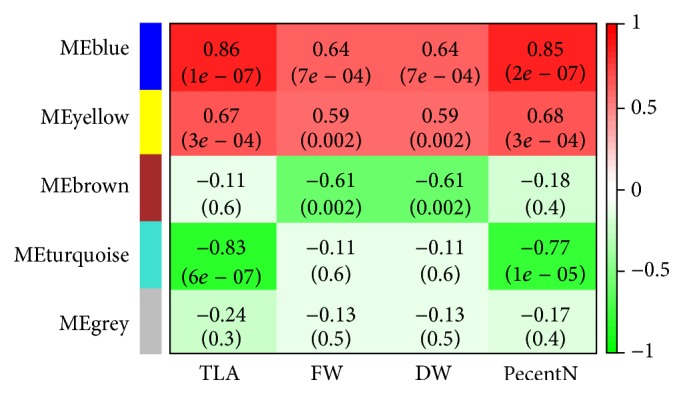
Module-trait association. Each row corresponds to a module and each column corresponds to a specific trait: total leaf area (TLA), fresh weight (FW), frozen weight (DW), and percent nitrogen (PercentN). The color of the cell indicates the correlation coefficient between the module and trait, dark red color indicates high degree of positive correlation, and dark green indicates high degree of negative correlation between each module and trait. The numbers in the cell indicate the module-trait correlation value (top) and *p* value (below).

The module-trait (TLA, FW, DW, and PercentN) relationship (Figure 4) revealed that the blue module had a strong positive correlation with the leaf area (TLA; *R* = 0.86; *p* < 1 × 10^−7^) and nitrogen percent (PercentN; *R* = 0.85; *p* < 2 × 10^−7^) and a moderate positive correlation with fresh weight (FW; *R* = 0.64; *p* < 7 × 10^−4^) and frozen weight (DW; *R* ^2^ = 0.64; *p* < 7 × 10^−4^). The yellow module had a moderate positive correlation with all the traits. The turquoise module on the other hand had a strong negative correlation with TLA (*R* = −0.83; *p* < 6 × 10^−7^) and PercentN (*R* = −0.77; *p* < 1 × 10^−5^) and the brown module showed a moderate negative correlation with FW (*R* = −0.61; *p* < 0.002) and DW (*R* = −0.61; *p* < 0.002) (Figure 4).

**Figure 5:**
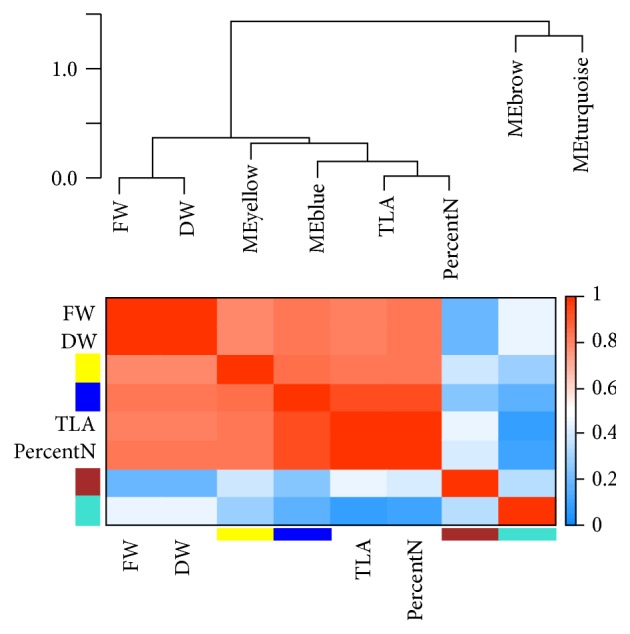
Module eigengene network and its relationship with growth traits. The dendrogram on the top illustrates the relationship among the module eigengenes and traits total leaf area (TLA), fresh weight (FW), frozen weight (DW), and percent nitrogen (PercentN). Each row and column on the heat map correspond to a module or a trait. The color of the cell indicates the adjacencies among the module eigengenes and traits; dark red color indicates close ardencies and dark blue no or little adjacencies.

To study the relationships among modules and how the modules were related to the four growth traits, an eigengene network was inferred by calculating their adjacencies (Figure 5). The network indicated that there were two major branches: one comprising the blue and yellow module eigengenes and the other comprising the brown and turquoise module eigengenes. The four growth traits were collectively determined to be closer to the blue and yellow module eigengenes than to those of the brown and turquoise modules eigengenes. The FW and DW were extremely close to each other and were nearly equally related to the eigengenes of blue module and those of yellow module. However, the TLA and PercentN were highly related to each other and were markedly close to the blue module eigengenes. These results strongly suggest that the blue module eigengenes were most closely related to the four growth traits; therefore the blue module was determined to be of the greatest importance and was investigated in more detail (Figure 5).

**Figure 6:**
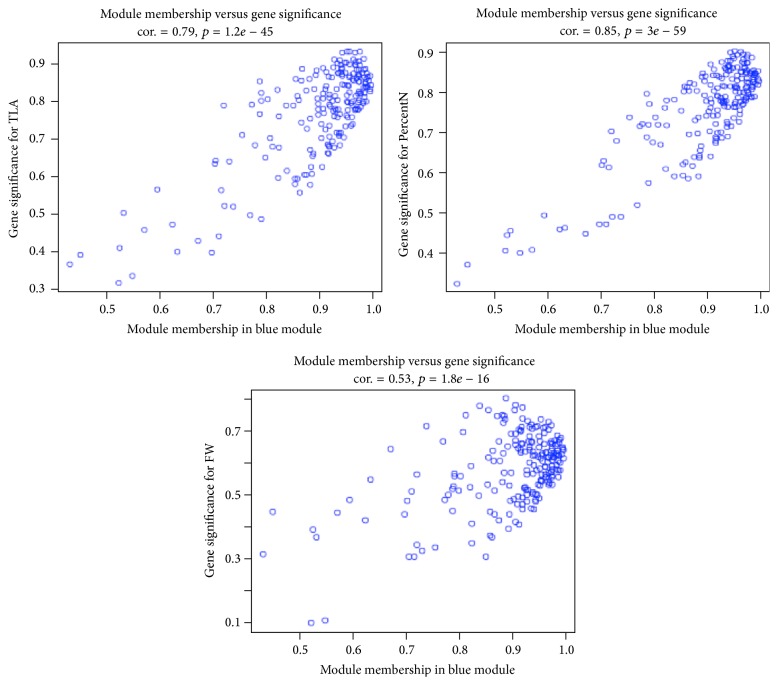
Intramodular analysis of gene significance (GS) and module membership (MM) of the genes in the blue module. Data for trait DW was not included, which is nearly identical to that of FW.

The intramodular analysis of gene significance (GS), the absolute value of the correlation between the gene and the trait, and module membership (MM) and the correlation of the module eigengene and the gene expression profile of the 208 genes in the blue module identified a number of genes of high significance for the growth traits as well as high module membership in the module (Figure 6). The data suggested that GS and MM were highly correlated with all four growth traits, especially for PercentN and TLA (Figure 5).

**Figure 7:**
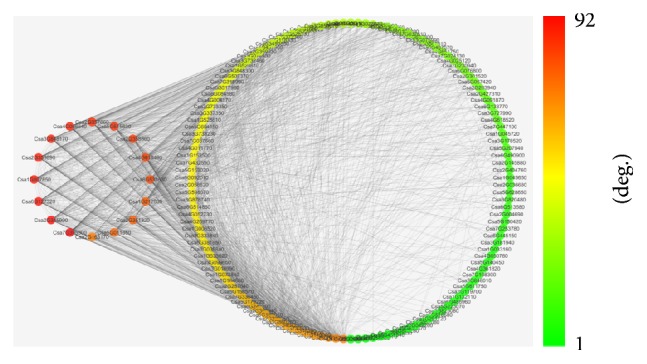
Visualization of a coexpression network of 150 of 208 genes in the blue module. The network TOM similarity was calculated with a power of 30 and edges are of weight higher than 0.25. The 16 hub genes of the highest network degree (60–92) are shown on the left panel of the figure.

For better visualization of the intramodular connectivity (network degree), the gene network in the blue module was exported into Cytoscape 3.1 (Figure 7). Network analysis using Network Analyzer [34], a Cytoscape plugin, identified 16 network hub genes of the highest network degree (60–92) in the blue module: Csa2G357860, Csa5G615830, Csa2G358860, Csa4G618490, Csa6G538630, Csa1G212830, Csa2G351820, Csa5G011650, Csa2G163170, Csa7G395800, Csa2G345990, Csa6G127320, Csa1G007850, Csa2G359890, Csa3G848170, and Csa4G056640 (Figure 7, Table 3). Of these network hub genes, five are of particular interest, including Csa6G127320 encoding a NAC transcription factor, Csa1G007850 a MYB transcription factor, Csa5G615830 an amino acid transporter, Csa2G163170 a plasma membrane localized ammonium transporter, and Csa7G395800 a xenobiotic detoxifier-glutathione S-transferase (Figure 7, Table 3).

### 3.6. Expression Analysis Using qRT-PCR

**Figure 8:**
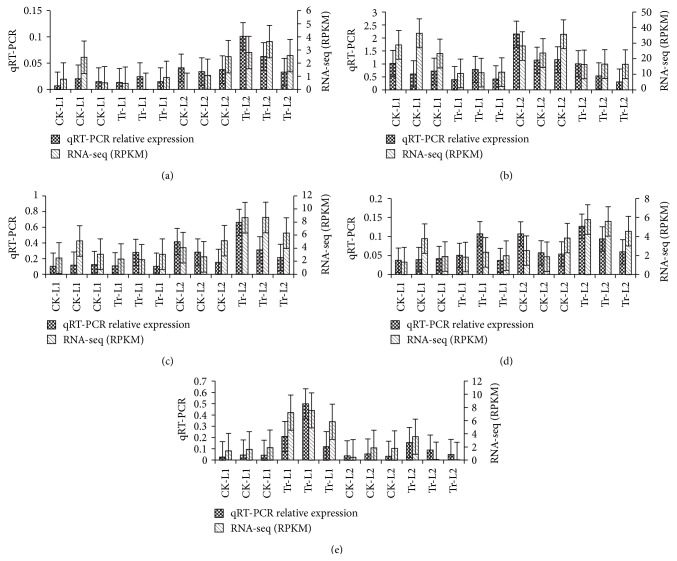
Comparison of gene expression between RNA-seq and qRT-PCR. The data were transformed by square root. Bars represent standard deviation. (a) Csa1G066570 (*R* ^2^ = 0.597, *p* = 0.0032), (b) Csa1G590300 (*R* ^2^ = 0.519, *p* = 0.0082), (c) Csa5G589260 (*R* ^2^ = 0.483, *p* = 0.0121), (d) Csa5H140480 (*R* ^2^ = 0.798, *p* = 0.0001), and (e) Csa5G319910 (*R* ^2^ = 0.591, *p* = 0.0035).

Five genes (Csa1G066570, Csa1G590300, Csa5G589260, Csa5G140480, and Csa5G319910) were selected from the 620 DEGs for the qRT-PCR validation of their expression levels measured by RNA-seq (RPKM). Correlation analysis between the qRT-PCR relative expression value and the RPKM of RNA-seq indicated that they were significantly correlated (*R* ^2^ = 0.48–0.79, *p* = 0.0121–0.0001), validating the expression levels measured by RNA-seq (Figures 8(a)–8(e)).

## 4. Discussion

### 4.1. Amino Acid and Nitrogen Transport

The increased expression of nitrogen source transporters in plants treated with gelatin capsules provided an insight into the mechanism of amino acid transport from the gelatin capsule to the plant as well as the nitrogen translocation within the plant. These transport proteins include amino acid transporter (Csa5G615830; Table 3), amino acid permease 3 (AAP3; Csa6G381850; Supplementary Table 1), amino acid permease 6 (AAP6; Csa3G894480; Supplementary Table 1), ammonium transporter 1;1 (AMT1;1; Csa2G163170; Table 3), and ammonium transporter 2 (AMT2; Csa3G730930; Supplementary Table 1).

Gelatin capsules are composed of hydrolyzed collagen, a polypeptide found primarily in the flesh and connective tissues of animals [35, 36]. Collagen can be broken down through hydrolysis to form hydrolyzed collagen, and further hydrolysis will break down the peptide to single amino acids [35, 37]. Plants are capable of acquiring nitrogen as nitrate (NO_3_^−^) and ammonium (NH_4_^+^), but also as organic forms such as amino acids and proteins from the soil [38]. Assimilation pathways of amino acids have been demonstrated with ^15^N-labelled amino acids [39, 40], as well as through metabolic profile analysis using GC-MS [41, 42]. Transport studies with plant tissues have demonstrated the presence of multiple transport systems for amino acids in plants [43–46]. The amino acids enter the xylem in roots [47] and are exported from the xylem to the surrounding tissue [48]. The amino acids are then transported from xylem to the phloem, leading to cycling within the plant [48].

One of the amino acid transporters upregulated with the gelatin capsule treatment, AAP3, is preferentially expressed in the phloem and has been associated with long distance transport of basic amino acids such as arginine, histidine, and lysine [49–51]. The long distance transport by AAP3 has been attributed to amino acid loading from the apoplast into the phloem [46, 48, 49]. AAP6, on the other hand, is expressed mainly in xylem parenchyma cells in sink tissues such as sink leaf, cauline leaf, and roots [52]. Unlike AAP3, which is responsible for high affinity transport of basic amino acids, AAP6 is a high affinity transporter of acidic and neutral amino acids such as aspartic acid, proline, alanine, and valine [48, 51, 52]. The relatively low concentration of amino acids in the xylem compared to that in the phloem sap means that a high affinity transporter would be necessary in the xylem parenchyma cells to mediate the amino acid transfer from the xylem to the phloem [48]. The high affinity nature of AAP6, as well as its expression in the xylem parenchyma cells, suggests that AAP6 plays a role in the xylem to phloem transfer of amino acids. Hunt et al. [53] demonstrated that AAP6 mutant (aap6) had a reduced amino acid content in the sieve element sap.

The increased expression of amino acid transporters AAP3 (Csa6G381850) and AAP6 (Csa3G894480) in the plants treated with gelatin capsules suggests that amino acid transport in the plants was positively affected by the treatment, possibly by the amino acids from the treatment being taken up by the plants. Wang et al. [42] demonstrated that in pak choi (*Brassica rapa* var.* chinensis*) amino acid supply was considered an N-limiting condition compared to N source from NO_3_^−^, which further enforces the effect of availability of amino acids in plant growth promotion.

AAPs are selectively expressed in areas where expression of H^+^ATPases is localized, as AAPs are driven by a proton gradient and require a high proton concentration to fuel the active transport involved in uptake of amino acids from the apoplast into the phloem [48, 54, 55]. Expression of both AAP3 and AAP6 is localized in the plasma membrane [48], which is where the expression of H^+^ATPase 4 (AHA4; Csa1G008520; Supplementary Table 1), another gene highly expressed in plants treated with gelatin capsules, is found to be localized [55]. Expression of AAP1, AAP2, and AAP8 correlates with the expression pattern of two H^+^ATPase genes, AHA10 and AHA3 [56, 57]. This suggests coupling of H^+^ATPases with AAPs, which have been characterized as proton symporters energized by H^+^ATPase [58, 59]. It is possible that AAP3 and AAP6 transport activity is coupled with AHA4, generating proton motive force required for high affinity transport of the amino acids between xylem and phloem; however, further examination is necessary to link these genes together in a concrete manner.

AMT2 (Csa3G730930; Supplementary Table 1) and AMT1;1 (Csa2G163170; Table 3) were another two genes involved in nitrogen metabolism that showed high expression in plants treated with gelatin capsule and were also shown to be in the same module (blue) in WGCNA analysis. Ammonium is an important intermediate in nitrogen metabolite, and the transport of and distribution of NH_4_^+^ across cellular membranes depend on ammonium transporters (AMT). In plants, AMT-mediated ammonium transport is critical for providing sufficient nitrogen for optimal growth [60, 61]. GFP studies with AMT2 demonstrated its expression in the vascular tissue in roots, pith of stem, petiole, and leaf hydathodes, and it mediated electroneutral ammonium transport in the form of NH_3_ [61]. This is especially advantageous for the plant as there is no need to actively export cotransported protons by energy consuming H^+^ pumps, allowing AMT2 to function as a high affinity NH_4_^+^ transporter in the plasma membrane [61–63]. Root AMT2 expression has been reported to be regulated by N-supply by Sohlenkamp et al. [62], in which the AMT2 transcript increased in roots after N-deprivation; however, transcript level in shoots remained unchanged. The increase in AMT2 transcript in plants treated with gelatin capsules suggests an increase in N-transport, exporting NH_4_^+^ from the vascular tissue and importing NH_3_ into the cytoplasm. The increase in total N observed in plants treated with gelatin capsules is hypothesized to be due to the increase in N-assimilation. However, none of the genes associated with N-assimilation (GS/GOGAT) were differentially expressed in the plants treated with gelatin capsules. As the GS/GOGAT system is dependent upon the N-concentration in the tissue, it may be that the concentration of NH_3_ in the tissue was not high enough to upregulate gene involved in N-assimilation.

### 4.2. NAC Transcription Factors

NAC (NAM, ATAF1,2, and CUC2) proteins are one of the largest families of plant-specific transcription factors, and the family is present in a wide range of plants, with more than 100 predicted members in* Arabidopsis thaliana* [64, 65]. Due to such large number, only a small portion of the NAC proteins has been characterized, and yet the family has been implicated in various plant processes including developmental programs, defense, and abiotic responses [65]. NAC proteins commonly have highly conserved sequences named NAC domains in their N-terminal regions [66]. NAC domains consist of five subdomains (A–E) [67], and subdomains D and E are required for DNA-binding, while the C-terminal regions can function as a transcriptional activation domain [64, 68]. NAC proteins have been classified into 18 subgroups by similarity in the amino acid sequence in the NAC domains [69]. The NAC036 gene (Csa6G127320, Table 3) is one of the network hub genes identified in the WGCNA analysis and is a member of the ONAC022 subgroup. RNA gel blot analysis has revealed that NAC036 gene is strongly expressed in leaves, and plants overexpressing NAC036 gene have a semidwarf phenotype [64]. Kato et al. [64] through microscopic observation determined that dwarfing in these plants was due to reduction in cell size; however, the mechanism by which overexpression of NAC036 gene causes reduction of cell size is unclear. Microarray data has confirmed that expression of NAC036 gene was induced by abiotic stress such as osmotic stress and salt stress, which lead the authors to speculate that the overexpression of NAC036 leads to excessive stress responses, which subsequently compromises cell growth. In this study, NAC036 expression was downregulated in the treated plants in T2-L2, which agrees with the leaf area data, in which there was an increase in leaf area in the treated plants in T2-L2. It is possible that the NAC036 genes are involved in other abiotic stress response pathways, such as ABA, SA, and JA; however, this relationship is unclear without further investigation.

### 4.3. Detoxifying Proteins

Plants are continually exposed to potentially toxic chemicals (xenobiotic). These chemicals cannot be used for nutrition or source of energy and thus plants have found a way to remove such compounds from their system by either storing in a secure place (i.e., vacuole) or destroying them by biodegradation [70]. The process of chemical modification of xenobiotic compounds in plants is classified by three phases: phase I (activation reaction, which involves hydrolysis or oxidation), phase II (conjugation reaction), and phase III (compartmentalization and processing). In phase I, the hydrolysis reactions are catalyzed by esterases, but most of the reactions are oxidation, which are catalyzed by cytochrome P450 system [70]. In phase II, the phase I activated metabolites are deactivated by covalent linkage to an endogenous molecule such as glutathione, to form a water-soluble conjugate [71]. Glutathione S-transferase (GST), for instance, is a soluble enzyme that is often thought of as detoxification enzyme with the ability to metabolize a wide variety xenobiotics via GHS conjugation [71–73]. GSTs are grouped into four classes: phi, zeta, tau, and theta. Zeta and theta GST are found in both animals and plants, but tau and phi classes are only found in plants [73]. Phi and tau GSTs are induced after exposure to biotic and abiotic stresses. The GST phi 8 (GSTF8; Csa7G395800; Table 3) was found to be highly associated with plant treated with gelatin capsules which was upregulated in the second leaf of the treated plants, during the second week after emergence (T2-L2). The hub gene, GSTF8, is used as a marker for early stress and defense responses. GSTF8 expression can be induced by a range of biotic and abiotic stresses including SA, auxin, herbicides, and microbial infections [74]. The endogenous function of GSTF8 is still poorly understood. It is cytosolic and highly expressed in roots, where it may act to detoxify products of oxidative stress resulting from pathogen attach or foreign chemicals in the soil [74].

The inactive, water-soluble conjugates formed in phase II are then exported from the cytosol by membrane-located transport proteins such as plasma membrane intrinsic protein 1C (PIP1C; Csa5G53020; Supplementary Table 1) which initiate phase III, the compartmentalization and processing of the detoxification system [70]. The transport of glutathione conjugates from the cytosol to the vacuole requires passing through the tonoplast. The tonoplast intrinsic proteins and ABC transporters are thought to be involved in this process. There were numerous genes associated with detoxifying function in the list of genes that are highly associated with gelatin capsule treatments (Supplementary Table 1), such as ABC protein 9 (NAP9; Csa1G181360), tonoplast intrinsic protein 2;3 (TIP2;3; Csa7G447100), and tonoplast intrinsic protein 2 (TIP2; Csa6G486670). The NAP9 and TIP2 genes were upregulated in the second leaf of the treated plant during the second week after emergence (T2-L2), whereas TIP2;3 was upregulated in the first leaf during the first week after emergence in the treated plants. ABC transporter superfamily mediates excretion of potentially toxic compounds, conferring heavy-metal tolerance [75]. The UDP-glycotransferase (UGT; Csa4G618520; Supplementary Table 1), a gene involved in biodegradation of xenobiotics, is one of the genes highly associated with gelatin capsule treatment, which further supports the hypothesis that gelatin capsule treatment increases the expression of genes associated with detoxification, which allows for increased plant growth and accumulation of biomass. The UGT gene was upregulated in the second leaf during the second week after emergence in the treated plants. It is possible that GSTF8 and other GSTs that are upregulated in plants treated with gelatin capsule treatments are involved in detoxifying products of oxidative stress in the root, which allows for efficient uptake of amino acids and nitrogen from the soil.

## 5. Conclusions

In this study we confirmed that gelatin treatment of seeds enhanced the growth of cucumber seedlings although the enhancement effect was more significant in two-week-old plants than in one-week-old ones. Based on comparative RNA-seq data analyses, the beneficial effect of seed gelatin treatment could be defined by the differential expression of the 620 DEGs between the treated and nontreated control. Further analysis of the 620 DEGs using WGCNA suggested that the blue coexpression gene network module of 208 of 620 DEGs is the most relevant for the gelatin growth enhancement, which is featured with 16 most interconnected hub genes. Members of the 16 hub genes include a NAC transcription factor (Csa6G127320), a MYB transcription factor (Csa1G007850), an amino acid transporter (Csa5G615830), an ammonium transporter (Csa2G163170), a xenobiotic detoxifier-glutathione S-transferase (Csa7G395800), and others. These results suggested that the increased expression of amino acid and nitrogen transporter genes and the xenobiotic detoxification system involved genes and their possible transcriptional regulation through the two transcription factors might be an important mechanism explaining the enhanced growth and increased abiotic stress tolerance of gelatin seed treatment.

## Supplementary Material

Supplementary Figure 1: Pictures of seed encapsulation of various seeds A) pelleted and B) multiple seeds as used commercially by Alliance Seed Capsules. C) Picture of the representation of how the gelatin capsules were used in the experiment, placed adjacent to the seed when planted in the pots
Supplementary Table 1: List of all differentially expressed genes from both first and second leaves in the two time points sampled between control and gelatin capsule treated plants that show high correlation to the growth parameters. FDR p-value has been corrected by Baggerley's test, and Mapman classification is used for gene ontology. Annotation contains BLAST annotation and e-value, a parameter that describes the number of hits one can expect to see by change when searching a database. The module color refers to the module in which the gene is classified to.

## Figures and Tables

**Table 1:** Overview of mapping of RNA-seq reads against the reference genome.

Sample	Treatment	Time point/leaf	Rep.	Number of Total reads	Uniquely mapped reads	Percent read uniquely mapped to reference (%)	Percent read uniquely mapped to exons (%)	Percent read uniquely mapped to introns (%)
1	Control	T1-L1	1	12,019,363	9,350,227	77.79	98.71	1.29
2	Control	T1-L1	2	8,624,277	6,592,181	76.44	98.61	1.39
3	Control	T1-L1	3	14,970,567	10,499,989	70.14	99.36	0.64
4	Control	T1-L2	1	10,128,190	7,724,322	77.03	98.73	1.27
5	Control	T1-L2	2	9,216,330	6,472,955	70.23	98.33	1.67
6	Control	T1-L2	3	14,144,786	9,498,041	67.15	98.86	1.14
7	Treated	T1-L1	1	17,361,035	13,764,974	79.29	99.10	0.90
8	Treated	T1-L1	2	8,794,498	4,518,865	51.38	98.99	1.01
9	Treated	T1-L1	3	16,744,466	12,843,001	76.70	99.17	0.83
10	Treated	T1-L2	1	21,897,381	16,628,402	75.94	98.64	1.36
11	Treated	T1-L2	2	8,393,223	5,533,323	65.93	98.58	1.42
12	Treated	T1-L2	3	16,743,616	12,757,850	76.20	98.09	1.91
13	Control	T2-L1	1	13,141,492	10,676,776	81.24	98.62	1.38
14	Control	T2-L1	2	13,736,384	10,695,841	77.87	98.61	1.39
15	Control	T2-L1	3	23,334,193	17,068,298	73.15	98.98	1.02
16	Control	T2-L2	1	12,638,497	10,042,325	79.46	99.19	0.81
17	Control	T2-L2	2	16,590,125	2,877,719	17.35	99.06	0.94
18	Control	T2-L2	3	32,818,854	23,045,970	70.22	99.17	0.83
19	Treated	T2-L1	1	8,454,166	5,946,132	70.33	98.50	1.50
20	Treated	T2-L1	2	14,493,239	11,039,888	76.17	98.61	1.39
21	Treated	T2-L1	3	25,311,395	19,265,850	76.12	98.53	1.47
22	Treated	T2-L2	1	8,279,148	6,515,229	78.69	99.11	0.89
23	Treated	T2-L2	2	25,182,470	4,169,826	16.56	98.99	1.01
24	Treated	T2-L2	3	24,438,920	17,400,788	71.20	98.00	2.00

Total				377,456,615	254,928,772			
Mean				15,727,359	10,622,032	68.86	98.77	1.23
SD				6,671,306	5,195,189	17.15	0.35	0.35

**Table 2:** qRT-PCR primer sequence (forward and reverse) by gene (feature ID).

Feature ID	Forward (5′-3′)	Reverse (5′-3′)
ACT	CCGTTCTGTCCCTCTACGCTAGTG	GGAACTGCTCTTTGCAGTCTCGAG
Csa1G066570	TGAGCAATGCCCTGTCCAAC	ATCACCTTCCTGGCCCACAA
Csa1G590300	ATTGCTGGCGTTGTCACTGG	TCCCAAGCATGGCTTCACAA
Csa5G140480	CTGCATTTCCCCGGATTCTG	GCGGGGTCGAACTTGTCATC
Csa5G319910	AGCCCATGTGCCTTCGTTGT	GATCGCCTGTTCCCCGATTT
Csa5G589260	GGTGTTGGCCGGTTTTTGAA	CTTTCAGTGCGGCAGCTCCT

**Table 3:** List of 16 network hub genes identified in the blue module. Degree represents the number of edges between genes, which represent coexpression correlations.

Gene	MapMan gene ontology	Annotation	Degree
**Csa7G395800**	Miscellaneous	Glutathione S-transferase PARB	92
Csa2G345990	Not assigned	Unknown protein	90
**Csa6G127320**	Development	NAC domain containing protein 36 (NAC036)	89
**Csa1G007850**	Transcription factor	Myb-like DNA binding domain	87
Csa2G359890	Not assigned	Unknown protein	86
Csa4G056640	Hormone metabolism	Nine-cis-epoxycarotenoid dioxygenase 4 (NCED4)	85
Csa3G848170	Protein degradation	Aminopeptidase M1 (APM1)	85
Csa2G357860	Not assigned	Unknown protein	81
**Csa5G615830**	Transport	Transmembrane amino acid transporter family protein	81
Csa2G358860	Not assigned	Unknown protein	77
Csa4G618490	Transport	Zinc transporter 5 precursor (ZIP5)	75
Csa6G538630	Cofactor and vitamin metabolism	Molybdenum cofactor sulfurase family protein	74
Csa2G351820	Protein targeting	Vacuolar-processing enzyme precursor	68
Csa1G212830	Secondary metabolism	2-oxoglutarate (2OG) and Fe(II)-dependent oxygenase superfamily protein	68
Csa5G011650	Miscellaneous	NAD(P)-binding Rossmann-fold superfamily protein	66
**Csa2G163170**	Transport	Ammonium transporter 1;1 (AMT1;1)	60
