# Supplementary material for: Transcriptome Analysis of Gelatin Seed Treatment as a Biostimulant of Cucumber Plant Growth
Source: ScientificWorldJournal. 2015 Oct 8;2015:391234. doi: 10.1155/2015/391234 (PMC4617879; doi:10.1155/2015/391234)
Supplement: Supplementary file 1 — Supplementary Figure 1: Pictures of seed encapsulation of various seeds A) pelleted and B) multiple seeds as used commercially by Alliance Seed Capsules. C) Picture of the representation of how the gelatin capsules were used in the experiment, placed adjacent to the seed when planted in the pots Supplementary Table 1: List of all differentially expressed genes from both first and second leaves in the two time points sampled between control and gelatin capsule treated plants that show high correlation to the growth parameters. FDR p-value has been corrected by Baggerley's test, and Mapman classification is used for gene ontology. Annotation contains BLAST annotation and e-value, a parameter that describes the number of hits one can expect to see by change when searching a database. The module color refers to the module in which the gene is classified to. [file 391234.f1.pdf]

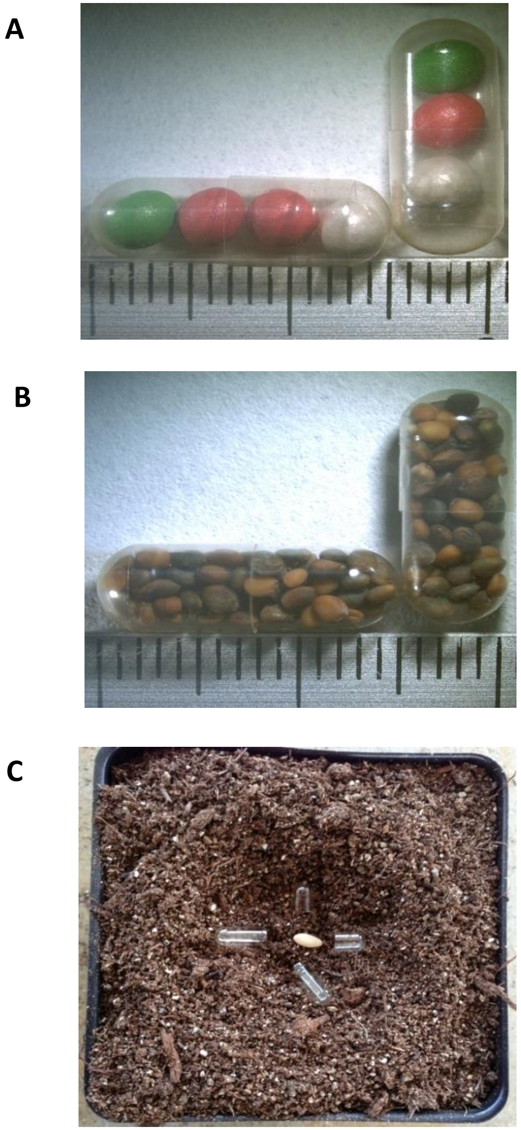

Supplementary Figure 1. Gelatin treatment of seeds. (A) Seed Encapsulation of pelleted seeds. (B) Seed Encapsulation of multiple seeds (C) Two gelatin capsules (shown in four halves) placed adjacent to a seed in a 4-inch pot.

Supplementary Table 1: List of DEG in the first leaf (L1) and second leaf (L2) in first time point (T1), and second leaf (L2) in second time point (L2) between control and gelatin capsule treatment that show high correlation to the growth parameters (TLA, FW, PercentN). FDR p-value ha been corrected by Baggerley's test. Mapman classification is used for gene ontology (GO). Annotation contains annotation from BLAST and the e-value, which is parameter that describes the number of hits one can "expect" to see by chance when searching a database. The module color refers to the module in which the gene is classified to.

Supplementary Table 1 (Continued)

Differentially expressed	Module Color	Gene	Fold change	Baggerley's test: FDR p-value correction	MapMan GO Classification	Annotations	e-value
T1-L1	blue	Csa4G431960	3.195	0	Protein targeting	Sigma factor binding protein 1 (SIB1)	2.00E-14
T1-L1	blue	Csa2G072490	1.618	0.049	Protein degradation	Insulinase (Peptidase family M16) family protein	0
T1-L1	blue	Csa3G782680	1.16	1.20E-04	Cell vesicle transport	Syntaxin related protein 1 (SYR1)	7.00E-123
T1-L1	brown	Csa4G124910	1.404	0.000006196	Miscellaneous	Polyamine oxidase 2 (PAO2)	3.00E-228
T1-L1	brown	Csa3G120410	3.575	0.00005376	Transcription factor	WRKY DNA-binding protein 53 (WRKY53)	1.00E-102
T1-L1	brown	Csa7G448050	NA	Not assigned	unknown		
T1-L1	brown	Csa2G247040	1.987	9.97E-04	RNA binding	Glycine rich protein 7 (GRP7)	1.00E-58
T1-L1	brown	Csa4G000030	1.544	1.91E-03	Not assigned	DUF1005	1.00E-118
T1-L1	brown	Csa6G109650	-1.75	1.71E-05	Miscellaneous	Bi-functional inhibitor/lipid-transfer protein/seed storage 2S albumin superfamily protein	6.00E-41
T1-L1	brown	Csa6G425840	3.098	6.03E-08	Biotic stress	Late embryogenesis abundant (LEA) hydroxyproline-rich glycoprotein family	2.00E-65
T1-L1	turquoise	Csa7G047970	2.18	0.0009962	Transport	MATE efflux family protein	4.00E-205
T1-L1	turquoise	Csa5G207960	2.293	3.454E-10	Transcription factor	Salt-inducible zinc finger 1 (SZF1)	1.00E-116
T1-L1	turquoise	Csa6G194150	5.147	1.779E-09	Cell wall	Expansin A4 (EXPA4)	2.00E-137
T1-L1	turquoise	Csa2G355030	1.363	4.70E-03	Transcription factor	MYB domain protein 77 (MYB77)	4.00E-115
T1-L1	turquoise	Csa7G398090	1.705	0.041	Development	Senescence-associated gene 21 (SAG21)	2.00E-20
T1-L1	yellow	Csa6G118330	1.98	4.437E-10	Amino acid metabolism	Embryo defective 1017 (emb1075)	7.00E-235
T1-L1	yellow	Csa5G623650	2.467	7.05E-03	Not assigned	unknown	
T1-L2	blue	Csa1G533620	-2.242	5.36E-03	Not assigned	unknown	
T1-L2	blue	Csa6G517180	1.68	0.000001159	Miscellaneous	alpha/beta-hydrolases superfamily protein	1.00E-128
T1-L2	blue	Csa5G523190	5.13	0.00002257	Redox	Glutaredoxin	9.00E-41
T1-L2	blue	Csa3G016990	1.432	6.40E-03	Development	unknown	1.00E-23
T1-L2	turquoise	Csa5G161900	-2.457	1.80E-06	Miscellaneous	Plant invertase/pectin methylesterase inhibitor superfamily protein	3.00E-24

Supplementary Table 1 (Continued)

T1-L2	turquoise	Csa1G569290	1.313	3.46E-10	Abiotic stress	A member of heat shock protein 90 (HSP90) gene family (*Arabidopsis thaliana*)	0
T1-L2	turquoise	Csa1G569270	2.042	7.34E-05	Protein synthesis	Ribosomal protein L18e/L15 superfamily protein	0
T2-L2	blue	Csa4G638480	-71.384	1.63E-03	Biotic stress	ADR1-like 1 (ADR1-L1)	8.00E-257
T2-L2	blue	Csa1G231530	-5.347	1.18E-04	Hormone metabolism	Indole-3acetic acid inducible 12 (IAA12)	
T2-L2	blue	Csa5G139420	-3.252	4.53E-05	Protein degradation	Prolyl oligo-peptidase family protein	3.00E-69
T2-L2	blue	Csa7G336450	-3.105	5.63E-05	Not assigned	unknown	
T2-L2	blue	Csa6G517960	-2.436	7.19E-06	Development	Squamosa promoter binding protein-like 8 (SPL8)	4.00E-162
T2-L2	blue	Csa3G337350	-2.409	5.31E-05	Biotic stress	MLP-like protein 34 (MLP34)	2.00E-26
T2-L2	blue	Csa1G524760	-2.299	7.66E-05	Hormone metabolism	highly ABA-induced PP2C gene 2 (HAI2)	
T2-L2	blue	Csa3G062590	-2.236	2.65E-10	Transcription factor	NAC domain transcription factor family	1.00E-50
T2-L2	blue	Csa5G522970	-2.079	4.50E-04	Miscellaneous	Beta-1,4-N-acetylglucosaminyltransferase family protein	4.00E-144
T2-L2	blue	Csa2G263940	-2.007	7.86E-09	Cell wall	Proline-rich protein 4 (PRP4)	6.00E-65
T2-L2	blue	Csa2G357860	-1.953	1.01E-06	Not assigned	unknown	
T2-L2	blue	Csa6G076800	-1.921	8.36E-03	Cell wall	Fasciclin-like arabinoglaaractan protein 8 (FLA8)	2.00E-54
T2-L2	blue	Csa6G448110	-1.869	1.83E-05	Transport	Gamma tonoplast intrinsic protein (GAMMA-TIP)	2.00E-67
T2-L2	blue	Csa3G073930	-1.822	7.68E-04	Protein degradation	Small ubiquitin-like modifier 1 (SUMO1)	1.00E-32
T2-L2	blue	Csa3G690300	-1.686	4.28E-05	Not assigned	unknown	
T2-L2	blue	Csa7G108300	-1.647	9.38E-06	Cell wall	Fasciclin-like arabinogalaractan protein 1 (FLA1)	9.00E-70
T2-L2	blue	Csa5G598070	-1.634	1.24E-07	Not assigned	unknown	
T2-L2	blue	Csa1G420360	-1.607	6.30E-03	Biotic stress	Cystein-rich secretory proteins, Antigen 5, and Pathogenesis-related 1 protein	
T2-L2	blue	Csa6G507370	-1.596	8.96E-03	Development	Senescence 1 (SEN1)	5.00E-138
T2-L2	blue	Csa1G073840	-1.59	2.16E-03	Glycolysis	Phosphoglycerate/bisphosphoglycerate mutase family protein	5.00E-91
T2-L2	blue	Csa5G615830	-1.576	1.20E-05	Transport	Transmembrane amino acid transporter family protein	1.00E-102
T2-L2	blue	Csa6G487570	-1.558	9.20E-04	Transport	General control non-repressible 5 (GCN5)	7.00E-122
T2-L2	blue	Csa2G145880	-1.522	4.76E-03	Photosynthesis	Serine hydroxymethyltransferase 4 (SHM4)	3.00E-137
T2-L2	blue	Csa3G075990	-1.49	8.18E-03	Miscellaneous	GDSL-like Lipase/Acylhydrolase superfamily protein	7.00E-17
T2-L2	blue	Csa2G361590	-1.274	3.12E-03	Not assigned	unknown	
T2-L2	blue	Csa2G036680	-1.209	1.49E-03	Polyamine metabolism	S-adenosylmethionine decarboxylase proenzyme	4.00E-165
T2-L2	blue	Csa3G134910	1.136	6.18E-03	Not assigned	unknown	

Supplementary Table 1 (Continued)

T2-L2	blue	Csa1G574970	1.827	1.18E-05	Major CHO metabolism	Hexokinase 2 (HXK2)	7.00E-51
T2-L2	blue	Csa4G618520	1.837	0.0001765	Biodegradation of xenobiotic	UDP glycosyltransferase (UGT)	1.00E-132
T2-L2	blue	Csa2G003610	1.841	0.0003098	Lipid metabolism	Abnormal inflorescence meristem (AIM1)	3.00E-294
T2-L2	blue	Csa3G730930	1.847	0.00004243	Transport	Ammonium transporter 2 (AMT2)	8E-220
T2-L2	blue	Csa6G493850	1.86	1.50E-05	Not assigned	unknown	
T2-L2	blue	Csa3G159420	1.871	1.61E-03	Not assigned	unknown	
T2-L2	blue	Csa1G528610	1.874	0.00435	Lipid metabolism	Thylakoid membrane anion transporter 1 (ANTR1)	7.00E-236
T2-L2	blue	Csa1G181360	1.875	0.001947	Transport	non-intrinsic ABC protein 9 (NAP9)	3.00E-156
T2-L2	blue	Csa3G154410	2.05	1.63E-10	Not assigned	unknown	
T2-L2	blue	Csa3G183950	2.092	0.0004285	Abiotic stress	Heat shock protein 83	0.00E+00
T2-L2	blue	Csa2G296090	2.143	2.44E-04	Not assigned	unknown	
T2-L2	blue	Csa7G291720	2.169	0.006566	Lipid metabolism	Choline kinase	4.00E-133
T2-L2	blue	Csa2G427310	2.238	0.007581	Transcription factor	MYB domain protein 73 (MYB73)	1.00E-88
T2-L2	blue	Csa3G881750	2.24	0.009561	Lipid metabolism	PR5-like receptor kinase (PR5K)	4.00E-154
T2-L2	blue	Csa5G589260	2.243	0	Amino acid metabolism	O-acetylserine(thiol) lyase (OAS-TL)	4.00E-162
T2-L2	blue	Csa3G733350	2.317	0.00004435	Cell organization	RHO guanyl-nucleotide exchange factor 11 (ROPGEF11)	9.00E-41
T2-L2	blue	Csa2G004690	2.522	0.000004593	Transcription factor	Heat shock transcription factor C1 (HSFC1)	5.00E-67
T2-L2	blue	Csa7G387690	2.631	0.000001654	Transport	Sulfate transporter 3;1 (SULTR 3;1)	3.00E-307
T2-L2	blue	Csa1G008520	2.771	0.00804	Transport	Plasma membrane proton ATPase 4 (HA4)	0.00E+00
T2-L2	blue	Csa6G514850	3.048	0.00009803	Miscellaneous	Cytochrome P450 family 77 subfamily B polypeptide 1 (CYP77B1)	1.00E-241
T2-L2	blue	Csa5G604150	3.14	0.01	Redox	Thioredoxin 2 (TRX2)	
T2-L2	blue	Csa5G223070	3.216	0.003151	Transcription factor	WRKY DNA-binding protein 54 (WRKY54)	3.00E-96
T2-L2	blue	Csa2G359890	3.289	3.70E-03	Not assigned	unknown	
T2-L2	blue	Csa1G596520	3.4	0.0002165	Protein degradation	Cystein proteinases superfamily protein	2.00E-168
T2-L2	blue	Csa7G395800	3.415	0.001823	Miscellaneous	Glutathione S-transferase phi 8 (GSTF8)	3.00E-82
T2-L2	blue	Csa3G119700	3.575	0.00005376	Transcription factor	WRKY DNA-binding protein 53 (WRKY53)	1.00E-102
T2-L2	blue	Csa6G381850	3.831	0.001156	Transport	Amino acid permease 3 (AAP3)	2.00E-234
T2-L2	blue	Csa1G033160	4.495	0.006049	Miscellaneous	Glutathione S-transferase tau 9 (GSTU9)	3.00E-68
T2-L2	blue	Csa6G486960	4.496	0.000002502	Transcription factor	WRKY DNA-binding protein 51 (WRKY51)	1.00E-36
T2-L2	blue	Csa2G058620	5.345	0.003945	Abiotic stress	DNAJ heat shock N-terminal domain-containing protein	2.00E-137

Supplementary Table 1 (Continued)

T2-L2	blue	Csa1G574970	1.827	1.18E-05	Major CHO metabolism	Hexokinase 2 (HXK2)	7.00E-51
T2-L2	blue	Csa4G618520	1.837	0.0001765	Biodegradation of xenobiotic	UDP glycosyltransferase (UGT)	1.00E-132
T2-L2	blue	Csa2G003610	1.841	0.0003098	Lipid metabolism	Abnormal inflorescence meristem (AIM1)	3.00E-294
T2-L2	blue	Csa3G730930	1.847	0.00004243	Transport	Ammonium transporter 2 (AMT2)	8E-220
T2-L2	blue	Csa6G493850	1.86	1.50E-05	Not assigned	unknown	
T2-L2	blue	Csa3G159420	1.871	1.61E-03	Not assigned	unknown	
T2-L2	blue	Csa1G528610	1.874	0.00435	Lipid metabolism	Thylakoid membrane anion transporter 1 (ANTR1)	7.00E-236
T2-L2	blue	Csa1G181360	1.875	0.001947	Transport	non-intrinsic ABC protein 9 (NAP9)	3.00E-156
T2-L2	blue	Csa3G154410	2.05	1.63E-10	Not assigned	unknown	
T2-L2	blue	Csa3G183950	2.092	0.0004285	Abiotic stress	Heat shock protein 83	0.00E+00
T2-L2	blue	Csa2G296090	2.143	2.44E-04	Not assigned	unknown	
T2-L2	blue	Csa7G291720	2.169	0.006566	Lipid metabolism	Choline kinase	4.00E-133
T2-L2	blue	Csa2G427310	2.238	0.007581	Transcription factor	MYB domain protein 73 (MYB73)	1.00E-88
T2-L2	blue	Csa3G881750	2.24	0.009561	Lipid metabolism	PR5-like receptor kinase (PR5K)	4.00E-154
T2-L2	blue	Csa5G589260	2.243	0	Amino acid metabolism	O-acetylserine(thiol) lyase (OAS-TL)	4.00E-162
T2-L2	blue	Csa3G733350	2.317	0.00004435	Cell organization	RHO guanyl-nucleotide exchange factor 11 (ROPGEF11)	9.00E-41
T2-L2	blue	Csa2G004690	2.522	0.000004593	Transcription factor	Heat shock transcription factor C1 (HSFC1)	5.00E-67
T2-L2	blue	Csa7G387690	2.631	0.000001654	Transport	Sulfate transporter 3;1 (SULTR 3;1)	3.00E-307
T2-L2	blue	Csa1G008520	2.771	0.00804	Transport	Plasma membrane proton ATPase 4 (HA4)	0.00E+00
T2-L2	blue	Csa6G514850	3.048	0.00009803	Miscellaneous	Cytochrome P450 family 77 subfamily B polypeptide 1 (CYP77B1)	1.00E-241
T2-L2	blue	Csa5G604150	3.14	0.01	Redox	Thioredoxin 2 (TRX2)	
T2-L2	blue	Csa5G223070	3.216	0.003151	Transcription factor	WRKY DNA-binding protein 54 (WRKY54)	3.00E-96
T2-L2	blue	Csa2G359890	3.289	3.70E-03	Not assigned	unknown	
T2-L2	blue	Csa1G596520	3.4	0.0002165	Protein degradation	Cystein proteinases superfamily protein	2.00E-168
T2-L2	blue	Csa7G395800	3.415	0.001823	Miscellaneous	Glutathione S-transferase phi 8 (GSTF8)	3.00E-82
T2-L2	blue	Csa3G119700	3.575	0.00005376	Transcription factor	WRKY DNA-binding protein 53 (WRKY53)	1.00E-102
T2-L2	blue	Csa6G381850	3.831	0.001156	Transport	Amino acid permease 3 (AAP3)	2.00E-234
T2-L2	blue	Csa1G033160	4.495	0.006049	Miscellaneous	Glutathione S-transferase tau 9 (GSTU9)	3.00E-68
T2-L2	blue	Csa6G486960	4.496	0.000002502	Transcription factor	WRKY DNA-binding protein 51 (WRKY51)	1.00E-36
T2-L2	blue	Csa2G058620	5.345	0.003945	Abiotic stress	DNAJ heat shock N-terminal domain-containing protein	2.00E-137

Supplementary Table 1 (Continued)

T2-L2	blue	Csa1G574970	1.827	1.18E-05	Major CHO metabolism	Hexokinase 2 (HXK2)	7.00E-51
T2-L2	blue	Csa4G618520	1.837	0.0001765	Biodegradation of xenobiotic	UDP glycosyltransferase (UGT)	1.00E-132
T2-L2	blue	Csa2G003610	1.841	0.0003098	Lipid metabolism	Abnormal inflorescence meristem (AIM1)	3.00E-294
T2-L2	blue	Csa3G730930	1.847	0.00004243	Transport	Ammonium transporter 2 (AMT2)	8E-220
T2-L2	blue	Csa6G493850	1.86	1.50E-05	Not assigned	unknown	
T2-L2	blue	Csa3G159420	1.871	1.61E-03	Not assigned	unknown	
T2-L2	blue	Csa1G528610	1.874	0.00435	Lipid metabolism	Thylakoid membrane anion transporter 1 (ANTR1)	7.00E-236
T2-L2	blue	Csa1G181360	1.875	0.001947	Transport	non-intrinsic ABC protein 9 (NAP9)	3.00E-156
T2-L2	blue	Csa3G154410	2.05	1.63E-10	Not assigned	unknown	
T2-L2	blue	Csa3G183950	2.092	0.0004285	Abiotic stress	Heat shock protein 83	0.00E+00
T2-L2	blue	Csa2G296090	2.143	2.44E-04	Not assigned	unknown	
T2-L2	blue	Csa7G291720	2.169	0.006566	Lipid metabolism	Choline kinase	4.00E-133
T2-L2	blue	Csa2G427310	2.238	0.007581	Transcription factor	MYB domain protein 73 (MYB73)	1.00E-88
T2-L2	blue	Csa3G881750	2.24	0.009561	Lipid metabolism	PR5-like receptor kinase (PR5K)	4.00E-154
T2-L2	blue	Csa5G589260	2.243	0	Amino acid metabolism	O-acetylserine(thiol) lyase (OAS-TL)	4.00E-162
T2-L2	blue	Csa3G733350	2.317	0.00004435	Cell organization	RHO guanyl-nucleotide exchange factor 11 (ROPGEF11)	9.00E-41
T2-L2	blue	Csa2G004690	2.522	0.000004593	Transcription factor	Heat shock transcription factor C1 (HSFC1)	5.00E-67
T2-L2	blue	Csa7G387690	2.631	0.000001654	Transport	Sulfate transporter 3;1 (SULTR 3;1)	3.00E-307
T2-L2	blue	Csa1G008520	2.771	0.00804	Transport	Plasma membrane proton ATPase 4 (HA4)	0.00E+00
T2-L2	blue	Csa6G514850	3.048	0.00009803	Miscellaneous	Cytochrome P450 family 77 subfamily B polypeptide 1 (CYP77B1)	1.00E-241
T2-L2	blue	Csa5G604150	3.14	0.01	Redox	Thioredoxin 2 (TRX2)	
T2-L2	blue	Csa5G223070	3.216	0.003151	Transcription factor	WRKY DNA-binding protein 54 (WRKY54)	3.00E-96
T2-L2	blue	Csa2G359890	3.289	3.70E-03	Not assigned	unknown	
T2-L2	blue	Csa1G596520	3.4	0.0002165	Protein degradation	Cystein proteinases superfamily protein	2.00E-168
T2-L2	blue	Csa7G395800	3.415	0.001823	Miscellaneous	Glutathione S-transferase phi 8 (GSTF8)	3.00E-82
T2-L2	blue	Csa3G119700	3.575	0.00005376	Transcription factor	WRKY DNA-binding protein 53 (WRKY53)	1.00E-102
T2-L2	blue	Csa6G381850	3.831	0.001156	Transport	Amino acid permease 3 (AAP3)	2.00E-234
T2-L2	blue	Csa1G033160	4.495	0.006049	Miscellaneous	Glutathione S-transferase tau 9 (GSTU9)	3.00E-68
T2-L2	blue	Csa6G486960	4.496	0.000002502	Transcription factor	WRKY DNA-binding protein 51 (WRKY51)	1.00E-36
T2-L2	blue	Csa2G058620	5.345	0.003945	Abiotic stress	DNAJ heat shock N-terminal domain-containing protein	2.00E-137

Supplementary Table 1 (Continued)

T2-L2	blue	Csa1G574970	1.827	1.18E-05	Major CHO metabolism	Hexokinase 2 (HXK2)	7.00E-51
T2-L2	blue	Csa4G618520	1.837	0.0001765	Biodegradation of xenobiotic	UDP glycosyltransferase (UGT)	1.00E-132
T2-L2	blue	Csa2G003610	1.841	0.0003098	Lipid metabolism	Abnormal inflorescence meristem (AIM1)	3.00E-294
T2-L2	blue	Csa3G730930	1.847	0.00004243	Transport	Ammonium transporter 2 (AMT2)	8E-220
T2-L2	blue	Csa6G493850	1.86	1.50E-05	Not assigned	unknown	
T2-L2	blue	Csa3G159420	1.871	1.61E-03	Not assigned	unknown	
T2-L2	blue	Csa1G528610	1.874	0.00435	Lipid metabolism	Thylakoid membrane anion transporter 1 (ANTR1)	7.00E-236
T2-L2	blue	Csa1G181360	1.875	0.001947	Transport	non-intrinsic ABC protein 9 (NAP9)	3.00E-156
T2-L2	blue	Csa3G154410	2.05	1.63E-10	Not assigned	unknown	
T2-L2	blue	Csa3G183950	2.092	0.0004285	Abiotic stress	Heat shock protein 83	0.00E+00
T2-L2	blue	Csa2G296090	2.143	2.44E-04	Not assigned	unknown	
T2-L2	blue	Csa7G291720	2.169	0.006566	Lipid metabolism	Choline kinase	4.00E-133
T2-L2	blue	Csa2G427310	2.238	0.007581	Transcription factor	MYB domain protein 73 (MYB73)	1.00E-88
T2-L2	blue	Csa3G881750	2.24	0.009561	Lipid metabolism	PR5-like receptor kinase (PR5K)	4.00E-154
T2-L2	blue	Csa5G589260	2.243	0	Amino acid metabolism	O-acetylserine(thiol) lyase (OAS-TL)	4.00E-162
T2-L2	blue	Csa3G733350	2.317	0.00004435	Cell organization	RHO guanyl-nucleotide exchange factor 11 (ROPGEF11)	9.00E-41
T2-L2	blue	Csa2G004690	2.522	0.000004593	Transcription factor	Heat shock transcription factor C1 (HSFC1)	5.00E-67
T2-L2	blue	Csa7G387690	2.631	0.000001654	Transport	Sulfate transporter 3;1 (SULTR 3;1)	3.00E-307
T2-L2	blue	Csa1G008520	2.771	0.00804	Transport	Plasma membrane proton ATPase 4 (HA4)	0.00E+00
T2-L2	blue	Csa6G514850	3.048	0.00009803	Miscellaneous	Cytochrome P450 family 77 subfamily B polypeptide 1 (CYP77B1)	1.00E-241
T2-L2	blue	Csa5G604150	3.14	0.01	Redox	Thioredoxin 2 (TRX2)	
T2-L2	blue	Csa5G223070	3.216	0.003151	Transcription factor	WRKY DNA-binding protein 54 (WRKY54)	3.00E-96
T2-L2	blue	Csa2G359890	3.289	3.70E-03	Not assigned	unknown	
T2-L2	blue	Csa1G596520	3.4	0.0002165	Protein degradation	Cystein proteinases superfamily protein	2.00E-168
T2-L2	blue	Csa7G395800	3.415	0.001823	Miscellaneous	Glutathione S-transferase phi 8 (GSTF8)	3.00E-82
T2-L2	blue	Csa3G119700	3.575	0.00005376	Transcription factor	WRKY DNA-binding protein 53 (WRKY53)	1.00E-102
T2-L2	blue	Csa6G381850	3.831	0.001156	Transport	Amino acid permease 3 (AAP3)	2.00E-234
T2-L2	blue	Csa1G033160	4.495	0.006049	Miscellaneous	Glutathione S-transferase tau 9 (GSTU9)	3.00E-68
T2-L2	blue	Csa6G486960	4.496	0.000002502	Transcription factor	WRKY DNA-binding protein 51 (WRKY51)	1.00E-36
T2-L2	blue	Csa2G058620	5.345	0.003945	Abiotic stress	DNAJ heat shock N-terminal domain-containing protein	2.00E-137

Supplementary Table 1 (Continued)

T2-L2	blue	Csa1G574970	1.827	1.18E-05	Major CHO metabolism	Hexokinase 2 (HXK2)	7.00E-51
T2-L2	blue	Csa4G618520	1.837	0.0001765	Biodegradation of xenobiotic	UDP glycosyltransferase (UGT)	1.00E-132
T2-L2	blue	Csa2G003610	1.841	0.0003098	Lipid metabolism	Abnormal inflorescence meristem (AIM1)	3.00E-294
T2-L2	blue	Csa3G730930	1.847	0.00004243	Transport	Ammonium transporter 2 (AMT2)	8E-220
T2-L2	blue	Csa6G493850	1.86	1.50E-05	Not assigned	unknown	
T2-L2	blue	Csa3G159420	1.871	1.61E-03	Not assigned	unknown	
T2-L2	blue	Csa1G528610	1.874	0.00435	Lipid metabolism	Thylakoid membrane anion transporter 1 (ANTR1)	7.00E-236
T2-L2	blue	Csa1G181360	1.875	0.001947	Transport	non-intrinsic ABC protein 9 (NAP9)	3.00E-156
T2-L2	blue	Csa3G154410	2.05	1.63E-10	Not assigned	unknown	
T2-L2	blue	Csa3G183950	2.092	0.0004285	Abiotic stress	Heat shock protein 83	0.00E+00
T2-L2	blue	Csa2G296090	2.143	2.44E-04	Not assigned	unknown	
T2-L2	blue	Csa7G291720	2.169	0.006566	Lipid metabolism	Choline kinase	4.00E-133
T2-L2	blue	Csa2G427310	2.238	0.007581	Transcription factor	MYB domain protein 73 (MYB73)	1.00E-88
T2-L2	blue	Csa3G881750	2.24	0.009561	Lipid metabolism	PR5-like receptor kinase (PR5K)	4.00E-154
T2-L2	blue	Csa5G589260	2.243	0	Amino acid metabolism	O-acetylserine(thiol) lyase (OAS-TL)	4.00E-162
T2-L2	blue	Csa3G733350	2.317	0.00004435	Cell organization	RHO guanyl-nucleotide exchange factor 11 (ROPGEF11)	9.00E-41
T2-L2	blue	Csa2G004690	2.522	0.000004593	Transcription factor	Heat shock transcription factor C1 (HSFC1)	5.00E-67
T2-L2	blue	Csa7G387690	2.631	0.000001654	Transport	Sulfate transporter 3;1 (SULTR 3;1)	3.00E-307
T2-L2	blue	Csa1G008520	2.771	0.00804	Transport	Plasma membrane proton ATPase 4 (HA4)	0.00E+00
T2-L2	blue	Csa6G514850	3.048	0.00009803	Miscellaneous	Cytochrome P450 family 77 subfamily B polypeptide 1 (CYP77B1)	1.00E-241
T2-L2	blue	Csa5G604150	3.14	0.01	Redox	Thioredoxin 2 (TRX2)	
T2-L2	blue	Csa5G223070	3.216	0.003151	Transcription factor	WRKY DNA-binding protein 54 (WRKY54)	3.00E-96
T2-L2	blue	Csa2G359890	3.289	3.70E-03	Not assigned	unknown	
T2-L2	blue	Csa1G596520	3.4	0.0002165	Protein degradation	Cystein proteinases superfamily protein	2.00E-168
T2-L2	blue	Csa7G395800	3.415	0.001823	Miscellaneous	Glutathione S-transferase phi 8 (GSTF8)	3.00E-82
T2-L2	blue	Csa3G119700	3.575	0.00005376	Transcription factor	WRKY DNA-binding protein 53 (WRKY53)	1.00E-102
T2-L2	blue	Csa6G381850	3.831	0.001156	Transport	Amino acid permease 3 (AAP3)	2.00E-234
T2-L2	blue	Csa1G033160	4.495	0.006049	Miscellaneous	Glutathione S-transferase tau 9 (GSTU9)	3.00E-68
T2-L2	blue	Csa6G486960	4.496	0.000002502	Transcription factor	WRKY DNA-binding protein 51 (WRKY51)	1.00E-36
T2-L2	blue	Csa2G058620	5.345	0.003945	Abiotic stress	DNAJ heat shock N-terminal domain-containing protein	2.00E-137

Supplementary Table 1 (Continued)

T2-L2	blue	Csa1G574970	1.827	1.18E-05	Major CHO metabolism	Hexokinase 2 (HXK2)	7.00E-51
T2-L2	blue	Csa4G618520	1.837	0.0001765	Biodegradation of xenobiotic	UDP glycosyltransferase (UGT)	1.00E-132
T2-L2	blue	Csa2G003610	1.841	0.0003098	Lipid metabolism	Abnormal inflorescence meristem (AIM1)	3.00E-294
T2-L2	blue	Csa3G730930	1.847	0.00004243	Transport	Ammonium transporter 2 (AMT2)	8E-220
T2-L2	blue	Csa6G493850	1.86	1.50E-05	Not assigned	unknown	
T2-L2	blue	Csa3G159420	1.871	1.61E-03	Not assigned	unknown	
T2-L2	blue	Csa1G528610	1.874	0.00435	Lipid metabolism	Thylakoid membrane anion transporter 1 (ANTR1)	7.00E-236
T2-L2	blue	Csa1G181360	1.875	0.001947	Transport	non-intrinsic ABC protein 9 (NAP9)	3.00E-156
T2-L2	blue	Csa3G154410	2.05	1.63E-10	Not assigned	unknown	
T2-L2	blue	Csa3G183950	2.092	0.0004285	Abiotic stress	Heat shock protein 83	0.00E+00
T2-L2	blue	Csa2G296090	2.143	2.44E-04	Not assigned	unknown	
T2-L2	blue	Csa7G291720	2.169	0.006566	Lipid metabolism	Choline kinase	4.00E-133
T2-L2	blue	Csa2G427310	2.238	0.007581	Transcription factor	MYB domain protein 73 (MYB73)	1.00E-88
T2-L2	blue	Csa3G881750	2.24	0.009561	Lipid metabolism	PR5-like receptor kinase (PR5K)	4.00E-154
T2-L2	blue	Csa5G589260	2.243	0	Amino acid metabolism	O-acetylserine(thiol) lyase (OAS-TL)	4.00E-162
T2-L2	blue	Csa3G733350	2.317	0.00004435	Cell organization	RHO guanyl-nucleotide exchange factor 11 (ROPGEF11)	9.00E-41
T2-L2	blue	Csa2G004690	2.522	0.000004593	Transcription factor	Heat shock transcription factor C1 (HSFC1)	5.00E-67
T2-L2	blue	Csa7G387690	2.631	0.000001654	Transport	Sulfate transporter 3;1 (SULTR 3;1)	3.00E-307
T2-L2	blue	Csa1G008520	2.771	0.00804	Transport	Plasma membrane proton ATPase 4 (HA4)	0.00E+00
T2-L2	blue	Csa6G514850	3.048	0.00009803	Miscellaneous	Cytochrome P450 family 77 subfamily B polypeptide 1 (CYP77B1)	1.00E-241
T2-L2	blue	Csa5G604150	3.14	0.01	Redox	Thioredoxin 2 (TRX2)	
T2-L2	blue	Csa5G223070	3.216	0.003151	Transcription factor	WRKY DNA-binding protein 54 (WRKY54)	3.00E-96
T2-L2	blue	Csa2G359890	3.289	3.70E-03	Not assigned	unknown	
T2-L2	blue	Csa1G596520	3.4	0.0002165	Protein degradation	Cystein proteinases superfamily protein	2.00E-168
T2-L2	blue	Csa7G395800	3.415	0.001823	Miscellaneous	Glutathione S-transferase phi 8 (GSTF8)	3.00E-82
T2-L2	blue	Csa3G119700	3.575	0.00005376	Transcription factor	WRKY DNA-binding protein 53 (WRKY53)	1.00E-102
T2-L2	blue	Csa6G381850	3.831	0.001156	Transport	Amino acid permease 3 (AAP3)	2.00E-234
T2-L2	blue	Csa1G033160	4.495	0.006049	Miscellaneous	Glutathione S-transferase tau 9 (GSTU9)	3.00E-68
T2-L2	blue	Csa6G486960	4.496	0.000002502	Transcription factor	WRKY DNA-binding protein 51 (WRKY51)	1.00E-36
T2-L2	blue	Csa2G058620	5.345	0.003945	Abiotic stress	DNAJ heat shock N-terminal domain-containing protein	2.00E-137

Supplementary Table 1 (Continued)

T2-L2	blue	Csa1G574970	1.827	1.18E-05	Major CHO metabolism	Hexokinase 2 (HXK2)	7.00E-51
T2-L2	blue	Csa4G618520	1.837	0.0001765	Biodegradation of xenobiotic	UDP glycosyltransferase (UGT)	1.00E-132
T2-L2	blue	Csa2G003610	1.841	0.0003098	Lipid metabolism	Abnormal inflorescence meristem (AIM1)	3.00E-294
T2-L2	blue	Csa3G730930	1.847	0.00004243	Transport	Ammonium transporter 2 (AMT2)	8E-220
T2-L2	blue	Csa6G493850	1.86	1.50E-05	Not assigned	unknown	
T2-L2	blue	Csa3G159420	1.871	1.61E-03	Not assigned	unknown	
T2-L2	blue	Csa1G528610	1.874	0.00435	Lipid metabolism	Thylakoid membrane anion transporter 1 (ANTR1)	7.00E-236
T2-L2	blue	Csa1G181360	1.875	0.001947	Transport	non-intrinsic ABC protein 9 (NAP9)	3.00E-156
T2-L2	blue	Csa3G154410	2.05	1.63E-10	Not assigned	unknown	
T2-L2	blue	Csa3G183950	2.092	0.0004285	Abiotic stress	Heat shock protein 83	0.00E+00
T2-L2	blue	Csa2G296090	2.143	2.44E-04	Not assigned	unknown	
T2-L2	blue	Csa7G291720	2.169	0.006566	Lipid metabolism	Choline kinase	4.00E-133
T2-L2	blue	Csa2G427310	2.238	0.007581	Transcription factor	MYB domain protein 73 (MYB73)	1.00E-88
T2-L2	blue	Csa3G881750	2.24	0.009561	Lipid metabolism	PR5-like receptor kinase (PR5K)	4.00E-154
T2-L2	blue	Csa5G589260	2.243	0	Amino acid metabolism	O-acetylserine(thiol) lyase (OAS-TL)	4.00E-162
T2-L2	blue	Csa3G733350	2.317	0.00004435	Cell organization	RHO guanyl-nucleotide exchange factor 11 (ROPGEF11)	9.00E-41
T2-L2	blue	Csa2G004690	2.522	0.000004593	Transcription factor	Heat shock transcription factor C1 (HSFC1)	5.00E-67
T2-L2	blue	Csa7G387690	2.631	0.000001654	Transport	Sulfate transporter 3;1 (SULTR 3;1)	3.00E-307
T2-L2	blue	Csa1G008520	2.771	0.00804	Transport	Plasma membrane proton ATPase 4 (HA4)	0.00E+00
T2-L2	blue	Csa6G514850	3.048	0.00009803	Miscellaneous	Cytochrome P450 family 77 subfamily B polypeptide 1 (CYP77B1)	1.00E-241
T2-L2	blue	Csa5G604150	3.14	0.01	Redox	Thioredoxin 2 (TRX2)	
T2-L2	blue	Csa5G223070	3.216	0.003151	Transcription factor	WRKY DNA-binding protein 54 (WRKY54)	3.00E-96
T2-L2	blue	Csa2G359890	3.289	3.70E-03	Not assigned	unknown	
T2-L2	blue	Csa1G596520	3.4	0.0002165	Protein degradation	Cystein proteinases superfamily protein	2.00E-168
T2-L2	blue	Csa7G395800	3.415	0.001823	Miscellaneous	Glutathione S-transferase phi 8 (GSTF8)	3.00E-82
T2-L2	blue	Csa3G119700	3.575	0.00005376	Transcription factor	WRKY DNA-binding protein 53 (WRKY53)	1.00E-102
T2-L2	blue	Csa6G381850	3.831	0.001156	Transport	Amino acid permease 3 (AAP3)	2.00E-234
T2-L2	blue	Csa1G033160	4.495	0.006049	Miscellaneous	Glutathione S-transferase tau 9 (GSTU9)	3.00E-68
T2-L2	blue	Csa6G486960	4.496	0.000002502	Transcription factor	WRKY DNA-binding protein 51 (WRKY51)	1.00E-36
T2-L2	blue	Csa2G058620	5.345	0.003945	Abiotic stress	DNAJ heat shock N-terminal domain-containing protein	2.00E-137

Supplementary Table 1 (Continued)

T2-L2	blue	Csa1G574970	1.827	1.18E-05	Major CHO metabolism	Hexokinase 2 (HXK2)	7.00E-51
T2-L2	blue	Csa4G618520	1.837	0.0001765	Biodegradation of xenobiotic	UDP glycosyltransferase (UGT)	1.00E-132
T2-L2	blue	Csa2G003610	1.841	0.0003098	Lipid metabolism	Abnormal inflorescence meristem (AIM1)	3.00E-294
T2-L2	blue	Csa3G730930	1.847	0.00004243	Transport	Ammonium transporter 2 (AMT2)	8E-220
T2-L2	blue	Csa6G493850	1.86	1.50E-05	Not assigned	unknown	
T2-L2	blue	Csa3G159420	1.871	1.61E-03	Not assigned	unknown	
T2-L2	blue	Csa1G528610	1.874	0.00435	Lipid metabolism	Thylakoid membrane anion transporter 1 (ANTR1)	7.00E-236
T2-L2	blue	Csa1G181360	1.875	0.001947	Transport	non-intrinsic ABC protein 9 (NAP9)	3.00E-156
T2-L2	blue	Csa3G154410	2.05	1.63E-10	Not assigned	unknown	
T2-L2	blue	Csa3G183950	2.092	0.0004285	Abiotic stress	Heat shock protein 83	0.00E+00
T2-L2	blue	Csa2G296090	2.143	2.44E-04	Not assigned	unknown	
T2-L2	blue	Csa7G291720	2.169	0.006566	Lipid metabolism	Choline kinase	4.00E-133
T2-L2	blue	Csa2G427310	2.238	0.007581	Transcription factor	MYB domain protein 73 (MYB73)	1.00E-88
T2-L2	blue	Csa3G881750	2.24	0.009561	Lipid metabolism	PR5-like receptor kinase (PR5K)	4.00E-154
T2-L2	blue	Csa5G589260	2.243	0	Amino acid metabolism	O-acetylserine(thiol) lyase (OAS-TL)	4.00E-162
T2-L2	blue	Csa3G733350	2.317	0.00004435	Cell organization	RHO guanyl-nucleotide exchange factor 11 (ROPGEF11)	9.00E-41
T2-L2	blue	Csa2G004690	2.522	0.000004593	Transcription factor	Heat shock transcription factor C1 (HSFC1)	5.00E-67
T2-L2	blue	Csa7G387690	2.631	0.000001654	Transport	Sulfate transporter 3;1 (SULTR 3;1)	3.00E-307
T2-L2	blue	Csa1G008520	2.771	0.00804	Transport	Plasma membrane proton ATPase 4 (HA4)	0.00E+00
T2-L2	blue	Csa6G514850	3.048	0.00009803	Miscellaneous	Cytochrome P450 family 77 subfamily B polypeptide 1 (CYP77B1)	1.00E-241
T2-L2	blue	Csa5G604150	3.14	0.01	Redox	Thioredoxin 2 (TRX2)	
T2-L2	blue	Csa5G223070	3.216	0.003151	Transcription factor	WRKY DNA-binding protein 54 (WRKY54)	3.00E-96
T2-L2	blue	Csa2G359890	3.289	3.70E-03	Not assigned	unknown	
T2-L2	blue	Csa1G596520	3.4	0.0002165	Protein degradation	Cystein proteinases superfamily protein	2.00E-168
T2-L2	blue	Csa7G395800	3.415	0.001823	Miscellaneous	Glutathione S-transferase phi 8 (GSTF8)	3.00E-82
T2-L2	blue	Csa3G119700	3.575	0.00005376	Transcription factor	WRKY DNA-binding protein 53 (WRKY53)	1.00E-102
T2-L2	blue	Csa6G381850	3.831	0.001156	Transport	Amino acid permease 3 (AAP3)	2.00E-234
T2-L2	blue	Csa1G033160	4.495	0.006049	Miscellaneous	Glutathione S-transferase tau 9 (GSTU9)	3.00E-68
T2-L2	blue	Csa6G486960	4.496	0.000002502	Transcription factor	WRKY DNA-binding protein 51 (WRKY51)	1.00E-36
T2-L2	blue	Csa2G058620	5.345	0.003945	Abiotic stress	DNAJ heat shock N-terminal domain-containing protein	2.00E-137

Supplementary Table 1 (Continued)

T2-L2	blue	Csa1G574970	1.827	1.18E-05	Major CHO metabolism	Hexokinase 2 (HXK2)	7.00E-51
T2-L2	blue	Csa4G618520	1.837	0.0001765	Biodegradation of xenobiotic	UDP glycosyltransferase (UGT)	1.00E-132
T2-L2	blue	Csa2G003610	1.841	0.0003098	Lipid metabolism	Abnormal inflorescence meristem (AIM1)	3.00E-294
T2-L2	blue	Csa3G730930	1.847	0.00004243	Transport	Ammonium transporter 2 (AMT2)	8E-220
T2-L2	blue	Csa6G493850	1.86	1.50E-05	Not assigned	unknown	
T2-L2	blue	Csa3G159420	1.871	1.61E-03	Not assigned	unknown	
T2-L2	blue	Csa1G528610	1.874	0.00435	Lipid metabolism	Thylakoid membrane anion transporter 1 (ANTR1)	7.00E-236
T2-L2	blue	Csa1G181360	1.875	0.001947	Transport	non-intrinsic ABC protein 9 (NAP9)	3.00E-156
T2-L2	blue	Csa3G154410	2.05	1.63E-10	Not assigned	unknown	
T2-L2	blue	Csa3G183950	2.092	0.0004285	Abiotic stress	Heat shock protein 83	0.00E+00
T2-L2	blue	Csa2G296090	2.143	2.44E-04	Not assigned	unknown	
T2-L2	blue	Csa7G291720	2.169	0.006566	Lipid metabolism	Choline kinase	4.00E-133
T2-L2	blue	Csa2G427310	2.238	0.007581	Transcription factor	MYB domain protein 73 (MYB73)	1.00E-88
T2-L2	blue	Csa3G881750	2.24	0.009561	Lipid metabolism	PR5-like receptor kinase (PR5K)	4.00E-154
T2-L2	blue	Csa5G589260	2.243	0	Amino acid metabolism	O-acetylserine(thiol) lyase (OAS-TL)	4.00E-162
T2-L2	blue	Csa3G733350	2.317	0.00004435	Cell organization	RHO guanyl-nucleotide exchange factor 11 (ROPGEF11)	9.00E-41
T2-L2	blue	Csa2G004690	2.522	0.000004593	Transcription factor	Heat shock transcription factor C1 (HSFC1)	5.00E-67
T2-L2	blue	Csa7G387690	2.631	0.000001654	Transport	Sulfate transporter 3;1 (SULTR 3;1)	3.00E-307
T2-L2	blue	Csa1G008520	2.771	0.00804	Transport	Plasma membrane proton ATPase 4 (HA4)	0.00E+00
T2-L2	blue	Csa6G514850	3.048	0.00009803	Miscellaneous	Cytochrome P450 family 77 subfamily B polypeptide 1 (CYP77B1)	1.00E-241
T2-L2	blue	Csa5G604150	3.14	0.01	Redox	Thioredoxin 2 (TRX2)	
T2-L2	blue	Csa5G223070	3.216	0.003151	Transcription factor	WRKY DNA-binding protein 54 (WRKY54)	3.00E-96
T2-L2	blue	Csa2G359890	3.289	3.70E-03	Not assigned	unknown	
T2-L2	blue	Csa1G596520	3.4	0.0002165	Protein degradation	Cystein proteinases superfamily protein	2.00E-168
T2-L2	blue	Csa7G395800	3.415	0.001823	Miscellaneous	Glutathione S-transferase phi 8 (GSTF8)	3.00E-82
T2-L2	blue	Csa3G119700	3.575	0.00005376	Transcription factor	WRKY DNA-binding protein 53 (WRKY53)	1.00E-102
T2-L2	blue	Csa6G381850	3.831	0.001156	Transport	Amino acid permease 3 (AAP3)	2.00E-234
T2-L2	blue	Csa1G033160	4.495	0.006049	Miscellaneous	Glutathione S-transferase tau 9 (GSTU9)	3.00E-68
T2-L2	blue	Csa6G486960	4.496	0.000002502	Transcription factor	WRKY DNA-binding protein 51 (WRKY51)	1.00E-36
T2-L2	blue	Csa2G058620	5.345	0.003945	Abiotic stress	DNAJ heat shock N-terminal domain-containing protein	2.00E-137

Supplementary Table 1 (Continued)

T2-L2	blue	Csa1G574970	1.827	1.18E-05	Major CHO metabolism	Hexokinase 2 (HXK2)	7.00E-51
T2-L2	blue	Csa4G618520	1.837	0.0001765	Biodegradation of xenobiotic	UDP glycosyltransferase (UGT)	1.00E-132
T2-L2	blue	Csa2G003610	1.841	0.0003098	Lipid metabolism	Abnormal inflorescence meristem (AIM1)	3.00E-294
T2-L2	blue	Csa3G730930	1.847	0.00004243	Transport	Ammonium transporter 2 (AMT2)	8E-220
T2-L2	blue	Csa6G493850	1.86	1.50E-05	Not assigned	unknown	
T2-L2	blue	Csa3G159420	1.871	1.61E-03	Not assigned	unknown	
T2-L2	blue	Csa1G528610	1.874	0.00435	Lipid metabolism	Thylakoid membrane anion transporter 1 (ANTR1)	7.00E-236
T2-L2	blue	Csa1G181360	1.875	0.001947	Transport	non-intrinsic ABC protein 9 (NAP9)	3.00E-156
T2-L2	blue	Csa3G154410	2.05	1.63E-10	Not assigned	unknown	
T2-L2	blue	Csa3G183950	2.092	0.0004285	Abiotic stress	Heat shock protein 83	0.00E+00
T2-L2	blue	Csa2G296090	2.143	2.44E-04	Not assigned	unknown	
T2-L2	blue	Csa7G291720	2.169	0.006566	Lipid metabolism	Choline kinase	4.00E-133
T2-L2	blue	Csa2G427310	2.238	0.007581	Transcription factor	MYB domain protein 73 (MYB73)	1.00E-88
T2-L2	blue	Csa3G881750	2.24	0.009561	Lipid metabolism	PR5-like receptor kinase (PR5K)	4.00E-154
T2-L2	blue	Csa5G589260	2.243	0	Amino acid metabolism	O-acetylserine(thiol) lyase (OAS-TL)	4.00E-162
T2-L2	blue	Csa3G733350	2.317	0.00004435	Cell organization	RHO guanyl-nucleotide exchange factor 11 (ROPGEF11)	9.00E-41
T2-L2	blue	Csa2G004690	2.522	0.000004593	Transcription factor	Heat shock transcription factor C1 (HSFC1)	5.00E-67
T2-L2	blue	Csa7G387690	2.631	0.000001654	Transport	Sulfate transporter 3;1 (SULTR 3;1)	3.00E-307
T2-L2	blue	Csa1G008520	2.771	0.00804	Transport	Plasma membrane proton ATPase 4 (HA4)	0.00E+00
T2-L2	blue	Csa6G514850	3.048	0.00009803	Miscellaneous	Cytochrome P450 family 77 subfamily B polypeptide 1 (CYP77B1)	1.00E-241
T2-L2	blue	Csa5G604150	3.14	0.01	Redox	Thioredoxin 2 (TRX2)	
T2-L2	blue	Csa5G223070	3.216	0.003151	Transcription factor	WRKY DNA-binding protein 54 (WRKY54)	3.00E-96
T2-L2	blue	Csa2G359890	3.289	3.70E-03	Not assigned	unknown	
T2-L2	blue	Csa1G596520	3.4	0.0002165	Protein degradation	Cystein proteinases superfamily protein	2.00E-168
T2-L2	blue	Csa7G395800	3.415	0.001823	Miscellaneous	Glutathione S-transferase phi 8 (GSTF8)	3.00E-82
T2-L2	blue	Csa3G119700	3.575	0.00005376	Transcription factor	WRKY DNA-binding protein 53 (WRKY53)	1.00E-102
T2-L2	blue	Csa6G381850	3.831	0.001156	Transport	Amino acid permease 3 (AAP3)	2.00E-234
T2-L2	blue	Csa1G033160	4.495	0.006049	Miscellaneous	Glutathione S-transferase tau 9 (GSTU9)	3.00E-68
T2-L2	blue	Csa6G486960	4.496	0.000002502	Transcription factor	WRKY DNA-binding protein 51 (WRKY51)	1.00E-36
T2-L2	blue	Csa2G058620	5.345	0.003945	Abiotic stress	DNAJ heat shock N-terminal domain-containing protein	2.00E-137

Supplementary Table 1 (Continued)

T2-L2	blue	Csa1G574970	1.827	1.18E-05	Major CHO metabolism	Hexokinase 2 (HXK2)	7.00E-51
T2-L2	blue	Csa4G618520	1.837	0.0001765	Biodegradation of xenobiotic	UDP glycosyltransferase (UGT)	1.00E-132
T2-L2	blue	Csa2G003610	1.841	0.0003098	Lipid metabolism	Abnormal inflorescence meristem (AIM1)	3.00E-294
T2-L2	blue	Csa3G730930	1.847	0.00004243	Transport	Ammonium transporter 2 (AMT2)	8E-220
T2-L2	blue	Csa6G493850	1.86	1.50E-05	Not assigned	unknown	
T2-L2	blue	Csa3G159420	1.871	1.61E-03	Not assigned	unknown	
T2-L2	blue	Csa1G528610	1.874	0.00435	Lipid metabolism	Thylakoid membrane anion transporter 1 (ANTR1)	7.00E-236
T2-L2	blue	Csa1G181360	1.875	0.001947	Transport	non-intrinsic ABC protein 9 (NAP9)	3.00E-156
T2-L2	blue	Csa3G154410	2.05	1.63E-10	Not assigned	unknown	
T2-L2	blue	Csa3G183950	2.092	0.0004285	Abiotic stress	Heat shock protein 83	0.00E+00
T2-L2	blue	Csa2G296090	2.143	2.44E-04	Not assigned	unknown	
T2-L2	blue	Csa7G291720	2.169	0.006566	Lipid metabolism	Choline kinase	4.00E-133
T2-L2	blue	Csa2G427310	2.238	0.007581	Transcription factor	MYB domain protein 73 (MYB73)	1.00E-88
T2-L2	blue	Csa3G881750	2.24	0.009561	Lipid metabolism	PR5-like receptor kinase (PR5K)	4.00E-154
T2-L2	blue	Csa5G589260	2.243	0	Amino acid metabolism	O-acetylserine(thiol) lyase (OAS-TL)	4.00E-162
T2-L2	blue	Csa3G733350	2.317	0.00004435	Cell organization	RHO guanyl-nucleotide exchange factor 11 (ROPGEF11)	9.00E-41
T2-L2	blue	Csa2G004690	2.522	0.000004593	Transcription factor	Heat shock transcription factor C1 (HSFC1)	5.00E-67
T2-L2	blue	Csa7G387690	2.631	0.000001654	Transport	Sulfate transporter 3;1 (SULTR 3;1)	3.00E-307
T2-L2	blue	Csa1G008520	2.771	0.00804	Transport	Plasma membrane proton ATPase 4 (HA4)	0.00E+00
T2-L2	blue	Csa6G514850	3.048	0.00009803	Miscellaneous	Cytochrome P450 family 77 subfamily B polypeptide 1 (CYP77B1)	1.00E-241
T2-L2	blue	Csa5G604150	3.14	0.01	Redox	Thioredoxin 2 (TRX2)	
T2-L2	blue	Csa5G223070	3.216	0.003151	Transcription factor	WRKY DNA-binding protein 54 (WRKY54)	3.00E-96
T2-L2	blue	Csa2G359890	3.289	3.70E-03	Not assigned	unknown	
T2-L2	blue	Csa1G596520	3.4	0.0002165	Protein degradation	Cystein proteinases superfamily protein	2.00E-168
T2-L2	blue	Csa7G395800	3.415	0.001823	Miscellaneous	Glutathione S-transferase phi 8 (GSTF8)	3.00E-82
T2-L2	blue	Csa3G119700	3.575	0.00005376	Transcription factor	WRKY DNA-binding protein 53 (WRKY53)	1.00E-102
T2-L2	blue	Csa6G381850	3.831	0.001156	Transport	Amino acid permease 3 (AAP3)	2.00E-234
T2-L2	blue	Csa1G033160	4.495	0.006049	Miscellaneous	Glutathione S-transferase tau 9 (GSTU9)	3.00E-68
T2-L2	blue	Csa6G486960	4.496	0.000002502	Transcription factor	WRKY DNA-binding protein 51 (WRKY51)	1.00E-36
T2-L2	blue	Csa2G058620	5.345	0.003945	Abiotic stress	DNAJ heat shock N-terminal domain-containing protein	2.00E-137

Supplementary Table 1 (Continued)

T2-L2	blue	Csa1G574970	1.827	1.18E-05	Major CHO metabolism	Hexokinase 2 (HXK2)	7.00E-51
T2-L2	blue	Csa4G618520	1.837	0.0001765	Biodegradation of xenobiotic	UDP glycosyltransferase (UGT)	1.00E-132
T2-L2	blue	Csa2G003610	1.841	0.0003098	Lipid metabolism	Abnormal inflorescence meristem (AIM1)	3.00E-294
T2-L2	blue	Csa3G730930	1.847	0.00004243	Transport	Ammonium transporter 2 (AMT2)	8E-220
T2-L2	blue	Csa6G493850	1.86	1.50E-05	Not assigned	unknown	
T2-L2	blue	Csa3G159420	1.871	1.61E-03	Not assigned	unknown	
T2-L2	blue	Csa1G528610	1.874	0.00435	Lipid metabolism	Thylakoid membrane anion transporter 1 (ANTR1)	7.00E-236
T2-L2	blue	Csa1G181360	1.875	0.001947	Transport	non-intrinsic ABC protein 9 (NAP9)	3.00E-156
T2-L2	blue	Csa3G154410	2.05	1.63E-10	Not assigned	unknown	
T2-L2	blue	Csa3G183950	2.092	0.0004285	Abiotic stress	Heat shock protein 83	0.00E+00
T2-L2	blue	Csa2G296090	2.143	2.44E-04	Not assigned	unknown	
T2-L2	blue	Csa7G291720	2.169	0.006566	Lipid metabolism	Choline kinase	4.00E-133
T2-L2	blue	Csa2G427310	2.238	0.007581	Transcription factor	MYB domain protein 73 (MYB73)	1.00E-88
T2-L2	blue	Csa3G881750	2.24	0.009561	Lipid metabolism	PR5-like receptor kinase (PR5K)	4.00E-154
T2-L2	blue	Csa5G589260	2.243	0	Amino acid metabolism	O-acetylserine(thiol) lyase (OAS-TL)	4.00E-162
T2-L2	blue	Csa3G733350	2.317	0.00004435	Cell organization	RHO guanyl-nucleotide exchange factor 11 (ROPGEF11)	9.00E-41
T2-L2	blue	Csa2G004690	2.522	0.000004593	Transcription factor	Heat shock transcription factor C1 (HSFC1)	5.00E-67
T2-L2	blue	Csa7G387690	2.631	0.000001654	Transport	Sulfate transporter 3;1 (SULTR 3;1)	3.00E-307
T2-L2	blue	Csa1G008520	2.771	0.00804	Transport	Plasma membrane proton ATPase 4 (HA4)	0.00E+00
T2-L2	blue	Csa6G514850	3.048	0.00009803	Miscellaneous	Cytochrome P450 family 77 subfamily B polypeptide 1 (CYP77B1)	1.00E-241
T2-L2	blue	Csa5G604150	3.14	0.01	Redox	Thioredoxin 2 (TRX2)	
T2-L2	blue	Csa5G223070	3.216	0.003151	Transcription factor	WRKY DNA-binding protein 54 (WRKY54)	3.00E-96
T2-L2	blue	Csa2G359890	3.289	3.70E-03	Not assigned	unknown	
T2-L2	blue	Csa1G596520	3.4	0.0002165	Protein degradation	Cystein proteinases superfamily protein	2.00E-168
T2-L2	blue	Csa7G395800	3.415	0.001823	Miscellaneous	Glutathione S-transferase phi 8 (GSTF8)	3.00E-82
T2-L2	blue	Csa3G119700	3.575	0.00005376	Transcription factor	WRKY DNA-binding protein 53 (WRKY53)	1.00E-102
T2-L2	blue	Csa6G381850	3.831	0.001156	Transport	Amino acid permease 3 (AAP3)	2.00E-234
T2-L2	blue	Csa1G033160	4.495	0.006049	Miscellaneous	Glutathione S-transferase tau 9 (GSTU9)	3.00E-68
T2-L2	blue	Csa6G486960	4.496	0.000002502	Transcription factor	WRKY DNA-binding protein 51 (WRKY51)	1.00E-36
T2-L2	blue	Csa2G058620	5.345	0.003945	Abiotic stress	DNAJ heat shock N-terminal domain-containing protein	2.00E-137

Supplementary Table 1 (Continued)

T2-L2	blue	Csa1G574970	1.827	1.18E-05	Major CHO metabolism	Hexokinase 2 (HXK2)	7.00E-51
T2-L2	blue	Csa4G618520	1.837	0.0001765	Biodegradation of xenobiotic	UDP glycosyltransferase (UGT)	1.00E-132
T2-L2	blue	Csa2G003610	1.841	0.0003098	Lipid metabolism	Abnormal inflorescence meristem (AIM1)	3.00E-294
T2-L2	blue	Csa3G730930	1.847	0.00004243	Transport	Ammonium transporter 2 (AMT2)	8E-220
T2-L2	blue	Csa6G493850	1.86	1.50E-05	Not assigned	unknown	
T2-L2	blue	Csa3G159420	1.871	1.61E-03	Not assigned	unknown	
T2-L2	blue	Csa1G528610	1.874	0.00435	Lipid metabolism	Thylakoid membrane anion transporter 1 (ANTR1)	7.00E-236
T2-L2	blue	Csa1G181360	1.875	0.001947	Transport	non-intrinsic ABC protein 9 (NAP9)	3.00E-156
T2-L2	blue	Csa3G154410	2.05	1.63E-10	Not assigned	unknown	
T2-L2	blue	Csa3G183950	2.092	0.0004285	Abiotic stress	Heat shock protein 83	0.00E+00
T2-L2	blue	Csa2G296090	2.143	2.44E-04	Not assigned	unknown	
T2-L2	blue	Csa7G291720	2.169	0.006566	Lipid metabolism	Choline kinase	4.00E-133
T2-L2	blue	Csa2G427310	2.238	0.007581	Transcription factor	MYB domain protein 73 (MYB73)	1.00E-88
T2-L2	blue	Csa3G881750	2.24	0.009561	Lipid metabolism	PR5-like receptor kinase (PR5K)	4.00E-154
T2-L2	blue	Csa5G589260	2.243	0	Amino acid metabolism	O-acetylserine(thiol) lyase (OAS-TL)	4.00E-162
T2-L2	blue	Csa3G733350	2.317	0.00004435	Cell organization	RHO guanyl-nucleotide exchange factor 11 (ROPGEF11)	9.00E-41
T2-L2	blue	Csa2G004690	2.522	0.000004593	Transcription factor	Heat shock transcription factor C1 (HSFC1)	5.00E-67
T2-L2	blue	Csa7G387690	2.631	0.000001654	Transport	Sulfate transporter 3;1 (SULTR 3;1)	3.00E-307
T2-L2	blue	Csa1G008520	2.771	0.00804	Transport	Plasma membrane proton ATPase 4 (HA4)	0.00E+00
T2-L2	blue	Csa6G514850	3.048	0.00009803	Miscellaneous	Cytochrome P450 family 77 subfamily B polypeptide 1 (CYP77B1)	1.00E-241
T2-L2	blue	Csa5G604150	3.14	0.01	Redox	Thioredoxin 2 (TRX2)	
T2-L2	blue	Csa5G223070	3.216	0.003151	Transcription factor	WRKY DNA-binding protein 54 (WRKY54)	3.00E-96
T2-L2	blue	Csa2G359890	3.289	3.70E-03	Not assigned	unknown	
T2-L2	blue	Csa1G596520	3.4	0.0002165	Protein degradation	Cystein proteinases superfamily protein	2.00E-168
T2-L2	blue	Csa7G395800	3.415	0.001823	Miscellaneous	Glutathione S-transferase phi 8 (GSTF8)	3.00E-82
T2-L2	blue	Csa3G119700	3.575	0.00005376	Transcription factor	WRKY DNA-binding protein 53 (WRKY53)	1.00E-102
T2-L2	blue	Csa6G381850	3.831	0.001156	Transport	Amino acid permease 3 (AAP3)	2.00E-234
T2-L2	blue	Csa1G033160	4.495	0.006049	Miscellaneous	Glutathione S-transferase tau 9 (GSTU9)	3.00E-68
T2-L2	blue	Csa6G486960	4.496	0.000002502	Transcription factor	WRKY DNA-binding protein 51 (WRKY51)	1.00E-36
T2-L2	blue	Csa2G058620	5.345	0.003945	Abiotic stress	DNAJ heat shock N-terminal domain-containing protein	2.00E-137

Supplementary Table 1 (Continued)

T2-L2	blue	Csa1G574970	1.827	1.18E-05	Major CHO metabolism	Hexokinase 2 (HXK2)	7.00E-51
T2-L2	blue	Csa4G618520	1.837	0.0001765	Biodegradation of xenobiotic	UDP glycosyltransferase (UGT)	1.00E-132
T2-L2	blue	Csa2G003610	1.841	0.0003098	Lipid metabolism	Abnormal inflorescence meristem (AIM1)	3.00E-294
T2-L2	blue	Csa3G730930	1.847	0.00004243	Transport	Ammonium transporter 2 (AMT2)	8E-220
T2-L2	blue	Csa6G493850	1.86	1.50E-05	Not assigned	unknown	
T2-L2	blue	Csa3G159420	1.871	1.61E-03	Not assigned	unknown	
T2-L2	blue	Csa1G528610	1.874	0.00435	Lipid metabolism	Thylakoid membrane anion transporter 1 (ANTR1)	7.00E-236
T2-L2	blue	Csa1G181360	1.875	0.001947	Transport	non-intrinsic ABC protein 9 (NAP9)	3.00E-156
T2-L2	blue	Csa3G154410	2.05	1.63E-10	Not assigned	unknown	
T2-L2	blue	Csa3G183950	2.092	0.0004285	Abiotic stress	Heat shock protein 83	0.00E+00
T2-L2	blue	Csa2G296090	2.143	2.44E-04	Not assigned	unknown	
T2-L2	blue	Csa7G291720	2.169	0.006566	Lipid metabolism	Choline kinase	4.00E-133
T2-L2	blue	Csa2G427310	2.238	0.007581	Transcription factor	MYB domain protein 73 (MYB73)	1.00E-88
T2-L2	blue	Csa3G881750	2.24	0.009561	Lipid metabolism	PR5-like receptor kinase (PR5K)	4.00E-154
T2-L2	blue	Csa5G589260	2.243	0	Amino acid metabolism	O-acetylserine(thiol) lyase (OAS-TL)	4.00E-162
T2-L2	blue	Csa3G733350	2.317	0.00004435	Cell organization	RHO guanyl-nucleotide exchange factor 11 (ROPGEF11)	9.00E-41
T2-L2	blue	Csa2G004690	2.522	0.000004593	Transcription factor	Heat shock transcription factor C1 (HSFC1)	5.00E-67
T2-L2	blue	Csa7G387690	2.631	0.000001654	Transport	Sulfate transporter 3;1 (SULTR 3;1)	3.00E-307
T2-L2	blue	Csa1G008520	2.771	0.00804	Transport	Plasma membrane proton ATPase 4 (HA4)	0.00E+00
T2-L2	blue	Csa6G514850	3.048	0.00009803	Miscellaneous	Cytochrome P450 family 77 subfamily B polypeptide 1 (CYP77B1)	1.00E-241
T2-L2	blue	Csa5G604150	3.14	0.01	Redox	Thioredoxin 2 (TRX2)	
T2-L2	blue	Csa5G223070	3.216	0.003151	Transcription factor	WRKY DNA-binding protein 54 (WRKY54)	3.00E-96
T2-L2	blue	Csa2G359890	3.289	3.70E-03	Not assigned	unknown	
T2-L2	blue	Csa1G596520	3.4	0.0002165	Protein degradation	Cystein proteinases superfamily protein	2.00E-168
T2-L2	blue	Csa7G395800	3.415	0.001823	Miscellaneous	Glutathione S-transferase phi 8 (GSTF8)	3.00E-82
T2-L2	blue	Csa3G119700	3.575	0.00005376	Transcription factor	WRKY DNA-binding protein 53 (WRKY53)	1.00E-102
T2-L2	blue	Csa6G381850	3.831	0.001156	Transport	Amino acid permease 3 (AAP3)	2.00E-234
T2-L2	blue	Csa1G033160	4.495	0.006049	Miscellaneous	Glutathione S-transferase tau 9 (GSTU9)	3.00E-68
T2-L2	blue	Csa6G486960	4.496	0.000002502	Transcription factor	WRKY DNA-binding protein 51 (WRKY51)	1.00E-36
T2-L2	blue	Csa2G058620	5.345	0.003945	Abiotic stress	DNAJ heat shock N-terminal domain-containing protein	2.00E-137

Supplementary Table 1 (Continued)

T2-L2	blue	Csa1G574970	1.827	1.18E-05	Major CHO metabolism	Hexokinase 2 (HXK2)	7.00E-51
T2-L2	blue	Csa4G618520	1.837	0.0001765	Biodegradation of xenobiotic	UDP glycosyltransferase (UGT)	1.00E-132
T2-L2	blue	Csa2G003610	1.841	0.0003098	Lipid metabolism	Abnormal inflorescence meristem (AIM1)	3.00E-294
T2-L2	blue	Csa3G730930	1.847	0.00004243	Transport	Ammonium transporter 2 (AMT2)	8E-220
T2-L2	blue	Csa6G493850	1.86	1.50E-05	Not assigned	unknown	
T2-L2	blue	Csa3G159420	1.871	1.61E-03	Not assigned	unknown	
T2-L2	blue	Csa1G528610	1.874	0.00435	Lipid metabolism	Thylakoid membrane anion transporter 1 (ANTR1)	7.00E-236
T2-L2	blue	Csa1G181360	1.875	0.001947	Transport	non-intrinsic ABC protein 9 (NAP9)	3.00E-156
T2-L2	blue	Csa3G154410	2.05	1.63E-10	Not assigned	unknown	
T2-L2	blue	Csa3G183950	2.092	0.0004285	Abiotic stress	Heat shock protein 83	0.00E+00
T2-L2	blue	Csa2G296090	2.143	2.44E-04	Not assigned	unknown	
T2-L2	blue	Csa7G291720	2.169	0.006566	Lipid metabolism	Choline kinase	4.00E-133
T2-L2	blue	Csa2G427310	2.238	0.007581	Transcription factor	MYB domain protein 73 (MYB73)	1.00E-88
T2-L2	blue	Csa3G881750	2.24	0.009561	Lipid metabolism	PR5-like receptor kinase (PR5K)	4.00E-154
T2-L2	blue	Csa5G589260	2.243	0	Amino acid metabolism	O-acetylserine(thiol) lyase (OAS-TL)	4.00E-162
T2-L2	blue	Csa3G733350	2.317	0.00004435	Cell organization	RHO guanyl-nucleotide exchange factor 11 (ROPGEF11)	9.00E-41
T2-L2	blue	Csa2G004690	2.522	0.000004593	Transcription factor	Heat shock transcription factor C1 (HSFC1)	5.00E-67
T2-L2	blue	Csa7G387690	2.631	0.000001654	Transport	Sulfate transporter 3;1 (SULTR 3;1)	3.00E-307
T2-L2	blue	Csa1G008520	2.771	0.00804	Transport	Plasma membrane proton ATPase 4 (HA4)	0.00E+00
T2-L2	blue	Csa6G514850	3.048	0.00009803	Miscellaneous	Cytochrome P450 family 77 subfamily B polypeptide 1 (CYP77B1)	1.00E-241
T2-L2	blue	Csa5G604150	3.14	0.01	Redox	Thioredoxin 2 (TRX2)	
T2-L2	blue	Csa5G223070	3.216	0.003151	Transcription factor	WRKY DNA-binding protein 54 (WRKY54)	3.00E-96
T2-L2	blue	Csa2G359890	3.289	3.70E-03	Not assigned	unknown	
T2-L2	blue	Csa1G596520	3.4	0.0002165	Protein degradation	Cystein proteinases superfamily protein	2.00E-168
T2-L2	blue	Csa7G395800	3.415	0.001823	Miscellaneous	Glutathione S-transferase phi 8 (GSTF8)	3.00E-82
T2-L2	blue	Csa3G119700	3.575	0.00005376	Transcription factor	WRKY DNA-binding protein 53 (WRKY53)	1.00E-102
T2-L2	blue	Csa6G381850	3.831	0.001156	Transport	Amino acid permease 3 (AAP3)	2.00E-234
T2-L2	blue	Csa1G033160	4.495	0.006049	Miscellaneous	Glutathione S-transferase tau 9 (GSTU9)	3.00E-68
T2-L2	blue	Csa6G486960	4.496	0.000002502	Transcription factor	WRKY DNA-binding protein 51 (WRKY51)	1.00E-36
T2-L2	blue	Csa2G058620	5.345	0.003945	Abiotic stress	DNAJ heat shock N-terminal domain-containing protein	2.00E-137

Supplementary Table 1 (Continued)

T2-L2	blue	Csa1G574970	1.827	1.18E-05	Major CHO metabolism	Hexokinase 2 (HXK2)	7.00E-51
T2-L2	blue	Csa4G618520	1.837	0.0001765	Biodegradation of xenobiotic	UDP glycosyltransferase (UGT)	1.00E-132
T2-L2	blue	Csa2G003610	1.841	0.0003098	Lipid metabolism	Abnormal inflorescence meristem (AIM1)	3.00E-294
T2-L2	blue	Csa3G730930	1.847	0.00004243	Transport	Ammonium transporter 2 (AMT2)	8E-220
T2-L2	blue	Csa6G493850	1.86	1.50E-05	Not assigned	unknown	
T2-L2	blue	Csa3G159420	1.871	1.61E-03	Not assigned	unknown	
T2-L2	blue	Csa1G528610	1.874	0.00435	Lipid metabolism	Thylakoid membrane anion transporter 1 (ANTR1)	7.00E-236
T2-L2	blue	Csa1G181360	1.875	0.001947	Transport	non-intrinsic ABC protein 9 (NAP9)	3.00E-156
T2-L2	blue	Csa3G154410	2.05	1.63E-10	Not assigned	unknown	
T2-L2	blue	Csa3G183950	2.092	0.0004285	Abiotic stress	Heat shock protein 83	0.00E+00
T2-L2	blue	Csa2G296090	2.143	2.44E-04	Not assigned	unknown	
T2-L2	blue	Csa7G291720	2.169	0.006566	Lipid metabolism	Choline kinase	4.00E-133
T2-L2	blue	Csa2G427310	2.238	0.007581	Transcription factor	MYB domain protein 73 (MYB73)	1.00E-88
T2-L2	blue	Csa3G881750	2.24	0.009561	Lipid metabolism	PR5-like receptor kinase (PR5K)	4.00E-154
T2-L2	blue	Csa5G589260	2.243	0	Amino acid metabolism	O-acetylserine(thiol) lyase (OAS-TL)	4.00E-162
T2-L2	blue	Csa3G733350	2.317	0.00004435	Cell organization	RHO guanyl-nucleotide exchange factor 11 (ROPGEF11)	9.00E-41
T2-L2	blue	Csa2G004690	2.522	0.000004593	Transcription factor	Heat shock transcription factor C1 (HSFC1)	5.00E-67
T2-L2	blue	Csa7G387690	2.631	0.000001654	Transport	Sulfate transporter 3;1 (SULTR 3;1)	3.00E-307
T2-L2	blue	Csa1G008520	2.771	0.00804	Transport	Plasma membrane proton ATPase 4 (HA4)	0.00E+00
T2-L2	blue	Csa6G514850	3.048	0.00009803	Miscellaneous	Cytochrome P450 family 77 subfamily B polypeptide 1 (CYP77B1)	1.00E-241
T2-L2	blue	Csa5G604150	3.14	0.01	Redox	Thioredoxin 2 (TRX2)	
T2-L2	blue	Csa5G223070	3.216	0.003151	Transcription factor	WRKY DNA-binding protein 54 (WRKY54)	3.00E-96
T2-L2	blue	Csa2G359890	3.289	3.70E-03	Not assigned	unknown	
T2-L2	blue	Csa1G596520	3.4	0.0002165	Protein degradation	Cystein proteinases superfamily protein	2.00E-168
T2-L2	blue	Csa7G395800	3.415	0.001823	Miscellaneous	Glutathione S-transferase phi 8 (GSTF8)	3.00E-82
T2-L2	blue	Csa3G119700	3.575	0.00005376	Transcription factor	WRKY DNA-binding protein 53 (WRKY53)	1.00E-102
T2-L2	blue	Csa6G381850	3.831	0.001156	Transport	Amino acid permease 3 (AAP3)	2.00E-234
T2-L2	blue	Csa1G033160	4.495	0.006049	Miscellaneous	Glutathione S-transferase tau 9 (GSTU9)	3.00E-68
T2-L2	blue	Csa6G486960	4.496	0.000002502	Transcription factor	WRKY DNA-binding protein 51 (WRKY51)	1.00E-36
T2-L2	blue	Csa2G058620	5.345	0.003945	Abiotic stress	DNAJ heat shock N-terminal domain-containing protein	2.00E-137

Supplementary Table 1 (Continued)

T2-L2	blue	Csa1G574970	1.827	1.18E-05	Major CHO metabolism	Hexokinase 2 (HXK2)	7.00E-51
T2-L2	blue	Csa4G618520	1.837	0.0001765	Biodegradation of xenobiotic	UDP glycosyltransferase (UGT)	1.00E-132
T2-L2	blue	Csa2G003610	1.841	0.0003098	Lipid metabolism	Abnormal inflorescence meristem (AIM1)	3.00E-294
T2-L2	blue	Csa3G730930	1.847	0.00004243	Transport	Ammonium transporter 2 (AMT2)	8E-220
T2-L2	blue	Csa6G493850	1.86	1.50E-05	Not assigned	unknown	
T2-L2	blue	Csa3G159420	1.871	1.61E-03	Not assigned	unknown	
T2-L2	blue	Csa1G528610	1.874	0.00435	Lipid metabolism	Thylakoid membrane anion transporter 1 (ANTR1)	7.00E-236
T2-L2	blue	Csa1G181360	1.875	0.001947	Transport	non-intrinsic ABC protein 9 (NAP9)	3.00E-156
T2-L2	blue	Csa3G154410	2.05	1.63E-10	Not assigned	unknown	
T2-L2	blue	Csa3G183950	2.092	0.0004285	Abiotic stress	Heat shock protein 83	0.00E+00
T2-L2	blue	Csa2G296090	2.143	2.44E-04	Not assigned	unknown	
T2-L2	blue	Csa7G291720	2.169	0.006566	Lipid metabolism	Choline kinase	4.00E-133
T2-L2	blue	Csa2G427310	2.238	0.007581	Transcription factor	MYB domain protein 73 (MYB73)	1.00E-88
T2-L2	blue	Csa3G881750	2.24	0.009561	Lipid metabolism	PR5-like receptor kinase (PR5K)	4.00E-154
T2-L2	blue	Csa5G589260	2.243	0	Amino acid metabolism	O-acetylserine(thiol) lyase (OAS-TL)	4.00E-162
T2-L2	blue	Csa3G733350	2.317	0.00004435	Cell organization	RHO guanyl-nucleotide exchange factor 11 (ROPGEF11)	9.00E-41
T2-L2	blue	Csa2G004690	2.522	0.000004593	Transcription factor	Heat shock transcription factor C1 (HSFC1)	5.00E-67
T2-L2	blue	Csa7G387690	2.631	0.000001654	Transport	Sulfate transporter 3;1 (SULTR 3;1)	3.00E-307
T2-L2	blue	Csa1G008520	2.771	0.00804	Transport	Plasma membrane proton ATPase 4 (HA4)	0.00E+00
T2-L2	blue	Csa6G514850	3.048	0.00009803	Miscellaneous	Cytochrome P450 family 77 subfamily B polypeptide 1 (CYP77B1)	1.00E-241
T2-L2	blue	Csa5G604150	3.14	0.01	Redox	Thioredoxin 2 (TRX2)	
T2-L2	blue	Csa5G223070	3.216	0.003151	Transcription factor	WRKY DNA-binding protein 54 (WRKY54)	3.00E-96
T2-L2	blue	Csa2G359890	3.289	3.70E-03	Not assigned	unknown	
T2-L2	blue	Csa1G596520	3.4	0.0002165	Protein degradation	Cystein proteinases superfamily protein	2.00E-168
T2-L2	blue	Csa7G395800	3.415	0.001823	Miscellaneous	Glutathione S-transferase phi 8 (GSTF8)	3.00E-82
T2-L2	blue	Csa3G119700	3.575	0.00005376	Transcription factor	WRKY DNA-binding protein 53 (WRKY53)	1.00E-102
T2-L2	blue	Csa6G381850	3.831	0.001156	Transport	Amino acid permease 3 (AAP3)	2.00E-234
T2-L2	blue	Csa1G033160	4.495	0.006049	Miscellaneous	Glutathione S-transferase tau 9 (GSTU9)	3.00E-68
T2-L2	blue	Csa6G486960	4.496	0.000002502	Transcription factor	WRKY DNA-binding protein 51 (WRKY51)	1.00E-36
T2-L2	blue	Csa2G058620	5.345	0.003945	Abiotic stress	DNAJ heat shock N-terminal domain-containing protein	2.00E-137

Supplementary Table 1 (Continued)

T2-L2	blue	Csa1G574970	1.827	1.18E-05	Major CHO metabolism	Hexokinase 2 (HXK2)	7.00E-51
T2-L2	blue	Csa4G618520	1.837	0.0001765	Biodegradation of xenobiotic	UDP glycosyltransferase (UGT)	1.00E-132
T2-L2	blue	Csa2G003610	1.841	0.0003098	Lipid metabolism	Abnormal inflorescence meristem (AIM1)	3.00E-294
T2-L2	blue	Csa3G730930	1.847	0.00004243	Transport	Ammonium transporter 2 (AMT2)	8E-220
T2-L2	blue	Csa6G493850	1.86	1.50E-05	Not assigned	unknown	
T2-L2	blue	Csa3G159420	1.871	1.61E-03	Not assigned	unknown	
T2-L2	blue	Csa1G528610	1.874	0.00435	Lipid metabolism	Thylakoid membrane anion transporter 1 (ANTR1)	7.00E-236
T2-L2	blue	Csa1G181360	1.875	0.001947	Transport	non-intrinsic ABC protein 9 (NAP9)	3.00E-156
T2-L2	blue	Csa3G154410	2.05	1.63E-10	Not assigned	unknown	
T2-L2	blue	Csa3G183950	2.092	0.0004285	Abiotic stress	Heat shock protein 83	0.00E+00
T2-L2	blue	Csa2G296090	2.143	2.44E-04	Not assigned	unknown	
T2-L2	blue	Csa7G291720	2.169	0.006566	Lipid metabolism	Choline kinase	4.00E-133
T2-L2	blue	Csa2G427310	2.238	0.007581	Transcription factor	MYB domain protein 73 (MYB73)	1.00E-88
T2-L2	blue	Csa3G881750	2.24	0.009561	Lipid metabolism	PR5-like receptor kinase (PR5K)	4.00E-154
T2-L2	blue	Csa5G589260	2.243	0	Amino acid metabolism	O-acetylserine(thiol) lyase (OAS-TL)	4.00E-162
T2-L2	blue	Csa3G733350	2.317	0.00004435	Cell organization	RHO guanyl-nucleotide exchange factor 11 (ROPGEF11)	9.00E-41
T2-L2	blue	Csa2G004690	2.522	0.000004593	Transcription factor	Heat shock transcription factor C1 (HSFC1)	5.00E-67
T2-L2	blue	Csa7G387690	2.631	0.000001654	Transport	Sulfate transporter 3;1 (SULTR 3;1)	3.00E-307
T2-L2	blue	Csa1G008520	2.771	0.00804	Transport	Plasma membrane proton ATPase 4 (HA4)	0.00E+00
T2-L2	blue	Csa6G514850	3.048	0.00009803	Miscellaneous	Cytochrome P450 family 77 subfamily B polypeptide 1 (CYP77B1)	1.00E-241
T2-L2	blue	Csa5G604150	3.14	0.01	Redox	Thioredoxin 2 (TRX2)	
T2-L2	blue	Csa5G223070	3.216	0.003151	Transcription factor	WRKY DNA-binding protein 54 (WRKY54)	3.00E-96
T2-L2	blue	Csa2G359890	3.289	3.70E-03	Not assigned	unknown	
T2-L2	blue	Csa1G596520	3.4	0.0002165	Protein degradation	Cystein proteinases superfamily protein	2.00E-168
T2-L2	blue	Csa7G395800	3.415	0.001823	Miscellaneous	Glutathione S-transferase phi 8 (GSTF8)	3.00E-82
T2-L2	blue	Csa3G119700	3.575	0.00005376	Transcription factor	WRKY DNA-binding protein 53 (WRKY53)	1.00E-102
T2-L2	blue	Csa6G381850	3.831	0.001156	Transport	Amino acid permease 3 (AAP3)	2.00E-234
T2-L2	blue	Csa1G033160	4.495	0.006049	Miscellaneous	Glutathione S-transferase tau 9 (GSTU9)	3.00E-68
T2-L2	blue	Csa6G486960	4.496	0.000002502	Transcription factor	WRKY DNA-binding protein 51 (WRKY51)	1.00E-36
T2-L2	blue	Csa2G058620	5.345	0.003945	Abiotic stress	DNAJ heat shock N-terminal domain-containing protein	2.00E-137

Supplementary Table 1 (Continued)

T2-L2	blue	Csa1G574970	1.827	1.18E-05	Major CHO metabolism	Hexokinase 2 (HXK2)	7.00E-51
T2-L2	blue	Csa4G618520	1.837	0.0001765	Biodegradation of xenobiotic	UDP glycosyltransferase (UGT)	1.00E-132
T2-L2	blue	Csa2G003610	1.841	0.0003098	Lipid metabolism	Abnormal inflorescence meristem (AIM1)	3.00E-294
T2-L2	blue	Csa3G730930	1.847	0.00004243	Transport	Ammonium transporter 2 (AMT2)	8E-220
T2-L2	blue	Csa6G493850	1.86	1.50E-05	Not assigned	unknown	
T2-L2	blue	Csa3G159420	1.871	1.61E-03	Not assigned	unknown	
T2-L2	blue	Csa1G528610	1.874	0.00435	Lipid metabolism	Thylakoid membrane anion transporter 1 (ANTR1)	7.00E-236
T2-L2	blue	Csa1G181360	1.875	0.001947	Transport	non-intrinsic ABC protein 9 (NAP9)	3.00E-156
T2-L2	blue	Csa3G154410	2.05	1.63E-10	Not assigned	unknown	
T2-L2	blue	Csa3G183950	2.092	0.0004285	Abiotic stress	Heat shock protein 83	0.00E+00
T2-L2	blue	Csa2G296090	2.143	2.44E-04	Not assigned	unknown	
T2-L2	blue	Csa7G291720	2.169	0.006566	Lipid metabolism	Choline kinase	4.00E-133
T2-L2	blue	Csa2G427310	2.238	0.007581	Transcription factor	MYB domain protein 73 (MYB73)	1.00E-88
T2-L2	blue	Csa3G881750	2.24	0.009561	Lipid metabolism	PR5-like receptor kinase (PR5K)	4.00E-154
T2-L2	blue	Csa5G589260	2.243	0	Amino acid metabolism	O-acetylserine(thiol) lyase (OAS-TL)	4.00E-162
T2-L2	blue	Csa3G733350	2.317	0.00004435	Cell organization	RHO guanyl-nucleotide exchange factor 11 (ROPGEF11)	9.00E-41
T2-L2	blue	Csa2G004690	2.522	0.000004593	Transcription factor	Heat shock transcription factor C1 (HSFC1)	5.00E-67
T2-L2	blue	Csa7G387690	2.631	0.000001654	Transport	Sulfate transporter 3;1 (SULTR 3;1)	3.00E-307
T2-L2	blue	Csa1G008520	2.771	0.00804	Transport	Plasma membrane proton ATPase 4 (HA4)	0.00E+00
T2-L2	blue	Csa6G514850	3.048	0.00009803	Miscellaneous	Cytochrome P450 family 77 subfamily B polypeptide 1 (CYP77B1)	1.00E-241
T2-L2	blue	Csa5G604150	3.14	0.01	Redox	Thioredoxin 2 (TRX2)	
T2-L2	blue	Csa5G223070	3.216	0.003151	Transcription factor	WRKY DNA-binding protein 54 (WRKY54)	3.00E-96
T2-L2	blue	Csa2G359890	3.289	3.70E-03	Not assigned	unknown	
T2-L2	blue	Csa1G596520	3.4	0.0002165	Protein degradation	Cystein proteinases superfamily protein	2.00E-168
T2-L2	blue	Csa7G395800	3.415	0.001823	Miscellaneous	Glutathione S-transferase phi 8 (GSTF8)	3.00E-82
T2-L2	blue	Csa3G119700	3.575	0.00005376	Transcription factor	WRKY DNA-binding protein 53 (WRKY53)	1.00E-102
T2-L2	blue	Csa6G381850	3.831	0.001156	Transport	Amino acid permease 3 (AAP3)	2.00E-234
T2-L2	blue	Csa1G033160	4.495	0.006049	Miscellaneous	Glutathione S-transferase tau 9 (GSTU9)	3.00E-68
T2-L2	blue	Csa6G486960	4.496	0.000002502	Transcription factor	WRKY DNA-binding protein 51 (WRKY51)	1.00E-36
T2-L2	blue	Csa2G058620	5.345	0.003945	Abiotic stress	DNAJ heat shock N-terminal domain-containing protein	2.00E-137

Supplementary Table 1 (Continued)

T2-L2	blue	Csa1G574970	1.827	1.18E-05	Major CHO metabolism	Hexokinase 2 (HXK2)	7.00E-51
T2-L2	blue	Csa4G618520	1.837	0.0001765	Biodegradation of xenobiotic	UDP glycosyltransferase (UGT)	1.00E-132
T2-L2	blue	Csa2G003610	1.841	0.0003098	Lipid metabolism	Abnormal inflorescence meristem (AIM1)	3.00E-294
T2-L2	blue	Csa3G730930	1.847	0.00004243	Transport	Ammonium transporter 2 (AMT2)	8E-220
T2-L2	blue	Csa6G493850	1.86	1.50E-05	Not assigned	unknown	
T2-L2	blue	Csa3G159420	1.871	1.61E-03	Not assigned	unknown	
T2-L2	blue	Csa1G528610	1.874	0.00435	Lipid metabolism	Thylakoid membrane anion transporter 1 (ANTR1)	7.00E-236
T2-L2	blue	Csa1G181360	1.875	0.001947	Transport	non-intrinsic ABC protein 9 (NAP9)	3.00E-156
T2-L2	blue	Csa3G154410	2.05	1.63E-10	Not assigned	unknown	
T2-L2	blue	Csa3G183950	2.092	0.0004285	Abiotic stress	Heat shock protein 83	0.00E+00
T2-L2	blue	Csa2G296090	2.143	2.44E-04	Not assigned	unknown	
T2-L2	blue	Csa7G291720	2.169	0.006566	Lipid metabolism	Choline kinase	4.00E-133
T2-L2	blue	Csa2G427310	2.238	0.007581	Transcription factor	MYB domain protein 73 (MYB73)	1.00E-88
T2-L2	blue	Csa3G881750	2.24	0.009561	Lipid metabolism	PR5-like receptor kinase (PR5K)	4.00E-154
T2-L2	blue	Csa5G589260	2.243	0	Amino acid metabolism	O-acetylserine(thiol) lyase (OAS-TL)	4.00E-162
T2-L2	blue	Csa3G733350	2.317	0.00004435	Cell organization	RHO guanyl-nucleotide exchange factor 11 (ROPGEF11)	9.00E-41
T2-L2	blue	Csa2G004690	2.522	0.000004593	Transcription factor	Heat shock transcription factor C1 (HSFC1)	5.00E-67
T2-L2	blue	Csa7G387690	2.631	0.000001654	Transport	Sulfate transporter 3;1 (SULTR 3;1)	3.00E-307
T2-L2	blue	Csa1G008520	2.771	0.00804	Transport	Plasma membrane proton ATPase 4 (HA4)	0.00E+00
T2-L2	blue	Csa6G514850	3.048	0.00009803	Miscellaneous	Cytochrome P450 family 77 subfamily B polypeptide 1 (CYP77B1)	1.00E-241
T2-L2	blue	Csa5G604150	3.14	0.01	Redox	Thioredoxin 2 (TRX2)	
T2-L2	blue	Csa5G223070	3.216	0.003151	Transcription factor	WRKY DNA-binding protein 54 (WRKY54)	3.00E-96
T2-L2	blue	Csa2G359890	3.289	3.70E-03	Not assigned	unknown	
T2-L2	blue	Csa1G596520	3.4	0.0002165	Protein degradation	Cystein proteinases superfamily protein	2.00E-168
T2-L2	blue	Csa7G395800	3.415	0.001823	Miscellaneous	Glutathione S-transferase phi 8 (GSTF8)	3.00E-82
T2-L2	blue	Csa3G119700	3.575	0.00005376	Transcription factor	WRKY DNA-binding protein 53 (WRKY53)	1.00E-102
T2-L2	blue	Csa6G381850	3.831	0.001156	Transport	Amino acid permease 3 (AAP3)	2.00E-234
T2-L2	blue	Csa1G033160	4.495	0.006049	Miscellaneous	Glutathione S-transferase tau 9 (GSTU9)	3.00E-68
T2-L2	blue	Csa6G486960	4.496	0.000002502	Transcription factor	WRKY DNA-binding protein 51 (WRKY51)	1.00E-36
T2-L2	blue	Csa2G058620	5.345	0.003945	Abiotic stress	DNAJ heat shock N-terminal domain-containing protein	2.00E-137

Supplementary Table 1 (Continued)

T2-L2	blue	Csa1G574970	1.827	1.18E-05	Major CHO metabolism	Hexokinase 2 (HXK2)	7.00E-51
T2-L2	blue	Csa4G618520	1.837	0.0001765	Biodegradation of xenobiotic	UDP glycosyltransferase (UGT)	1.00E-132
T2-L2	blue	Csa2G003610	1.841	0.0003098	Lipid metabolism	Abnormal inflorescence meristem (AIM1)	3.00E-294
T2-L2	blue	Csa3G730930	1.847	0.00004243	Transport	Ammonium transporter 2 (AMT2)	8E-220
T2-L2	blue	Csa6G493850	1.86	1.50E-05	Not assigned	unknown	
T2-L2	blue	Csa3G159420	1.871	1.61E-03	Not assigned	unknown	
T2-L2	blue	Csa1G528610	1.874	0.00435	Lipid metabolism	Thylakoid membrane anion transporter 1 (ANTR1)	7.00E-236
T2-L2	blue	Csa1G181360	1.875	0.001947	Transport	non-intrinsic ABC protein 9 (NAP9)	3.00E-156
T2-L2	blue	Csa3G154410	2.05	1.63E-10	Not assigned	unknown	
T2-L2	blue	Csa3G183950	2.092	0.0004285	Abiotic stress	Heat shock protein 83	0.00E+00
T2-L2	blue	Csa2G296090	2.143	2.44E-04	Not assigned	unknown	
T2-L2	blue	Csa7G291720	2.169	0.006566	Lipid metabolism	Choline kinase	4.00E-133
T2-L2	blue	Csa2G427310	2.238	0.007581	Transcription factor	MYB domain protein 73 (MYB73)	1.00E-88
T2-L2	blue	Csa3G881750	2.24	0.009561	Lipid metabolism	PR5-like receptor kinase (PR5K)	4.00E-154
T2-L2	blue	Csa5G589260	2.243	0	Amino acid metabolism	O-acetylserine(thiol) lyase (OAS-TL)	4.00E-162
T2-L2	blue	Csa3G733350	2.317	0.00004435	Cell organization	RHO guanyl-nucleotide exchange factor 11 (ROPGEF11)	9.00E-41
T2-L2	blue	Csa2G004690	2.522	0.000004593	Transcription factor	Heat shock transcription factor C1 (HSFC1)	5.00E-67
T2-L2	blue	Csa7G387690	2.631	0.000001654	Transport	Sulfate transporter 3;1 (SULTR 3;1)	3.00E-307
T2-L2	blue	Csa1G008520	2.771	0.00804	Transport	Plasma membrane proton ATPase 4 (HA4)	0.00E+00
T2-L2	blue	Csa6G514850	3.048	0.00009803	Miscellaneous	Cytochrome P450 family 77 subfamily B polypeptide 1 (CYP77B1)	1.00E-241
T2-L2	blue	Csa5G604150	3.14	0.01	Redox	Thioredoxin 2 (TRX2)	
T2-L2	blue	Csa5G223070	3.216	0.003151	Transcription factor	WRKY DNA-binding protein 54 (WRKY54)	3.00E-96
T2-L2	blue	Csa2G359890	3.289	3.70E-03	Not assigned	unknown	
T2-L2	blue	Csa1G596520	3.4	0.0002165	Protein degradation	Cystein proteinases superfamily protein	2.00E-168
T2-L2	blue	Csa7G395800	3.415	0.001823	Miscellaneous	Glutathione S-transferase phi 8 (GSTF8)	3.00E-82
T2-L2	blue	Csa3G119700	3.575	0.00005376	Transcription factor	WRKY DNA-binding protein 53 (WRKY53)	1.00E-102
T2-L2	blue	Csa6G381850	3.831	0.001156	Transport	Amino acid permease 3 (AAP3)	2.00E-234
T2-L2	blue	Csa1G033160	4.495	0.006049	Miscellaneous	Glutathione S-transferase tau 9 (GSTU9)	3.00E-68
T2-L2	blue	Csa6G486960	4.496	0.000002502	Transcription factor	WRKY DNA-binding protein 51 (WRKY51)	1.00E-36
T2-L2	blue	Csa2G058620	5.345	0.003945	Abiotic stress	DNAJ heat shock N-terminal domain-containing protein	2.00E-137

Supplementary Table 1 (Continued)

T2-L2	blue	Csa1G574970	1.827	1.18E-05	Major CHO metabolism	Hexokinase 2 (HXK2)	7.00E-51
T2-L2	blue	Csa4G618520	1.837	0.0001765	Biodegradation of xenobiotic	UDP glycosyltransferase (UGT)	1.00E-132
T2-L2	blue	Csa2G003610	1.841	0.0003098	Lipid metabolism	Abnormal inflorescence meristem (AIM1)	3.00E-294
T2-L2	blue	Csa3G730930	1.847	0.00004243	Transport	Ammonium transporter 2 (AMT2)	8E-220
T2-L2	blue	Csa6G493850	1.86	1.50E-05	Not assigned	unknown	
T2-L2	blue	Csa3G159420	1.871	1.61E-03	Not assigned	unknown	
T2-L2	blue	Csa1G528610	1.874	0.00435	Lipid metabolism	Thylakoid membrane anion transporter 1 (ANTR1)	7.00E-236
T2-L2	blue	Csa1G181360	1.875	0.001947	Transport	non-intrinsic ABC protein 9 (NAP9)	3.00E-156
T2-L2	blue	Csa3G154410	2.05	1.63E-10	Not assigned	unknown	
T2-L2	blue	Csa3G183950	2.092	0.0004285	Abiotic stress	Heat shock protein 83	0.00E+00
T2-L2	blue	Csa2G296090	2.143	2.44E-04	Not assigned	unknown	
T2-L2	blue	Csa7G291720	2.169	0.006566	Lipid metabolism	Choline kinase	4.00E-133
T2-L2	blue	Csa2G427310	2.238	0.007581	Transcription factor	MYB domain protein 73 (MYB73)	1.00E-88
T2-L2	blue	Csa3G881750	2.24	0.009561	Lipid metabolism	PR5-like receptor kinase (PR5K)	4.00E-154
T2-L2	blue	Csa5G589260	2.243	0	Amino acid metabolism	O-acetylserine(thiol) lyase (OAS-TL)	4.00E-162
T2-L2	blue	Csa3G733350	2.317	0.00004435	Cell organization	RHO guanyl-nucleotide exchange factor 11 (ROPGEF11)	9.00E-41
T2-L2	blue	Csa2G004690	2.522	0.000004593	Transcription factor	Heat shock transcription factor C1 (HSFC1)	5.00E-67
T2-L2	blue	Csa7G387690	2.631	0.000001654	Transport	Sulfate transporter 3;1 (SULTR 3;1)	3.00E-307
T2-L2	blue	Csa1G008520	2.771	0.00804	Transport	Plasma membrane proton ATPase 4 (HA4)	0.00E+00
T2-L2	blue	Csa6G514850	3.048	0.00009803	Miscellaneous	Cytochrome P450 family 77 subfamily B polypeptide 1 (CYP77B1)	1.00E-241
T2-L2	blue	Csa5G604150	3.14	0.01	Redox	Thioredoxin 2 (TRX2)	
T2-L2	blue	Csa5G223070	3.216	0.003151	Transcription factor	WRKY DNA-binding protein 54 (WRKY54)	3.00E-96
T2-L2	blue	Csa2G359890	3.289	3.70E-03	Not assigned	unknown	
T2-L2	blue	Csa1G596520	3.4	0.0002165	Protein degradation	Cystein proteinases superfamily protein	2.00E-168
T2-L2	blue	Csa7G395800	3.415	0.001823	Miscellaneous	Glutathione S-transferase phi 8 (GSTF8)	3.00E-82
T2-L2	blue	Csa3G119700	3.575	0.00005376	Transcription factor	WRKY DNA-binding protein 53 (WRKY53)	1.00E-102
T2-L2	blue	Csa6G381850	3.831	0.001156	Transport	Amino acid permease 3 (AAP3)	2.00E-234
T2-L2	blue	Csa1G033160	4.495	0.006049	Miscellaneous	Glutathione S-transferase tau 9 (GSTU9)	3.00E-68
T2-L2	blue	Csa6G486960	4.496	0.000002502	Transcription factor	WRKY DNA-binding protein 51 (WRKY51)	1.00E-36
T2-L2	blue	Csa2G058620	5.345	0.003945	Abiotic stress	DNAJ heat shock N-terminal domain-containing protein	2.00E-137

Supplementary Table 1 (Continued)

T2-L2	blue	Csa1G574970	1.827	1.18E-05	Major CHO metabolism	Hexokinase 2 (HXK2)	7.00E-51
T2-L2	blue	Csa4G618520	1.837	0.0001765	Biodegradation of xenobiotic	UDP glycosyltransferase (UGT)	1.00E-132
T2-L2	blue	Csa2G003610	1.841	0.0003098	Lipid metabolism	Abnormal inflorescence meristem (AIM1)	3.00E-294
T2-L2	blue	Csa3G730930	1.847	0.00004243	Transport	Ammonium transporter 2 (AMT2)	8E-220
T2-L2	blue	Csa6G493850	1.86	1.50E-05	Not assigned	unknown	
T2-L2	blue	Csa3G159420	1.871	1.61E-03	Not assigned	unknown	
T2-L2	blue	Csa1G528610	1.874	0.00435	Lipid metabolism	Thylakoid membrane anion transporter 1 (ANTR1)	7.00E-236
T2-L2	blue	Csa1G181360	1.875	0.001947	Transport	non-intrinsic ABC protein 9 (NAP9)	3.00E-156
T2-L2	blue	Csa3G154410	2.05	1.63E-10	Not assigned	unknown	
T2-L2	blue	Csa3G183950	2.092	0.0004285	Abiotic stress	Heat shock protein 83	0.00E+00
T2-L2	blue	Csa2G296090	2.143	2.44E-04	Not assigned	unknown	
T2-L2	blue	Csa7G291720	2.169	0.006566	Lipid metabolism	Choline kinase	4.00E-133
T2-L2	blue	Csa2G427310	2.238	0.007581	Transcription factor	MYB domain protein 73 (MYB73)	1.00E-88
T2-L2	blue	Csa3G881750	2.24	0.009561	Lipid metabolism	PR5-like receptor kinase (PR5K)	4.00E-154
T2-L2	blue	Csa5G589260	2.243	0	Amino acid metabolism	O-acetylserine(thiol) lyase (OAS-TL)	4.00E-162
T2-L2	blue	Csa3G733350	2.317	0.00004435	Cell organization	RHO guanyl-nucleotide exchange factor 11 (ROPGEF11)	9.00E-41
T2-L2	blue	Csa2G004690	2.522	0.000004593	Transcription factor	Heat shock transcription factor C1 (HSFC1)	5.00E-67
T2-L2	blue	Csa7G387690	2.631	0.000001654	Transport	Sulfate transporter 3;1 (SULTR 3;1)	3.00E-307
T2-L2	blue	Csa1G008520	2.771	0.00804	Transport	Plasma membrane proton ATPase 4 (HA4)	0.00E+00
T2-L2	blue	Csa6G514850	3.048	0.00009803	Miscellaneous	Cytochrome P450 family 77 subfamily B polypeptide 1 (CYP77B1)	1.00E-241
T2-L2	blue	Csa5G604150	3.14	0.01	Redox	Thioredoxin 2 (TRX2)	
T2-L2	blue	Csa5G223070	3.216	0.003151	Transcription factor	WRKY DNA-binding protein 54 (WRKY54)	3.00E-96
T2-L2	blue	Csa2G359890	3.289	3.70E-03	Not assigned	unknown	
T2-L2	blue	Csa1G596520	3.4	0.0002165	Protein degradation	Cystein proteinases superfamily protein	2.00E-168
T2-L2	blue	Csa7G395800	3.415	0.001823	Miscellaneous	Glutathione S-transferase phi 8 (GSTF8)	3.00E-82
T2-L2	blue	Csa3G119700	3.575	0.00005376	Transcription factor	WRKY DNA-binding protein 53 (WRKY53)	1.00E-102
T2-L2	blue	Csa6G381850	3.831	0.001156	Transport	Amino acid permease 3 (AAP3)	2.00E-234
T2-L2	blue	Csa1G033160	4.495	0.006049	Miscellaneous	Glutathione S-transferase tau 9 (GSTU9)	3.00E-68
T2-L2	blue	Csa6G486960	4.496	0.000002502	Transcription factor	WRKY DNA-binding protein 51 (WRKY51)	1.00E-36
T2-L2	blue	Csa2G058620	5.345	0.003945	Abiotic stress	DNAJ heat shock N-terminal domain-containing protein	2.00E-137

Supplementary Table 1 (Continued)

T2-L2	blue	Csa1G574970	1.827	1.18E-05	Major CHO metabolism	Hexokinase 2 (HXK2)	7.00E-51
T2-L2	blue	Csa4G618520	1.837	0.0001765	Biodegradation of xenobiotic	UDP glycosyltransferase (UGT)	1.00E-132
T2-L2	blue	Csa2G003610	1.841	0.0003098	Lipid metabolism	Abnormal inflorescence meristem (AIM1)	3.00E-294
T2-L2	blue	Csa3G730930	1.847	0.00004243	Transport	Ammonium transporter 2 (AMT2)	8E-220
T2-L2	blue	Csa6G493850	1.86	1.50E-05	Not assigned	unknown	
T2-L2	blue	Csa3G159420	1.871	1.61E-03	Not assigned	unknown	
T2-L2	blue	Csa1G528610	1.874	0.00435	Lipid metabolism	Thylakoid membrane anion transporter 1 (ANTR1)	7.00E-236
T2-L2	blue	Csa1G181360	1.875	0.001947	Transport	non-intrinsic ABC protein 9 (NAP9)	3.00E-156
T2-L2	blue	Csa3G154410	2.05	1.63E-10	Not assigned	unknown	
T2-L2	blue	Csa3G183950	2.092	0.0004285	Abiotic stress	Heat shock protein 83	0.00E+00
T2-L2	blue	Csa2G296090	2.143	2.44E-04	Not assigned	unknown	
T2-L2	blue	Csa7G291720	2.169	0.006566	Lipid metabolism	Choline kinase	4.00E-133
T2-L2	blue	Csa2G427310	2.238	0.007581	Transcription factor	MYB domain protein 73 (MYB73)	1.00E-88
T2-L2	blue	Csa3G881750	2.24	0.009561	Lipid metabolism	PR5-like receptor kinase (PR5K)	4.00E-154
T2-L2	blue	Csa5G589260	2.243	0	Amino acid metabolism	O-acetylserine(thiol) lyase (OAS-TL)	4.00E-162
T2-L2	blue	Csa3G733350	2.317	0.00004435	Cell organization	RHO guanyl-nucleotide exchange factor 11 (ROPGEF11)	9.00E-41
T2-L2	blue	Csa2G004690	2.522	0.000004593	Transcription factor	Heat shock transcription factor C1 (HSFC1)	5.00E-67
T2-L2	blue	Csa7G387690	2.631	0.000001654	Transport	Sulfate transporter 3;1 (SULTR 3;1)	3.00E-307
T2-L2	blue	Csa1G008520	2.771	0.00804	Transport	Plasma membrane proton ATPase 4 (HA4)	0.00E+00
T2-L2	blue	Csa6G514850	3.048	0.00009803	Miscellaneous	Cytochrome P450 family 77 subfamily B polypeptide 1 (CYP77B1)	1.00E-241
T2-L2	blue	Csa5G604150	3.14	0.01	Redox	Thioredoxin 2 (TRX2)	
T2-L2	blue	Csa5G223070	3.216	0.003151	Transcription factor	WRKY DNA-binding protein 54 (WRKY54)	3.00E-96
T2-L2	blue	Csa2G359890	3.289	3.70E-03	Not assigned	unknown	
T2-L2	blue	Csa1G596520	3.4	0.0002165	Protein degradation	Cystein proteinases superfamily protein	2.00E-168
T2-L2	blue	Csa7G395800	3.415	0.001823	Miscellaneous	Glutathione S-transferase phi 8 (GSTF8)	3.00E-82
T2-L2	blue	Csa3G119700	3.575	0.00005376	Transcription factor	WRKY DNA-binding protein 53 (WRKY53)	1.00E-102
T2-L2	blue	Csa6G381850	3.831	0.001156	Transport	Amino acid permease 3 (AAP3)	2.00E-234
T2-L2	blue	Csa1G033160	4.495	0.006049	Miscellaneous	Glutathione S-transferase tau 9 (GSTU9)	3.00E-68
T2-L2	blue	Csa6G486960	4.496	0.000002502	Transcription factor	WRKY DNA-binding protein 51 (WRKY51)	1.00E-36
T2-L2	blue	Csa2G058620	5.345	0.003945	Abiotic stress	DNAJ heat shock N-terminal domain-containing protein	2.00E-137

Supplementary Table 1 (Continued)

T2-L2	blue	Csa1G574970	1.827	1.18E-05	Major CHO metabolism	Hexokinase 2 (HXK2)	7.00E-51
T2-L2	blue	Csa4G618520	1.837	0.0001765	Biodegradation of xenobiotic	UDP glycosyltransferase (UGT)	1.00E-132
T2-L2	blue	Csa2G003610	1.841	0.0003098	Lipid metabolism	Abnormal inflorescence meristem (AIM1)	3.00E-294
T2-L2	blue	Csa3G730930	1.847	0.00004243	Transport	Ammonium transporter 2 (AMT2)	8E-220
T2-L2	blue	Csa6G493850	1.86	1.50E-05	Not assigned	unknown	
T2-L2	blue	Csa3G159420	1.871	1.61E-03	Not assigned	unknown	
T2-L2	blue	Csa1G528610	1.874	0.00435	Lipid metabolism	Thylakoid membrane anion transporter 1 (ANTR1)	7.00E-236
T2-L2	blue	Csa1G181360	1.875	0.001947	Transport	non-intrinsic ABC protein 9 (NAP9)	3.00E-156
T2-L2	blue	Csa3G154410	2.05	1.63E-10	Not assigned	unknown	
T2-L2	blue	Csa3G183950	2.092	0.0004285	Abiotic stress	Heat shock protein 83	0.00E+00
T2-L2	blue	Csa2G296090	2.143	2.44E-04	Not assigned	unknown	
T2-L2	blue	Csa7G291720	2.169	0.006566	Lipid metabolism	Choline kinase	4.00E-133
T2-L2	blue	Csa2G427310	2.238	0.007581	Transcription factor	MYB domain protein 73 (MYB73)	1.00E-88
T2-L2	blue	Csa3G881750	2.24	0.009561	Lipid metabolism	PR5-like receptor kinase (PR5K)	4.00E-154
T2-L2	blue	Csa5G589260	2.243	0	Amino acid metabolism	O-acetylserine(thiol) lyase (OAS-TL)	4.00E-162
T2-L2	blue	Csa3G733350	2.317	0.00004435	Cell organization	RHO guanyl-nucleotide exchange factor 11 (ROPGEF11)	9.00E-41
T2-L2	blue	Csa2G004690	2.522	0.000004593	Transcription factor	Heat shock transcription factor C1 (HSFC1)	5.00E-67
T2-L2	blue	Csa7G387690	2.631	0.000001654	Transport	Sulfate transporter 3;1 (SULTR 3;1)	3.00E-307
T2-L2	blue	Csa1G008520	2.771	0.00804	Transport	Plasma membrane proton ATPase 4 (HA4)	0.00E+00
T2-L2	blue	Csa6G514850	3.048	0.00009803	Miscellaneous	Cytochrome P450 family 77 subfamily B polypeptide 1 (CYP77B1)	1.00E-241
T2-L2	blue	Csa5G604150	3.14	0.01	Redox	Thioredoxin 2 (TRX2)	
T2-L2	blue	Csa5G223070	3.216	0.003151	Transcription factor	WRKY DNA-binding protein 54 (WRKY54)	3.00E-96
T2-L2	blue	Csa2G359890	3.289	3.70E-03	Not assigned	unknown	
T2-L2	blue	Csa1G596520	3.4	0.0002165	Protein degradation	Cystein proteinases superfamily protein	2.00E-168
T2-L2	blue	Csa7G395800	3.415	0.001823	Miscellaneous	Glutathione S-transferase phi 8 (GSTF8)	3.00E-82
T2-L2	blue	Csa3G119700	3.575	0.00005376	Transcription factor	WRKY DNA-binding protein 53 (WRKY53)	1.00E-102
T2-L2	blue	Csa6G381850	3.831	0.001156	Transport	Amino acid permease 3 (AAP3)	2.00E-234
T2-L2	blue	Csa1G033160	4.495	0.006049	Miscellaneous	Glutathione S-transferase tau 9 (GSTU9)	3.00E-68
T2-L2	blue	Csa6G486960	4.496	0.000002502	Transcription factor	WRKY DNA-binding protein 51 (WRKY51)	1.00E-36
T2-L2	blue	Csa2G058620	5.345	0.003945	Abiotic stress	DNAJ heat shock N-terminal domain-containing protein	2.00E-137

Supplementary Table 1 (Continued)

T2-L2	blue	Csa1G574970	1.827	1.18E-05	Major CHO metabolism	Hexokinase 2 (HXK2)	7.00E-51
T2-L2	blue	Csa4G618520	1.837	0.0001765	Biodegradation of xenobiotic	UDP glycosyltransferase (UGT)	1.00E-132
T2-L2	blue	Csa2G003610	1.841	0.0003098	Lipid metabolism	Abnormal inflorescence meristem (AIM1)	3.00E-294
T2-L2	blue	Csa3G730930	1.847	0.00004243	Transport	Ammonium transporter 2 (AMT2)	8E-220
T2-L2	blue	Csa6G493850	1.86	1.50E-05	Not assigned	unknown	
T2-L2	blue	Csa3G159420	1.871	1.61E-03	Not assigned	unknown	
T2-L2	blue	Csa1G528610	1.874	0.00435	Lipid metabolism	Thylakoid membrane anion transporter 1 (ANTR1)	7.00E-236
T2-L2	blue	Csa1G181360	1.875	0.001947	Transport	non-intrinsic ABC protein 9 (NAP9)	3.00E-156
T2-L2	blue	Csa3G154410	2.05	1.63E-10	Not assigned	unknown	
T2-L2	blue	Csa3G183950	2.092	0.0004285	Abiotic stress	Heat shock protein 83	0.00E+00
T2-L2	blue	Csa2G296090	2.143	2.44E-04	Not assigned	unknown	
T2-L2	blue	Csa7G291720	2.169	0.006566	Lipid metabolism	Choline kinase	4.00E-133
T2-L2	blue	Csa2G427310	2.238	0.007581	Transcription factor	MYB domain protein 73 (MYB73)	1.00E-88
T2-L2	blue	Csa3G881750	2.24	0.009561	Lipid metabolism	PR5-like receptor kinase (PR5K)	4.00E-154
T2-L2	blue	Csa5G589260	2.243	0	Amino acid metabolism	O-acetylserine(thiol) lyase (OAS-TL)	4.00E-162
T2-L2	blue	Csa3G733350	2.317	0.00004435	Cell organization	RHO guanyl-nucleotide exchange factor 11 (ROPGEF11)	9.00E-41
T2-L2	blue	Csa2G004690	2.522	0.000004593	Transcription factor	Heat shock transcription factor C1 (HSFC1)	5.00E-67
T2-L2	blue	Csa7G387690	2.631	0.000001654	Transport	Sulfate transporter 3;1 (SULTR 3;1)	3.00E-307
T2-L2	blue	Csa1G008520	2.771	0.00804	Transport	Plasma membrane proton ATPase 4 (HA4)	0.00E+00
T2-L2	blue	Csa6G514850	3.048	0.00009803	Miscellaneous	Cytochrome P450 family 77 subfamily B polypeptide 1 (CYP77B1)	1.00E-241
T2-L2	blue	Csa5G604150	3.14	0.01	Redox	Thioredoxin 2 (TRX2)	
T2-L2	blue	Csa5G223070	3.216	0.003151	Transcription factor	WRKY DNA-binding protein 54 (WRKY54)	3.00E-96
T2-L2	blue	Csa2G359890	3.289	3.70E-03	Not assigned	unknown	
T2-L2	blue	Csa1G596520	3.4	0.0002165	Protein degradation	Cystein proteinases superfamily protein	2.00E-168
T2-L2	blue	Csa7G395800	3.415	0.001823	Miscellaneous	Glutathione S-transferase phi 8 (GSTF8)	3.00E-82
T2-L2	blue	Csa3G119700	3.575	0.00005376	Transcription factor	WRKY DNA-binding protein 53 (WRKY53)	1.00E-102
T2-L2	blue	Csa6G381850	3.831	0.001156	Transport	Amino acid permease 3 (AAP3)	2.00E-234
T2-L2	blue	Csa1G033160	4.495	0.006049	Miscellaneous	Glutathione S-transferase tau 9 (GSTU9)	3.00E-68
T2-L2	blue	Csa6G486960	4.496	0.000002502	Transcription factor	WRKY DNA-binding protein 51 (WRKY51)	1.00E-36
T2-L2	blue	Csa2G058620	5.345	0.003945	Abiotic stress	DNAJ heat shock N-terminal domain-containing protein	2.00E-137

Supplementary Table 1 (Continued)

T2-L2	blue	Csa1G574970	1.827	1.18E-05	Major CHO metabolism	Hexokinase 2 (HXK2)	7.00E-51
T2-L2	blue	Csa4G618520	1.837	0.0001765	Biodegradation of xenobiotic	UDP glycosyltransferase (UGT)	1.00E-132
T2-L2	blue	Csa2G003610	1.841	0.0003098	Lipid metabolism	Abnormal inflorescence meristem (AIM1)	3.00E-294
T2-L2	blue	Csa3G730930	1.847	0.00004243	Transport	Ammonium transporter 2 (AMT2)	8E-220
T2-L2	blue	Csa6G493850	1.86	1.50E-05	Not assigned	unknown	
T2-L2	blue	Csa3G159420	1.871	1.61E-03	Not assigned	unknown	
T2-L2	blue	Csa1G528610	1.874	0.00435	Lipid metabolism	Thylakoid membrane anion transporter 1 (ANTR1)	7.00E-236
T2-L2	blue	Csa1G181360	1.875	0.001947	Transport	non-intrinsic ABC protein 9 (NAP9)	3.00E-156
T2-L2	blue	Csa3G154410	2.05	1.63E-10	Not assigned	unknown	
T2-L2	blue	Csa3G183950	2.092	0.0004285	Abiotic stress	Heat shock protein 83	0.00E+00
T2-L2	blue	Csa2G296090	2.143	2.44E-04	Not assigned	unknown	
T2-L2	blue	Csa7G291720	2.169	0.006566	Lipid metabolism	Choline kinase	4.00E-133
T2-L2	blue	Csa2G427310	2.238	0.007581	Transcription factor	MYB domain protein 73 (MYB73)	1.00E-88
T2-L2	blue	Csa3G881750	2.24	0.009561	Lipid metabolism	PR5-like receptor kinase (PR5K)	4.00E-154
T2-L2	blue	Csa5G589260	2.243	0	Amino acid metabolism	O-acetylserine(thiol) lyase (OAS-TL)	4.00E-162
T2-L2	blue	Csa3G733350	2.317	0.00004435	Cell organization	RHO guanyl-nucleotide exchange factor 11 (ROPGEF11)	9.00E-41
T2-L2	blue	Csa2G004690	2.522	0.000004593	Transcription factor	Heat shock transcription factor C1 (HSFC1)	5.00E-67
T2-L2	blue	Csa7G387690	2.631	0.000001654	Transport	Sulfate transporter 3;1 (SULTR 3;1)	3.00E-307
T2-L2	blue	Csa1G008520	2.771	0.00804	Transport	Plasma membrane proton ATPase 4 (HA4)	0.00E+00
T2-L2	blue	Csa6G514850	3.048	0.00009803	Miscellaneous	Cytochrome P450 family 77 subfamily B polypeptide 1 (CYP77B1)	1.00E-241
T2-L2	blue	Csa5G604150	3.14	0.01	Redox	Thioredoxin 2 (TRX2)	
T2-L2	blue	Csa5G223070	3.216	0.003151	Transcription factor	WRKY DNA-binding protein 54 (WRKY54)	3.00E-96
T2-L2	blue	Csa2G359890	3.289	3.70E-03	Not assigned	unknown	
T2-L2	blue	Csa1G596520	3.4	0.0002165	Protein degradation	Cystein proteinases superfamily protein	2.00E-168
T2-L2	blue	Csa7G395800	3.415	0.001823	Miscellaneous	Glutathione S-transferase phi 8 (GSTF8)	3.00E-82
T2-L2	blue	Csa3G119700	3.575	0.00005376	Transcription factor	WRKY DNA-binding protein 53 (WRKY53)	1.00E-102
T2-L2	blue	Csa6G381850	3.831	0.001156	Transport	Amino acid permease 3 (AAP3)	2.00E-234
T2-L2	blue	Csa1G033160	4.495	0.006049	Miscellaneous	Glutathione S-transferase tau 9 (GSTU9)	3.00E-68
T2-L2	blue	Csa6G486960	4.496	0.000002502	Transcription factor	WRKY DNA-binding protein 51 (WRKY51)	1.00E-36
T2-L2	blue	Csa2G058620	5.345	0.003945	Abiotic stress	DNAJ heat shock N-terminal domain-containing protein	2.00E-137

Supplementary Table 1 (Continued)

T2-L2	blue	Csa1G574970	1.827	1.18E-05	Major CHO metabolism	Hexokinase 2 (HXK2)	7.00E-51
T2-L2	blue	Csa4G618520	1.837	0.0001765	Biodegradation of xenobiotic	UDP glycosyltransferase (UGT)	1.00E-132
T2-L2	blue	Csa2G003610	1.841	0.0003098	Lipid metabolism	Abnormal inflorescence meristem (AIM1)	3.00E-294
T2-L2	blue	Csa3G730930	1.847	0.00004243	Transport	Ammonium transporter 2 (AMT2)	8E-220
T2-L2	blue	Csa6G493850	1.86	1.50E-05	Not assigned	unknown	
T2-L2	blue	Csa3G159420	1.871	1.61E-03	Not assigned	unknown	
T2-L2	blue	Csa1G528610	1.874	0.00435	Lipid metabolism	Thylakoid membrane anion transporter 1 (ANTR1)	7.00E-236
T2-L2	blue	Csa1G181360	1.875	0.001947	Transport	non-intrinsic ABC protein 9 (NAP9)	3.00E-156
T2-L2	blue	Csa3G154410	2.05	1.63E-10	Not assigned	unknown	
T2-L2	blue	Csa3G183950	2.092	0.0004285	Abiotic stress	Heat shock protein 83	0.00E+00
T2-L2	blue	Csa2G296090	2.143	2.44E-04	Not assigned	unknown	
T2-L2	blue	Csa7G291720	2.169	0.006566	Lipid metabolism	Choline kinase	4.00E-133
T2-L2	blue	Csa2G427310	2.238	0.007581	Transcription factor	MYB domain protein 73 (MYB73)	1.00E-88
T2-L2	blue	Csa3G881750	2.24	0.009561	Lipid metabolism	PR5-like receptor kinase (PR5K)	4.00E-154
T2-L2	blue	Csa5G589260	2.243	0	Amino acid metabolism	O-acetylserine(thiol) lyase (OAS-TL)	4.00E-162
T2-L2	blue	Csa3G733350	2.317	0.00004435	Cell organization	RHO guanyl-nucleotide exchange factor 11 (ROPGEF11)	9.00E-41
T2-L2	blue	Csa2G004690	2.522	0.000004593	Transcription factor	Heat shock transcription factor C1 (HSFC1)	5.00E-67
T2-L2	blue	Csa7G387690	2.631	0.000001654	Transport	Sulfate transporter 3;1 (SULTR 3;1)	3.00E-307
T2-L2	blue	Csa1G008520	2.771	0.00804	Transport	Plasma membrane proton ATPase 4 (HA4)	0.00E+00
T2-L2	blue	Csa6G514850	3.048	0.00009803	Miscellaneous	Cytochrome P450 family 77 subfamily B polypeptide 1 (CYP77B1)	1.00E-241
T2-L2	blue	Csa5G604150	3.14	0.01	Redox	Thioredoxin 2 (TRX2)	
T2-L2	blue	Csa5G223070	3.216	0.003151	Transcription factor	WRKY DNA-binding protein 54 (WRKY54)	3.00E-96
T2-L2	blue	Csa2G359890	3.289	3.70E-03	Not assigned	unknown	
T2-L2	blue	Csa1G596520	3.4	0.0002165	Protein degradation	Cystein proteinases superfamily protein	2.00E-168
T2-L2	blue	Csa7G395800	3.415	0.001823	Miscellaneous	Glutathione S-transferase phi 8 (GSTF8)	3.00E-82
T2-L2	blue	Csa3G119700	3.575	0.00005376	Transcription factor	WRKY DNA-binding protein 53 (WRKY53)	1.00E-102
T2-L2	blue	Csa6G381850	3.831	0.001156	Transport	Amino acid permease 3 (AAP3)	2.00E-234
T2-L2	blue	Csa1G033160	4.495	0.006049	Miscellaneous	Glutathione S-transferase tau 9 (GSTU9)	3.00E-68
T2-L2	blue	Csa6G486960	4.496	0.000002502	Transcription factor	WRKY DNA-binding protein 51 (WRKY51)	1.00E-36
T2-L2	blue	Csa2G058620	5.345	0.003945	Abiotic stress	DNAJ heat shock N-terminal domain-containing protein	2.00E-137

Supplementary Table 1 (Continued)

T2-L2	blue	Csa1G574970	1.827	1.18E-05	Major CHO metabolism	Hexokinase 2 (HXK2)	7.00E-51
T2-L2	blue	Csa4G618520	1.837	0.0001765	Biodegradation of xenobiotic	UDP glycosyltransferase (UGT)	1.00E-132
T2-L2	blue	Csa2G003610	1.841	0.0003098	Lipid metabolism	Abnormal inflorescence meristem (AIM1)	3.00E-294
T2-L2	blue	Csa3G730930	1.847	0.00004243	Transport	Ammonium transporter 2 (AMT2)	8E-220
T2-L2	blue	Csa6G493850	1.86	1.50E-05	Not assigned	unknown	
T2-L2	blue	Csa3G159420	1.871	1.61E-03	Not assigned	unknown	
T2-L2	blue	Csa1G528610	1.874	0.00435	Lipid metabolism	Thylakoid membrane anion transporter 1 (ANTR1)	7.00E-236
T2-L2	blue	Csa1G181360	1.875	0.001947	Transport	non-intrinsic ABC protein 9 (NAP9)	3.00E-156
T2-L2	blue	Csa3G154410	2.05	1.63E-10	Not assigned	unknown	
T2-L2	blue	Csa3G183950	2.092	0.0004285	Abiotic stress	Heat shock protein 83	0.00E+00
T2-L2	blue	Csa2G296090	2.143	2.44E-04	Not assigned	unknown	
T2-L2	blue	Csa7G291720	2.169	0.006566	Lipid metabolism	Choline kinase	4.00E-133
T2-L2	blue	Csa2G427310	2.238	0.007581	Transcription factor	MYB domain protein 73 (MYB73)	1.00E-88
T2-L2	blue	Csa3G881750	2.24	0.009561	Lipid metabolism	PR5-like receptor kinase (PR5K)	4.00E-154
T2-L2	blue	Csa5G589260	2.243	0	Amino acid metabolism	O-acetylserine(thiol) lyase (OAS-TL)	4.00E-162
T2-L2	blue	Csa3G733350	2.317	0.00004435	Cell organization	RHO guanyl-nucleotide exchange factor 11 (ROPGEF11)	9.00E-41
T2-L2	blue	Csa2G004690	2.522	0.000004593	Transcription factor	Heat shock transcription factor C1 (HSFC1)	5.00E-67
T2-L2	blue	Csa7G387690	2.631	0.000001654	Transport	Sulfate transporter 3;1 (SULTR 3;1)	3.00E-307
T2-L2	blue	Csa1G008520	2.771	0.00804	Transport	Plasma membrane proton ATPase 4 (HA4)	0.00E+00
T2-L2	blue	Csa6G514850	3.048	0.00009803	Miscellaneous	Cytochrome P450 family 77 subfamily B polypeptide 1 (CYP77B1)	1.00E-241
T2-L2	blue	Csa5G604150	3.14	0.01	Redox	Thioredoxin 2 (TRX2)	
T2-L2	blue	Csa5G223070	3.216	0.003151	Transcription factor	WRKY DNA-binding protein 54 (WRKY54)	3.00E-96
T2-L2	blue	Csa2G359890	3.289	3.70E-03	Not assigned	unknown	
T2-L2	blue	Csa1G596520	3.4	0.0002165	Protein degradation	Cystein proteinases superfamily protein	2.00E-168
T2-L2	blue	Csa7G395800	3.415	0.001823	Miscellaneous	Glutathione S-transferase phi 8 (GSTF8)	3.00E-82
T2-L2	blue	Csa3G119700	3.575	0.00005376	Transcription factor	WRKY DNA-binding protein 53 (WRKY53)	1.00E-102
T2-L2	blue	Csa6G381850	3.831	0.001156	Transport	Amino acid permease 3 (AAP3)	2.00E-234
T2-L2	blue	Csa1G033160	4.495	0.006049	Miscellaneous	Glutathione S-transferase tau 9 (GSTU9)	3.00E-68
T2-L2	blue	Csa6G486960	4.496	0.000002502	Transcription factor	WRKY DNA-binding protein 51 (WRKY51)	1.00E-36
T2-L2	blue	Csa2G058620	5.345	0.003945	Abiotic stress	DNAJ heat shock N-terminal domain-containing protein	2.00E-137

Supplementary Table 1 (Continued)

T2-L2	blue	Csa1G574970	1.827	1.18E-05	Major CHO metabolism	Hexokinase 2 (HXK2)	7.00E-51
T2-L2	blue	Csa4G618520	1.837	0.0001765	Biodegradation of xenobiotic	UDP glycosyltransferase (UGT)	1.00E-132
T2-L2	blue	Csa2G003610	1.841	0.0003098	Lipid metabolism	Abnormal inflorescence meristem (AIM1)	3.00E-294
T2-L2	blue	Csa3G730930	1.847	0.00004243	Transport	Ammonium transporter 2 (AMT2)	8E-220
T2-L2	blue	Csa6G493850	1.86	1.50E-05	Not assigned	unknown	
T2-L2	blue	Csa3G159420	1.871	1.61E-03	Not assigned	unknown	
T2-L2	blue	Csa1G528610	1.874	0.00435	Lipid metabolism	Thylakoid membrane anion transporter 1 (ANTR1)	7.00E-236
T2-L2	blue	Csa1G181360	1.875	0.001947	Transport	non-intrinsic ABC protein 9 (NAP9)	3.00E-156
T2-L2	blue	Csa3G154410	2.05	1.63E-10	Not assigned	unknown	
T2-L2	blue	Csa3G183950	2.092	0.0004285	Abiotic stress	Heat shock protein 83	0.00E+00
T2-L2	blue	Csa2G296090	2.143	2.44E-04	Not assigned	unknown	
T2-L2	blue	Csa7G291720	2.169	0.006566	Lipid metabolism	Choline kinase	4.00E-133
T2-L2	blue	Csa2G427310	2.238	0.007581	Transcription factor	MYB domain protein 73 (MYB73)	1.00E-88
T2-L2	blue	Csa3G881750	2.24	0.009561	Lipid metabolism	PR5-like receptor kinase (PR5K)	4.00E-154
T2-L2	blue	Csa5G589260	2.243	0	Amino acid metabolism	O-acetylserine(thiol) lyase (OAS-TL)	4.00E-162
T2-L2	blue	Csa3G733350	2.317	0.00004435	Cell organization	RHO guanyl-nucleotide exchange factor 11 (ROPGEF11)	9.00E-41
T2-L2	blue	Csa2G004690	2.522	0.000004593	Transcription factor	Heat shock transcription factor C1 (HSFC1)	5.00E-67
T2-L2	blue	Csa7G387690	2.631	0.000001654	Transport	Sulfate transporter 3;1 (SULTR 3;1)	3.00E-307
T2-L2	blue	Csa1G008520	2.771	0.00804	Transport	Plasma membrane proton ATPase 4 (HA4)	0.00E+00
T2-L2	blue	Csa6G514850	3.048	0.00009803	Miscellaneous	Cytochrome P450 family 77 subfamily B polypeptide 1 (CYP77B1)	1.00E-241
T2-L2	blue	Csa5G604150	3.14	0.01	Redox	Thioredoxin 2 (TRX2)	
T2-L2	blue	Csa5G223070	3.216	0.003151	Transcription factor	WRKY DNA-binding protein 54 (WRKY54)	3.00E-96
T2-L2	blue	Csa2G359890	3.289	3.70E-03	Not assigned	unknown	
T2-L2	blue	Csa1G596520	3.4	0.0002165	Protein degradation	Cystein proteinases superfamily protein	2.00E-168
T2-L2	blue	Csa7G395800	3.415	0.001823	Miscellaneous	Glutathione S-transferase phi 8 (GSTF8)	3.00E-82
T2-L2	blue	Csa3G119700	3.575	0.00005376	Transcription factor	WRKY DNA-binding protein 53 (WRKY53)	1.00E-102
T2-L2	blue	Csa6G381850	3.831	0.001156	Transport	Amino acid permease 3 (AAP3)	2.00E-234
T2-L2	blue	Csa1G033160	4.495	0.006049	Miscellaneous	Glutathione S-transferase tau 9 (GSTU9)	3.00E-68
T2-L2	blue	Csa6G486960	4.496	0.000002502	Transcription factor	WRKY DNA-binding protein 51 (WRKY51)	1.00E-36
T2-L2	blue	Csa2G058620	5.345	0.003945	Abiotic stress	DNAJ heat shock N-terminal domain-containing protein	2.00E-137

Supplementary Table 1 (Continued)

T2-L2	blue	Csa1G574970	1.827	1.18E-05	Major CHO metabolism	Hexokinase 2 (HXK2)	7.00E-51
T2-L2	blue	Csa4G618520	1.837	0.0001765	Biodegradation of xenobiotic	UDP glycosyltransferase (UGT)	1.00E-132
T2-L2	blue	Csa2G003610	1.841	0.0003098	Lipid metabolism	Abnormal inflorescence meristem (AIM1)	3.00E-294
T2-L2	blue	Csa3G730930	1.847	0.00004243	Transport	Ammonium transporter 2 (AMT2)	8E-220
T2-L2	blue	Csa6G493850	1.86	1.50E-05	Not assigned	unknown	
T2-L2	blue	Csa3G159420	1.871	1.61E-03	Not assigned	unknown	
T2-L2	blue	Csa1G528610	1.874	0.00435	Lipid metabolism	Thylakoid membrane anion transporter 1 (ANTR1)	7.00E-236
T2-L2	blue	Csa1G181360	1.875	0.001947	Transport	non-intrinsic ABC protein 9 (NAP9)	3.00E-156
T2-L2	blue	Csa3G154410	2.05	1.63E-10	Not assigned	unknown	
T2-L2	blue	Csa3G183950	2.092	0.0004285	Abiotic stress	Heat shock protein 83	0.00E+00
T2-L2	blue	Csa2G296090	2.143	2.44E-04	Not assigned	unknown	
T2-L2	blue	Csa7G291720	2.169	0.006566	Lipid metabolism	Choline kinase	4.00E-133
T2-L2	blue	Csa2G427310	2.238	0.007581	Transcription factor	MYB domain protein 73 (MYB73)	1.00E-88
T2-L2	blue	Csa3G881750	2.24	0.009561	Lipid metabolism	PR5-like receptor kinase (PR5K)	4.00E-154
T2-L2	blue	Csa5G589260	2.243	0	Amino acid metabolism	O-acetylserine(thiol) lyase (OAS-TL)	4.00E-162
T2-L2	blue	Csa3G733350	2.317	0.00004435	Cell organization	RHO guanyl-nucleotide exchange factor 11 (ROPGEF11)	9.00E-41
T2-L2	blue	Csa2G004690	2.522	0.000004593	Transcription factor	Heat shock transcription factor C1 (HSFC1)	5.00E-67
T2-L2	blue	Csa7G387690	2.631	0.000001654	Transport	Sulfate transporter 3;1 (SULTR 3;1)	3.00E-307
T2-L2	blue	Csa1G008520	2.771	0.00804	Transport	Plasma membrane proton ATPase 4 (HA4)	0.00E+00
T2-L2	blue	Csa6G514850	3.048	0.00009803	Miscellaneous	Cytochrome P450 family 77 subfamily B polypeptide 1 (CYP77B1)	1.00E-241
T2-L2	blue	Csa5G604150	3.14	0.01	Redox	Thioredoxin 2 (TRX2)	
T2-L2	blue	Csa5G223070	3.216	0.003151	Transcription factor	WRKY DNA-binding protein 54 (WRKY54)	3.00E-96
T2-L2	blue	Csa2G359890	3.289	3.70E-03	Not assigned	unknown	
T2-L2	blue	Csa1G596520	3.4	0.0002165	Protein degradation	Cystein proteinases superfamily protein	2.00E-168
T2-L2	blue	Csa7G395800	3.415	0.001823	Miscellaneous	Glutathione S-transferase phi 8 (GSTF8)	3.00E-82
T2-L2	blue	Csa3G119700	3.575	0.00005376	Transcription factor	WRKY DNA-binding protein 53 (WRKY53)	1.00E-102
T2-L2	blue	Csa6G381850	3.831	0.001156	Transport	Amino acid permease 3 (AAP3)	2.00E-234
T2-L2	blue	Csa1G033160	4.495	0.006049	Miscellaneous	Glutathione S-transferase tau 9 (GSTU9)	3.00E-68
T2-L2	blue	Csa6G486960	4.496	0.000002502	Transcription factor	WRKY DNA-binding protein 51 (WRKY51)	1.00E-36
T2-L2	blue	Csa2G058620	5.345	0.003945	Abiotic stress	DNAJ heat shock N-terminal domain-containing protein	2.00E-137

Supplementary Table 1 (Continued)

T2-L2	blue	Csa1G574970	1.827	1.18E-05	Major CHO metabolism	Hexokinase 2 (HXK2)	7.00E-51
T2-L2	blue	Csa4G618520	1.837	0.0001765	Biodegradation of xenobiotic	UDP glycosyltransferase (UGT)	1.00E-132
T2-L2	blue	Csa2G003610	1.841	0.0003098	Lipid metabolism	Abnormal inflorescence meristem (AIM1)	3.00E-294
T2-L2	blue	Csa3G730930	1.847	0.00004243	Transport	Ammonium transporter 2 (AMT2)	8E-220
T2-L2	blue	Csa6G493850	1.86	1.50E-05	Not assigned	unknown	
T2-L2	blue	Csa3G159420	1.871	1.61E-03	Not assigned	unknown	
T2-L2	blue	Csa1G528610	1.874	0.00435	Lipid metabolism	Thylakoid membrane anion transporter 1 (ANTR1)	7.00E-236
T2-L2	blue	Csa1G181360	1.875	0.001947	Transport	non-intrinsic ABC protein 9 (NAP9)	3.00E-156
T2-L2	blue	Csa3G154410	2.05	1.63E-10	Not assigned	unknown	
T2-L2	blue	Csa3G183950	2.092	0.0004285	Abiotic stress	Heat shock protein 83	0.00E+00
T2-L2	blue	Csa2G296090	2.143	2.44E-04	Not assigned	unknown	
T2-L2	blue	Csa7G291720	2.169	0.006566	Lipid metabolism	Choline kinase	4.00E-133
T2-L2	blue	Csa2G427310	2.238	0.007581	Transcription factor	MYB domain protein 73 (MYB73)	1.00E-88
T2-L2	blue	Csa3G881750	2.24	0.009561	Lipid metabolism	PR5-like receptor kinase (PR5K)	4.00E-154
T2-L2	blue	Csa5G589260	2.243	0	Amino acid metabolism	O-acetylserine(thiol) lyase (OAS-TL)	4.00E-162
T2-L2	blue	Csa3G733350	2.317	0.00004435	Cell organization	RHO guanyl-nucleotide exchange factor 11 (ROPGEF11)	9.00E-41
T2-L2	blue	Csa2G004690	2.522	0.000004593	Transcription factor	Heat shock transcription factor C1 (HSFC1)	5.00E-67
T2-L2	blue	Csa7G387690	2.631	0.000001654	Transport	Sulfate transporter 3;1 (SULTR 3;1)	3.00E-307
T2-L2	blue	Csa1G008520	2.771	0.00804	Transport	Plasma membrane proton ATPase 4 (HA4)	0.00E+00
T2-L2	blue	Csa6G514850	3.048	0.00009803	Miscellaneous	Cytochrome P450 family 77 subfamily B polypeptide 1 (CYP77B1)	1.00E-241
T2-L2	blue	Csa5G604150	3.14	0.01	Redox	Thioredoxin 2 (TRX2)	
T2-L2	blue	Csa5G223070	3.216	0.003151	Transcription factor	WRKY DNA-binding protein 54 (WRKY54)	3.00E-96
T2-L2	blue	Csa2G359890	3.289	3.70E-03	Not assigned	unknown	
T2-L2	blue	Csa1G596520	3.4	0.0002165	Protein degradation	Cystein proteinases superfamily protein	2.00E-168
T2-L2	blue	Csa7G395800	3.415	0.001823	Miscellaneous	Glutathione S-transferase phi 8 (GSTF8)	3.00E-82
T2-L2	blue	Csa3G119700	3.575	0.00005376	Transcription factor	WRKY DNA-binding protein 53 (WRKY53)	1.00E-102
T2-L2	blue	Csa6G381850	3.831	0.001156	Transport	Amino acid permease 3 (AAP3)	2.00E-234
T2-L2	blue	Csa1G033160	4.495	0.006049	Miscellaneous	Glutathione S-transferase tau 9 (GSTU9)	3.00E-68
T2-L2	blue	Csa6G486960	4.496	0.000002502	Transcription factor	WRKY DNA-binding protein 51 (WRKY51)	1.00E-36
T2-L2	blue	Csa2G058620	5.345	0.003945	Abiotic stress	DNAJ heat shock N-terminal domain-containing protein	2.00E-137

Supplementary Table 1 (Continued)

T2-L2	blue	Csa1G574970	1.827	1.18E-05	Major CHO metabolism	Hexokinase 2 (HXK2)	7.00E-51
T2-L2	blue	Csa4G618520	1.837	0.0001765	Biodegradation of xenobiotic	UDP glycosyltransferase (UGT)	1.00E-132
T2-L2	blue	Csa2G003610	1.841	0.0003098	Lipid metabolism	Abnormal inflorescence meristem (AIM1)	3.00E-294
T2-L2	blue	Csa3G730930	1.847	0.00004243	Transport	Ammonium transporter 2 (AMT2)	8E-220
T2-L2	blue	Csa6G493850	1.86	1.50E-05	Not assigned	unknown	
T2-L2	blue	Csa3G159420	1.871	1.61E-03	Not assigned	unknown	
T2-L2	blue	Csa1G528610	1.874	0.00435	Lipid metabolism	Thylakoid membrane anion transporter 1 (ANTR1)	7.00E-236
T2-L2	blue	Csa1G181360	1.875	0.001947	Transport	non-intrinsic ABC protein 9 (NAP9)	3.00E-156
T2-L2	blue	Csa3G154410	2.05	1.63E-10	Not assigned	unknown	
T2-L2	blue	Csa3G183950	2.092	0.0004285	Abiotic stress	Heat shock protein 83	0.00E+00
T2-L2	blue	Csa2G296090	2.143	2.44E-04	Not assigned	unknown	
T2-L2	blue	Csa7G291720	2.169	0.006566	Lipid metabolism	Choline kinase	4.00E-133
T2-L2	blue	Csa2G427310	2.238	0.007581	Transcription factor	MYB domain protein 73 (MYB73)	1.00E-88
T2-L2	blue	Csa3G881750	2.24	0.009561	Lipid metabolism	PR5-like receptor kinase (PR5K)	4.00E-154
T2-L2	blue	Csa5G589260	2.243	0	Amino acid metabolism	O-acetylserine(thiol) lyase (OAS-TL)	4.00E-162
T2-L2	blue	Csa3G733350	2.317	0.00004435	Cell organization	RHO guanyl-nucleotide exchange factor 11 (ROPGEF11)	9.00E-41
T2-L2	blue	Csa2G004690	2.522	0.000004593	Transcription factor	Heat shock transcription factor C1 (HSFC1)	5.00E-67
T2-L2	blue	Csa7G387690	2.631	0.000001654	Transport	Sulfate transporter 3;1 (SULTR 3;1)	3.00E-307
T2-L2	blue	Csa1G008520	2.771	0.00804	Transport	Plasma membrane proton ATPase 4 (HA4)	0.00E+00
T2-L2	blue	Csa6G514850	3.048	0.00009803	Miscellaneous	Cytochrome P450 family 77 subfamily B polypeptide 1 (CYP77B1)	1.00E-241
T2-L2	blue	Csa5G604150	3.14	0.01	Redox	Thioredoxin 2 (TRX2)	
T2-L2	blue	Csa5G223070	3.216	0.003151	Transcription factor	WRKY DNA-binding protein 54 (WRKY54)	3.00E-96
T2-L2	blue	Csa2G359890	3.289	3.70E-03	Not assigned	unknown	
T2-L2	blue	Csa1G596520	3.4	0.0002165	Protein degradation	Cystein proteinases superfamily protein	2.00E-168
T2-L2	blue	Csa7G395800	3.415	0.001823	Miscellaneous	Glutathione S-transferase phi 8 (GSTF8)	3.00E-82
T2-L2	blue	Csa3G119700	3.575	0.00005376	Transcription factor	WRKY DNA-binding protein 53 (WRKY53)	1.00E-102
T2-L2	blue	Csa6G381850	3.831	0.001156	Transport	Amino acid permease 3 (AAP3)	2.00E-234
T2-L2	blue	Csa1G033160	4.495	0.006049	Miscellaneous	Glutathione S-transferase tau 9 (GSTU9)	3.00E-68
T2-L2	blue	Csa6G486960	4.496	0.000002502	Transcription factor	WRKY DNA-binding protein 51 (WRKY51)	1.00E-36
T2-L2	blue	Csa2G058620	5.345	0.003945	Abiotic stress	DNAJ heat shock N-terminal domain-containing protein	2.00E-137

Supplementary Table 1 (Continued)

T2-L2	blue	Csa1G574970	1.827	1.18E-05	Major CHO metabolism	Hexokinase 2 (HXK2)	7.00E-51
T2-L2	blue	Csa4G618520	1.837	0.0001765	Biodegradation of xenobiotic	UDP glycosyltransferase (UGT)	1.00E-132
T2-L2	blue	Csa2G003610	1.841	0.0003098	Lipid metabolism	Abnormal inflorescence meristem (AIM1)	3.00E-294
T2-L2	blue	Csa3G730930	1.847	0.00004243	Transport	Ammonium transporter 2 (AMT2)	8E-220
T2-L2	blue	Csa6G493850	1.86	1.50E-05	Not assigned	unknown	
T2-L2	blue	Csa3G159420	1.871	1.61E-03	Not assigned	unknown	
T2-L2	blue	Csa1G528610	1.874	0.00435	Lipid metabolism	Thylakoid membrane anion transporter 1 (ANTR1)	7.00E-236
T2-L2	blue	Csa1G181360	1.875	0.001947	Transport	non-intrinsic ABC protein 9 (NAP9)	3.00E-156
T2-L2	blue	Csa3G154410	2.05	1.63E-10	Not assigned	unknown	
T2-L2	blue	Csa3G183950	2.092	0.0004285	Abiotic stress	Heat shock protein 83	0.00E+00
T2-L2	blue	Csa2G296090	2.143	2.44E-04	Not assigned	unknown	
T2-L2	blue	Csa7G291720	2.169	0.006566	Lipid metabolism	Choline kinase	4.00E-133
T2-L2	blue	Csa2G427310	2.238	0.007581	Transcription factor	MYB domain protein 73 (MYB73)	1.00E-88
T2-L2	blue	Csa3G881750	2.24	0.009561	Lipid metabolism	PR5-like receptor kinase (PR5K)	4.00E-154
T2-L2	blue	Csa5G589260	2.243	0	Amino acid metabolism	O-acetylserine(thiol) lyase (OAS-TL)	4.00E-162
T2-L2	blue	Csa3G733350	2.317	0.00004435	Cell organization	RHO guanyl-nucleotide exchange factor 11 (ROPGEF11)	9.00E-41
T2-L2	blue	Csa2G004690	2.522	0.000004593	Transcription factor	Heat shock transcription factor C1 (HSFC1)	5.00E-67
T2-L2	blue	Csa7G387690	2.631	0.000001654	Transport	Sulfate transporter 3;1 (SULTR 3;1)	3.00E-307
T2-L2	blue	Csa1G008520	2.771	0.00804	Transport	Plasma membrane proton ATPase 4 (HA4)	0.00E+00
T2-L2	blue	Csa6G514850	3.048	0.00009803	Miscellaneous	Cytochrome P450 family 77 subfamily B polypeptide 1 (CYP77B1)	1.00E-241
T2-L2	blue	Csa5G604150	3.14	0.01	Redox	Thioredoxin 2 (TRX2)	
T2-L2	blue	Csa5G223070	3.216	0.003151	Transcription factor	WRKY DNA-binding protein 54 (WRKY54)	3.00E-96
T2-L2	blue	Csa2G359890	3.289	3.70E-03	Not assigned	unknown	
T2-L2	blue	Csa1G596520	3.4	0.0002165	Protein degradation	Cystein proteinases superfamily protein	2.00E-168
T2-L2	blue	Csa7G395800	3.415	0.001823	Miscellaneous	Glutathione S-transferase phi 8 (GSTF8)	3.00E-82
T2-L2	blue	Csa3G119700	3.575	0.00005376	Transcription factor	WRKY DNA-binding protein 53 (WRKY53)	1.00E-102
T2-L2	blue	Csa6G381850	3.831	0.001156	Transport	Amino acid permease 3 (AAP3)	2.00E-234
T2-L2	blue	Csa1G033160	4.495	0.006049	Miscellaneous	Glutathione S-transferase tau 9 (GSTU9)	3.00E-68
T2-L2	blue	Csa6G486960	4.496	0.000002502	Transcription factor	WRKY DNA-binding protein 51 (WRKY51)	1.00E-36
T2-L2	blue	Csa2G058620	5.345	0.003945	Abiotic stress	DNAJ heat shock N-terminal domain-containing protein	2.00E-137

Supplementary Table 1 (Continued)

T2-L2	blue	Csa1G574970	1.827	1.18E-05	Major CHO metabolism	Hexokinase 2 (HXK2)	7.00E-51
T2-L2	blue	Csa4G618520	1.837	0.0001765	Biodegradation of xenobiotic	UDP glycosyltransferase (UGT)	1.00E-132
T2-L2	blue	Csa2G003610	1.841	0.0003098	Lipid metabolism	Abnormal inflorescence meristem (AIM1)	3.00E-294
T2-L2	blue	Csa3G730930	1.847	0.00004243	Transport	Ammonium transporter 2 (AMT2)	8E-220
T2-L2	blue	Csa6G493850	1.86	1.50E-05	Not assigned	unknown	
T2-L2	blue	Csa3G159420	1.871	1.61E-03	Not assigned	unknown	
T2-L2	blue	Csa1G528610	1.874	0.00435	Lipid metabolism	Thylakoid membrane anion transporter 1 (ANTR1)	7.00E-236
T2-L2	blue	Csa1G181360	1.875	0.001947	Transport	non-intrinsic ABC protein 9 (NAP9)	3.00E-156
T2-L2	blue	Csa3G154410	2.05	1.63E-10	Not assigned	unknown	
T2-L2	blue	Csa3G183950	2.092	0.0004285	Abiotic stress	Heat shock protein 83	0.00E+00
T2-L2	blue	Csa2G296090	2.143	2.44E-04	Not assigned	unknown	
T2-L2	blue	Csa7G291720	2.169	0.006566	Lipid metabolism	Choline kinase	4.00E-133
T2-L2	blue	Csa2G427310	2.238	0.007581	Transcription factor	MYB domain protein 73 (MYB73)	1.00E-88
T2-L2	blue	Csa3G881750	2.24	0.009561	Lipid metabolism	PR5-like receptor kinase (PR5K)	4.00E-154
T2-L2	blue	Csa5G589260	2.243	0	Amino acid metabolism	O-acetylserine(thiol) lyase (OAS-TL)	4.00E-162
T2-L2	blue	Csa3G733350	2.317	0.00004435	Cell organization	RHO guanyl-nucleotide exchange factor 11 (ROPGEF11)	9.00E-41
T2-L2	blue	Csa2G004690	2.522	0.000004593	Transcription factor	Heat shock transcription factor C1 (HSFC1)	5.00E-67
T2-L2	blue	Csa7G387690	2.631	0.000001654	Transport	Sulfate transporter 3;1 (SULTR 3;1)	3.00E-307
T2-L2	blue	Csa1G008520	2.771	0.00804	Transport	Plasma membrane proton ATPase 4 (HA4)	0.00E+00
T2-L2	blue	Csa6G514850	3.048	0.00009803	Miscellaneous	Cytochrome P450 family 77 subfamily B polypeptide 1 (CYP77B1)	1.00E-241
T2-L2	blue	Csa5G604150	3.14	0.01	Redox	Thioredoxin 2 (TRX2)	
T2-L2	blue	Csa5G223070	3.216	0.003151	Transcription factor	WRKY DNA-binding protein 54 (WRKY54)	3.00E-96
T2-L2	blue	Csa2G359890	3.289	3.70E-03	Not assigned	unknown	
T2-L2	blue	Csa1G596520	3.4	0.0002165	Protein degradation	Cystein proteinases superfamily protein	2.00E-168
T2-L2	blue	Csa7G395800	3.415	0.001823	Miscellaneous	Glutathione S-transferase phi 8 (GSTF8)	3.00E-82
T2-L2	blue	Csa3G119700	3.575	0.00005376	Transcription factor	WRKY DNA-binding protein 53 (WRKY53)	1.00E-102
T2-L2	blue	Csa6G381850	3.831	0.001156	Transport	Amino acid permease 3 (AAP3)	2.00E-234
T2-L2	blue	Csa1G033160	4.495	0.006049	Miscellaneous	Glutathione S-transferase tau 9 (GSTU9)	3.00E-68
T2-L2	blue	Csa6G486960	4.496	0.000002502	Transcription factor	WRKY DNA-binding protein 51 (WRKY51)	1.00E-36
T2-L2	blue	Csa2G058620	5.345	0.003945	Abiotic stress	DNAJ heat shock N-terminal domain-containing protein	2.00E-137

Supplementary Table 1 (Continued)

T2-L2	blue	Csa1G574970	1.827	1.18E-05	Major CHO metabolism	Hexokinase 2 (HXK2)	7.00E-51
T2-L2	blue	Csa4G618520	1.837	0.0001765	Biodegradation of xenobiotic	UDP glycosyltransferase (UGT)	1.00E-132
T2-L2	blue	Csa2G003610	1.841	0.0003098	Lipid metabolism	Abnormal inflorescence meristem (AIM1)	3.00E-294
T2-L2	blue	Csa3G730930	1.847	0.00004243	Transport	Ammonium transporter 2 (AMT2)	8E-220
T2-L2	blue	Csa6G493850	1.86	1.50E-05	Not assigned	unknown	
T2-L2	blue	Csa3G159420	1.871	1.61E-03	Not assigned	unknown	
T2-L2	blue	Csa1G528610	1.874	0.00435	Lipid metabolism	Thylakoid membrane anion transporter 1 (ANTR1)	7.00E-236
T2-L2	blue	Csa1G181360	1.875	0.001947	Transport	non-intrinsic ABC protein 9 (NAP9)	3.00E-156
T2-L2	blue	Csa3G154410	2.05	1.63E-10	Not assigned	unknown	
T2-L2	blue	Csa3G183950	2.092	0.0004285	Abiotic stress	Heat shock protein 83	0.00E+00
T2-L2	blue	Csa2G296090	2.143	2.44E-04	Not assigned	unknown	
T2-L2	blue	Csa7G291720	2.169	0.006566	Lipid metabolism	Choline kinase	4.00E-133
T2-L2	blue	Csa2G427310	2.238	0.007581	Transcription factor	MYB domain protein 73 (MYB73)	1.00E-88
T2-L2	blue	Csa3G881750	2.24	0.009561	Lipid metabolism	PR5-like receptor kinase (PR5K)	4.00E-154
T2-L2	blue	Csa5G589260	2.243	0	Amino acid metabolism	O-acetylserine(thiol) lyase (OAS-TL)	4.00E-162
T2-L2	blue	Csa3G733350	2.317	0.00004435	Cell organization	RHO guanyl-nucleotide exchange factor 11 (ROPGEF11)	9.00E-41
T2-L2	blue	Csa2G004690	2.522	0.000004593	Transcription factor	Heat shock transcription factor C1 (HSFC1)	5.00E-67
T2-L2	blue	Csa7G387690	2.631	0.000001654	Transport	Sulfate transporter 3;1 (SULTR 3;1)	3.00E-307
T2-L2	blue	Csa1G008520	2.771	0.00804	Transport	Plasma membrane proton ATPase 4 (HA4)	0.00E+00
T2-L2	blue	Csa6G514850	3.048	0.00009803	Miscellaneous	Cytochrome P450 family 77 subfamily B polypeptide 1 (CYP77B1)	1.00E-241
T2-L2	blue	Csa5G604150	3.14	0.01	Redox	Thioredoxin 2 (TRX2)	
T2-L2	blue	Csa5G223070	3.216	0.003151	Transcription factor	WRKY DNA-binding protein 54 (WRKY54)	3.00E-96
T2-L2	blue	Csa2G359890	3.289	3.70E-03	Not assigned	unknown	
T2-L2	blue	Csa1G596520	3.4	0.0002165	Protein degradation	Cystein proteinases superfamily protein	2.00E-168
T2-L2	blue	Csa7G395800	3.415	0.001823	Miscellaneous	Glutathione S-transferase phi 8 (GSTF8)	3.00E-82
T2-L2	blue	Csa3G119700	3.575	0.00005376	Transcription factor	WRKY DNA-binding protein 53 (WRKY53)	1.00E-102
T2-L2	blue	Csa6G381850	3.831	0.001156	Transport	Amino acid permease 3 (AAP3)	2.00E-234
T2-L2	blue	Csa1G033160	4.495	0.006049	Miscellaneous	Glutathione S-transferase tau 9 (GSTU9)	3.00E-68
T2-L2	blue	Csa6G486960	4.496	0.000002502	Transcription factor	WRKY DNA-binding protein 51 (WRKY51)	1.00E-36
T2-L2	blue	Csa2G058620	5.345	0.003945	Abiotic stress	DNAJ heat shock N-terminal domain-containing protein	2.00E-137

Supplementary Table 1 (Continued)

T2-L2	blue	Csa1G574970	1.827	1.18E-05	Major CHO metabolism	Hexokinase 2 (HXK2)	7.00E-51
T2-L2	blue	Csa4G618520	1.837	0.0001765	Biodegradation of xenobiotic	UDP glycosyltransferase (UGT)	1.00E-132
T2-L2	blue	Csa2G003610	1.841	0.0003098	Lipid metabolism	Abnormal inflorescence meristem (AIM1)	3.00E-294
T2-L2	blue	Csa3G730930	1.847	0.00004243	Transport	Ammonium transporter 2 (AMT2)	8E-220
T2-L2	blue	Csa6G493850	1.86	1.50E-05	Not assigned	unknown	
T2-L2	blue	Csa3G159420	1.871	1.61E-03	Not assigned	unknown	
T2-L2	blue	Csa1G528610	1.874	0.00435	Lipid metabolism	Thylakoid membrane anion transporter 1 (ANTR1)	7.00E-236
T2-L2	blue	Csa1G181360	1.875	0.001947	Transport	non-intrinsic ABC protein 9 (NAP9)	3.00E-156
T2-L2	blue	Csa3G154410	2.05	1.63E-10	Not assigned	unknown	
T2-L2	blue	Csa3G183950	2.092	0.0004285	Abiotic stress	Heat shock protein 83	0.00E+00
T2-L2	blue	Csa2G296090	2.143	2.44E-04	Not assigned	unknown	
T2-L2	blue	Csa7G291720	2.169	0.006566	Lipid metabolism	Choline kinase	4.00E-133
T2-L2	blue	Csa2G427310	2.238	0.007581	Transcription factor	MYB domain protein 73 (MYB73)	1.00E-88
T2-L2	blue	Csa3G881750	2.24	0.009561	Lipid metabolism	PR5-like receptor kinase (PR5K)	4.00E-154
T2-L2	blue	Csa5G589260	2.243	0	Amino acid metabolism	O-acetylserine(thiol) lyase (OAS-TL)	4.00E-162
T2-L2	blue	Csa3G733350	2.317	0.00004435	Cell organization	RHO guanyl-nucleotide exchange factor 11 (ROPGEF11)	9.00E-41
T2-L2	blue	Csa2G004690	2.522	0.000004593	Transcription factor	Heat shock transcription factor C1 (HSFC1)	5.00E-67
T2-L2	blue	Csa7G387690	2.631	0.000001654	Transport	Sulfate transporter 3;1 (SULTR 3;1)	3.00E-307
T2-L2	blue	Csa1G008520	2.771	0.00804	Transport	Plasma membrane proton ATPase 4 (HA4)	0.00E+00
T2-L2	blue	Csa6G514850	3.048	0.00009803	Miscellaneous	Cytochrome P450 family 77 subfamily B polypeptide 1 (CYP77B1)	1.00E-241
T2-L2	blue	Csa5G604150	3.14	0.01	Redox	Thioredoxin 2 (TRX2)	
T2-L2	blue	Csa5G223070	3.216	0.003151	Transcription factor	WRKY DNA-binding protein 54 (WRKY54)	3.00E-96
T2-L2	blue	Csa2G359890	3.289	3.70E-03	Not assigned	unknown	
T2-L2	blue	Csa1G596520	3.4	0.0002165	Protein degradation	Cystein proteinases superfamily protein	2.00E-168
T2-L2	blue	Csa7G395800	3.415	0.001823	Miscellaneous	Glutathione S-transferase phi 8 (GSTF8)	3.00E-82
T2-L2	blue	Csa3G119700	3.575	0.00005376	Transcription factor	WRKY DNA-binding protein 53 (WRKY53)	1.00E-102
T2-L2	blue	Csa6G381850	3.831	0.001156	Transport	Amino acid permease 3 (AAP3)	2.00E-234
T2-L2	blue	Csa1G033160	4.495	0.006049	Miscellaneous	Glutathione S-transferase tau 9 (GSTU9)	3.00E-68
T2-L2	blue	Csa6G486960	4.496	0.000002502	Transcription factor	WRKY DNA-binding protein 51 (WRKY51)	1.00E-36
T2-L2	blue	Csa2G058620	5.345	0.003945	Abiotic stress	DNAJ heat shock N-terminal domain-containing protein	2.00E-137

Supplementary Table 1 (Continued)

T2-L2	blue	Csa1G574970	1.827	1.18E-05	Major CHO metabolism	Hexokinase 2 (HXK2)	7.00E-51
T2-L2	blue	Csa4G618520	1.837	0.0001765	Biodegradation of xenobiotic	UDP glycosyltransferase (UGT)	1.00E-132
T2-L2	blue	Csa2G003610	1.841	0.0003098	Lipid metabolism	Abnormal inflorescence meristem (AIM1)	3.00E-294
T2-L2	blue	Csa3G730930	1.847	0.00004243	Transport	Ammonium transporter 2 (AMT2)	8E-220
T2-L2	blue	Csa6G493850	1.86	1.50E-05	Not assigned	unknown	
T2-L2	blue	Csa3G159420	1.871	1.61E-03	Not assigned	unknown	
T2-L2	blue	Csa1G528610	1.874	0.00435	Lipid metabolism	Thylakoid membrane anion transporter 1 (ANTR1)	7.00E-236
T2-L2	blue	Csa1G181360	1.875	0.001947	Transport	non-intrinsic ABC protein 9 (NAP9)	3.00E-156
T2-L2	blue	Csa3G154410	2.05	1.63E-10	Not assigned	unknown	
T2-L2	blue	Csa3G183950	2.092	0.0004285	Abiotic stress	Heat shock protein 83	0.00E+00
T2-L2	blue	Csa2G296090	2.143	2.44E-04	Not assigned	unknown	
T2-L2	blue	Csa7G291720	2.169	0.006566	Lipid metabolism	Choline kinase	4.00E-133
T2-L2	blue	Csa2G427310	2.238	0.007581	Transcription factor	MYB domain protein 73 (MYB73)	1.00E-88
T2-L2	blue	Csa3G881750	2.24	0.009561	Lipid metabolism	PR5-like receptor kinase (PR5K)	4.00E-154
T2-L2	blue	Csa5G589260	2.243	0	Amino acid metabolism	O-acetylserine(thiol) lyase (OAS-TL)	4.00E-162
T2-L2	blue	Csa3G733350	2.317	0.00004435	Cell organization	RHO guanyl-nucleotide exchange factor 11 (ROPGEF11)	9.00E-41
T2-L2	blue	Csa2G004690	2.522	0.000004593	Transcription factor	Heat shock transcription factor C1 (HSFC1)	5.00E-67
T2-L2	blue	Csa7G387690	2.631	0.000001654	Transport	Sulfate transporter 3;1 (SULTR 3;1)	3.00E-307
T2-L2	blue	Csa1G008520	2.771	0.00804	Transport	Plasma membrane proton ATPase 4 (HA4)	0.00E+00
T2-L2	blue	Csa6G514850	3.048	0.00009803	Miscellaneous	Cytochrome P450 family 77 subfamily B polypeptide 1 (CYP77B1)	1.00E-241
T2-L2	blue	Csa5G604150	3.14	0.01	Redox	Thioredoxin 2 (TRX2)	
T2-L2	blue	Csa5G223070	3.216	0.003151	Transcription factor	WRKY DNA-binding protein 54 (WRKY54)	3.00E-96
T2-L2	blue	Csa2G359890	3.289	3.70E-03	Not assigned	unknown	
T2-L2	blue	Csa1G596520	3.4	0.0002165	Protein degradation	Cystein proteinases superfamily protein	2.00E-168
T2-L2	blue	Csa7G395800	3.415	0.001823	Miscellaneous	Glutathione S-transferase phi 8 (GSTF8)	3.00E-82
T2-L2	blue	Csa3G119700	3.575	0.00005376	Transcription factor	WRKY DNA-binding protein 53 (WRKY53)	1.00E-102
T2-L2	blue	Csa6G381850	3.831	0.001156	Transport	Amino acid permease 3 (AAP3)	2.00E-234
T2-L2	blue	Csa1G033160	4.495	0.006049	Miscellaneous	Glutathione S-transferase tau 9 (GSTU9)	3.00E-68
T2-L2	blue	Csa6G486960	4.496	0.000002502	Transcription factor	WRKY DNA-binding protein 51 (WRKY51)	1.00E-36
T2-L2	blue	Csa2G058620	5.345	0.003945	Abiotic stress	DNAJ heat shock N-terminal domain-containing protein	2.00E-137

Supplementary Table 1 (Continued)

T2-L2	blue	Csa1G574970	1.827	1.18E-05	Major CHO metabolism	Hexokinase 2 (HXK2)	7.00E-51
T2-L2	blue	Csa4G618520	1.837	0.0001765	Biodegradation of xenobiotic	UDP glycosyltransferase (UGT)	1.00E-132
T2-L2	blue	Csa2G003610	1.841	0.0003098	Lipid metabolism	Abnormal inflorescence meristem (AIM1)	3.00E-294
T2-L2	blue	Csa3G730930	1.847	0.00004243	Transport	Ammonium transporter 2 (AMT2)	8E-220
T2-L2	blue	Csa6G493850	1.86	1.50E-05	Not assigned	unknown	
T2-L2	blue	Csa3G159420	1.871	1.61E-03	Not assigned	unknown	
T2-L2	blue	Csa1G528610	1.874	0.00435	Lipid metabolism	Thylakoid membrane anion transporter 1 (ANTR1)	7.00E-236
T2-L2	blue	Csa1G181360	1.875	0.001947	Transport	non-intrinsic ABC protein 9 (NAP9)	3.00E-156
T2-L2	blue	Csa3G154410	2.05	1.63E-10	Not assigned	unknown	
T2-L2	blue	Csa3G183950	2.092	0.0004285	Abiotic stress	Heat shock protein 83	0.00E+00
T2-L2	blue	Csa2G296090	2.143	2.44E-04	Not assigned	unknown	
T2-L2	blue	Csa7G291720	2.169	0.006566	Lipid metabolism	Choline kinase	4.00E-133
T2-L2	blue	Csa2G427310	2.238	0.007581	Transcription factor	MYB domain protein 73 (MYB73)	1.00E-88
T2-L2	blue	Csa3G881750	2.24	0.009561	Lipid metabolism	PR5-like receptor kinase (PR5K)	4.00E-154
T2-L2	blue	Csa5G589260	2.243	0	Amino acid metabolism	O-acetylserine(thiol) lyase (OAS-TL)	4.00E-162
T2-L2	blue	Csa3G733350	2.317	0.00004435	Cell organization	RHO guanyl-nucleotide exchange factor 11 (ROPGEF11)	9.00E-41
T2-L2	blue	Csa2G004690	2.522	0.000004593	Transcription factor	Heat shock transcription factor C1 (HSFC1)	5.00E-67
T2-L2	blue	Csa7G387690	2.631	0.000001654	Transport	Sulfate transporter 3;1 (SULTR 3;1)	3.00E-307
T2-L2	blue	Csa1G008520	2.771	0.00804	Transport	Plasma membrane proton ATPase 4 (HA4)	0.00E+00
T2-L2	blue	Csa6G514850	3.048	0.00009803	Miscellaneous	Cytochrome P450 family 77 subfamily B polypeptide 1 (CYP77B1)	1.00E-241
T2-L2	blue	Csa5G604150	3.14	0.01	Redox	Thioredoxin 2 (TRX2)	
T2-L2	blue	Csa5G223070	3.216	0.003151	Transcription factor	WRKY DNA-binding protein 54 (WRKY54)	3.00E-96
T2-L2	blue	Csa2G359890	3.289	3.70E-03	Not assigned	unknown	
T2-L2	blue	Csa1G596520	3.4	0.0002165	Protein degradation	Cystein proteinases superfamily protein	2.00E-168
T2-L2	blue	Csa7G395800	3.415	0.001823	Miscellaneous	Glutathione S-transferase phi 8 (GSTF8)	3.00E-82
T2-L2	blue	Csa3G119700	3.575	0.00005376	Transcription factor	WRKY DNA-binding protein 53 (WRKY53)	1.00E-102
T2-L2	blue	Csa6G381850	3.831	0.001156	Transport	Amino acid permease 3 (AAP3)	2.00E-234
T2-L2	blue	Csa1G033160	4.495	0.006049	Miscellaneous	Glutathione S-transferase tau 9 (GSTU9)	3.00E-68
T2-L2	blue	Csa6G486960	4.496	0.000002502	Transcription factor	WRKY DNA-binding protein 51 (WRKY51)	1.00E-36
T2-L2	blue	Csa2G058620	5.345	0.003945	Abiotic stress	DNAJ heat shock N-terminal domain-containing protein	2.00E-137

Supplementary Table 1 (Continued)

T2-L2	blue	Csa1G574970	1.827	1.18E-05	Major CHO metabolism	Hexokinase 2 (HXK2)	7.00E-51
T2-L2	blue	Csa4G618520	1.837	0.0001765	Biodegradation of xenobiotic	UDP glycosyltransferase (UGT)	1.00E-132
T2-L2	blue	Csa2G003610	1.841	0.0003098	Lipid metabolism	Abnormal inflorescence meristem (AIM1)	3.00E-294
T2-L2	blue	Csa3G730930	1.847	0.00004243	Transport	Ammonium transporter 2 (AMT2)	8E-220
T2-L2	blue	Csa6G493850	1.86	1.50E-05	Not assigned	unknown	
T2-L2	blue	Csa3G159420	1.871	1.61E-03	Not assigned	unknown	
T2-L2	blue	Csa1G528610	1.874	0.00435	Lipid metabolism	Thylakoid membrane anion transporter 1 (ANTR1)	7.00E-236
T2-L2	blue	Csa1G181360	1.875	0.001947	Transport	non-intrinsic ABC protein 9 (NAP9)	3.00E-156
T2-L2	blue	Csa3G154410	2.05	1.63E-10	Not assigned	unknown	
T2-L2	blue	Csa3G183950	2.092	0.0004285	Abiotic stress	Heat shock protein 83	0.00E+00
T2-L2	blue	Csa2G296090	2.143	2.44E-04	Not assigned	unknown	
T2-L2	blue	Csa7G291720	2.169	0.006566	Lipid metabolism	Choline kinase	4.00E-133
T2-L2	blue	Csa2G427310	2.238	0.007581	Transcription factor	MYB domain protein 73 (MYB73)	1.00E-88
T2-L2	blue	Csa3G881750	2.24	0.009561	Lipid metabolism	PR5-like receptor kinase (PR5K)	4.00E-154
T2-L2	blue	Csa5G589260	2.243	0	Amino acid metabolism	O-acetylserine(thiol) lyase (OAS-TL)	4.00E-162
T2-L2	blue	Csa3G733350	2.317	0.00004435	Cell organization	RHO guanyl-nucleotide exchange factor 11 (ROPGEF11)	9.00E-41
T2-L2	blue	Csa2G004690	2.522	0.000004593	Transcription factor	Heat shock transcription factor C1 (HSFC1)	5.00E-67
T2-L2	blue	Csa7G387690	2.631	0.000001654	Transport	Sulfate transporter 3;1 (SULTR 3;1)	3.00E-307
T2-L2	blue	Csa1G008520	2.771	0.00804	Transport	Plasma membrane proton ATPase 4 (HA4)	0.00E+00
T2-L2	blue	Csa6G514850	3.048	0.00009803	Miscellaneous	Cytochrome P450 family 77 subfamily B polypeptide 1 (CYP77B1)	1.00E-241
T2-L2	blue	Csa5G604150	3.14	0.01	Redox	Thioredoxin 2 (TRX2)	
T2-L2	blue	Csa5G223070	3.216	0.003151	Transcription factor	WRKY DNA-binding protein 54 (WRKY54)	3.00E-96
T2-L2	blue	Csa2G359890	3.289	3.70E-03	Not assigned	unknown	
T2-L2	blue	Csa1G596520	3.4	0.0002165	Protein degradation	Cystein proteinases superfamily protein	2.00E-168
T2-L2	blue	Csa7G395800	3.415	0.001823	Miscellaneous	Glutathione S-transferase phi 8 (GSTF8)	3.00E-82
T2-L2	blue	Csa3G119700	3.575	0.00005376	Transcription factor	WRKY DNA-binding protein 53 (WRKY53)	1.00E-102
T2-L2	blue	Csa6G381850	3.831	0.001156	Transport	Amino acid permease 3 (AAP3)	2.00E-234
T2-L2	blue	Csa1G033160	4.495	0.006049	Miscellaneous	Glutathione S-transferase tau 9 (GSTU9)	3.00E-68
T2-L2	blue	Csa6G486960	4.496	0.000002502	Transcription factor	WRKY DNA-binding protein 51 (WRKY51)	1.00E-36
T2-L2	blue	Csa2G058620	5.345	0.003945	Abiotic stress	DNAJ heat shock N-terminal domain-containing protein	2.00E-137

Supplementary Table 1 (Continued)

T2-L2	blue	Csa1G574970	1.827	1.18E-05	Major CHO metabolism	Hexokinase 2 (HXK2)	7.00E-51
T2-L2	blue	Csa4G618520	1.837	0.0001765	Biodegradation of xenobiotic	UDP glycosyltransferase (UGT)	1.00E-132
T2-L2	blue	Csa2G003610	1.841	0.0003098	Lipid metabolism	Abnormal inflorescence meristem (AIM1)	3.00E-294
T2-L2	blue	Csa3G730930	1.847	0.00004243	Transport	Ammonium transporter 2 (AMT2)	8E-220
T2-L2	blue	Csa6G493850	1.86	1.50E-05	Not assigned	unknown	
T2-L2	blue	Csa3G159420	1.871	1.61E-03	Not assigned	unknown	
T2-L2	blue	Csa1G528610	1.874	0.00435	Lipid metabolism	Thylakoid membrane anion transporter 1 (ANTR1)	7.00E-236
T2-L2	blue	Csa1G181360	1.875	0.001947	Transport	non-intrinsic ABC protein 9 (NAP9)	3.00E-156
T2-L2	blue	Csa3G154410	2.05	1.63E-10	Not assigned	unknown	
T2-L2	blue	Csa3G183950	2.092	0.0004285	Abiotic stress	Heat shock protein 83	0.00E+00
T2-L2	blue	Csa2G296090	2.143	2.44E-04	Not assigned	unknown	
T2-L2	blue	Csa7G291720	2.169	0.006566	Lipid metabolism	Choline kinase	4.00E-133
T2-L2	blue	Csa2G427310	2.238	0.007581	Transcription factor	MYB domain protein 73 (MYB73)	1.00E-88
T2-L2	blue	Csa3G881750	2.24	0.009561	Lipid metabolism	PR5-like receptor kinase (PR5K)	4.00E-154
T2-L2	blue	Csa5G589260	2.243	0	Amino acid metabolism	O-acetylserine(thiol) lyase (OAS-TL)	4.00E-162
T2-L2	blue	Csa3G733350	2.317	0.00004435	Cell organization	RHO guanyl-nucleotide exchange factor 11 (ROPGEF11)	9.00E-41
T2-L2	blue	Csa2G004690	2.522	0.000004593	Transcription factor	Heat shock transcription factor C1 (HSFC1)	5.00E-67
T2-L2	blue	Csa7G387690	2.631	0.000001654	Transport	Sulfate transporter 3;1 (SULTR 3;1)	3.00E-307
T2-L2	blue	Csa1G008520	2.771	0.00804	Transport	Plasma membrane proton ATPase 4 (HA4)	0.00E+00
T2-L2	blue	Csa6G514850	3.048	0.00009803	Miscellaneous	Cytochrome P450 family 77 subfamily B polypeptide 1 (CYP77B1)	1.00E-241
T2-L2	blue	Csa5G604150	3.14	0.01	Redox	Thioredoxin 2 (TRX2)	
T2-L2	blue	Csa5G223070	3.216	0.003151	Transcription factor	WRKY DNA-binding protein 54 (WRKY54)	3.00E-96
T2-L2	blue	Csa2G359890	3.289	3.70E-03	Not assigned	unknown	
T2-L2	blue	Csa1G596520	3.4	0.0002165	Protein degradation	Cystein proteinases superfamily protein	2.00E-168
T2-L2	blue	Csa7G395800	3.415	0.001823	Miscellaneous	Glutathione S-transferase phi 8 (GSTF8)	3.00E-82
T2-L2	blue	Csa3G119700	3.575	0.00005376	Transcription factor	WRKY DNA-binding protein 53 (WRKY53)	1.00E-102
T2-L2	blue	Csa6G381850	3.831	0.001156	Transport	Amino acid permease 3 (AAP3)	2.00E-234
T2-L2	blue	Csa1G033160	4.495	0.006049	Miscellaneous	Glutathione S-transferase tau 9 (GSTU9)	3.00E-68
T2-L2	blue	Csa6G486960	4.496	0.000002502	Transcription factor	WRKY DNA-binding protein 51 (WRKY51)	1.00E-36
T2-L2	blue	Csa2G058620	5.345	0.003945	Abiotic stress	DNAJ heat shock N-terminal domain-containing protein	2.00E-137

Supplementary Table 1 (Continued)

T2-L2	blue	Csa1G574970	1.827	1.18E-05	Major CHO metabolism	Hexokinase 2 (HXK2)	7.00E-51
T2-L2	blue	Csa4G618520	1.837	0.0001765	Biodegradation of xenobiotic	UDP glycosyltransferase (UGT)	1.00E-132
T2-L2	blue	Csa2G003610	1.841	0.0003098	Lipid metabolism	Abnormal inflorescence meristem (AIM1)	3.00E-294
T2-L2	blue	Csa3G730930	1.847	0.00004243	Transport	Ammonium transporter 2 (AMT2)	8E-220
T2-L2	blue	Csa6G493850	1.86	1.50E-05	Not assigned	unknown	
T2-L2	blue	Csa3G159420	1.871	1.61E-03	Not assigned	unknown	
T2-L2	blue	Csa1G528610	1.874	0.00435	Lipid metabolism	Thylakoid membrane anion transporter 1 (ANTR1)	7.00E-236
T2-L2	blue	Csa1G181360	1.875	0.001947	Transport	non-intrinsic ABC protein 9 (NAP9)	3.00E-156
T2-L2	blue	Csa3G154410	2.05	1.63E-10	Not assigned	unknown	
T2-L2	blue	Csa3G183950	2.092	0.0004285	Abiotic stress	Heat shock protein 83	0.00E+00
T2-L2	blue	Csa2G296090	2.143	2.44E-04	Not assigned	unknown	
T2-L2	blue	Csa7G291720	2.169	0.006566	Lipid metabolism	Choline kinase	4.00E-133
T2-L2	blue	Csa2G427310	2.238	0.007581	Transcription factor	MYB domain protein 73 (MYB73)	1.00E-88
T2-L2	blue	Csa3G881750	2.24	0.009561	Lipid metabolism	PR5-like receptor kinase (PR5K)	4.00E-154
T2-L2	blue	Csa5G589260	2.243	0	Amino acid metabolism	O-acetylserine(thiol) lyase (OAS-TL)	4.00E-162
T2-L2	blue	Csa3G733350	2.317	0.00004435	Cell organization	RHO guanyl-nucleotide exchange factor 11 (ROPGEF11)	9.00E-41
T2-L2	blue	Csa2G004690	2.522	0.000004593	Transcription factor	Heat shock transcription factor C1 (HSFC1)	5.00E-67
T2-L2	blue	Csa7G387690	2.631	0.000001654	Transport	Sulfate transporter 3;1 (SULTR 3;1)	3.00E-307
T2-L2	blue	Csa1G008520	2.771	0.00804	Transport	Plasma membrane proton ATPase 4 (HA4)	0.00E+00
T2-L2	blue	Csa6G514850	3.048	0.00009803	Miscellaneous	Cytochrome P450 family 77 subfamily B polypeptide 1 (CYP77B1)	1.00E-241
T2-L2	blue	Csa5G604150	3.14	0.01	Redox	Thioredoxin 2 (TRX2)	
T2-L2	blue	Csa5G223070	3.216	0.003151	Transcription factor	WRKY DNA-binding protein 54 (WRKY54)	3.00E-96
T2-L2	blue	Csa2G359890	3.289	3.70E-03	Not assigned	unknown	
T2-L2	blue	Csa1G596520	3.4	0.0002165	Protein degradation	Cystein proteinases superfamily protein	2.00E-168
T2-L2	blue	Csa7G395800	3.415	0.001823	Miscellaneous	Glutathione S-transferase phi 8 (GSTF8)	3.00E-82
T2-L2	blue	Csa3G119700	3.575	0.00005376	Transcription factor	WRKY DNA-binding protein 53 (WRKY53)	1.00E-102
T2-L2	blue	Csa6G381850	3.831	0.001156	Transport	Amino acid permease 3 (AAP3)	2.00E-234
T2-L2	blue	Csa1G033160	4.495	0.006049	Miscellaneous	Glutathione S-transferase tau 9 (GSTU9)	3.00E-68
T2-L2	blue	Csa6G486960	4.496	0.000002502	Transcription factor	WRKY DNA-binding protein 51 (WRKY51)	1.00E-36
T2-L2	blue	Csa2G058620	5.345	0.003945	Abiotic stress	DNAJ heat shock N-terminal domain-containing protein	2.00E-137

Supplementary Table 1 (Continued)

T2-L2	blue	Csa1G574970	1.827	1.18E-05	Major CHO metabolism	Hexokinase 2 (HXK2)	7.00E-51
T2-L2	blue	Csa4G618520	1.837	0.0001765	Biodegradation of xenobiotic	UDP glycosyltransferase (UGT)	1.00E-132
T2-L2	blue	Csa2G003610	1.841	0.0003098	Lipid metabolism	Abnormal inflorescence meristem (AIM1)	3.00E-294
T2-L2	blue	Csa3G730930	1.847	0.00004243	Transport	Ammonium transporter 2 (AMT2)	8E-220
T2-L2	blue	Csa6G493850	1.86	1.50E-05	Not assigned	unknown	
T2-L2	blue	Csa3G159420	1.871	1.61E-03	Not assigned	unknown	
T2-L2	blue	Csa1G528610	1.874	0.00435	Lipid metabolism	Thylakoid membrane anion transporter 1 (ANTR1)	7.00E-236
T2-L2	blue	Csa1G181360	1.875	0.001947	Transport	non-intrinsic ABC protein 9 (NAP9)	3.00E-156
T2-L2	blue	Csa3G154410	2.05	1.63E-10	Not assigned	unknown	
T2-L2	blue	Csa3G183950	2.092	0.0004285	Abiotic stress	Heat shock protein 83	0.00E+00
T2-L2	blue	Csa2G296090	2.143	2.44E-04	Not assigned	unknown	
T2-L2	blue	Csa7G291720	2.169	0.006566	Lipid metabolism	Choline kinase	4.00E-133
T2-L2	blue	Csa2G427310	2.238	0.007581	Transcription factor	MYB domain protein 73 (MYB73)	1.00E-88
T2-L2	blue	Csa3G881750	2.24	0.009561	Lipid metabolism	PR5-like receptor kinase (PR5K)	4.00E-154
T2-L2	blue	Csa5G589260	2.243	0	Amino acid metabolism	O-acetylserine(thiol) lyase (OAS-TL)	4.00E-162
T2-L2	blue	Csa3G733350	2.317	0.00004435	Cell organization	RHO guanyl-nucleotide exchange factor 11 (ROPGEF11)	9.00E-41
T2-L2	blue	Csa2G004690	2.522	0.000004593	Transcription factor	Heat shock transcription factor C1 (HSFC1)	5.00E-67
T2-L2	blue	Csa7G387690	2.631	0.000001654	Transport	Sulfate transporter 3;1 (SULTR 3;1)	3.00E-307
T2-L2	blue	Csa1G008520	2.771	0.00804	Transport	Plasma membrane proton ATPase 4 (HA4)	0.00E+00
T2-L2	blue	Csa6G514850	3.048	0.00009803	Miscellaneous	Cytochrome P450 family 77 subfamily B polypeptide 1 (CYP77B1)	1.00E-241
T2-L2	blue	Csa5G604150	3.14	0.01	Redox	Thioredoxin 2 (TRX2)	
T2-L2	blue	Csa5G223070	3.216	0.003151	Transcription factor	WRKY DNA-binding protein 54 (WRKY54)	3.00E-96
T2-L2	blue	Csa2G359890	3.289	3.70E-03	Not assigned	unknown	
T2-L2	blue	Csa1G596520	3.4	0.0002165	Protein degradation	Cystein proteinases superfamily protein	2.00E-168
T2-L2	blue	Csa7G395800	3.415	0.001823	Miscellaneous	Glutathione S-transferase phi 8 (GSTF8)	3.00E-82
T2-L2	blue	Csa3G119700	3.575	0.00005376	Transcription factor	WRKY DNA-binding protein 53 (WRKY53)	1.00E-102
T2-L2	blue	Csa6G381850	3.831	0.001156	Transport	Amino acid permease 3 (AAP3)	2.00E-234
T2-L2	blue	Csa1G033160	4.495	0.006049	Miscellaneous	Glutathione S-transferase tau 9 (GSTU9)	3.00E-68
T2-L2	blue	Csa6G486960	4.496	0.000002502	Transcription factor	WRKY DNA-binding protein 51 (WRKY51)	1.00E-36
T2-L2	blue	Csa2G058620	5.345	0.003945	Abiotic stress	DNAJ heat shock N-terminal domain-containing protein	2.00E-137

Supplementary Table 1 (Continued)

T2-L2	blue	Csa1G574970	1.827	1.18E-05	Major CHO metabolism	Hexokinase 2 (HXK2)	7.00E-51
T2-L2	blue	Csa4G618520	1.837	0.0001765	Biodegradation of xenobiotic	UDP glycosyltransferase (UGT)	1.00E-132
T2-L2	blue	Csa2G003610	1.841	0.0003098	Lipid metabolism	Abnormal inflorescence meristem (AIM1)	3.00E-294
T2-L2	blue	Csa3G730930	1.847	0.00004243	Transport	Ammonium transporter 2 (AMT2)	8E-220
T2-L2	blue	Csa6G493850	1.86	1.50E-05	Not assigned	unknown	
T2-L2	blue	Csa3G159420	1.871	1.61E-03	Not assigned	unknown	
T2-L2	blue	Csa1G528610	1.874	0.00435	Lipid metabolism	Thylakoid membrane anion transporter 1 (ANTR1)	7.00E-236
T2-L2	blue	Csa1G181360	1.875	0.001947	Transport	non-intrinsic ABC protein 9 (NAP9)	3.00E-156
T2-L2	blue	Csa3G154410	2.05	1.63E-10	Not assigned	unknown	
T2-L2	blue	Csa3G183950	2.092	0.0004285	Abiotic stress	Heat shock protein 83	0.00E+00
T2-L2	blue	Csa2G296090	2.143	2.44E-04	Not assigned	unknown	
T2-L2	blue	Csa7G291720	2.169	0.006566	Lipid metabolism	Choline kinase	4.00E-133
T2-L2	blue	Csa2G427310	2.238	0.007581	Transcription factor	MYB domain protein 73 (MYB73)	1.00E-88
T2-L2	blue	Csa3G881750	2.24	0.009561	Lipid metabolism	PR5-like receptor kinase (PR5K)	4.00E-154
T2-L2	blue	Csa5G589260	2.243	0	Amino acid metabolism	O-acetylserine(thiol) lyase (OAS-TL)	4.00E-162
T2-L2	blue	Csa3G733350	2.317	0.00004435	Cell organization	RHO guanyl-nucleotide exchange factor 11 (ROPGEF11)	9.00E-41
T2-L2	blue	Csa2G004690	2.522	0.000004593	Transcription factor	Heat shock transcription factor C1 (HSFC1)	5.00E-67
T2-L2	blue	Csa7G387690	2.631	0.000001654	Transport	Sulfate transporter 3;1 (SULTR 3;1)	3.00E-307
T2-L2	blue	Csa1G008520	2.771	0.00804	Transport	Plasma membrane proton ATPase 4 (HA4)	0.00E+00
T2-L2	blue	Csa6G514850	3.048	0.00009803	Miscellaneous	Cytochrome P450 family 77 subfamily B polypeptide 1 (CYP77B1)	1.00E-241
T2-L2	blue	Csa5G604150	3.14	0.01	Redox	Thioredoxin 2 (TRX2)	
T2-L2	blue	Csa5G223070	3.216	0.003151	Transcription factor	WRKY DNA-binding protein 54 (WRKY54)	3.00E-96
T2-L2	blue	Csa2G359890	3.289	3.70E-03	Not assigned	unknown	
T2-L2	blue	Csa1G596520	3.4	0.0002165	Protein degradation	Cystein proteinases superfamily protein	2.00E-168
T2-L2	blue	Csa7G395800	3.415	0.001823	Miscellaneous	Glutathione S-transferase phi 8 (GSTF8)	3.00E-82
T2-L2	blue	Csa3G119700	3.575	0.00005376	Transcription factor	WRKY DNA-binding protein 53 (WRKY53)	1.00E-102
T2-L2	blue	Csa6G381850	3.831	0.001156	Transport	Amino acid permease 3 (AAP3)	2.00E-234
T2-L2	blue	Csa1G033160	4.495	0.006049	Miscellaneous	Glutathione S-transferase tau 9 (GSTU9)	3.00E-68
T2-L2	blue	Csa6G486960	4.496	0.000002502	Transcription factor	WRKY DNA-binding protein 51 (WRKY51)	1.00E-36
T2-L2	blue	Csa2G058620	5.345	0.003945	Abiotic stress	DNAJ heat shock N-terminal domain-containing protein	2.00E-137

Supplementary Table 1 (Continued)

T2-L2	blue	Csa1G574970	1.827	1.18E-05	Major CHO metabolism	Hexokinase 2 (HXK2)	7.00E-51
T2-L2	blue	Csa4G618520	1.837	0.0001765	Biodegradation of xenobiotic	UDP glycosyltransferase (UGT)	1.00E-132
T2-L2	blue	Csa2G003610	1.841	0.0003098	Lipid metabolism	Abnormal inflorescence meristem (AIM1)	3.00E-294
T2-L2	blue	Csa3G730930	1.847	0.00004243	Transport	Ammonium transporter 2 (AMT2)	8E-220
T2-L2	blue	Csa6G493850	1.86	1.50E-05	Not assigned	unknown	
T2-L2	blue	Csa3G159420	1.871	1.61E-03	Not assigned	unknown	
T2-L2	blue	Csa1G528610	1.874	0.00435	Lipid metabolism	Thylakoid membrane anion transporter 1 (ANTR1)	7.00E-236
T2-L2	blue	Csa1G181360	1.875	0.001947	Transport	non-intrinsic ABC protein 9 (NAP9)	3.00E-156
T2-L2	blue	Csa3G154410	2.05	1.63E-10	Not assigned	unknown	
T2-L2	blue	Csa3G183950	2.092	0.0004285	Abiotic stress	Heat shock protein 83	0.00E+00
T2-L2	blue	Csa2G296090	2.143	2.44E-04	Not assigned	unknown	
T2-L2	blue	Csa7G291720	2.169	0.006566	Lipid metabolism	Choline kinase	4.00E-133
T2-L2	blue	Csa2G427310	2.238	0.007581	Transcription factor	MYB domain protein 73 (MYB73)	1.00E-88
T2-L2	blue	Csa3G881750	2.24	0.009561	Lipid metabolism	PR5-like receptor kinase (PR5K)	4.00E-154
T2-L2	blue	Csa5G589260	2.243	0	Amino acid metabolism	O-acetylserine(thiol) lyase (OAS-TL)	4.00E-162
T2-L2	blue	Csa3G733350	2.317	0.00004435	Cell organization	RHO guanyl-nucleotide exchange factor 11 (ROPGEF11)	9.00E-41
T2-L2	blue	Csa2G004690	2.522	0.000004593	Transcription factor	Heat shock transcription factor C1 (HSFC1)	5.00E-67
T2-L2	blue	Csa7G387690	2.631	0.000001654	Transport	Sulfate transporter 3;1 (SULTR 3;1)	3.00E-307
T2-L2	blue	Csa1G008520	2.771	0.00804	Transport	Plasma membrane proton ATPase 4 (HA4)	0.00E+00
T2-L2	blue	Csa6G514850	3.048	0.00009803	Miscellaneous	Cytochrome P450 family 77 subfamily B polypeptide 1 (CYP77B1)	1.00E-241
T2-L2	blue	Csa5G604150	3.14	0.01	Redox	Thioredoxin 2 (TRX2)	
T2-L2	blue	Csa5G223070	3.216	0.003151	Transcription factor	WRKY DNA-binding protein 54 (WRKY54)	3.00E-96
T2-L2	blue	Csa2G359890	3.289	3.70E-03	Not assigned	unknown	
T2-L2	blue	Csa1G596520	3.4	0.0002165	Protein degradation	Cystein proteinases superfamily protein	2.00E-168
T2-L2	blue	Csa7G395800	3.415	0.001823	Miscellaneous	Glutathione S-transferase phi 8 (GSTF8)	3.00E-82
T2-L2	blue	Csa3G119700	3.575	0.00005376	Transcription factor	WRKY DNA-binding protein 53 (WRKY53)	1.00E-102
T2-L2	blue	Csa6G381850	3.831	0.001156	Transport	Amino acid permease 3 (AAP3)	2.00E-234
T2-L2	blue	Csa1G033160	4.495	0.006049	Miscellaneous	Glutathione S-transferase tau 9 (GSTU9)	3.00E-68
T2-L2	blue	Csa6G486960	4.496	0.000002502	Transcription factor	WRKY DNA-binding protein 51 (WRKY51)	1.00E-36
T2-L2	blue	Csa2G058620	5.345	0.003945	Abiotic stress	DNAJ heat shock N-terminal domain-containing protein	2.00E-137

Supplementary Table 1 (Continued)

T2-L2	blue	Csa1G574970	1.827	1.18E-05	Major CHO metabolism	Hexokinase 2 (HXK2)	7.00E-51
T2-L2	blue	Csa4G618520	1.837	0.0001765	Biodegradation of xenobiotic	UDP glycosyltransferase (UGT)	1.00E-132
T2-L2	blue	Csa2G003610	1.841	0.0003098	Lipid metabolism	Abnormal inflorescence meristem (AIM1)	3.00E-294
T2-L2	blue	Csa3G730930	1.847	0.00004243	Transport	Ammonium transporter 2 (AMT2)	8E-220
T2-L2	blue	Csa6G493850	1.86	1.50E-05	Not assigned	unknown	
T2-L2	blue	Csa3G159420	1.871	1.61E-03	Not assigned	unknown	
T2-L2	blue	Csa1G528610	1.874	0.00435	Lipid metabolism	Thylakoid membrane anion transporter 1 (ANTR1)	7.00E-236
T2-L2	blue	Csa1G181360	1.875	0.001947	Transport	non-intrinsic ABC protein 9 (NAP9)	3.00E-156
T2-L2	blue	Csa3G154410	2.05	1.63E-10	Not assigned	unknown	
T2-L2	blue	Csa3G183950	2.092	0.0004285	Abiotic stress	Heat shock protein 83	0.00E+00
T2-L2	blue	Csa2G296090	2.143	2.44E-04	Not assigned	unknown	
T2-L2	blue	Csa7G291720	2.169	0.006566	Lipid metabolism	Choline kinase	4.00E-133
T2-L2	blue	Csa2G427310	2.238	0.007581	Transcription factor	MYB domain protein 73 (MYB73)	1.00E-88
T2-L2	blue	Csa3G881750	2.24	0.009561	Lipid metabolism	PR5-like receptor kinase (PR5K)	4.00E-154
T2-L2	blue	Csa5G589260	2.243	0	Amino acid metabolism	O-acetylserine(thiol) lyase (OAS-TL)	4.00E-162
T2-L2	blue	Csa3G733350	2.317	0.00004435	Cell organization	RHO guanyl-nucleotide exchange factor 11 (ROPGEF11)	9.00E-41
T2-L2	blue	Csa2G004690	2.522	0.000004593	Transcription factor	Heat shock transcription factor C1 (HSFC1)	5.00E-67
T2-L2	blue	Csa7G387690	2.631	0.000001654	Transport	Sulfate transporter 3;1 (SULTR 3;1)	3.00E-307
T2-L2	blue	Csa1G008520	2.771	0.00804	Transport	Plasma membrane proton ATPase 4 (HA4)	0.00E+00
T2-L2	blue	Csa6G514850	3.048	0.00009803	Miscellaneous	Cytochrome P450 family 77 subfamily B polypeptide 1 (CYP77B1)	1.00E-241
T2-L2	blue	Csa5G604150	3.14	0.01	Redox	Thioredoxin 2 (TRX2)	
T2-L2	blue	Csa5G223070	3.216	0.003151	Transcription factor	WRKY DNA-binding protein 54 (WRKY54)	3.00E-96
T2-L2	blue	Csa2G359890	3.289	3.70E-03	Not assigned	unknown	
T2-L2	blue	Csa1G596520	3.4	0.0002165	Protein degradation	Cystein proteinases superfamily protein	2.00E-168
T2-L2	blue	Csa7G395800	3.415	0.001823	Miscellaneous	Glutathione S-transferase phi 8 (GSTF8)	3.00E-82
T2-L2	blue	Csa3G119700	3.575	0.00005376	Transcription factor	WRKY DNA-binding protein 53 (WRKY53)	1.00E-102
T2-L2	blue	Csa6G381850	3.831	0.001156	Transport	Amino acid permease 3 (AAP3)	2.00E-234
T2-L2	blue	Csa1G033160	4.495	0.006049	Miscellaneous	Glutathione S-transferase tau 9 (GSTU9)	3.00E-68
T2-L2	blue	Csa6G486960	4.496	0.000002502	Transcription factor	WRKY DNA-binding protein 51 (WRKY51)	1.00E-36
T2-L2	blue	Csa2G058620	5.345	0.003945	Abiotic stress	DNAJ heat shock N-terminal domain-containing protein	2.00E-137

Supplementary Table 1 (Continued)

T2-L2	blue	Csa1G574970	1.827	1.18E-05	Major CHO metabolism	Hexokinase 2 (HXK2)	7.00E-51
T2-L2	blue	Csa4G618520	1.837	0.0001765	Biodegradation of xenobiotic	UDP glycosyltransferase (UGT)	1.00E-132
T2-L2	blue	Csa2G003610	1.841	0.0003098	Lipid metabolism	Abnormal inflorescence meristem (AIM1)	3.00E-294
T2-L2	blue	Csa3G730930	1.847	0.00004243	Transport	Ammonium transporter 2 (AMT2)	8E-220
T2-L2	blue	Csa6G493850	1.86	1.50E-05	Not assigned	unknown	
T2-L2	blue	Csa3G159420	1.871	1.61E-03	Not assigned	unknown	
T2-L2	blue	Csa1G528610	1.874	0.00435	Lipid metabolism	Thylakoid membrane anion transporter 1 (ANTR1)	7.00E-236
T2-L2	blue	Csa1G181360	1.875	0.001947	Transport	non-intrinsic ABC protein 9 (NAP9)	3.00E-156
T2-L2	blue	Csa3G154410	2.05	1.63E-10	Not assigned	unknown	
T2-L2	blue	Csa3G183950	2.092	0.0004285	Abiotic stress	Heat shock protein 83	0.00E+00
T2-L2	blue	Csa2G296090	2.143	2.44E-04	Not assigned	unknown	
T2-L2	blue	Csa7G291720	2.169	0.006566	Lipid metabolism	Choline kinase	4.00E-133
T2-L2	blue	Csa2G427310	2.238	0.007581	Transcription factor	MYB domain protein 73 (MYB73)	1.00E-88
T2-L2	blue	Csa3G881750	2.24	0.009561	Lipid metabolism	PR5-like receptor kinase (PR5K)	4.00E-154
T2-L2	blue	Csa5G589260	2.243	0	Amino acid metabolism	O-acetylserine(thiol) lyase (OAS-TL)	4.00E-162
T2-L2	blue	Csa3G733350	2.317	0.00004435	Cell organization	RHO guanyl-nucleotide exchange factor 11 (ROPGEF11)	9.00E-41
T2-L2	blue	Csa2G004690	2.522	0.000004593	Transcription factor	Heat shock transcription factor C1 (HSFC1)	5.00E-67
T2-L2	blue	Csa7G387690	2.631	0.000001654	Transport	Sulfate transporter 3;1 (SULTR 3;1)	3.00E-307
T2-L2	blue	Csa1G008520	2.771	0.00804	Transport	Plasma membrane proton ATPase 4 (HA4)	0.00E+00
T2-L2	blue	Csa6G514850	3.048	0.00009803	Miscellaneous	Cytochrome P450 family 77 subfamily B polypeptide 1 (CYP77B1)	1.00E-241
T2-L2	blue	Csa5G604150	3.14	0.01	Redox	Thioredoxin 2 (TRX2)	
T2-L2	blue	Csa5G223070	3.216	0.003151	Transcription factor	WRKY DNA-binding protein 54 (WRKY54)	3.00E-96
T2-L2	blue	Csa2G359890	3.289	3.70E-03	Not assigned	unknown	
T2-L2	blue	Csa1G596520	3.4	0.0002165	Protein degradation	Cystein proteinases superfamily protein	2.00E-168
T2-L2	blue	Csa7G395800	3.415	0.001823	Miscellaneous	Glutathione S-transferase phi 8 (GSTF8)	3.00E-82
T2-L2	blue	Csa3G119700	3.575	0.00005376	Transcription factor	WRKY DNA-binding protein 53 (WRKY53)	1.00E-102
T2-L2	blue	Csa6G381850	3.831	0.001156	Transport	Amino acid permease 3 (AAP3)	2.00E-234
T2-L2	blue	Csa1G033160	4.495	0.006049	Miscellaneous	Glutathione S-transferase tau 9 (GSTU9)	3.00E-68
T2-L2	blue	Csa6G486960	4.496	0.000002502	Transcription factor	WRKY DNA-binding protein 51 (WRKY51)	1.00E-36
T2-L2	blue	Csa2G058620	5.345	0.003945	Abiotic stress	DNAJ heat shock N-terminal domain-containing protein	2.00E-137

Supplementary Table 1 (Continued)

T2-L2	blue	Csa1G574970	1.827	1.18E-05	Major CHO metabolism	Hexokinase 2 (HXK2)	7.00E-51
T2-L2	blue	Csa4G618520	1.837	0.0001765	Biodegradation of xenobiotic	UDP glycosyltransferase (UGT)	1.00E-132
T2-L2	blue	Csa2G003610	1.841	0.0003098	Lipid metabolism	Abnormal inflorescence meristem (AIM1)	3.00E-294
T2-L2	blue	Csa3G730930	1.847	0.00004243	Transport	Ammonium transporter 2 (AMT2)	8E-220
T2-L2	blue	Csa6G493850	1.86	1.50E-05	Not assigned	unknown	
T2-L2	blue	Csa3G159420	1.871	1.61E-03	Not assigned	unknown	
T2-L2	blue	Csa1G528610	1.874	0.00435	Lipid metabolism	Thylakoid membrane anion transporter 1 (ANTR1)	7.00E-236
T2-L2	blue	Csa1G181360	1.875	0.001947	Transport	non-intrinsic ABC protein 9 (NAP9)	3.00E-156
T2-L2	blue	Csa3G154410	2.05	1.63E-10	Not assigned	unknown	
T2-L2	blue	Csa3G183950	2.092	0.0004285	Abiotic stress	Heat shock protein 83	0.00E+00
T2-L2	blue	Csa2G296090	2.143	2.44E-04	Not assigned	unknown	
T2-L2	blue	Csa7G291720	2.169	0.006566	Lipid metabolism	Choline kinase	4.00E-133
T2-L2	blue	Csa2G427310	2.238	0.007581	Transcription factor	MYB domain protein 73 (MYB73)	1.00E-88
T2-L2	blue	Csa3G881750	2.24	0.009561	Lipid metabolism	PR5-like receptor kinase (PR5K)	4.00E-154
T2-L2	blue	Csa5G589260	2.243	0	Amino acid metabolism	O-acetylserine(thiol) lyase (OAS-TL)	4.00E-162
T2-L2	blue	Csa3G733350	2.317	0.00004435	Cell organization	RHO guanyl-nucleotide exchange factor 11 (ROPGEF11)	9.00E-41
T2-L2	blue	Csa2G004690	2.522	0.000004593	Transcription factor	Heat shock transcription factor C1 (HSFC1)	5.00E-67
T2-L2	blue	Csa7G387690	2.631	0.000001654	Transport	Sulfate transporter 3;1 (SULTR 3;1)	3.00E-307
T2-L2	blue	Csa1G008520	2.771	0.00804	Transport	Plasma membrane proton ATPase 4 (HA4)	0.00E+00
T2-L2	blue	Csa6G514850	3.048	0.00009803	Miscellaneous	Cytochrome P450 family 77 subfamily B polypeptide 1 (CYP77B1)	1.00E-241
T2-L2	blue	Csa5G604150	3.14	0.01	Redox	Thioredoxin 2 (TRX2)	
T2-L2	blue	Csa5G223070	3.216	0.003151	Transcription factor	WRKY DNA-binding protein 54 (WRKY54)	3.00E-96
T2-L2	blue	Csa2G359890	3.289	3.70E-03	Not assigned	unknown	
T2-L2	blue	Csa1G596520	3.4	0.0002165	Protein degradation	Cystein proteinases superfamily protein	2.00E-168
T2-L2	blue	Csa7G395800	3.415	0.001823	Miscellaneous	Glutathione S-transferase phi 8 (GSTF8)	3.00E-82
T2-L2	blue	Csa3G119700	3.575	0.00005376	Transcription factor	WRKY DNA-binding protein 53 (WRKY53)	1.00E-102
T2-L2	blue	Csa6G381850	3.831	0.001156	Transport	Amino acid permease 3 (AAP3)	2.00E-234
T2-L2	blue	Csa1G033160	4.495	0.006049	Miscellaneous	Glutathione S-transferase tau 9 (GSTU9)	3.00E-68
T2-L2	blue	Csa6G486960	4.496	0.000002502	Transcription factor	WRKY DNA-binding protein 51 (WRKY51)	1.00E-36
T2-L2	blue	Csa2G058620	5.345	0.003945	Abiotic stress	DNAJ heat shock N-terminal domain-containing protein	2.00E-137

Supplementary Table 1 (Continued)

T2-L2	blue	Csa1G574970	1.827	1.18E-05	Major CHO metabolism	Hexokinase 2 (HXK2)	7.00E-51
T2-L2	blue	Csa4G618520	1.837	0.0001765	Biodegradation of xenobiotic	UDP glycosyltransferase (UGT)	1.00E-132
T2-L2	blue	Csa2G003610	1.841	0.0003098	Lipid metabolism	Abnormal inflorescence meristem (AIM1)	3.00E-294
T2-L2	blue	Csa3G730930	1.847	0.00004243	Transport	Ammonium transporter 2 (AMT2)	8E-220
T2-L2	blue	Csa6G493850	1.86	1.50E-05	Not assigned	unknown	
T2-L2	blue	Csa3G159420	1.871	1.61E-03	Not assigned	unknown	
T2-L2	blue	Csa1G528610	1.874	0.00435	Lipid metabolism	Thylakoid membrane anion transporter 1 (ANTR1)	7.00E-236
T2-L2	blue	Csa1G181360	1.875	0.001947	Transport	non-intrinsic ABC protein 9 (NAP9)	3.00E-156
T2-L2	blue	Csa3G154410	2.05	1.63E-10	Not assigned	unknown	
T2-L2	blue	Csa3G183950	2.092	0.0004285	Abiotic stress	Heat shock protein 83	0.00E+00
T2-L2	blue	Csa2G296090	2.143	2.44E-04	Not assigned	unknown	
T2-L2	blue	Csa7G291720	2.169	0.006566	Lipid metabolism	Choline kinase	4.00E-133
T2-L2	blue	Csa2G427310	2.238	0.007581	Transcription factor	MYB domain protein 73 (MYB73)	1.00E-88
T2-L2	blue	Csa3G881750	2.24	0.009561	Lipid metabolism	PR5-like receptor kinase (PR5K)	4.00E-154
T2-L2	blue	Csa5G589260	2.243	0	Amino acid metabolism	O-acetylserine(thiol) lyase (OAS-TL)	4.00E-162
T2-L2	blue	Csa3G733350	2.317	0.00004435	Cell organization	RHO guanyl-nucleotide exchange factor 11 (ROPGEF11)	9.00E-41
T2-L2	blue	Csa2G004690	2.522	0.000004593	Transcription factor	Heat shock transcription factor C1 (HSFC1)	5.00E-67
T2-L2	blue	Csa7G387690	2.631	0.000001654	Transport	Sulfate transporter 3;1 (SULTR 3;1)	3.00E-307
T2-L2	blue	Csa1G008520	2.771	0.00804	Transport	Plasma membrane proton ATPase 4 (HA4)	0.00E+00
T2-L2	blue	Csa6G514850	3.048	0.00009803	Miscellaneous	Cytochrome P450 family 77 subfamily B polypeptide 1 (CYP77B1)	1.00E-241
T2-L2	blue	Csa5G604150	3.14	0.01	Redox	Thioredoxin 2 (TRX2)	
T2-L2	blue	Csa5G223070	3.216	0.003151	Transcription factor	WRKY DNA-binding protein 54 (WRKY54)	3.00E-96
T2-L2	blue	Csa2G359890	3.289	3.70E-03	Not assigned	unknown	
T2-L2	blue	Csa1G596520	3.4	0.0002165	Protein degradation	Cystein proteinases superfamily protein	2.00E-168
T2-L2	blue	Csa7G395800	3.415	0.001823	Miscellaneous	Glutathione S-transferase phi 8 (GSTF8)	3.00E-82
T2-L2	blue	Csa3G119700	3.575	0.00005376	Transcription factor	WRKY DNA-binding protein 53 (WRKY53)	1.00E-102
T2-L2	blue	Csa6G381850	3.831	0.001156	Transport	Amino acid permease 3 (AAP3)	2.00E-234
T2-L2	blue	Csa1G033160	4.495	0.006049	Miscellaneous	Glutathione S-transferase tau 9 (GSTU9)	3.00E-68
T2-L2	blue	Csa6G486960	4.496	0.000002502	Transcription factor	WRKY DNA-binding protein 51 (WRKY51)	1.00E-36
T2-L2	blue	Csa2G058620	5.345	0.003945	Abiotic stress	DNAJ heat shock N-terminal domain-containing protein	2.00E-137

Supplementary Table 1 (Continued)

T2-L2	blue	Csa1G574970	1.827	1.18E-05	Major CHO metabolism	Hexokinase 2 (HXK2)	7.00E-51
T2-L2	blue	Csa4G618520	1.837	0.0001765	Biodegradation of xenobiotic	UDP glycosyltransferase (UGT)	1.00E-132
T2-L2	blue	Csa2G003610	1.841	0.0003098	Lipid metabolism	Abnormal inflorescence meristem (AIM1)	3.00E-294
T2-L2	blue	Csa3G730930	1.847	0.00004243	Transport	Ammonium transporter 2 (AMT2)	8E-220
T2-L2	blue	Csa6G493850	1.86	1.50E-05	Not assigned	unknown	
T2-L2	blue	Csa3G159420	1.871	1.61E-03	Not assigned	unknown	
T2-L2	blue	Csa1G528610	1.874	0.00435	Lipid metabolism	Thylakoid membrane anion transporter 1 (ANTR1)	7.00E-236
T2-L2	blue	Csa1G181360	1.875	0.001947	Transport	non-intrinsic ABC protein 9 (NAP9)	3.00E-156
T2-L2	blue	Csa3G154410	2.05	1.63E-10	Not assigned	unknown	
T2-L2	blue	Csa3G183950	2.092	0.0004285	Abiotic stress	Heat shock protein 83	0.00E+00
T2-L2	blue	Csa2G296090	2.143	2.44E-04	Not assigned	unknown	
T2-L2	blue	Csa7G291720	2.169	0.006566	Lipid metabolism	Choline kinase	4.00E-133
T2-L2	blue	Csa2G427310	2.238	0.007581	Transcription factor	MYB domain protein 73 (MYB73)	1.00E-88
T2-L2	blue	Csa3G881750	2.24	0.009561	Lipid metabolism	PR5-like receptor kinase (PR5K)	4.00E-154
T2-L2	blue	Csa5G589260	2.243	0	Amino acid metabolism	O-acetylserine(thiol) lyase (OAS-TL)	4.00E-162
T2-L2	blue	Csa3G733350	2.317	0.00004435	Cell organization	RHO guanyl-nucleotide exchange factor 11 (ROPGEF11)	9.00E-41
T2-L2	blue	Csa2G004690	2.522	0.000004593	Transcription factor	Heat shock transcription factor C1 (HSFC1)	5.00E-67
T2-L2	blue	Csa7G387690	2.631	0.000001654	Transport	Sulfate transporter 3;1 (SULTR 3;1)	3.00E-307
T2-L2	blue	Csa1G008520	2.771	0.00804	Transport	Plasma membrane proton ATPase 4 (HA4)	0.00E+00
T2-L2	blue	Csa6G514850	3.048	0.00009803	Miscellaneous	Cytochrome P450 family 77 subfamily B polypeptide 1 (CYP77B1)	1.00E-241
T2-L2	blue	Csa5G604150	3.14	0.01	Redox	Thioredoxin 2 (TRX2)	
T2-L2	blue	Csa5G223070	3.216	0.003151	Transcription factor	WRKY DNA-binding protein 54 (WRKY54)	3.00E-96
T2-L2	blue	Csa2G359890	3.289	3.70E-03	Not assigned	unknown	
T2-L2	blue	Csa1G596520	3.4	0.0002165	Protein degradation	Cystein proteinases superfamily protein	2.00E-168
T2-L2	blue	Csa7G395800	3.415	0.001823	Miscellaneous	Glutathione S-transferase phi 8 (GSTF8)	3.00E-82
T2-L2	blue	Csa3G119700	3.575	0.00005376	Transcription factor	WRKY DNA-binding protein 53 (WRKY53)	1.00E-102
T2-L2	blue	Csa6G381850	3.831	0.001156	Transport	Amino acid permease 3 (AAP3)	2.00E-234
T2-L2	blue	Csa1G033160	4.495	0.006049	Miscellaneous	Glutathione S-transferase tau 9 (GSTU9)	3.00E-68
T2-L2	blue	Csa6G486960	4.496	0.000002502	Transcription factor	WRKY DNA-binding protein 51 (WRKY51)	1.00E-36
T2-L2	blue	Csa2G058620	5.345	0.003945	Abiotic stress	DNAJ heat shock N-terminal domain-containing protein	2.00E-137

Supplementary Table 1 (Continued)

T2-L2	blue	Csa1G574970	1.827	1.18E-05	Major CHO metabolism	Hexokinase 2 (HXK2)	7.00E-51
T2-L2	blue	Csa4G618520	1.837	0.0001765	Biodegradation of xenobiotic	UDP glycosyltransferase (UGT)	1.00E-132
T2-L2	blue	Csa2G003610	1.841	0.0003098	Lipid metabolism	Abnormal inflorescence meristem (AIM1)	3.00E-294
T2-L2	blue	Csa3G730930	1.847	0.00004243	Transport	Ammonium transporter 2 (AMT2)	8E-220
T2-L2	blue	Csa6G493850	1.86	1.50E-05	Not assigned	unknown	
T2-L2	blue	Csa3G159420	1.871	1.61E-03	Not assigned	unknown	
T2-L2	blue	Csa1G528610	1.874	0.00435	Lipid metabolism	Thylakoid membrane anion transporter 1 (ANTR1)	7.00E-236
T2-L2	blue	Csa1G181360	1.875	0.001947	Transport	non-intrinsic ABC protein 9 (NAP9)	3.00E-156
T2-L2	blue	Csa3G154410	2.05	1.63E-10	Not assigned	unknown	
T2-L2	blue	Csa3G183950	2.092	0.0004285	Abiotic stress	Heat shock protein 83	0.00E+00
T2-L2	blue	Csa2G296090	2.143	2.44E-04	Not assigned	unknown	
T2-L2	blue	Csa7G291720	2.169	0.006566	Lipid metabolism	Choline kinase	4.00E-133
T2-L2	blue	Csa2G427310	2.238	0.007581	Transcription factor	MYB domain protein 73 (MYB73)	1.00E-88
T2-L2	blue	Csa3G881750	2.24	0.009561	Lipid metabolism	PR5-like receptor kinase (PR5K)	4.00E-154
T2-L2	blue	Csa5G589260	2.243	0	Amino acid metabolism	O-acetylserine(thiol) lyase (OAS-TL)	4.00E-162
T2-L2	blue	Csa3G733350	2.317	0.00004435	Cell organization	RHO guanyl-nucleotide exchange factor 11 (ROPGEF11)	9.00E-41
T2-L2	blue	Csa2G004690	2.522	0.000004593	Transcription factor	Heat shock transcription factor C1 (HSFC1)	5.00E-67
T2-L2	blue	Csa7G387690	2.631	0.000001654	Transport	Sulfate transporter 3;1 (SULTR 3;1)	3.00E-307
T2-L2	blue	Csa1G008520	2.771	0.00804	Transport	Plasma membrane proton ATPase 4 (HA4)	0.00E+00
T2-L2	blue	Csa6G514850	3.048	0.00009803	Miscellaneous	Cytochrome P450 family 77 subfamily B polypeptide 1 (CYP77B1)	1.00E-241
T2-L2	blue	Csa5G604150	3.14	0.01	Redox	Thioredoxin 2 (TRX2)	
T2-L2	blue	Csa5G223070	3.216	0.003151	Transcription factor	WRKY DNA-binding protein 54 (WRKY54)	3.00E-96
T2-L2	blue	Csa2G359890	3.289	3.70E-03	Not assigned	unknown	
T2-L2	blue	Csa1G596520	3.4	0.0002165	Protein degradation	Cystein proteinases superfamily protein	2.00E-168
T2-L2	blue	Csa7G395800	3.415	0.001823	Miscellaneous	Glutathione S-transferase phi 8 (GSTF8)	3.00E-82
T2-L2	blue	Csa3G119700	3.575	0.00005376	Transcription factor	WRKY DNA-binding protein 53 (WRKY53)	1.00E-102
T2-L2	blue	Csa6G381850	3.831	0.001156	Transport	Amino acid permease 3 (AAP3)	2.00E-234
T2-L2	blue	Csa1G033160	4.495	0.006049	Miscellaneous	Glutathione S-transferase tau 9 (GSTU9)	3.00E-68
T2-L2	blue	Csa6G486960	4.496	0.000002502	Transcription factor	WRKY DNA-binding protein 51 (WRKY51)	1.00E-36
T2-L2	blue	Csa2G058620	5.345	0.003945	Abiotic stress	DNAJ heat shock N-terminal domain-containing protein	2.00E-137

Supplementary Table 1 (Continued)

T2-L2	blue	Csa1G574970	1.827	1.18E-05	Major CHO metabolism	Hexokinase 2 (HXK2)	7.00E-51
T2-L2	blue	Csa4G618520	1.837	0.0001765	Biodegradation of xenobiotic	UDP glycosyltransferase (UGT)	1.00E-132
T2-L2	blue	Csa2G003610	1.841	0.0003098	Lipid metabolism	Abnormal inflorescence meristem (AIM1)	3.00E-294
T2-L2	blue	Csa3G730930	1.847	0.00004243	Transport	Ammonium transporter 2 (AMT2)	8E-220
T2-L2	blue	Csa6G493850	1.86	1.50E-05	Not assigned	unknown	
T2-L2	blue	Csa3G159420	1.871	1.61E-03	Not assigned	unknown	
T2-L2	blue	Csa1G528610	1.874	0.00435	Lipid metabolism	Thylakoid membrane anion transporter 1 (ANTR1)	7.00E-236
T2-L2	blue	Csa1G181360	1.875	0.001947	Transport	non-intrinsic ABC protein 9 (NAP9)	3.00E-156
T2-L2	blue	Csa3G154410	2.05	1.63E-10	Not assigned	unknown	
T2-L2	blue	Csa3G183950	2.092	0.0004285	Abiotic stress	Heat shock protein 83	0.00E+00
T2-L2	blue	Csa2G296090	2.143	2.44E-04	Not assigned	unknown	
T2-L2	blue	Csa7G291720	2.169	0.006566	Lipid metabolism	Choline kinase	4.00E-133
T2-L2	blue	Csa2G427310	2.238	0.007581	Transcription factor	MYB domain protein 73 (MYB73)	1.00E-88
T2-L2	blue	Csa3G881750	2.24	0.009561	Lipid metabolism	PR5-like receptor kinase (PR5K)	4.00E-154
T2-L2	blue	Csa5G589260	2.243	0	Amino acid metabolism	O-acetylserine(thiol) lyase (OAS-TL)	4.00E-162
T2-L2	blue	Csa3G733350	2.317	0.00004435	Cell organization	RHO guanyl-nucleotide exchange factor 11 (ROPGEF11)	9.00E-41
T2-L2	blue	Csa2G004690	2.522	0.000004593	Transcription factor	Heat shock transcription factor C1 (HSFC1)	5.00E-67
T2-L2	blue	Csa7G387690	2.631	0.000001654	Transport	Sulfate transporter 3;1 (SULTR 3;1)	3.00E-307
T2-L2	blue	Csa1G008520	2.771	0.00804	Transport	Plasma membrane proton ATPase 4 (HA4)	0.00E+00
T2-L2	blue	Csa6G514850	3.048	0.00009803	Miscellaneous	Cytochrome P450 family 77 subfamily B polypeptide 1 (CYP77B1)	1.00E-241
T2-L2	blue	Csa5G604150	3.14	0.01	Redox	Thioredoxin 2 (TRX2)	
T2-L2	blue	Csa5G223070	3.216	0.003151	Transcription factor	WRKY DNA-binding protein 54 (WRKY54)	3.00E-96
T2-L2	blue	Csa2G359890	3.289	3.70E-03	Not assigned	unknown	
T2-L2	blue	Csa1G596520	3.4	0.0002165	Protein degradation	Cystein proteinases superfamily protein	2.00E-168
T2-L2	blue	Csa7G395800	3.415	0.001823	Miscellaneous	Glutathione S-transferase phi 8 (GSTF8)	3.00E-82
T2-L2	blue	Csa3G119700	3.575	0.00005376	Transcription factor	WRKY DNA-binding protein 53 (WRKY53)	1.00E-102
T2-L2	blue	Csa6G381850	3.831	0.001156	Transport	Amino acid permease 3 (AAP3)	2.00E-234
T2-L2	blue	Csa1G033160	4.495	0.006049	Miscellaneous	Glutathione S-transferase tau 9 (GSTU9)	3.00E-68
T2-L2	blue	Csa6G486960	4.496	0.000002502	Transcription factor	WRKY DNA-binding protein 51 (WRKY51)	1.00E-36
T2-L2	blue	Csa2G058620	5.345	0.003945	Abiotic stress	DNAJ heat shock N-terminal domain-containing protein	2.00E-137

Supplementary Table 1 (Continued)

T2-L2	blue	Csa1G574970	1.827	1.18E-05	Major CHO metabolism	Hexokinase 2 (HXK2)	7.00E-51
T2-L2	blue	Csa4G618520	1.837	0.0001765	Biodegradation of xenobiotic	UDP glycosyltransferase (UGT)	1.00E-132
T2-L2	blue	Csa2G003610	1.841	0.0003098	Lipid metabolism	Abnormal inflorescence meristem (AIM1)	3.00E-294
T2-L2	blue	Csa3G730930	1.847	0.00004243	Transport	Ammonium transporter 2 (AMT2)	8E-220
T2-L2	blue	Csa6G493850	1.86	1.50E-05	Not assigned	unknown	
T2-L2	blue	Csa3G159420	1.871	1.61E-03	Not assigned	unknown	
T2-L2	blue	Csa1G528610	1.874	0.00435	Lipid metabolism	Thylakoid membrane anion transporter 1 (ANTR1)	7.00E-236
T2-L2	blue	Csa1G181360	1.875	0.001947	Transport	non-intrinsic ABC protein 9 (NAP9)	3.00E-156
T2-L2	blue	Csa3G154410	2.05	1.63E-10	Not assigned	unknown	
T2-L2	blue	Csa3G183950	2.092	0.0004285	Abiotic stress	Heat shock protein 83	0.00E+00
T2-L2	blue	Csa2G296090	2.143	2.44E-04	Not assigned	unknown	
T2-L2	blue	Csa7G291720	2.169	0.006566	Lipid metabolism	Choline kinase	4.00E-133
T2-L2	blue	Csa2G427310	2.238	0.007581	Transcription factor	MYB domain protein 73 (MYB73)	1.00E-88
T2-L2	blue	Csa3G881750	2.24	0.009561	Lipid metabolism	PR5-like receptor kinase (PR5K)	4.00E-154
T2-L2	blue	Csa5G589260	2.243	0	Amino acid metabolism	O-acetylserine(thiol) lyase (OAS-TL)	4.00E-162
T2-L2	blue	Csa3G733350	2.317	0.00004435	Cell organization	RHO guanyl-nucleotide exchange factor 11 (ROPGEF11)	9.00E-41
T2-L2	blue	Csa2G004690	2.522	0.000004593	Transcription factor	Heat shock transcription factor C1 (HSFC1)	5.00E-67
T2-L2	blue	Csa7G387690	2.631	0.000001654	Transport	Sulfate transporter 3;1 (SULTR 3;1)	3.00E-307
T2-L2	blue	Csa1G008520	2.771	0.00804	Transport	Plasma membrane proton ATPase 4 (HA4)	0.00E+00
T2-L2	blue	Csa6G514850	3.048	0.00009803	Miscellaneous	Cytochrome P450 family 77 subfamily B polypeptide 1 (CYP77B1)	1.00E-241
T2-L2	blue	Csa5G604150	3.14	0.01	Redox	Thioredoxin 2 (TRX2)	
T2-L2	blue	Csa5G223070	3.216	0.003151	Transcription factor	WRKY DNA-binding protein 54 (WRKY54)	3.00E-96
T2-L2	blue	Csa2G359890	3.289	3.70E-03	Not assigned	unknown	
T2-L2	blue	Csa1G596520	3.4	0.0002165	Protein degradation	Cystein proteinases superfamily protein	2.00E-168
T2-L2	blue	Csa7G395800	3.415	0.001823	Miscellaneous	Glutathione S-transferase phi 8 (GSTF8)	3.00E-82
T2-L2	blue	Csa3G119700	3.575	0.00005376	Transcription factor	WRKY DNA-binding protein 53 (WRKY53)	1.00E-102
T2-L2	blue	Csa6G381850	3.831	0.001156	Transport	Amino acid permease 3 (AAP3)	2.00E-234
T2-L2	blue	Csa1G033160	4.495	0.006049	Miscellaneous	Glutathione S-transferase tau 9 (GSTU9)	3.00E-68
T2-L2	blue	Csa6G486960	4.496	0.000002502	Transcription factor	WRKY DNA-binding protein 51 (WRKY51)	1.00E-36
T2-L2	blue	Csa2G058620	5.345	0.003945	Abiotic stress	DNAJ heat shock N-terminal domain-containing protein	2.00E-137

Supplementary Table 1 (Continued)

T2-L2	blue	Csa1G574970	1.827	1.18E-05	Major CHO metabolism	Hexokinase 2 (HXK2)	7.00E-51
T2-L2	blue	Csa4G618520	1.837	0.0001765	Biodegradation of xenobiotic	UDP glycosyltransferase (UGT)	1.00E-132
T2-L2	blue	Csa2G003610	1.841	0.0003098	Lipid metabolism	Abnormal inflorescence meristem (AIM1)	3.00E-294
T2-L2	blue	Csa3G730930	1.847	0.00004243	Transport	Ammonium transporter 2 (AMT2)	8E-220
T2-L2	blue	Csa6G493850	1.86	1.50E-05	Not assigned	unknown	
T2-L2	blue	Csa3G159420	1.871	1.61E-03	Not assigned	unknown	
T2-L2	blue	Csa1G528610	1.874	0.00435	Lipid metabolism	Thylakoid membrane anion transporter 1 (ANTR1)	7.00E-236
T2-L2	blue	Csa1G181360	1.875	0.001947	Transport	non-intrinsic ABC protein 9 (NAP9)	3.00E-156
T2-L2	blue	Csa3G154410	2.05	1.63E-10	Not assigned	unknown	
T2-L2	blue	Csa3G183950	2.092	0.0004285	Abiotic stress	Heat shock protein 83	0.00E+00
T2-L2	blue	Csa2G296090	2.143	2.44E-04	Not assigned	unknown	
T2-L2	blue	Csa7G291720	2.169	0.006566	Lipid metabolism	Choline kinase	4.00E-133
T2-L2	blue	Csa2G427310	2.238	0.007581	Transcription factor	MYB domain protein 73 (MYB73)	1.00E-88
T2-L2	blue	Csa3G881750	2.24	0.009561	Lipid metabolism	PR5-like receptor kinase (PR5K)	4.00E-154
T2-L2	blue	Csa5G589260	2.243	0	Amino acid metabolism	O-acetylserine(thiol) lyase (OAS-TL)	4.00E-162
T2-L2	blue	Csa3G733350	2.317	0.00004435	Cell organization	RHO guanyl-nucleotide exchange factor 11 (ROPGEF11)	9.00E-41
T2-L2	blue	Csa2G004690	2.522	0.000004593	Transcription factor	Heat shock transcription factor C1 (HSFC1)	5.00E-67
T2-L2	blue	Csa7G387690	2.631	0.000001654	Transport	Sulfate transporter 3;1 (SULTR 3;1)	3.00E-307
T2-L2	blue	Csa1G008520	2.771	0.00804	Transport	Plasma membrane proton ATPase 4 (HA4)	0.00E+00
T2-L2	blue	Csa6G514850	3.048	0.00009803	Miscellaneous	Cytochrome P450 family 77 subfamily B polypeptide 1 (CYP77B1)	1.00E-241
T2-L2	blue	Csa5G604150	3.14	0.01	Redox	Thioredoxin 2 (TRX2)	
T2-L2	blue	Csa5G223070	3.216	0.003151	Transcription factor	WRKY DNA-binding protein 54 (WRKY54)	3.00E-96
T2-L2	blue	Csa2G359890	3.289	3.70E-03	Not assigned	unknown	
T2-L2	blue	Csa1G596520	3.4	0.0002165	Protein degradation	Cystein proteinases superfamily protein	2.00E-168
T2-L2	blue	Csa7G395800	3.415	0.001823	Miscellaneous	Glutathione S-transferase phi 8 (GSTF8)	3.00E-82
T2-L2	blue	Csa3G119700	3.575	0.00005376	Transcription factor	WRKY DNA-binding protein 53 (WRKY53)	1.00E-102
T2-L2	blue	Csa6G381850	3.831	0.001156	Transport	Amino acid permease 3 (AAP3)	2.00E-234
T2-L2	blue	Csa1G033160	4.495	0.006049	Miscellaneous	Glutathione S-transferase tau 9 (GSTU9)	3.00E-68
T2-L2	blue	Csa6G486960	4.496	0.000002502	Transcription factor	WRKY DNA-binding protein 51 (WRKY51)	1.00E-36
T2-L2	blue	Csa2G058620	5.345	0.003945	Abiotic stress	DNAJ heat shock N-terminal domain-containing protein	2.00E-137

Supplementary Table 1 (Continued)

T2-L2	blue	Csa1G574970	1.827	1.18E-05	Major CHO metabolism	Hexokinase 2 (HXK2)	7.00E-51
T2-L2	blue	Csa4G618520	1.837	0.0001765	Biodegradation of xenobiotic	UDP glycosyltransferase (UGT)	1.00E-132
T2-L2	blue	Csa2G003610	1.841	0.0003098	Lipid metabolism	Abnormal inflorescence meristem (AIM1)	3.00E-294
T2-L2	blue	Csa3G730930	1.847	0.00004243	Transport	Ammonium transporter 2 (AMT2)	8E-220
T2-L2	blue	Csa6G493850	1.86	1.50E-05	Not assigned	unknown	
T2-L2	blue	Csa3G159420	1.871	1.61E-03	Not assigned	unknown	
T2-L2	blue	Csa1G528610	1.874	0.00435	Lipid metabolism	Thylakoid membrane anion transporter 1 (ANTR1)	7.00E-236
T2-L2	blue	Csa1G181360	1.875	0.001947	Transport	non-intrinsic ABC protein 9 (NAP9)	3.00E-156
T2-L2	blue	Csa3G154410	2.05	1.63E-10	Not assigned	unknown	
T2-L2	blue	Csa3G183950	2.092	0.0004285	Abiotic stress	Heat shock protein 83	0.00E+00
T2-L2	blue	Csa2G296090	2.143	2.44E-04	Not assigned	unknown	
T2-L2	blue	Csa7G291720	2.169	0.006566	Lipid metabolism	Choline kinase	4.00E-133
T2-L2	blue	Csa2G427310	2.238	0.007581	Transcription factor	MYB domain protein 73 (MYB73)	1.00E-88
T2-L2	blue	Csa3G881750	2.24	0.009561	Lipid metabolism	PR5-like receptor kinase (PR5K)	4.00E-154
T2-L2	blue	Csa5G589260	2.243	0	Amino acid metabolism	O-acetylserine(thiol) lyase (OAS-TL)	4.00E-162
T2-L2	blue	Csa3G733350	2.317	0.00004435	Cell organization	RHO guanyl-nucleotide exchange factor 11 (ROPGEF11)	9.00E-41
T2-L2	blue	Csa2G004690	2.522	0.000004593	Transcription factor	Heat shock transcription factor C1 (HSFC1)	5.00E-67
T2-L2	blue	Csa7G387690	2.631	0.000001654	Transport	Sulfate transporter 3;1 (SULTR 3;1)	3.00E-307
T2-L2	blue	Csa1G008520	2.771	0.00804	Transport	Plasma membrane proton ATPase 4 (HA4)	0.00E+00
T2-L2	blue	Csa6G514850	3.048	0.00009803	Miscellaneous	Cytochrome P450 family 77 subfamily B polypeptide 1 (CYP77B1)	1.00E-241
T2-L2	blue	Csa5G604150	3.14	0.01	Redox	Thioredoxin 2 (TRX2)	
T2-L2	blue	Csa5G223070	3.216	0.003151	Transcription factor	WRKY DNA-binding protein 54 (WRKY54)	3.00E-96
T2-L2	blue	Csa2G359890	3.289	3.70E-03	Not assigned	unknown	
T2-L2	blue	Csa1G596520	3.4	0.0002165	Protein degradation	Cystein proteinases superfamily protein	2.00E-168
T2-L2	blue	Csa7G395800	3.415	0.001823	Miscellaneous	Glutathione S-transferase phi 8 (GSTF8)	3.00E-82
T2-L2	blue	Csa3G119700	3.575	0.00005376	Transcription factor	WRKY DNA-binding protein 53 (WRKY53)	1.00E-102
T2-L2	blue	Csa6G381850	3.831	0.001156	Transport	Amino acid permease 3 (AAP3)	2.00E-234
T2-L2	blue	Csa1G033160	4.495	0.006049	Miscellaneous	Glutathione S-transferase tau 9 (GSTU9)	3.00E-68
T2-L2	blue	Csa6G486960	4.496	0.000002502	Transcription factor	WRKY DNA-binding protein 51 (WRKY51)	1.00E-36
T2-L2	blue	Csa2G058620	5.345	0.003945	Abiotic stress	DNAJ heat shock N-terminal domain-containing protein	2.00E-137

Supplementary Table 1 (Continued)

T2-L2	blue	Csa1G574970	1.827	1.18E-05	Major CHO metabolism	Hexokinase 2 (HXK2)	7.00E-51
T2-L2	blue	Csa4G618520	1.837	0.0001765	Biodegradation of xenobiotic	UDP glycosyltransferase (UGT)	1.00E-132
T2-L2	blue	Csa2G003610	1.841	0.0003098	Lipid metabolism	Abnormal inflorescence meristem (AIM1)	3.00E-294
T2-L2	blue	Csa3G730930	1.847	0.00004243	Transport	Ammonium transporter 2 (AMT2)	8E-220
T2-L2	blue	Csa6G493850	1.86	1.50E-05	Not assigned	unknown	
T2-L2	blue	Csa3G159420	1.871	1.61E-03	Not assigned	unknown	
T2-L2	blue	Csa1G528610	1.874	0.00435	Lipid metabolism	Thylakoid membrane anion transporter 1 (ANTR1)	7.00E-236
T2-L2	blue	Csa1G181360	1.875	0.001947	Transport	non-intrinsic ABC protein 9 (NAP9)	3.00E-156
T2-L2	blue	Csa3G154410	2.05	1.63E-10	Not assigned	unknown	
T2-L2	blue	Csa3G183950	2.092	0.0004285	Abiotic stress	Heat shock protein 83	0.00E+00
T2-L2	blue	Csa2G296090	2.143	2.44E-04	Not assigned	unknown	
T2-L2	blue	Csa7G291720	2.169	0.006566	Lipid metabolism	Choline kinase	4.00E-133
T2-L2	blue	Csa2G427310	2.238	0.007581	Transcription factor	MYB domain protein 73 (MYB73)	1.00E-88
T2-L2	blue	Csa3G881750	2.24	0.009561	Lipid metabolism	PR5-like receptor kinase (PR5K)	4.00E-154
T2-L2	blue	Csa5G589260	2.243	0	Amino acid metabolism	O-acetylserine(thiol) lyase (OAS-TL)	4.00E-162
T2-L2	blue	Csa3G733350	2.317	0.00004435	Cell organization	RHO guanyl-nucleotide exchange factor 11 (ROPGEF11)	9.00E-41
T2-L2	blue	Csa2G004690	2.522	0.000004593	Transcription factor	Heat shock transcription factor C1 (HSFC1)	5.00E-67
T2-L2	blue	Csa7G387690	2.631	0.000001654	Transport	Sulfate transporter 3;1 (SULTR 3;1)	3.00E-307
T2-L2	blue	Csa1G008520	2.771	0.00804	Transport	Plasma membrane proton ATPase 4 (HA4)	0.00E+00
T2-L2	blue	Csa6G514850	3.048	0.00009803	Miscellaneous	Cytochrome P450 family 77 subfamily B polypeptide 1 (CYP77B1)	1.00E-241
T2-L2	blue	Csa5G604150	3.14	0.01	Redox	Thioredoxin 2 (TRX2)	
T2-L2	blue	Csa5G223070	3.216	0.003151	Transcription factor	WRKY DNA-binding protein 54 (WRKY54)	3.00E-96
T2-L2	blue	Csa2G359890	3.289	3.70E-03	Not assigned	unknown	
T2-L2	blue	Csa1G596520	3.4	0.0002165	Protein degradation	Cystein proteinases superfamily protein	2.00E-168
T2-L2	blue	Csa7G395800	3.415	0.001823	Miscellaneous	Glutathione S-transferase phi 8 (GSTF8)	3.00E-82
T2-L2	blue	Csa3G119700	3.575	0.00005376	Transcription factor	WRKY DNA-binding protein 53 (WRKY53)	1.00E-102
T2-L2	blue	Csa6G381850	3.831	0.001156	Transport	Amino acid permease 3 (AAP3)	2.00E-234
T2-L2	blue	Csa1G033160	4.495	0.006049	Miscellaneous	Glutathione S-transferase tau 9 (GSTU9)	3.00E-68
T2-L2	blue	Csa6G486960	4.496	0.000002502	Transcription factor	WRKY DNA-binding protein 51 (WRKY51)	1.00E-36
T2-L2	blue	Csa2G058620	5.345	0.003945	Abiotic stress	DNAJ heat shock N-terminal domain-containing protein	2.00E-137

Supplementary Table 1 (Continued)

T2-L2	blue	Csa1G574970	1.827	1.18E-05	Major CHO metabolism	Hexokinase 2 (HXK2)	7.00E-51
T2-L2	blue	Csa4G618520	1.837	0.0001765	Biodegradation of xenobiotic	UDP glycosyltransferase (UGT)	1.00E-132
T2-L2	blue	Csa2G003610	1.841	0.0003098	Lipid metabolism	Abnormal inflorescence meristem (AIM1)	3.00E-294
T2-L2	blue	Csa3G730930	1.847	0.00004243	Transport	Ammonium transporter 2 (AMT2)	8E-220
T2-L2	blue	Csa6G493850	1.86	1.50E-05	Not assigned	unknown	
T2-L2	blue	Csa3G159420	1.871	1.61E-03	Not assigned	unknown	
T2-L2	blue	Csa1G528610	1.874	0.00435	Lipid metabolism	Thylakoid membrane anion transporter 1 (ANTR1)	7.00E-236
T2-L2	blue	Csa1G181360	1.875	0.001947	Transport	non-intrinsic ABC protein 9 (NAP9)	3.00E-156
T2-L2	blue	Csa3G154410	2.05	1.63E-10	Not assigned	unknown	
T2-L2	blue	Csa3G183950	2.092	0.0004285	Abiotic stress	Heat shock protein 83	0.00E+00
T2-L2	blue	Csa2G296090	2.143	2.44E-04	Not assigned	unknown	
T2-L2	blue	Csa7G291720	2.169	0.006566	Lipid metabolism	Choline kinase	4.00E-133
T2-L2	blue	Csa2G427310	2.238	0.007581	Transcription factor	MYB domain protein 73 (MYB73)	1.00E-88
T2-L2	blue	Csa3G881750	2.24	0.009561	Lipid metabolism	PR5-like receptor kinase (PR5K)	4.00E-154
T2-L2	blue	Csa5G589260	2.243	0	Amino acid metabolism	O-acetylserine(thiol) lyase (OAS-TL)	4.00E-162
T2-L2	blue	Csa3G733350	2.317	0.00004435	Cell organization	RHO guanyl-nucleotide exchange factor 11 (ROPGEF11)	9.00E-41
T2-L2	blue	Csa2G004690	2.522	0.000004593	Transcription factor	Heat shock transcription factor C1 (HSFC1)	5.00E-67
T2-L2	blue	Csa7G387690	2.631	0.000001654	Transport	Sulfate transporter 3;1 (SULTR 3;1)	3.00E-307
T2-L2	blue	Csa1G008520	2.771	0.00804	Transport	Plasma membrane proton ATPase 4 (HA4)	0.00E+00
T2-L2	blue	Csa6G514850	3.048	0.00009803	Miscellaneous	Cytochrome P450 family 77 subfamily B polypeptide 1 (CYP77B1)	1.00E-241
T2-L2	blue	Csa5G604150	3.14	0.01	Redox	Thioredoxin 2 (TRX2)	
T2-L2	blue	Csa5G223070	3.216	0.003151	Transcription factor	WRKY DNA-binding protein 54 (WRKY54)	3.00E-96
T2-L2	blue	Csa2G359890	3.289	3.70E-03	Not assigned	unknown	
T2-L2	blue	Csa1G596520	3.4	0.0002165	Protein degradation	Cystein proteinases superfamily protein	2.00E-168
T2-L2	blue	Csa7G395800	3.415	0.001823	Miscellaneous	Glutathione S-transferase phi 8 (GSTF8)	3.00E-82
T2-L2	blue	Csa3G119700	3.575	0.00005376	Transcription factor	WRKY DNA-binding protein 53 (WRKY53)	1.00E-102
T2-L2	blue	Csa6G381850	3.831	0.001156	Transport	Amino acid permease 3 (AAP3)	2.00E-234
T2-L2	blue	Csa1G033160	4.495	0.006049	Miscellaneous	Glutathione S-transferase tau 9 (GSTU9)	3.00E-68
T2-L2	blue	Csa6G486960	4.496	0.000002502	Transcription factor	WRKY DNA-binding protein 51 (WRKY51)	1.00E-36
T2-L2	blue	Csa2G058620	5.345	0.003945	Abiotic stress	DNAJ heat shock N-terminal domain-containing protein	2.00E-137

Supplementary Table 1 (Continued)

T2-L2	blue	Csa1G574970	1.827	1.18E-05	Major CHO metabolism	Hexokinase 2 (HXK2)	7.00E-51
T2-L2	blue	Csa4G618520	1.837	0.0001765	Biodegradation of xenobiotic	UDP glycosyltransferase (UGT)	1.00E-132
T2-L2	blue	Csa2G003610	1.841	0.0003098	Lipid metabolism	Abnormal inflorescence meristem (AIM1)	3.00E-294
T2-L2	blue	Csa3G730930	1.847	0.00004243	Transport	Ammonium transporter 2 (AMT2)	8E-220
T2-L2	blue	Csa6G493850	1.86	1.50E-05	Not assigned	unknown	
T2-L2	blue	Csa3G159420	1.871	1.61E-03	Not assigned	unknown	
T2-L2	blue	Csa1G528610	1.874	0.00435	Lipid metabolism	Thylakoid membrane anion transporter 1 (ANTR1)	7.00E-236
T2-L2	blue	Csa1G181360	1.875	0.001947	Transport	non-intrinsic ABC protein 9 (NAP9)	3.00E-156
T2-L2	blue	Csa3G154410	2.05	1.63E-10	Not assigned	unknown	
T2-L2	blue	Csa3G183950	2.092	0.0004285	Abiotic stress	Heat shock protein 83	0.00E+00
T2-L2	blue	Csa2G296090	2.143	2.44E-04	Not assigned	unknown	
T2-L2	blue	Csa7G291720	2.169	0.006566	Lipid metabolism	Choline kinase	4.00E-133
T2-L2	blue	Csa2G427310	2.238	0.007581	Transcription factor	MYB domain protein 73 (MYB73)	1.00E-88
T2-L2	blue	Csa3G881750	2.24	0.009561	Lipid metabolism	PR5-like receptor kinase (PR5K)	4.00E-154
T2-L2	blue	Csa5G589260	2.243	0	Amino acid metabolism	O-acetylserine(thiol) lyase (OAS-TL)	4.00E-162
T2-L2	blue	Csa3G733350	2.317	0.00004435	Cell organization	RHO guanyl-nucleotide exchange factor 11 (ROPGEF11)	9.00E-41
T2-L2	blue	Csa2G004690	2.522	0.000004593	Transcription factor	Heat shock transcription factor C1 (HSFC1)	5.00E-67
T2-L2	blue	Csa7G387690	2.631	0.000001654	Transport	Sulfate transporter 3;1 (SULTR 3;1)	3.00E-307
T2-L2	blue	Csa1G008520	2.771	0.00804	Transport	Plasma membrane proton ATPase 4 (HA4)	0.00E+00
T2-L2	blue	Csa6G514850	3.048	0.00009803	Miscellaneous	Cytochrome P450 family 77 subfamily B polypeptide 1 (CYP77B1)	1.00E-241
T2-L2	blue	Csa5G604150	3.14	0.01	Redox	Thioredoxin 2 (TRX2)	
T2-L2	blue	Csa5G223070	3.216	0.003151	Transcription factor	WRKY DNA-binding protein 54 (WRKY54)	3.00E-96
T2-L2	blue	Csa2G359890	3.289	3.70E-03	Not assigned	unknown	
T2-L2	blue	Csa1G596520	3.4	0.0002165	Protein degradation	Cystein proteinases superfamily protein	2.00E-168
T2-L2	blue	Csa7G395800	3.415	0.001823	Miscellaneous	Glutathione S-transferase phi 8 (GSTF8)	3.00E-82
T2-L2	blue	Csa3G119700	3.575	0.00005376	Transcription factor	WRKY DNA-binding protein 53 (WRKY53)	1.00E-102
T2-L2	blue	Csa6G381850	3.831	0.001156	Transport	Amino acid permease 3 (AAP3)	2.00E-234
T2-L2	blue	Csa1G033160	4.495	0.006049	Miscellaneous	Glutathione S-transferase tau 9 (GSTU9)	3.00E-68
T2-L2	blue	Csa6G486960	4.496	0.000002502	Transcription factor	WRKY DNA-binding protein 51 (WRKY51)	1.00E-36
T2-L2	blue	Csa2G058620	5.345	0.003945	Abiotic stress	DNAJ heat shock N-terminal domain-containing protein	2.00E-137

Supplementary Table 1 (Continued)

T2-L2	blue	Csa1G574970	1.827	1.18E-05	Major CHO metabolism	Hexokinase 2 (HXK2)	7.00E-51
T2-L2	blue	Csa4G618520	1.837	0.0001765	Biodegradation of xenobiotic	UDP glycosyltransferase (UGT)	1.00E-132
T2-L2	blue	Csa2G003610	1.841	0.0003098	Lipid metabolism	Abnormal inflorescence meristem (AIM1)	3.00E-294
T2-L2	blue	Csa3G730930	1.847	0.00004243	Transport	Ammonium transporter 2 (AMT2)	8E-220
T2-L2	blue	Csa6G493850	1.86	1.50E-05	Not assigned	unknown	
T2-L2	blue	Csa3G159420	1.871	1.61E-03	Not assigned	unknown	
T2-L2	blue	Csa1G528610	1.874	0.00435	Lipid metabolism	Thylakoid membrane anion transporter 1 (ANTR1)	7.00E-236
T2-L2	blue	Csa1G181360	1.875	0.001947	Transport	non-intrinsic ABC protein 9 (NAP9)	3.00E-156
T2-L2	blue	Csa3G154410	2.05	1.63E-10	Not assigned	unknown	
T2-L2	blue	Csa3G183950	2.092	0.0004285	Abiotic stress	Heat shock protein 83	0.00E+00
T2-L2	blue	Csa2G296090	2.143	2.44E-04	Not assigned	unknown	
T2-L2	blue	Csa7G291720	2.169	0.006566	Lipid metabolism	Choline kinase	4.00E-133
T2-L2	blue	Csa2G427310	2.238	0.007581	Transcription factor	MYB domain protein 73 (MYB73)	1.00E-88
T2-L2	blue	Csa3G881750	2.24	0.009561	Lipid metabolism	PR5-like receptor kinase (PR5K)	4.00E-154
T2-L2	blue	Csa5G589260	2.243	0	Amino acid metabolism	O-acetylserine(thiol) lyase (OAS-TL)	4.00E-162
T2-L2	blue	Csa3G733350	2.317	0.00004435	Cell organization	RHO guanyl-nucleotide exchange factor 11 (ROPGEF11)	9.00E-41
T2-L2	blue	Csa2G004690	2.522	0.000004593	Transcription factor	Heat shock transcription factor C1 (HSFC1)	5.00E-67
T2-L2	blue	Csa7G387690	2.631	0.000001654	Transport	Sulfate transporter 3;1 (SULTR 3;1)	3.00E-307
T2-L2	blue	Csa1G008520	2.771	0.00804	Transport	Plasma membrane proton ATPase 4 (HA4)	0.00E+00
T2-L2	blue	Csa6G514850	3.048	0.00009803	Miscellaneous	Cytochrome P450 family 77 subfamily B polypeptide 1 (CYP77B1)	1.00E-241
T2-L2	blue	Csa5G604150	3.14	0.01	Redox	Thioredoxin 2 (TRX2)	
T2-L2	blue	Csa5G223070	3.216	0.003151	Transcription factor	WRKY DNA-binding protein 54 (WRKY54)	3.00E-96
T2-L2	blue	Csa2G359890	3.289	3.70E-03	Not assigned	unknown	
T2-L2	blue	Csa1G596520	3.4	0.0002165	Protein degradation	Cystein proteinases superfamily protein	2.00E-168
T2-L2	blue	Csa7G395800	3.415	0.001823	Miscellaneous	Glutathione S-transferase phi 8 (GSTF8)	3.00E-82
T2-L2	blue	Csa3G119700	3.575	0.00005376	Transcription factor	WRKY DNA-binding protein 53 (WRKY53)	1.00E-102
T2-L2	blue	Csa6G381850	3.831	0.001156	Transport	Amino acid permease 3 (AAP3)	2.00E-234
T2-L2	blue	Csa1G033160	4.495	0.006049	Miscellaneous	Glutathione S-transferase tau 9 (GSTU9)	3.00E-68
T2-L2	blue	Csa6G486960	4.496	0.000002502	Transcription factor	WRKY DNA-binding protein 51 (WRKY51)	1.00E-36
T2-L2	blue	Csa2G058620	5.345	0.003945	Abiotic stress	DNAJ heat shock N-terminal domain-containing protein	2.00E-137

Supplementary Table 1 (Continued)

T2-L2	blue	Csa1G574970	1.827	1.18E-05	Major CHO metabolism	Hexokinase 2 (HXK2)	7.00E-51
T2-L2	blue	Csa4G618520	1.837	0.0001765	Biodegradation of xenobiotic	UDP glycosyltransferase (UGT)	1.00E-132
T2-L2	blue	Csa2G003610	1.841	0.0003098	Lipid metabolism	Abnormal inflorescence meristem (AIM1)	3.00E-294
T2-L2	blue	Csa3G730930	1.847	0.00004243	Transport	Ammonium transporter 2 (AMT2)	8E-220
T2-L2	blue	Csa6G493850	1.86	1.50E-05	Not assigned	unknown	
T2-L2	blue	Csa3G159420	1.871	1.61E-03	Not assigned	unknown	
T2-L2	blue	Csa1G528610	1.874	0.00435	Lipid metabolism	Thylakoid membrane anion transporter 1 (ANTR1)	7.00E-236
T2-L2	blue	Csa1G181360	1.875	0.001947	Transport	non-intrinsic ABC protein 9 (NAP9)	3.00E-156
T2-L2	blue	Csa3G154410	2.05	1.63E-10	Not assigned	unknown	
T2-L2	blue	Csa3G183950	2.092	0.0004285	Abiotic stress	Heat shock protein 83	0.00E+00
T2-L2	blue	Csa2G296090	2.143	2.44E-04	Not assigned	unknown	
T2-L2	blue	Csa7G291720	2.169	0.006566	Lipid metabolism	Choline kinase	4.00E-133
T2-L2	blue	Csa2G427310	2.238	0.007581	Transcription factor	MYB domain protein 73 (MYB73)	1.00E-88
T2-L2	blue	Csa3G881750	2.24	0.009561	Lipid metabolism	PR5-like receptor kinase (PR5K)	4.00E-154
T2-L2	blue	Csa5G589260	2.243	0	Amino acid metabolism	O-acetylserine(thiol) lyase (OAS-TL)	4.00E-162
T2-L2	blue	Csa3G733350	2.317	0.00004435	Cell organization	RHO guanyl-nucleotide exchange factor 11 (ROPGEF11)	9.00E-41
T2-L2	blue	Csa2G004690	2.522	0.000004593	Transcription factor	Heat shock transcription factor C1 (HSFC1)	5.00E-67
T2-L2	blue	Csa7G387690	2.631	0.000001654	Transport	Sulfate transporter 3;1 (SULTR 3;1)	3.00E-307
T2-L2	blue	Csa1G008520	2.771	0.00804	Transport	Plasma membrane proton ATPase 4 (HA4)	0.00E+00
T2-L2	blue	Csa6G514850	3.048	0.00009803	Miscellaneous	Cytochrome P450 family 77 subfamily B polypeptide 1 (CYP77B1)	1.00E-241
T2-L2	blue	Csa5G604150	3.14	0.01	Redox	Thioredoxin 2 (TRX2)	
T2-L2	blue	Csa5G223070	3.216	0.003151	Transcription factor	WRKY DNA-binding protein 54 (WRKY54)	3.00E-96
T2-L2	blue	Csa2G359890	3.289	3.70E-03	Not assigned	unknown	
T2-L2	blue	Csa1G596520	3.4	0.0002165	Protein degradation	Cystein proteinases superfamily protein	2.00E-168
T2-L2	blue	Csa7G395800	3.415	0.001823	Miscellaneous	Glutathione S-transferase phi 8 (GSTF8)	3.00E-82
T2-L2	blue	Csa3G119700	3.575	0.00005376	Transcription factor	WRKY DNA-binding protein 53 (WRKY53)	1.00E-102
T2-L2	blue	Csa6G381850	3.831	0.001156	Transport	Amino acid permease 3 (AAP3)	2.00E-234
T2-L2	blue	Csa1G033160	4.495	0.006049	Miscellaneous	Glutathione S-transferase tau 9 (GSTU9)	3.00E-68
T2-L2	blue	Csa6G486960	4.496	0.000002502	Transcription factor	WRKY DNA-binding protein 51 (WRKY51)	1.00E-36
T2-L2	blue	Csa2G058620	5.345	0.003945	Abiotic stress	DNAJ heat shock N-terminal domain-containing protein	2.00E-137

Supplementary Table 1 (Continued)

T2-L2	blue	Csa1G574970	1.827	1.18E-05	Major CHO metabolism	Hexokinase 2 (HXK2)	7.00E-51
T2-L2	blue	Csa4G618520	1.837	0.0001765	Biodegradation of xenobiotic	UDP glycosyltransferase (UGT)	1.00E-132
T2-L2	blue	Csa2G003610	1.841	0.0003098	Lipid metabolism	Abnormal inflorescence meristem (AIM1)	3.00E-294
T2-L2	blue	Csa3G730930	1.847	0.00004243	Transport	Ammonium transporter 2 (AMT2)	8E-220
T2-L2	blue	Csa6G493850	1.86	1.50E-05	Not assigned	unknown	
T2-L2	blue	Csa3G159420	1.871	1.61E-03	Not assigned	unknown	
T2-L2	blue	Csa1G528610	1.874	0.00435	Lipid metabolism	Thylakoid membrane anion transporter 1 (ANTR1)	7.00E-236
T2-L2	blue	Csa1G181360	1.875	0.001947	Transport	non-intrinsic ABC protein 9 (NAP9)	3.00E-156
T2-L2	blue	Csa3G154410	2.05	1.63E-10	Not assigned	unknown	
T2-L2	blue	Csa3G183950	2.092	0.0004285	Abiotic stress	Heat shock protein 83	0.00E+00
T2-L2	blue	Csa2G296090	2.143	2.44E-04	Not assigned	unknown	
T2-L2	blue	Csa7G291720	2.169	0.006566	Lipid metabolism	Choline kinase	4.00E-133
T2-L2	blue	Csa2G427310	2.238	0.007581	Transcription factor	MYB domain protein 73 (MYB73)	1.00E-88
T2-L2	blue	Csa3G881750	2.24	0.009561	Lipid metabolism	PR5-like receptor kinase (PR5K)	4.00E-154
T2-L2	blue	Csa5G589260	2.243	0	Amino acid metabolism	O-acetylserine(thiol) lyase (OAS-TL)	4.00E-162
T2-L2	blue	Csa3G733350	2.317	0.00004435	Cell organization	RHO guanyl-nucleotide exchange factor 11 (ROPGEF11)	9.00E-41
T2-L2	blue	Csa2G004690	2.522	0.000004593	Transcription factor	Heat shock transcription factor C1 (HSFC1)	5.00E-67
T2-L2	blue	Csa7G387690	2.631	0.000001654	Transport	Sulfate transporter 3;1 (SULTR 3;1)	3.00E-307
T2-L2	blue	Csa1G008520	2.771	0.00804	Transport	Plasma membrane proton ATPase 4 (HA4)	0.00E+00
T2-L2	blue	Csa6G514850	3.048	0.00009803	Miscellaneous	Cytochrome P450 family 77 subfamily B polypeptide 1 (CYP77B1)	1.00E-241
T2-L2	blue	Csa5G604150	3.14	0.01	Redox	Thioredoxin 2 (TRX2)	
T2-L2	blue	Csa5G223070	3.216	0.003151	Transcription factor	WRKY DNA-binding protein 54 (WRKY54)	3.00E-96
T2-L2	blue	Csa2G359890	3.289	3.70E-03	Not assigned	unknown	
T2-L2	blue	Csa1G596520	3.4	0.0002165	Protein degradation	Cystein proteinases superfamily protein	2.00E-168
T2-L2	blue	Csa7G395800	3.415	0.001823	Miscellaneous	Glutathione S-transferase phi 8 (GSTF8)	3.00E-82
T2-L2	blue	Csa3G119700	3.575	0.00005376	Transcription factor	WRKY DNA-binding protein 53 (WRKY53)	1.00E-102
T2-L2	blue	Csa6G381850	3.831	0.001156	Transport	Amino acid permease 3 (AAP3)	2.00E-234
T2-L2	blue	Csa1G033160	4.495	0.006049	Miscellaneous	Glutathione S-transferase tau 9 (GSTU9)	3.00E-68
T2-L2	blue	Csa6G486960	4.496	0.000002502	Transcription factor	WRKY DNA-binding protein 51 (WRKY51)	1.00E-36
T2-L2	blue	Csa2G058620	5.345	0.003945	Abiotic stress	DNAJ heat shock N-terminal domain-containing protein	2.00E-137

Supplementary Table 1 (Continued)

T2-L2	blue	Csa1G574970	1.827	1.18E-05	Major CHO metabolism	Hexokinase 2 (HXK2)	7.00E-51
T2-L2	blue	Csa4G618520	1.837	0.0001765	Biodegradation of xenobiotic	UDP glycosyltransferase (UGT)	1.00E-132
T2-L2	blue	Csa2G003610	1.841	0.0003098	Lipid metabolism	Abnormal inflorescence meristem (AIM1)	3.00E-294
T2-L2	blue	Csa3G730930	1.847	0.00004243	Transport	Ammonium transporter 2 (AMT2)	8E-220
T2-L2	blue	Csa6G493850	1.86	1.50E-05	Not assigned	unknown	
T2-L2	blue	Csa3G159420	1.871	1.61E-03	Not assigned	unknown	
T2-L2	blue	Csa1G528610	1.874	0.00435	Lipid metabolism	Thylakoid membrane anion transporter 1 (ANTR1)	7.00E-236
T2-L2	blue	Csa1G181360	1.875	0.001947	Transport	non-intrinsic ABC protein 9 (NAP9)	3.00E-156
T2-L2	blue	Csa3G154410	2.05	1.63E-10	Not assigned	unknown	
T2-L2	blue	Csa3G183950	2.092	0.0004285	Abiotic stress	Heat shock protein 83	0.00E+00
T2-L2	blue	Csa2G296090	2.143	2.44E-04	Not assigned	unknown	
T2-L2	blue	Csa7G291720	2.169	0.006566	Lipid metabolism	Choline kinase	4.00E-133
T2-L2	blue	Csa2G427310	2.238	0.007581	Transcription factor	MYB domain protein 73 (MYB73)	1.00E-88
T2-L2	blue	Csa3G881750	2.24	0.009561	Lipid metabolism	PR5-like receptor kinase (PR5K)	4.00E-154
T2-L2	blue	Csa5G589260	2.243	0	Amino acid metabolism	O-acetylserine(thiol) lyase (OAS-TL)	4.00E-162
T2-L2	blue	Csa3G733350	2.317	0.00004435	Cell organization	RHO guanyl-nucleotide exchange factor 11 (ROPGEF11)	9.00E-41
T2-L2	blue	Csa2G004690	2.522	0.000004593	Transcription factor	Heat shock transcription factor C1 (HSFC1)	5.00E-67
T2-L2	blue	Csa7G387690	2.631	0.000001654	Transport	Sulfate transporter 3;1 (SULTR 3;1)	3.00E-307
T2-L2	blue	Csa1G008520	2.771	0.00804	Transport	Plasma membrane proton ATPase 4 (HA4)	0.00E+00
T2-L2	blue	Csa6G514850	3.048	0.00009803	Miscellaneous	Cytochrome P450 family 77 subfamily B polypeptide 1 (CYP77B1)	1.00E-241
T2-L2	blue	Csa5G604150	3.14	0.01	Redox	Thioredoxin 2 (TRX2)	
T2-L2	blue	Csa5G223070	3.216	0.003151	Transcription factor	WRKY DNA-binding protein 54 (WRKY54)	3.00E-96
T2-L2	blue	Csa2G359890	3.289	3.70E-03	Not assigned	unknown	
T2-L2	blue	Csa1G596520	3.4	0.0002165	Protein degradation	Cystein proteinases superfamily protein	2.00E-168
T2-L2	blue	Csa7G395800	3.415	0.001823	Miscellaneous	Glutathione S-transferase phi 8 (GSTF8)	3.00E-82
T2-L2	blue	Csa3G119700	3.575	0.00005376	Transcription factor	WRKY DNA-binding protein 53 (WRKY53)	1.00E-102
T2-L2	blue	Csa6G381850	3.831	0.001156	Transport	Amino acid permease 3 (AAP3)	2.00E-234
T2-L2	blue	Csa1G033160	4.495	0.006049	Miscellaneous	Glutathione S-transferase tau 9 (GSTU9)	3.00E-68
T2-L2	blue	Csa6G486960	4.496	0.000002502	Transcription factor	WRKY DNA-binding protein 51 (WRKY51)	1.00E-36
T2-L2	blue	Csa2G058620	5.345	0.003945	Abiotic stress	DNAJ heat shock N-terminal domain-containing protein	2.00E-137

Supplementary Table 1 (Continued)

T2-L2	blue	Csa1G574970	1.827	1.18E-05	Major CHO metabolism	Hexokinase 2 (HXK2)	7.00E-51
T2-L2	blue	Csa4G618520	1.837	0.0001765	Biodegradation of xenobiotic	UDP glycosyltransferase (UGT)	1.00E-132
T2-L2	blue	Csa2G003610	1.841	0.0003098	Lipid metabolism	Abnormal inflorescence meristem (AIM1)	3.00E-294
T2-L2	blue	Csa3G730930	1.847	0.00004243	Transport	Ammonium transporter 2 (AMT2)	8E-220
T2-L2	blue	Csa6G493850	1.86	1.50E-05	Not assigned	unknown	
T2-L2	blue	Csa3G159420	1.871	1.61E-03	Not assigned	unknown	
T2-L2	blue	Csa1G528610	1.874	0.00435	Lipid metabolism	Thylakoid membrane anion transporter 1 (ANTR1)	7.00E-236
T2-L2	blue	Csa1G181360	1.875	0.001947	Transport	non-intrinsic ABC protein 9 (NAP9)	3.00E-156
T2-L2	blue	Csa3G154410	2.05	1.63E-10	Not assigned	unknown	
T2-L2	blue	Csa3G183950	2.092	0.0004285	Abiotic stress	Heat shock protein 83	0.00E+00
T2-L2	blue	Csa2G296090	2.143	2.44E-04	Not assigned	unknown	
T2-L2	blue	Csa7G291720	2.169	0.006566	Lipid metabolism	Choline kinase	4.00E-133
T2-L2	blue	Csa2G427310	2.238	0.007581	Transcription factor	MYB domain protein 73 (MYB73)	1.00E-88
T2-L2	blue	Csa3G881750	2.24	0.009561	Lipid metabolism	PR5-like receptor kinase (PR5K)	4.00E-154
T2-L2	blue	Csa5G589260	2.243	0	Amino acid metabolism	O-acetylserine(thiol) lyase (OAS-TL)	4.00E-162
T2-L2	blue	Csa3G733350	2.317	0.00004435	Cell organization	RHO guanyl-nucleotide exchange factor 11 (ROPGEF11)	9.00E-41
T2-L2	blue	Csa2G004690	2.522	0.000004593	Transcription factor	Heat shock transcription factor C1 (HSFC1)	5.00E-67
T2-L2	blue	Csa7G387690	2.631	0.000001654	Transport	Sulfate transporter 3;1 (SULTR 3;1)	3.00E-307
T2-L2	blue	Csa1G008520	2.771	0.00804	Transport	Plasma membrane proton ATPase 4 (HA4)	0.00E+00
T2-L2	blue	Csa6G514850	3.048	0.00009803	Miscellaneous	Cytochrome P450 family 77 subfamily B polypeptide 1 (CYP77B1)	1.00E-241
T2-L2	blue	Csa5G604150	3.14	0.01	Redox	Thioredoxin 2 (TRX2)	
T2-L2	blue	Csa5G223070	3.216	0.003151	Transcription factor	WRKY DNA-binding protein 54 (WRKY54)	3.00E-96
T2-L2	blue	Csa2G359890	3.289	3.70E-03	Not assigned	unknown	
T2-L2	blue	Csa1G596520	3.4	0.0002165	Protein degradation	Cystein proteinases superfamily protein	2.00E-168
T2-L2	blue	Csa7G395800	3.415	0.001823	Miscellaneous	Glutathione S-transferase phi 8 (GSTF8)	3.00E-82
T2-L2	blue	Csa3G119700	3.575	0.00005376	Transcription factor	WRKY DNA-binding protein 53 (WRKY53)	1.00E-102
T2-L2	blue	Csa6G381850	3.831	0.001156	Transport	Amino acid permease 3 (AAP3)	2.00E-234
T2-L2	blue	Csa1G033160	4.495	0.006049	Miscellaneous	Glutathione S-transferase tau 9 (GSTU9)	3.00E-68
T2-L2	blue	Csa6G486960	4.496	0.000002502	Transcription factor	WRKY DNA-binding protein 51 (WRKY51)	1.00E-36
T2-L2	blue	Csa2G058620	5.345	0.003945	Abiotic stress	DNAJ heat shock N-terminal domain-containing protein	2.00E-137

Supplementary Table 1 (Continued)

T2-L2	blue	Csa1G574970	1.827	1.18E-05	Major CHO metabolism	Hexokinase 2 (HXK2)	7.00E-51
T2-L2	blue	Csa4G618520	1.837	0.0001765	Biodegradation of xenobiotic	UDP glycosyltransferase (UGT)	1.00E-132
T2-L2	blue	Csa2G003610	1.841	0.0003098	Lipid metabolism	Abnormal inflorescence meristem (AIM1)	3.00E-294
T2-L2	blue	Csa3G730930	1.847	0.00004243	Transport	Ammonium transporter 2 (AMT2)	8E-220
T2-L2	blue	Csa6G493850	1.86	1.50E-05	Not assigned	unknown	
T2-L2	blue	Csa3G159420	1.871	1.61E-03	Not assigned	unknown	
T2-L2	blue	Csa1G528610	1.874	0.00435	Lipid metabolism	Thylakoid membrane anion transporter 1 (ANTR1)	7.00E-236
T2-L2	blue	Csa1G181360	1.875	0.001947	Transport	non-intrinsic ABC protein 9 (NAP9)	3.00E-156
T2-L2	blue	Csa3G154410	2.05	1.63E-10	Not assigned	unknown	
T2-L2	blue	Csa3G183950	2.092	0.0004285	Abiotic stress	Heat shock protein 83	0.00E+00
T2-L2	blue	Csa2G296090	2.143	2.44E-04	Not assigned	unknown	
T2-L2	blue	Csa7G291720	2.169	0.006566	Lipid metabolism	Choline kinase	4.00E-133
T2-L2	blue	Csa2G427310	2.238	0.007581	Transcription factor	MYB domain protein 73 (MYB73)	1.00E-88
T2-L2	blue	Csa3G881750	2.24	0.009561	Lipid metabolism	PR5-like receptor kinase (PR5K)	4.00E-154
T2-L2	blue	Csa5G589260	2.243	0	Amino acid metabolism	O-acetylserine(thiol) lyase (OAS-TL)	4.00E-162
T2-L2	blue	Csa3G733350	2.317	0.00004435	Cell organization	RHO guanyl-nucleotide exchange factor 11 (ROPGEF11)	9.00E-41
T2-L2	blue	Csa2G004690	2.522	0.000004593	Transcription factor	Heat shock transcription factor C1 (HSFC1)	5.00E-67
T2-L2	blue	Csa7G387690	2.631	0.000001654	Transport	Sulfate transporter 3;1 (SULTR 3;1)	3.00E-307
T2-L2	blue	Csa1G008520	2.771	0.00804	Transport	Plasma membrane proton ATPase 4 (HA4)	0.00E+00
T2-L2	blue	Csa6G514850	3.048	0.00009803	Miscellaneous	Cytochrome P450 family 77 subfamily B polypeptide 1 (CYP77B1)	1.00E-241
T2-L2	blue	Csa5G604150	3.14	0.01	Redox	Thioredoxin 2 (TRX2)	
T2-L2	blue	Csa5G223070	3.216	0.003151	Transcription factor	WRKY DNA-binding protein 54 (WRKY54)	3.00E-96
T2-L2	blue	Csa2G359890	3.289	3.70E-03	Not assigned	unknown	
T2-L2	blue	Csa1G596520	3.4	0.0002165	Protein degradation	Cystein proteinases superfamily protein	2.00E-168
T2-L2	blue	Csa7G395800	3.415	0.001823	Miscellaneous	Glutathione S-transferase phi 8 (GSTF8)	3.00E-82
T2-L2	blue	Csa3G119700	3.575	0.00005376	Transcription factor	WRKY DNA-binding protein 53 (WRKY53)	1.00E-102
T2-L2	blue	Csa6G381850	3.831	0.001156	Transport	Amino acid permease 3 (AAP3)	2.00E-234
T2-L2	blue	Csa1G033160	4.495	0.006049	Miscellaneous	Glutathione S-transferase tau 9 (GSTU9)	3.00E-68
T2-L2	blue	Csa6G486960	4.496	0.000002502	Transcription factor	WRKY DNA-binding protein 51 (WRKY51)	1.00E-36
T2-L2	blue	Csa2G058620	5.345	0.003945	Abiotic stress	DNAJ heat shock N-terminal domain-containing protein	2.00E-137

Supplementary Table 1 (Continued)

T2-L2	blue	Csa1G574970	1.827	1.18E-05	Major CHO metabolism	Hexokinase 2 (HXK2)	7.00E-51
T2-L2	blue	Csa4G618520	1.837	0.0001765	Biodegradation of xenobiotic	UDP glycosyltransferase (UGT)	1.00E-132
T2-L2	blue	Csa2G003610	1.841	0.0003098	Lipid metabolism	Abnormal inflorescence meristem (AIM1)	3.00E-294
T2-L2	blue	Csa3G730930	1.847	0.00004243	Transport	Ammonium transporter 2 (AMT2)	8E-220
T2-L2	blue	Csa6G493850	1.86	1.50E-05	Not assigned	unknown	
T2-L2	blue	Csa3G159420	1.871	1.61E-03	Not assigned	unknown	
T2-L2	blue	Csa1G528610	1.874	0.00435	Lipid metabolism	Thylakoid membrane anion transporter 1 (ANTR1)	7.00E-236
T2-L2	blue	Csa1G181360	1.875	0.001947	Transport	non-intrinsic ABC protein 9 (NAP9)	3.00E-156
T2-L2	blue	Csa3G154410	2.05	1.63E-10	Not assigned	unknown	
T2-L2	blue	Csa3G183950	2.092	0.0004285	Abiotic stress	Heat shock protein 83	0.00E+00
T2-L2	blue	Csa2G296090	2.143	2.44E-04	Not assigned	unknown	
T2-L2	blue	Csa7G291720	2.169	0.006566	Lipid metabolism	Choline kinase	4.00E-133
T2-L2	blue	Csa2G427310	2.238	0.007581	Transcription factor	MYB domain protein 73 (MYB73)	1.00E-88
T2-L2	blue	Csa3G881750	2.24	0.009561	Lipid metabolism	PR5-like receptor kinase (PR5K)	4.00E-154
T2-L2	blue	Csa5G589260	2.243	0	Amino acid metabolism	O-acetylserine(thiol) lyase (OAS-TL)	4.00E-162
T2-L2	blue	Csa3G733350	2.317	0.00004435	Cell organization	RHO guanyl-nucleotide exchange factor 11 (ROPGEF11)	9.00E-41
T2-L2	blue	Csa2G004690	2.522	0.000004593	Transcription factor	Heat shock transcription factor C1 (HSFC1)	5.00E-67
T2-L2	blue	Csa7G387690	2.631	0.000001654	Transport	Sulfate transporter 3;1 (SULTR 3;1)	3.00E-307
T2-L2	blue	Csa1G008520	2.771	0.00804	Transport	Plasma membrane proton ATPase 4 (HA4)	0.00E+00
T2-L2	blue	Csa6G514850	3.048	0.00009803	Miscellaneous	Cytochrome P450 family 77 subfamily B polypeptide 1 (CYP77B1)	1.00E-241
T2-L2	blue	Csa5G604150	3.14	0.01	Redox	Thioredoxin 2 (TRX2)	
T2-L2	blue	Csa5G223070	3.216	0.003151	Transcription factor	WRKY DNA-binding protein 54 (WRKY54)	3.00E-96
T2-L2	blue	Csa2G359890	3.289	3.70E-03	Not assigned	unknown	
T2-L2	blue	Csa1G596520	3.4	0.0002165	Protein degradation	Cystein proteinases superfamily protein	2.00E-168
T2-L2	blue	Csa7G395800	3.415	0.001823	Miscellaneous	Glutathione S-transferase phi 8 (GSTF8)	3.00E-82
T2-L2	blue	Csa3G119700	3.575	0.00005376	Transcription factor	WRKY DNA-binding protein 53 (WRKY53)	1.00E-102
T2-L2	blue	Csa6G381850	3.831	0.001156	Transport	Amino acid permease 3 (AAP3)	2.00E-234
T2-L2	blue	Csa1G033160	4.495	0.006049	Miscellaneous	Glutathione S-transferase tau 9 (GSTU9)	3.00E-68
T2-L2	blue	Csa6G486960	4.496	0.000002502	Transcription factor	WRKY DNA-binding protein 51 (WRKY51)	1.00E-36
T2-L2	blue	Csa2G058620	5.345	0.003945	Abiotic stress	DNAJ heat shock N-terminal domain-containing protein	2.00E-137

Supplementary Table 1 (Continued)

T2-L2	blue	Csa1G574970	1.827	1.18E-05	Major CHO metabolism	Hexokinase 2 (HXK2)	7.00E-51
T2-L2	blue	Csa4G618520	1.837	0.0001765	Biodegradation of xenobiotic	UDP glycosyltransferase (UGT)	1.00E-132
T2-L2	blue	Csa2G003610	1.841	0.0003098	Lipid metabolism	Abnormal inflorescence meristem (AIM1)	3.00E-294
T2-L2	blue	Csa3G730930	1.847	0.00004243	Transport	Ammonium transporter 2 (AMT2)	8E-220
T2-L2	blue	Csa6G493850	1.86	1.50E-05	Not assigned	unknown	
T2-L2	blue	Csa3G159420	1.871	1.61E-03	Not assigned	unknown	
T2-L2	blue	Csa1G528610	1.874	0.00435	Lipid metabolism	Thylakoid membrane anion transporter 1 (ANTR1)	7.00E-236
T2-L2	blue	Csa1G181360	1.875	0.001947	Transport	non-intrinsic ABC protein 9 (NAP9)	3.00E-156
T2-L2	blue	Csa3G154410	2.05	1.63E-10	Not assigned	unknown	
T2-L2	blue	Csa3G183950	2.092	0.0004285	Abiotic stress	Heat shock protein 83	0.00E+00
T2-L2	blue	Csa2G296090	2.143	2.44E-04	Not assigned	unknown	
T2-L2	blue	Csa7G291720	2.169	0.006566	Lipid metabolism	Choline kinase	4.00E-133
T2-L2	blue	Csa2G427310	2.238	0.007581	Transcription factor	MYB domain protein 73 (MYB73)	1.00E-88
T2-L2	blue	Csa3G881750	2.24	0.009561	Lipid metabolism	PR5-like receptor kinase (PR5K)	4.00E-154
T2-L2	blue	Csa5G589260	2.243	0	Amino acid metabolism	O-acetylserine(thiol) lyase (OAS-TL)	4.00E-162
T2-L2	blue	Csa3G733350	2.317	0.00004435	Cell organization	RHO guanyl-nucleotide exchange factor 11 (ROPGEF11)	9.00E-41
T2-L2	blue	Csa2G004690	2.522	0.000004593	Transcription factor	Heat shock transcription factor C1 (HSFC1)	5.00E-67
T2-L2	blue	Csa7G387690	2.631	0.000001654	Transport	Sulfate transporter 3;1 (SULTR 3;1)	3.00E-307
T2-L2	blue	Csa1G008520	2.771	0.00804	Transport	Plasma membrane proton ATPase 4 (HA4)	0.00E+00
T2-L2	blue	Csa6G514850	3.048	0.00009803	Miscellaneous	Cytochrome P450 family 77 subfamily B polypeptide 1 (CYP77B1)	1.00E-241
T2-L2	blue	Csa5G604150	3.14	0.01	Redox	Thioredoxin 2 (TRX2)	
T2-L2	blue	Csa5G223070	3.216	0.003151	Transcription factor	WRKY DNA-binding protein 54 (WRKY54)	3.00E-96
T2-L2	blue	Csa2G359890	3.289	3.70E-03	Not assigned	unknown	
T2-L2	blue	Csa1G596520	3.4	0.0002165	Protein degradation	Cystein proteinases superfamily protein	2.00E-168
T2-L2	blue	Csa7G395800	3.415	0.001823	Miscellaneous	Glutathione S-transferase phi 8 (GSTF8)	3.00E-82
T2-L2	blue	Csa3G119700	3.575	0.00005376	Transcription factor	WRKY DNA-binding protein 53 (WRKY53)	1.00E-102
T2-L2	blue	Csa6G381850	3.831	0.001156	Transport	Amino acid permease 3 (AAP3)	2.00E-234
T2-L2	blue	Csa1G033160	4.495	0.006049	Miscellaneous	Glutathione S-transferase tau 9 (GSTU9)	3.00E-68
T2-L2	blue	Csa6G486960	4.496	0.000002502	Transcription factor	WRKY DNA-binding protein 51 (WRKY51)	1.00E-36
T2-L2	blue	Csa2G058620	5.345	0.003945	Abiotic stress	DNAJ heat shock N-terminal domain-containing protein	2.00E-137

Supplementary Table 1 (Continued)

T2-L2	blue	Csa1G574970	1.827	1.18E-05	Major CHO metabolism	Hexokinase 2 (HXK2)	7.00E-51
T2-L2	blue	Csa4G618520	1.837	0.0001765	Biodegradation of xenobiotic	UDP glycosyltransferase (UGT)	1.00E-132
T2-L2	blue	Csa2G003610	1.841	0.0003098	Lipid metabolism	Abnormal inflorescence meristem (AIM1)	3.00E-294
T2-L2	blue	Csa3G730930	1.847	0.00004243	Transport	Ammonium transporter 2 (AMT2)	8E-220
T2-L2	blue	Csa6G493850	1.86	1.50E-05	Not assigned	unknown	
T2-L2	blue	Csa3G159420	1.871	1.61E-03	Not assigned	unknown	
T2-L2	blue	Csa1G528610	1.874	0.00435	Lipid metabolism	Thylakoid membrane anion transporter 1 (ANTR1)	7.00E-236
T2-L2	blue	Csa1G181360	1.875	0.001947	Transport	non-intrinsic ABC protein 9 (NAP9)	3.00E-156
T2-L2	blue	Csa3G154410	2.05	1.63E-10	Not assigned	unknown	
T2-L2	blue	Csa3G183950	2.092	0.0004285	Abiotic stress	Heat shock protein 83	0.00E+00
T2-L2	blue	Csa2G296090	2.143	2.44E-04	Not assigned	unknown	
T2-L2	blue	Csa7G291720	2.169	0.006566	Lipid metabolism	Choline kinase	4.00E-133
T2-L2	blue	Csa2G427310	2.238	0.007581	Transcription factor	MYB domain protein 73 (MYB73)	1.00E-88
T2-L2	blue	Csa3G881750	2.24	0.009561	Lipid metabolism	PR5-like receptor kinase (PR5K)	4.00E-154
T2-L2	blue	Csa5G589260	2.243	0	Amino acid metabolism	O-acetylserine(thiol) lyase (OAS-TL)	4.00E-162
T2-L2	blue	Csa3G733350	2.317	0.00004435	Cell organization	RHO guanyl-nucleotide exchange factor 11 (ROPGEF11)	9.00E-41
T2-L2	blue	Csa2G004690	2.522	0.000004593	Transcription factor	Heat shock transcription factor C1 (HSFC1)	5.00E-67
T2-L2	blue	Csa7G387690	2.631	0.000001654	Transport	Sulfate transporter 3;1 (SULTR 3;1)	3.00E-307
T2-L2	blue	Csa1G008520	2.771	0.00804	Transport	Plasma membrane proton ATPase 4 (HA4)	0.00E+00
T2-L2	blue	Csa6G514850	3.048	0.00009803	Miscellaneous	Cytochrome P450 family 77 subfamily B polypeptide 1 (CYP77B1)	1.00E-241
T2-L2	blue	Csa5G604150	3.14	0.01	Redox	Thioredoxin 2 (TRX2)	
T2-L2	blue	Csa5G223070	3.216	0.003151	Transcription factor	WRKY DNA-binding protein 54 (WRKY54)	3.00E-96
T2-L2	blue	Csa2G359890	3.289	3.70E-03	Not assigned	unknown	
T2-L2	blue	Csa1G596520	3.4	0.0002165	Protein degradation	Cystein proteinases superfamily protein	2.00E-168
T2-L2	blue	Csa7G395800	3.415	0.001823	Miscellaneous	Glutathione S-transferase phi 8 (GSTF8)	3.00E-82
T2-L2	blue	Csa3G119700	3.575	0.00005376	Transcription factor	WRKY DNA-binding protein 53 (WRKY53)	1.00E-102
T2-L2	blue	Csa6G381850	3.831	0.001156	Transport	Amino acid permease 3 (AAP3)	2.00E-234
T2-L2	blue	Csa1G033160	4.495	0.006049	Miscellaneous	Glutathione S-transferase tau 9 (GSTU9)	3.00E-68
T2-L2	blue	Csa6G486960	4.496	0.000002502	Transcription factor	WRKY DNA-binding protein 51 (WRKY51)	1.00E-36
T2-L2	blue	Csa2G058620	5.345	0.003945	Abiotic stress	DNAJ heat shock N-terminal domain-containing protein	2.00E-137

Supplementary Table 1 (Continued)

T2-L2	blue	Csa1G574970	1.827	1.18E-05	Major CHO metabolism	Hexokinase 2 (HXK2)	7.00E-51
T2-L2	blue	Csa4G618520	1.837	0.0001765	Biodegradation of xenobiotic	UDP glycosyltransferase (UGT)	1.00E-132
T2-L2	blue	Csa2G003610	1.841	0.0003098	Lipid metabolism	Abnormal inflorescence meristem (AIM1)	3.00E-294
T2-L2	blue	Csa3G730930	1.847	0.00004243	Transport	Ammonium transporter 2 (AMT2)	8E-220
T2-L2	blue	Csa6G493850	1.86	1.50E-05	Not assigned	unknown	
T2-L2	blue	Csa3G159420	1.871	1.61E-03	Not assigned	unknown	
T2-L2	blue	Csa1G528610	1.874	0.00435	Lipid metabolism	Thylakoid membrane anion transporter 1 (ANTR1)	7.00E-236
T2-L2	blue	Csa1G181360	1.875	0.001947	Transport	non-intrinsic ABC protein 9 (NAP9)	3.00E-156
T2-L2	blue	Csa3G154410	2.05	1.63E-10	Not assigned	unknown	
T2-L2	blue	Csa3G183950	2.092	0.0004285	Abiotic stress	Heat shock protein 83	0.00E+00
T2-L2	blue	Csa2G296090	2.143	2.44E-04	Not assigned	unknown	
T2-L2	blue	Csa7G291720	2.169	0.006566	Lipid metabolism	Choline kinase	4.00E-133
T2-L2	blue	Csa2G427310	2.238	0.007581	Transcription factor	MYB domain protein 73 (MYB73)	1.00E-88
T2-L2	blue	Csa3G881750	2.24	0.009561	Lipid metabolism	PR5-like receptor kinase (PR5K)	4.00E-154
T2-L2	blue	Csa5G589260	2.243	0	Amino acid metabolism	O-acetylserine(thiol) lyase (OAS-TL)	4.00E-162
T2-L2	blue	Csa3G733350	2.317	0.00004435	Cell organization	RHO guanyl-nucleotide exchange factor 11 (ROPGEF11)	9.00E-41
T2-L2	blue	Csa2G004690	2.522	0.000004593	Transcription factor	Heat shock transcription factor C1 (HSFC1)	5.00E-67
T2-L2	blue	Csa7G387690	2.631	0.000001654	Transport	Sulfate transporter 3;1 (SULTR 3;1)	3.00E-307
T2-L2	blue	Csa1G008520	2.771	0.00804	Transport	Plasma membrane proton ATPase 4 (HA4)	0.00E+00
T2-L2	blue	Csa6G514850	3.048	0.00009803	Miscellaneous	Cytochrome P450 family 77 subfamily B polypeptide 1 (CYP77B1)	1.00E-241
T2-L2	blue	Csa5G604150	3.14	0.01	Redox	Thioredoxin 2 (TRX2)	
T2-L2	blue	Csa5G223070	3.216	0.003151	Transcription factor	WRKY DNA-binding protein 54 (WRKY54)	3.00E-96
T2-L2	blue	Csa2G359890	3.289	3.70E-03	Not assigned	unknown	
T2-L2	blue	Csa1G596520	3.4	0.0002165	Protein degradation	Cystein proteinases superfamily protein	2.00E-168
T2-L2	blue	Csa7G395800	3.415	0.001823	Miscellaneous	Glutathione S-transferase phi 8 (GSTF8)	3.00E-82
T2-L2	blue	Csa3G119700	3.575	0.00005376	Transcription factor	WRKY DNA-binding protein 53 (WRKY53)	1.00E-102
T2-L2	blue	Csa6G381850	3.831	0.001156	Transport	Amino acid permease 3 (AAP3)	2.00E-234
T2-L2	blue	Csa1G033160	4.495	0.006049	Miscellaneous	Glutathione S-transferase tau 9 (GSTU9)	3.00E-68
T2-L2	blue	Csa6G486960	4.496	0.000002502	Transcription factor	WRKY DNA-binding protein 51 (WRKY51)	1.00E-36
T2-L2	blue	Csa2G058620	5.345	0.003945	Abiotic stress	DNAJ heat shock N-terminal domain-containing protein	2.00E-137

Supplementary Table 1 (Continued)

T2-L2	blue	Csa1G574970	1.827	1.18E-05	Major CHO metabolism	Hexokinase 2 (HXK2)	7.00E-51
T2-L2	blue	Csa4G618520	1.837	0.0001765	Biodegradation of xenobiotic	UDP glycosyltransferase (UGT)	1.00E-132
T2-L2	blue	Csa2G003610	1.841	0.0003098	Lipid metabolism	Abnormal inflorescence meristem (AIM1)	3.00E-294
T2-L2	blue	Csa3G730930	1.847	0.00004243	Transport	Ammonium transporter 2 (AMT2)	8E-220
T2-L2	blue	Csa6G493850	1.86	1.50E-05	Not assigned	unknown	
T2-L2	blue	Csa3G159420	1.871	1.61E-03	Not assigned	unknown	
T2-L2	blue	Csa1G528610	1.874	0.00435	Lipid metabolism	Thylakoid membrane anion transporter 1 (ANTR1)	7.00E-236
T2-L2	blue	Csa1G181360	1.875	0.001947	Transport	non-intrinsic ABC protein 9 (NAP9)	3.00E-156
T2-L2	blue	Csa3G154410	2.05	1.63E-10	Not assigned	unknown	
T2-L2	blue	Csa3G183950	2.092	0.0004285	Abiotic stress	Heat shock protein 83	0.00E+00
T2-L2	blue	Csa2G296090	2.143	2.44E-04	Not assigned	unknown	
T2-L2	blue	Csa7G291720	2.169	0.006566	Lipid metabolism	Choline kinase	4.00E-133
T2-L2	blue	Csa2G427310	2.238	0.007581	Transcription factor	MYB domain protein 73 (MYB73)	1.00E-88
T2-L2	blue	Csa3G881750	2.24	0.009561	Lipid metabolism	PR5-like receptor kinase (PR5K)	4.00E-154
T2-L2	blue	Csa5G589260	2.243	0	Amino acid metabolism	O-acetylserine(thiol) lyase (OAS-TL)	4.00E-162
T2-L2	blue	Csa3G733350	2.317	0.00004435	Cell organization	RHO guanyl-nucleotide exchange factor 11 (ROPGEF11)	9.00E-41
T2-L2	blue	Csa2G004690	2.522	0.000004593	Transcription factor	Heat shock transcription factor C1 (HSFC1)	5.00E-67
T2-L2	blue	Csa7G387690	2.631	0.000001654	Transport	Sulfate transporter 3;1 (SULTR 3;1)	3.00E-307
T2-L2	blue	Csa1G008520	2.771	0.00804	Transport	Plasma membrane proton ATPase 4 (HA4)	0.00E+00
T2-L2	blue	Csa6G514850	3.048	0.00009803	Miscellaneous	Cytochrome P450 family 77 subfamily B polypeptide 1 (CYP77B1)	1.00E-241
T2-L2	blue	Csa5G604150	3.14	0.01	Redox	Thioredoxin 2 (TRX2)	
T2-L2	blue	Csa5G223070	3.216	0.003151	Transcription factor	WRKY DNA-binding protein 54 (WRKY54)	3.00E-96
T2-L2	blue	Csa2G359890	3.289	3.70E-03	Not assigned	unknown	
T2-L2	blue	Csa1G596520	3.4	0.0002165	Protein degradation	Cystein proteinases superfamily protein	2.00E-168
T2-L2	blue	Csa7G395800	3.415	0.001823	Miscellaneous	Glutathione S-transferase phi 8 (GSTF8)	3.00E-82
T2-L2	blue	Csa3G119700	3.575	0.00005376	Transcription factor	WRKY DNA-binding protein 53 (WRKY53)	1.00E-102
T2-L2	blue	Csa6G381850	3.831	0.001156	Transport	Amino acid permease 3 (AAP3)	2.00E-234
T2-L2	blue	Csa1G033160	4.495	0.006049	Miscellaneous	Glutathione S-transferase tau 9 (GSTU9)	3.00E-68
T2-L2	blue	Csa6G486960	4.496	0.000002502	Transcription factor	WRKY DNA-binding protein 51 (WRKY51)	1.00E-36
T2-L2	blue	Csa2G058620	5.345	0.003945	Abiotic stress	DNAJ heat shock N-terminal domain-containing protein	2.00E-137

Supplementary Table 1 (Continued)

T2-L2	blue	Csa1G574970	1.827	1.18E-05	Major CHO metabolism	Hexokinase 2 (HXK2)	7.00E-51
T2-L2	blue	Csa4G618520	1.837	0.0001765	Biodegradation of xenobiotic	UDP glycosyltransferase (UGT)	1.00E-132
T2-L2	blue	Csa2G003610	1.841	0.0003098	Lipid metabolism	Abnormal inflorescence meristem (AIM1)	3.00E-294
T2-L2	blue	Csa3G730930	1.847	0.00004243	Transport	Ammonium transporter 2 (AMT2)	8E-220
T2-L2	blue	Csa6G493850	1.86	1.50E-05	Not assigned	unknown	
T2-L2	blue	Csa3G159420	1.871	1.61E-03	Not assigned	unknown	
T2-L2	blue	Csa1G528610	1.874	0.00435	Lipid metabolism	Thylakoid membrane anion transporter 1 (ANTR1)	7.00E-236
T2-L2	blue	Csa1G181360	1.875	0.001947	Transport	non-intrinsic ABC protein 9 (NAP9)	3.00E-156
T2-L2	blue	Csa3G154410	2.05	1.63E-10	Not assigned	unknown	
T2-L2	blue	Csa3G183950	2.092	0.0004285	Abiotic stress	Heat shock protein 83	0.00E+00
T2-L2	blue	Csa2G296090	2.143	2.44E-04	Not assigned	unknown	
T2-L2	blue	Csa7G291720	2.169	0.006566	Lipid metabolism	Choline kinase	4.00E-133
T2-L2	blue	Csa2G427310	2.238	0.007581	Transcription factor	MYB domain protein 73 (MYB73)	1.00E-88
T2-L2	blue	Csa3G881750	2.24	0.009561	Lipid metabolism	PR5-like receptor kinase (PR5K)	4.00E-154
T2-L2	blue	Csa5G589260	2.243	0	Amino acid metabolism	O-acetylserine(thiol) lyase (OAS-TL)	4.00E-162
T2-L2	blue	Csa3G733350	2.317	0.00004435	Cell organization	RHO guanyl-nucleotide exchange factor 11 (ROPGEF11)	9.00E-41
T2-L2	blue	Csa2G004690	2.522	0.000004593	Transcription factor	Heat shock transcription factor C1 (HSFC1)	5.00E-67
T2-L2	blue	Csa7G387690	2.631	0.000001654	Transport	Sulfate transporter 3;1 (SULTR 3;1)	3.00E-307
T2-L2	blue	Csa1G008520	2.771	0.00804	Transport	Plasma membrane proton ATPase 4 (HA4)	0.00E+00
T2-L2	blue	Csa6G514850	3.048	0.00009803	Miscellaneous	Cytochrome P450 family 77 subfamily B polypeptide 1 (CYP77B1)	1.00E-241
T2-L2	blue	Csa5G604150	3.14	0.01	Redox	Thioredoxin 2 (TRX2)	
T2-L2	blue	Csa5G223070	3.216	0.003151	Transcription factor	WRKY DNA-binding protein 54 (WRKY54)	3.00E-96
T2-L2	blue	Csa2G359890	3.289	3.70E-03	Not assigned	unknown	
T2-L2	blue	Csa1G596520	3.4	0.0002165	Protein degradation	Cystein proteinases superfamily protein	2.00E-168
T2-L2	blue	Csa7G395800	3.415	0.001823	Miscellaneous	Glutathione S-transferase phi 8 (GSTF8)	3.00E-82
T2-L2	blue	Csa3G119700	3.575	0.00005376	Transcription factor	WRKY DNA-binding protein 53 (WRKY53)	1.00E-102
T2-L2	blue	Csa6G381850	3.831	0.001156	Transport	Amino acid permease 3 (AAP3)	2.00E-234
T2-L2	blue	Csa1G033160	4.495	0.006049	Miscellaneous	Glutathione S-transferase tau 9 (GSTU9)	3.00E-68
T2-L2	blue	Csa6G486960	4.496	0.000002502	Transcription factor	WRKY DNA-binding protein 51 (WRKY51)	1.00E-36
T2-L2	blue	Csa2G058620	5.345	0.003945	Abiotic stress	DNAJ heat shock N-terminal domain-containing protein	2.00E-137

Supplementary Table 1 (Continued)

T2-L2	blue	Csa1G574970	1.827	1.18E-05	Major CHO metabolism	Hexokinase 2 (HXK2)	7.00E-51
T2-L2	blue	Csa4G618520	1.837	0.0001765	Biodegradation of xenobiotic	UDP glycosyltransferase (UGT)	1.00E-132
T2-L2	blue	Csa2G003610	1.841	0.0003098	Lipid metabolism	Abnormal inflorescence meristem (AIM1)	3.00E-294
T2-L2	blue	Csa3G730930	1.847	0.00004243	Transport	Ammonium transporter 2 (AMT2)	8E-220
T2-L2	blue	Csa6G493850	1.86	1.50E-05	Not assigned	unknown	
T2-L2	blue	Csa3G159420	1.871	1.61E-03	Not assigned	unknown	
T2-L2	blue	Csa1G528610	1.874	0.00435	Lipid metabolism	Thylakoid membrane anion transporter 1 (ANTR1)	7.00E-236
T2-L2	blue	Csa1G181360	1.875	0.001947	Transport	non-intrinsic ABC protein 9 (NAP9)	3.00E-156
T2-L2	blue	Csa3G154410	2.05	1.63E-10	Not assigned	unknown	
T2-L2	blue	Csa3G183950	2.092	0.0004285	Abiotic stress	Heat shock protein 83	0.00E+00
T2-L2	blue	Csa2G296090	2.143	2.44E-04	Not assigned	unknown	
T2-L2	blue	Csa7G291720	2.169	0.006566	Lipid metabolism	Choline kinase	4.00E-133
T2-L2	blue	Csa2G427310	2.238	0.007581	Transcription factor	MYB domain protein 73 (MYB73)	1.00E-88
T2-L2	blue	Csa3G881750	2.24	0.009561	Lipid metabolism	PR5-like receptor kinase (PR5K)	4.00E-154
T2-L2	blue	Csa5G589260	2.243	0	Amino acid metabolism	O-acetylserine(thiol) lyase (OAS-TL)	4.00E-162
T2-L2	blue	Csa3G733350	2.317	0.00004435	Cell organization	RHO guanyl-nucleotide exchange factor 11 (ROPGEF11)	9.00E-41
T2-L2	blue	Csa2G004690	2.522	0.000004593	Transcription factor	Heat shock transcription factor C1 (HSFC1)	5.00E-67
T2-L2	blue	Csa7G387690	2.631	0.000001654	Transport	Sulfate transporter 3;1 (SULTR 3;1)	3.00E-307
T2-L2	blue	Csa1G008520	2.771	0.00804	Transport	Plasma membrane proton ATPase 4 (HA4)	0.00E+00
T2-L2	blue	Csa6G514850	3.048	0.00009803	Miscellaneous	Cytochrome P450 family 77 subfamily B polypeptide 1 (CYP77B1)	1.00E-241
T2-L2	blue	Csa5G604150	3.14	0.01	Redox	Thioredoxin 2 (TRX2)	
T2-L2	blue	Csa5G223070	3.216	0.003151	Transcription factor	WRKY DNA-binding protein 54 (WRKY54)	3.00E-96
T2-L2	blue	Csa2G359890	3.289	3.70E-03	Not assigned	unknown	
T2-L2	blue	Csa1G596520	3.4	0.0002165	Protein degradation	Cystein proteinases superfamily protein	2.00E-168
T2-L2	blue	Csa7G395800	3.415	0.001823	Miscellaneous	Glutathione S-transferase phi 8 (GSTF8)	3.00E-82
T2-L2	blue	Csa3G119700	3.575	0.00005376	Transcription factor	WRKY DNA-binding protein 53 (WRKY53)	1.00E-102
T2-L2	blue	Csa6G381850	3.831	0.001156	Transport	Amino acid permease 3 (AAP3)	2.00E-234
T2-L2	blue	Csa1G033160	4.495	0.006049	Miscellaneous	Glutathione S-transferase tau 9 (GSTU9)	3.00E-68
T2-L2	blue	Csa6G486960	4.496	0.000002502	Transcription factor	WRKY DNA-binding protein 51 (WRKY51)	1.00E-36
T2-L2	blue	Csa2G058620	5.345	0.003945	Abiotic stress	DNAJ heat shock N-terminal domain-containing protein	2.00E-137

Supplementary Table 1 (Continued)

T2-L2	blue	Csa1G574970	1.827	1.18E-05	Major CHO metabolism	Hexokinase 2 (HXK2)	7.00E-51
T2-L2	blue	Csa4G618520	1.837	0.0001765	Biodegradation of xenobiotic	UDP glycosyltransferase (UGT)	1.00E-132
T2-L2	blue	Csa2G003610	1.841	0.0003098	Lipid metabolism	Abnormal inflorescence meristem (AIM1)	3.00E-294
T2-L2	blue	Csa3G730930	1.847	0.00004243	Transport	Ammonium transporter 2 (AMT2)	8E-220
T2-L2	blue	Csa6G493850	1.86	1.50E-05	Not assigned	unknown	
T2-L2	blue	Csa3G159420	1.871	1.61E-03	Not assigned	unknown	
T2-L2	blue	Csa1G528610	1.874	0.00435	Lipid metabolism	Thylakoid membrane anion transporter 1 (ANTR1)	7.00E-236
T2-L2	blue	Csa1G181360	1.875	0.001947	Transport	non-intrinsic ABC protein 9 (NAP9)	3.00E-156
T2-L2	blue	Csa3G154410	2.05	1.63E-10	Not assigned	unknown	
T2-L2	blue	Csa3G183950	2.092	0.0004285	Abiotic stress	Heat shock protein 83	0.00E+00
T2-L2	blue	Csa2G296090	2.143	2.44E-04	Not assigned	unknown	
T2-L2	blue	Csa7G291720	2.169	0.006566	Lipid metabolism	Choline kinase	4.00E-133
T2-L2	blue	Csa2G427310	2.238	0.007581	Transcription factor	MYB domain protein 73 (MYB73)	1.00E-88
T2-L2	blue	Csa3G881750	2.24	0.009561	Lipid metabolism	PR5-like receptor kinase (PR5K)	4.00E-154
T2-L2	blue	Csa5G589260	2.243	0	Amino acid metabolism	O-acetylserine(thiol) lyase (OAS-TL)	4.00E-162
T2-L2	blue	Csa3G733350	2.317	0.00004435	Cell organization	RHO guanyl-nucleotide exchange factor 11 (ROPGEF11)	9.00E-41
T2-L2	blue	Csa2G004690	2.522	0.000004593	Transcription factor	Heat shock transcription factor C1 (HSFC1)	5.00E-67
T2-L2	blue	Csa7G387690	2.631	0.000001654	Transport	Sulfate transporter 3;1 (SULTR 3;1)	3.00E-307
T2-L2	blue	Csa1G008520	2.771	0.00804	Transport	Plasma membrane proton ATPase 4 (HA4)	0.00E+00
T2-L2	blue	Csa6G514850	3.048	0.00009803	Miscellaneous	Cytochrome P450 family 77 subfamily B polypeptide 1 (CYP77B1)	1.00E-241
T2-L2	blue	Csa5G604150	3.14	0.01	Redox	Thioredoxin 2 (TRX2)	
T2-L2	blue	Csa5G223070	3.216	0.003151	Transcription factor	WRKY DNA-binding protein 54 (WRKY54)	3.00E-96
T2-L2	blue	Csa2G359890	3.289	3.70E-03	Not assigned	unknown	
T2-L2	blue	Csa1G596520	3.4	0.0002165	Protein degradation	Cystein proteinases superfamily protein	2.00E-168
T2-L2	blue	Csa7G395800	3.415	0.001823	Miscellaneous	Glutathione S-transferase phi 8 (GSTF8)	3.00E-82
T2-L2	blue	Csa3G119700	3.575	0.00005376	Transcription factor	WRKY DNA-binding protein 53 (WRKY53)	1.00E-102
T2-L2	blue	Csa6G381850	3.831	0.001156	Transport	Amino acid permease 3 (AAP3)	2.00E-234
T2-L2	blue	Csa1G033160	4.495	0.006049	Miscellaneous	Glutathione S-transferase tau 9 (GSTU9)	3.00E-68
T2-L2	blue	Csa6G486960	4.496	0.000002502	Transcription factor	WRKY DNA-binding protein 51 (WRKY51)	1.00E-36
T2-L2	blue	Csa2G058620	5.345	0.003945	Abiotic stress	DNAJ heat shock N-terminal domain-containing protein	2.00E-137

Supplementary Table 1 (Continued)

T2-L2	blue	Csa1G574970	1.827	1.18E-05	Major CHO metabolism	Hexokinase 2 (HXK2)	7.00E-51
T2-L2	blue	Csa4G618520	1.837	0.0001765	Biodegradation of xenobiotic	UDP glycosyltransferase (UGT)	1.00E-132
T2-L2	blue	Csa2G003610	1.841	0.0003098	Lipid metabolism	Abnormal inflorescence meristem (AIM1)	3.00E-294
T2-L2	blue	Csa3G730930	1.847	0.00004243	Transport	Ammonium transporter 2 (AMT2)	8E-220
T2-L2	blue	Csa6G493850	1.86	1.50E-05	Not assigned	unknown	
T2-L2	blue	Csa3G159420	1.871	1.61E-03	Not assigned	unknown	
T2-L2	blue	Csa1G528610	1.874	0.00435	Lipid metabolism	Thylakoid membrane anion transporter 1 (ANTR1)	7.00E-236
T2-L2	blue	Csa1G181360	1.875	0.001947	Transport	non-intrinsic ABC protein 9 (NAP9)	3.00E-156
T2-L2	blue	Csa3G154410	2.05	1.63E-10	Not assigned	unknown	
T2-L2	blue	Csa3G183950	2.092	0.0004285	Abiotic stress	Heat shock protein 83	0.00E+00
T2-L2	blue	Csa2G296090	2.143	2.44E-04	Not assigned	unknown	
T2-L2	blue	Csa7G291720	2.169	0.006566	Lipid metabolism	Choline kinase	4.00E-133
T2-L2	blue	Csa2G427310	2.238	0.007581	Transcription factor	MYB domain protein 73 (MYB73)	1.00E-88
T2-L2	blue	Csa3G881750	2.24	0.009561	Lipid metabolism	PR5-like receptor kinase (PR5K)	4.00E-154
T2-L2	blue	Csa5G589260	2.243	0	Amino acid metabolism	O-acetylserine(thiol) lyase (OAS-TL)	4.00E-162
T2-L2	blue	Csa3G733350	2.317	0.00004435	Cell organization	RHO guanyl-nucleotide exchange factor 11 (ROPGEF11)	9.00E-41
T2-L2	blue	Csa2G004690	2.522	0.000004593	Transcription factor	Heat shock transcription factor C1 (HSFC1)	5.00E-67
T2-L2	blue	Csa7G387690	2.631	0.000001654	Transport	Sulfate transporter 3;1 (SULTR 3;1)	3.00E-307
T2-L2	blue	Csa1G008520	2.771	0.00804	Transport	Plasma membrane proton ATPase 4 (HA4)	0.00E+00
T2-L2	blue	Csa6G514850	3.048	0.00009803	Miscellaneous	Cytochrome P450 family 77 subfamily B polypeptide 1 (CYP77B1)	1.00E-241
T2-L2	blue	Csa5G604150	3.14	0.01	Redox	Thioredoxin 2 (TRX2)	
T2-L2	blue	Csa5G223070	3.216	0.003151	Transcription factor	WRKY DNA-binding protein 54 (WRKY54)	3.00E-96
T2-L2	blue	Csa2G359890	3.289	3.70E-03	Not assigned	unknown	
T2-L2	blue	Csa1G596520	3.4	0.0002165	Protein degradation	Cystein proteinases superfamily protein	2.00E-168
T2-L2	blue	Csa7G395800	3.415	0.001823	Miscellaneous	Glutathione S-transferase phi 8 (GSTF8)	3.00E-82
T2-L2	blue	Csa3G119700	3.575	0.00005376	Transcription factor	WRKY DNA-binding protein 53 (WRKY53)	1.00E-102
T2-L2	blue	Csa6G381850	3.831	0.001156	Transport	Amino acid permease 3 (AAP3)	2.00E-234
T2-L2	blue	Csa1G033160	4.495	0.006049	Miscellaneous	Glutathione S-transferase tau 9 (GSTU9)	3.00E-68
T2-L2	blue	Csa6G486960	4.496	0.000002502	Transcription factor	WRKY DNA-binding protein 51 (WRKY51)	1.00E-36
T2-L2	blue	Csa2G058620	5.345	0.003945	Abiotic stress	DNAJ heat shock N-terminal domain-containing protein	2.00E-137

Supplementary Table 1 (Continued)

T2-L2	blue	Csa1G574970	1.827	1.18E-05	Major CHO metabolism	Hexokinase 2 (HXK2)	7.00E-51
T2-L2	blue	Csa4G618520	1.837	0.0001765	Biodegradation of xenobiotic	UDP glycosyltransferase (UGT)	1.00E-132
T2-L2	blue	Csa2G003610	1.841	0.0003098	Lipid metabolism	Abnormal inflorescence meristem (AIM1)	3.00E-294
T2-L2	blue	Csa3G730930	1.847	0.00004243	Transport	Ammonium transporter 2 (AMT2)	8E-220
T2-L2	blue	Csa6G493850	1.86	1.50E-05	Not assigned	unknown	
T2-L2	blue	Csa3G159420	1.871	1.61E-03	Not assigned	unknown	
T2-L2	blue	Csa1G528610	1.874	0.00435	Lipid metabolism	Thylakoid membrane anion transporter 1 (ANTR1)	7.00E-236
T2-L2	blue	Csa1G181360	1.875	0.001947	Transport	non-intrinsic ABC protein 9 (NAP9)	3.00E-156
T2-L2	blue	Csa3G154410	2.05	1.63E-10	Not assigned	unknown	
T2-L2	blue	Csa3G183950	2.092	0.0004285	Abiotic stress	Heat shock protein 83	0.00E+00
T2-L2	blue	Csa2G296090	2.143	2.44E-04	Not assigned	unknown	
T2-L2	blue	Csa7G291720	2.169	0.006566	Lipid metabolism	Choline kinase	4.00E-133
T2-L2	blue	Csa2G427310	2.238	0.007581	Transcription factor	MYB domain protein 73 (MYB73)	1.00E-88
T2-L2	blue	Csa3G881750	2.24	0.009561	Lipid metabolism	PR5-like receptor kinase (PR5K)	4.00E-154
T2-L2	blue	Csa5G589260	2.243	0	Amino acid metabolism	O-acetylserine(thiol) lyase (OAS-TL)	4.00E-162
T2-L2	blue	Csa3G733350	2.317	0.00004435	Cell organization	RHO guanyl-nucleotide exchange factor 11 (ROPGEF11)	9.00E-41
T2-L2	blue	Csa2G004690	2.522	0.000004593	Transcription factor	Heat shock transcription factor C1 (HSFC1)	5.00E-67
T2-L2	blue	Csa7G387690	2.631	0.000001654	Transport	Sulfate transporter 3;1 (SULTR 3;1)	3.00E-307
T2-L2	blue	Csa1G008520	2.771	0.00804	Transport	Plasma membrane proton ATPase 4 (HA4)	0.00E+00
T2-L2	blue	Csa6G514850	3.048	0.00009803	Miscellaneous	Cytochrome P450 family 77 subfamily B polypeptide 1 (CYP77B1)	1.00E-241
T2-L2	blue	Csa5G604150	3.14	0.01	Redox	Thioredoxin 2 (TRX2)	
T2-L2	blue	Csa5G223070	3.216	0.003151	Transcription factor	WRKY DNA-binding protein 54 (WRKY54)	3.00E-96
T2-L2	blue	Csa2G359890	3.289	3.70E-03	Not assigned	unknown	
T2-L2	blue	Csa1G596520	3.4	0.0002165	Protein degradation	Cystein proteinases superfamily protein	2.00E-168
T2-L2	blue	Csa7G395800	3.415	0.001823	Miscellaneous	Glutathione S-transferase phi 8 (GSTF8)	3.00E-82
T2-L2	blue	Csa3G119700	3.575	0.00005376	Transcription factor	WRKY DNA-binding protein 53 (WRKY53)	1.00E-102
T2-L2	blue	Csa6G381850	3.831	0.001156	Transport	Amino acid permease 3 (AAP3)	2.00E-234
T2-L2	blue	Csa1G033160	4.495	0.006049	Miscellaneous	Glutathione S-transferase tau 9 (GSTU9)	3.00E-68
T2-L2	blue	Csa6G486960	4.496	0.000002502	Transcription factor	WRKY DNA-binding protein 51 (WRKY51)	1.00E-36
T2-L2	blue	Csa2G058620	5.345	0.003945	Abiotic stress	DNAJ heat shock N-terminal domain-containing protein	2.00E-137

Supplementary Table 1 (Continued)

T2-L2	blue	Csa1G574970	1.827	1.18E-05	Major CHO metabolism	Hexokinase 2 (HXK2)	7.00E-51
T2-L2	blue	Csa4G618520	1.837	0.0001765	Biodegradation of xenobiotic	UDP glycosyltransferase (UGT)	1.00E-132
T2-L2	blue	Csa2G003610	1.841	0.0003098	Lipid metabolism	Abnormal inflorescence meristem (AIM1)	3.00E-294
T2-L2	blue	Csa3G730930	1.847	0.00004243	Transport	Ammonium transporter 2 (AMT2)	8E-220
T2-L2	blue	Csa6G493850	1.86	1.50E-05	Not assigned	unknown	
T2-L2	blue	Csa3G159420	1.871	1.61E-03	Not assigned	unknown	
T2-L2	blue	Csa1G528610	1.874	0.00435	Lipid metabolism	Thylakoid membrane anion transporter 1 (ANTR1)	7.00E-236
T2-L2	blue	Csa1G181360	1.875	0.001947	Transport	non-intrinsic ABC protein 9 (NAP9)	3.00E-156
T2-L2	blue	Csa3G154410	2.05	1.63E-10	Not assigned	unknown	
T2-L2	blue	Csa3G183950	2.092	0.0004285	Abiotic stress	Heat shock protein 83	0.00E+00
T2-L2	blue	Csa2G296090	2.143	2.44E-04	Not assigned	unknown	
T2-L2	blue	Csa7G291720	2.169	0.006566	Lipid metabolism	Choline kinase	4.00E-133
T2-L2	blue	Csa2G427310	2.238	0.007581	Transcription factor	MYB domain protein 73 (MYB73)	1.00E-88
T2-L2	blue	Csa3G881750	2.24	0.009561	Lipid metabolism	PR5-like receptor kinase (PR5K)	4.00E-154
T2-L2	blue	Csa5G589260	2.243	0	Amino acid metabolism	O-acetylserine(thiol) lyase (OAS-TL)	4.00E-162
T2-L2	blue	Csa3G733350	2.317	0.00004435	Cell organization	RHO guanyl-nucleotide exchange factor 11 (ROPGEF11)	9.00E-41
T2-L2	blue	Csa2G004690	2.522	0.000004593	Transcription factor	Heat shock transcription factor C1 (HSFC1)	5.00E-67
T2-L2	blue	Csa7G387690	2.631	0.000001654	Transport	Sulfate transporter 3;1 (SULTR 3;1)	3.00E-307
T2-L2	blue	Csa1G008520	2.771	0.00804	Transport	Plasma membrane proton ATPase 4 (HA4)	0.00E+00
T2-L2	blue	Csa6G514850	3.048	0.00009803	Miscellaneous	Cytochrome P450 family 77 subfamily B polypeptide 1 (CYP77B1)	1.00E-241
T2-L2	blue	Csa5G604150	3.14	0.01	Redox	Thioredoxin 2 (TRX2)	
T2-L2	blue	Csa5G223070	3.216	0.003151	Transcription factor	WRKY DNA-binding protein 54 (WRKY54)	3.00E-96
T2-L2	blue	Csa2G359890	3.289	3.70E-03	Not assigned	unknown	
T2-L2	blue	Csa1G596520	3.4	0.0002165	Protein degradation	Cystein proteinases superfamily protein	2.00E-168
T2-L2	blue	Csa7G395800	3.415	0.001823	Miscellaneous	Glutathione S-transferase phi 8 (GSTF8)	3.00E-82
T2-L2	blue	Csa3G119700	3.575	0.00005376	Transcription factor	WRKY DNA-binding protein 53 (WRKY53)	1.00E-102
T2-L2	blue	Csa6G381850	3.831	0.001156	Transport	Amino acid permease 3 (AAP3)	2.00E-234
T2-L2	blue	Csa1G033160	4.495	0.006049	Miscellaneous	Glutathione S-transferase tau 9 (GSTU9)	3.00E-68
T2-L2	blue	Csa6G486960	4.496	0.000002502	Transcription factor	WRKY DNA-binding protein 51 (WRKY51)	1.00E-36
T2-L2	blue	Csa2G058620	5.345	0.003945	Abiotic stress	DNAJ heat shock N-terminal domain-containing protein	2.00E-137

Supplementary Table 1 (Continued)

T2-L2	blue	Csa1G574970	1.827	1.18E-05	Major CHO metabolism	Hexokinase 2 (HXK2)	7.00E-51
T2-L2	blue	Csa4G618520	1.837	0.0001765	Biodegradation of xenobiotic	UDP glycosyltransferase (UGT)	1.00E-132
T2-L2	blue	Csa2G003610	1.841	0.0003098	Lipid metabolism	Abnormal inflorescence meristem (AIM1)	3.00E-294
T2-L2	blue	Csa3G730930	1.847	0.00004243	Transport	Ammonium transporter 2 (AMT2)	8E-220
T2-L2	blue	Csa6G493850	1.86	1.50E-05	Not assigned	unknown	
T2-L2	blue	Csa3G159420	1.871	1.61E-03	Not assigned	unknown	
T2-L2	blue	Csa1G528610	1.874	0.00435	Lipid metabolism	Thylakoid membrane anion transporter 1 (ANTR1)	7.00E-236
T2-L2	blue	Csa1G181360	1.875	0.001947	Transport	non-intrinsic ABC protein 9 (NAP9)	3.00E-156
T2-L2	blue	Csa3G154410	2.05	1.63E-10	Not assigned	unknown	
T2-L2	blue	Csa3G183950	2.092	0.0004285	Abiotic stress	Heat shock protein 83	0.00E+00
T2-L2	blue	Csa2G296090	2.143	2.44E-04	Not assigned	unknown	
T2-L2	blue	Csa7G291720	2.169	0.006566	Lipid metabolism	Choline kinase	4.00E-133
T2-L2	blue	Csa2G427310	2.238	0.007581	Transcription factor	MYB domain protein 73 (MYB73)	1.00E-88
T2-L2	blue	Csa3G881750	2.24	0.009561	Lipid metabolism	PR5-like receptor kinase (PR5K)	4.00E-154
T2-L2	blue	Csa5G589260	2.243	0	Amino acid metabolism	O-acetylserine(thiol) lyase (OAS-TL)	4.00E-162
T2-L2	blue	Csa3G733350	2.317	0.00004435	Cell organization	RHO guanyl-nucleotide exchange factor 11 (ROPGEF11)	9.00E-41
T2-L2	blue	Csa2G004690	2.522	0.000004593	Transcription factor	Heat shock transcription factor C1 (HSFC1)	5.00E-67
T2-L2	blue	Csa7G387690	2.631	0.000001654	Transport	Sulfate transporter 3;1 (SULTR 3;1)	3.00E-307
T2-L2	blue	Csa1G008520	2.771	0.00804	Transport	Plasma membrane proton ATPase 4 (HA4)	0.00E+00
T2-L2	blue	Csa6G514850	3.048	0.00009803	Miscellaneous	Cytochrome P450 family 77 subfamily B polypeptide 1 (CYP77B1)	1.00E-241
T2-L2	blue	Csa5G604150	3.14	0.01	Redox	Thioredoxin 2 (TRX2)	
T2-L2	blue	Csa5G223070	3.216	0.003151	Transcription factor	WRKY DNA-binding protein 54 (WRKY54)	3.00E-96
T2-L2	blue	Csa2G359890	3.289	3.70E-03	Not assigned	unknown	
T2-L2	blue	Csa1G596520	3.4	0.0002165	Protein degradation	Cystein proteinases superfamily protein	2.00E-168
T2-L2	blue	Csa7G395800	3.415	0.001823	Miscellaneous	Glutathione S-transferase phi 8 (GSTF8)	3.00E-82
T2-L2	blue	Csa3G119700	3.575	0.00005376	Transcription factor	WRKY DNA-binding protein 53 (WRKY53)	1.00E-102
T2-L2	blue	Csa6G381850	3.831	0.001156	Transport	Amino acid permease 3 (AAP3)	2.00E-234
T2-L2	blue	Csa1G033160	4.495	0.006049	Miscellaneous	Glutathione S-transferase tau 9 (GSTU9)	3.00E-68
T2-L2	blue	Csa6G486960	4.496	0.000002502	Transcription factor	WRKY DNA-binding protein 51 (WRKY51)	1.00E-36
T2-L2	blue	Csa2G058620	5.345	0.003945	Abiotic stress	DNAJ heat shock N-terminal domain-containing protein	2.00E-137

Supplementary Table 1 (Continued)

T2-L2	blue	Csa1G574970	1.827	1.18E-05	Major CHO metabolism	Hexokinase 2 (HXK2)	7.00E-51
T2-L2	blue	Csa4G618520	1.837	0.0001765	Biodegradation of xenobiotic	UDP glycosyltransferase (UGT)	1.00E-132
T2-L2	blue	Csa2G003610	1.841	0.0003098	Lipid metabolism	Abnormal inflorescence meristem (AIM1)	3.00E-294
T2-L2	blue	Csa3G730930	1.847	0.00004243	Transport	Ammonium transporter 2 (AMT2)	8E-220
T2-L2	blue	Csa6G493850	1.86	1.50E-05	Not assigned	unknown	
T2-L2	blue	Csa3G159420	1.871	1.61E-03	Not assigned	unknown	
T2-L2	blue	Csa1G528610	1.874	0.00435	Lipid metabolism	Thylakoid membrane anion transporter 1 (ANTR1)	7.00E-236
T2-L2	blue	Csa1G181360	1.875	0.001947	Transport	non-intrinsic ABC protein 9 (NAP9)	3.00E-156
T2-L2	blue	Csa3G154410	2.05	1.63E-10	Not assigned	unknown	
T2-L2	blue	Csa3G183950	2.092	0.0004285	Abiotic stress	Heat shock protein 83	0.00E+00
T2-L2	blue	Csa2G296090	2.143	2.44E-04	Not assigned	unknown	
T2-L2	blue	Csa7G291720	2.169	0.006566	Lipid metabolism	Choline kinase	4.00E-133
T2-L2	blue	Csa2G427310	2.238	0.007581	Transcription factor	MYB domain protein 73 (MYB73)	1.00E-88
T2-L2	blue	Csa3G881750	2.24	0.009561	Lipid metabolism	PR5-like receptor kinase (PR5K)	4.00E-154
T2-L2	blue	Csa5G589260	2.243	0	Amino acid metabolism	O-acetylserine(thiol) lyase (OAS-TL)	4.00E-162
T2-L2	blue	Csa3G733350	2.317	0.00004435	Cell organization	RHO guanyl-nucleotide exchange factor 11 (ROPGEF11)	9.00E-41
T2-L2	blue	Csa2G004690	2.522	0.000004593	Transcription factor	Heat shock transcription factor C1 (HSFC1)	5.00E-67
T2-L2	blue	Csa7G387690	2.631	0.000001654	Transport	Sulfate transporter 3;1 (SULTR 3;1)	3.00E-307
T2-L2	blue	Csa1G008520	2.771	0.00804	Transport	Plasma membrane proton ATPase 4 (HA4)	0.00E+00
T2-L2	blue	Csa6G514850	3.048	0.00009803	Miscellaneous	Cytochrome P450 family 77 subfamily B polypeptide 1 (CYP77B1)	1.00E-241
T2-L2	blue	Csa5G604150	3.14	0.01	Redox	Thioredoxin 2 (TRX2)	
T2-L2	blue	Csa5G223070	3.216	0.003151	Transcription factor	WRKY DNA-binding protein 54 (WRKY54)	3.00E-96
T2-L2	blue	Csa2G359890	3.289	3.70E-03	Not assigned	unknown	
T2-L2	blue	Csa1G596520	3.4	0.0002165	Protein degradation	Cystein proteinases superfamily protein	2.00E-168
T2-L2	blue	Csa7G395800	3.415	0.001823	Miscellaneous	Glutathione S-transferase phi 8 (GSTF8)	3.00E-82
T2-L2	blue	Csa3G119700	3.575	0.00005376	Transcription factor	WRKY DNA-binding protein 53 (WRKY53)	1.00E-102
T2-L2	blue	Csa6G381850	3.831	0.001156	Transport	Amino acid permease 3 (AAP3)	2.00E-234
T2-L2	blue	Csa1G033160	4.495	0.006049	Miscellaneous	Glutathione S-transferase tau 9 (GSTU9)	3.00E-68
T2-L2	blue	Csa6G486960	4.496	0.000002502	Transcription factor	WRKY DNA-binding protein 51 (WRKY51)	1.00E-36
T2-L2	blue	Csa2G058620	5.345	0.003945	Abiotic stress	DNAJ heat shock N-terminal domain-containing protein	2.00E-137

Supplementary Table 1 (Continued)

T2-L2	blue	Csa1G574970	1.827	1.18E-05	Major CHO metabolism	Hexokinase 2 (HXK2)	7.00E-51
T2-L2	blue	Csa4G618520	1.837	0.0001765	Biodegradation of xenobiotic	UDP glycosyltransferase (UGT)	1.00E-132
T2-L2	blue	Csa2G003610	1.841	0.0003098	Lipid metabolism	Abnormal inflorescence meristem (AIM1)	3.00E-294
T2-L2	blue	Csa3G730930	1.847	0.00004243	Transport	Ammonium transporter 2 (AMT2)	8E-220
T2-L2	blue	Csa6G493850	1.86	1.50E-05	Not assigned	unknown	
T2-L2	blue	Csa3G159420	1.871	1.61E-03	Not assigned	unknown	
T2-L2	blue	Csa1G528610	1.874	0.00435	Lipid metabolism	Thylakoid membrane anion transporter 1 (ANTR1)	7.00E-236
T2-L2	blue	Csa1G181360	1.875	0.001947	Transport	non-intrinsic ABC protein 9 (NAP9)	3.00E-156
T2-L2	blue	Csa3G154410	2.05	1.63E-10	Not assigned	unknown	
T2-L2	blue	Csa3G183950	2.092	0.0004285	Abiotic stress	Heat shock protein 83	0.00E+00
T2-L2	blue	Csa2G296090	2.143	2.44E-04	Not assigned	unknown	
T2-L2	blue	Csa7G291720	2.169	0.006566	Lipid metabolism	Choline kinase	4.00E-133
T2-L2	blue	Csa2G427310	2.238	0.007581	Transcription factor	MYB domain protein 73 (MYB73)	1.00E-88
T2-L2	blue	Csa3G881750	2.24	0.009561	Lipid metabolism	PR5-like receptor kinase (PR5K)	4.00E-154
T2-L2	blue	Csa5G589260	2.243	0	Amino acid metabolism	O-acetylserine(thiol) lyase (OAS-TL)	4.00E-162
T2-L2	blue	Csa3G733350	2.317	0.00004435	Cell organization	RHO guanyl-nucleotide exchange factor 11 (ROPGEF11)	9.00E-41
T2-L2	blue	Csa2G004690	2.522	0.000004593	Transcription factor	Heat shock transcription factor C1 (HSFC1)	5.00E-67
T2-L2	blue	Csa7G387690	2.631	0.000001654	Transport	Sulfate transporter 3;1 (SULTR 3;1)	3.00E-307
T2-L2	blue	Csa1G008520	2.771	0.00804	Transport	Plasma membrane proton ATPase 4 (HA4)	0.00E+00
T2-L2	blue	Csa6G514850	3.048	0.00009803	Miscellaneous	Cytochrome P450 family 77 subfamily B polypeptide 1 (CYP77B1)	1.00E-241
T2-L2	blue	Csa5G604150	3.14	0.01	Redox	Thioredoxin 2 (TRX2)	
T2-L2	blue	Csa5G223070	3.216	0.003151	Transcription factor	WRKY DNA-binding protein 54 (WRKY54)	3.00E-96
T2-L2	blue	Csa2G359890	3.289	3.70E-03	Not assigned	unknown	
T2-L2	blue	Csa1G596520	3.4	0.0002165	Protein degradation	Cystein proteinases superfamily protein	2.00E-168
T2-L2	blue	Csa7G395800	3.415	0.001823	Miscellaneous	Glutathione S-transferase phi 8 (GSTF8)	3.00E-82
T2-L2	blue	Csa3G119700	3.575	0.00005376	Transcription factor	WRKY DNA-binding protein 53 (WRKY53)	1.00E-102
T2-L2	blue	Csa6G381850	3.831	0.001156	Transport	Amino acid permease 3 (AAP3)	2.00E-234
T2-L2	blue	Csa1G033160	4.495	0.006049	Miscellaneous	Glutathione S-transferase tau 9 (GSTU9)	3.00E-68
T2-L2	blue	Csa6G486960	4.496	0.000002502	Transcription factor	WRKY DNA-binding protein 51 (WRKY51)	1.00E-36
T2-L2	blue	Csa2G058620	5.345	0.003945	Abiotic stress	DNAJ heat shock N-terminal domain-containing protein	2.00E-137

Supplementary Table 1 (Continued)

T2-L2	blue	Csa1G574970	1.827	1.18E-05	Major CHO metabolism	Hexokinase 2 (HXK2)	7.00E-51
T2-L2	blue	Csa4G618520	1.837	0.0001765	Biodegradation of xenobiotic	UDP glycosyltransferase (UGT)	1.00E-132
T2-L2	blue	Csa2G003610	1.841	0.0003098	Lipid metabolism	Abnormal inflorescence meristem (AIM1)	3.00E-294
T2-L2	blue	Csa3G730930	1.847	0.00004243	Transport	Ammonium transporter 2 (AMT2)	8E-220
T2-L2	blue	Csa6G493850	1.86	1.50E-05	Not assigned	unknown	
T2-L2	blue	Csa3G159420	1.871	1.61E-03	Not assigned	unknown	
T2-L2	blue	Csa1G528610	1.874	0.00435	Lipid metabolism	Thylakoid membrane anion transporter 1 (ANTR1)	7.00E-236
T2-L2	blue	Csa1G181360	1.875	0.001947	Transport	non-intrinsic ABC protein 9 (NAP9)	3.00E-156
T2-L2	blue	Csa3G154410	2.05	1.63E-10	Not assigned	unknown	
T2-L2	blue	Csa3G183950	2.092	0.0004285	Abiotic stress	Heat shock protein 83	0.00E+00
T2-L2	blue	Csa2G296090	2.143	2.44E-04	Not assigned	unknown	
T2-L2	blue	Csa7G291720	2.169	0.006566	Lipid metabolism	Choline kinase	4.00E-133
T2-L2	blue	Csa2G427310	2.238	0.007581	Transcription factor	MYB domain protein 73 (MYB73)	1.00E-88
T2-L2	blue	Csa3G881750	2.24	0.009561	Lipid metabolism	PR5-like receptor kinase (PR5K)	4.00E-154
T2-L2	blue	Csa5G589260	2.243	0	Amino acid metabolism	O-acetylserine(thiol) lyase (OAS-TL)	4.00E-162
T2-L2	blue	Csa3G733350	2.317	0.00004435	Cell organization	RHO guanyl-nucleotide exchange factor 11 (ROPGEF11)	9.00E-41
T2-L2	blue	Csa2G004690	2.522	0.000004593	Transcription factor	Heat shock transcription factor C1 (HSFC1)	5.00E-67
T2-L2	blue	Csa7G387690	2.631	0.000001654	Transport	Sulfate transporter 3;1 (SULTR 3;1)	3.00E-307
T2-L2	blue	Csa1G008520	2.771	0.00804	Transport	Plasma membrane proton ATPase 4 (HA4)	0.00E+00
T2-L2	blue	Csa6G514850	3.048	0.00009803	Miscellaneous	Cytochrome P450 family 77 subfamily B polypeptide 1 (CYP77B1)	1.00E-241
T2-L2	blue	Csa5G604150	3.14	0.01	Redox	Thioredoxin 2 (TRX2)	
T2-L2	blue	Csa5G223070	3.216	0.003151	Transcription factor	WRKY DNA-binding protein 54 (WRKY54)	3.00E-96
T2-L2	blue	Csa2G359890	3.289	3.70E-03	Not assigned	unknown	
T2-L2	blue	Csa1G596520	3.4	0.0002165	Protein degradation	Cystein proteinases superfamily protein	2.00E-168
T2-L2	blue	Csa7G395800	3.415	0.001823	Miscellaneous	Glutathione S-transferase phi 8 (GSTF8)	3.00E-82
T2-L2	blue	Csa3G119700	3.575	0.00005376	Transcription factor	WRKY DNA-binding protein 53 (WRKY53)	1.00E-102
T2-L2	blue	Csa6G381850	3.831	0.001156	Transport	Amino acid permease 3 (AAP3)	2.00E-234
T2-L2	blue	Csa1G033160	4.495	0.006049	Miscellaneous	Glutathione S-transferase tau 9 (GSTU9)	3.00E-68
T2-L2	blue	Csa6G486960	4.496	0.000002502	Transcription factor	WRKY DNA-binding protein 51 (WRKY51)	1.00E-36
T2-L2	blue	Csa2G058620	5.345	0.003945	Abiotic stress	DNAJ heat shock N-terminal domain-containing protein	2.00E-137

Supplementary Table 1 (Continued)

T2-L2	blue	Csa1G574970	1.827	1.18E-05	Major CHO metabolism	Hexokinase 2 (HXK2)	7.00E-51
T2-L2	blue	Csa4G618520	1.837	0.0001765	Biodegradation of xenobiotic	UDP glycosyltransferase (UGT)	1.00E-132
T2-L2	blue	Csa2G003610	1.841	0.0003098	Lipid metabolism	Abnormal inflorescence meristem (AIM1)	3.00E-294
T2-L2	blue	Csa3G730930	1.847	0.00004243	Transport	Ammonium transporter 2 (AMT2)	8E-220
T2-L2	blue	Csa6G493850	1.86	1.50E-05	Not assigned	unknown	
T2-L2	blue	Csa3G159420	1.871	1.61E-03	Not assigned	unknown	
T2-L2	blue	Csa1G528610	1.874	0.00435	Lipid metabolism	Thylakoid membrane anion transporter 1 (ANTR1)	7.00E-236
T2-L2	blue	Csa1G181360	1.875	0.001947	Transport	non-intrinsic ABC protein 9 (NAP9)	3.00E-156
T2-L2	blue	Csa3G154410	2.05	1.63E-10	Not assigned	unknown	
T2-L2	blue	Csa3G183950	2.092	0.0004285	Abiotic stress	Heat shock protein 83	0.00E+00
T2-L2	blue	Csa2G296090	2.143	2.44E-04	Not assigned	unknown	
T2-L2	blue	Csa7G291720	2.169	0.006566	Lipid metabolism	Choline kinase	4.00E-133
T2-L2	blue	Csa2G427310	2.238	0.007581	Transcription factor	MYB domain protein 73 (MYB73)	1.00E-88
T2-L2	blue	Csa3G881750	2.24	0.009561	Lipid metabolism	PR5-like receptor kinase (PR5K)	4.00E-154
T2-L2	blue	Csa5G589260	2.243	0	Amino acid metabolism	O-acetylserine(thiol) lyase (OAS-TL)	4.00E-162
T2-L2	blue	Csa3G733350	2.317	0.00004435	Cell organization	RHO guanyl-nucleotide exchange factor 11 (ROPGEF11)	9.00E-41
T2-L2	blue	Csa2G004690	2.522	0.000004593	Transcription factor	Heat shock transcription factor C1 (HSFC1)	5.00E-67
T2-L2	blue	Csa7G387690	2.631	0.000001654	Transport	Sulfate transporter 3;1 (SULTR 3;1)	3.00E-307
T2-L2	blue	Csa1G008520	2.771	0.00804	Transport	Plasma membrane proton ATPase 4 (HA4)	0.00E+00
T2-L2	blue	Csa6G514850	3.048	0.00009803	Miscellaneous	Cytochrome P450 family 77 subfamily B polypeptide 1 (CYP77B1)	1.00E-241
T2-L2	blue	Csa5G604150	3.14	0.01	Redox	Thioredoxin 2 (TRX2)	
T2-L2	blue	Csa5G223070	3.216	0.003151	Transcription factor	WRKY DNA-binding protein 54 (WRKY54)	3.00E-96
T2-L2	blue	Csa2G359890	3.289	3.70E-03	Not assigned	unknown	
T2-L2	blue	Csa1G596520	3.4	0.0002165	Protein degradation	Cystein proteinases superfamily protein	2.00E-168
T2-L2	blue	Csa7G395800	3.415	0.001823	Miscellaneous	Glutathione S-transferase phi 8 (GSTF8)	3.00E-82
T2-L2	blue	Csa3G119700	3.575	0.00005376	Transcription factor	WRKY DNA-binding protein 53 (WRKY53)	1.00E-102
T2-L2	blue	Csa6G381850	3.831	0.001156	Transport	Amino acid permease 3 (AAP3)	2.00E-234
T2-L2	blue	Csa1G033160	4.495	0.006049	Miscellaneous	Glutathione S-transferase tau 9 (GSTU9)	3.00E-68
T2-L2	blue	Csa6G486960	4.496	0.000002502	Transcription factor	WRKY DNA-binding protein 51 (WRKY51)	1.00E-36
T2-L2	blue	Csa2G058620	5.345	0.003945	Abiotic stress	DNAJ heat shock N-terminal domain-containing protein	2.00E-137

Supplementary Table 1 (Continued)

T2-L2	blue	Csa1G574970	1.827	1.18E-05	Major CHO metabolism	Hexokinase 2 (HXK2)	7.00E-51
T2-L2	blue	Csa4G618520	1.837	0.0001765	Biodegradation of xenobiotic	UDP glycosyltransferase (UGT)	1.00E-132
T2-L2	blue	Csa2G003610	1.841	0.0003098	Lipid metabolism	Abnormal inflorescence meristem (AIM1)	3.00E-294
T2-L2	blue	Csa3G730930	1.847	0.00004243	Transport	Ammonium transporter 2 (AMT2)	8E-220
T2-L2	blue	Csa6G493850	1.86	1.50E-05	Not assigned	unknown	
T2-L2	blue	Csa3G159420	1.871	1.61E-03	Not assigned	unknown	
T2-L2	blue	Csa1G528610	1.874	0.00435	Lipid metabolism	Thylakoid membrane anion transporter 1 (ANTR1)	7.00E-236
T2-L2	blue	Csa1G181360	1.875	0.001947	Transport	non-intrinsic ABC protein 9 (NAP9)	3.00E-156
T2-L2	blue	Csa3G154410	2.05	1.63E-10	Not assigned	unknown	
T2-L2	blue	Csa3G183950	2.092	0.0004285	Abiotic stress	Heat shock protein 83	0.00E+00
T2-L2	blue	Csa2G296090	2.143	2.44E-04	Not assigned	unknown	
T2-L2	blue	Csa7G291720	2.169	0.006566	Lipid metabolism	Choline kinase	4.00E-133
T2-L2	blue	Csa2G427310	2.238	0.007581	Transcription factor	MYB domain protein 73 (MYB73)	1.00E-88
T2-L2	blue	Csa3G881750	2.24	0.009561	Lipid metabolism	PR5-like receptor kinase (PR5K)	4.00E-154
T2-L2	blue	Csa5G589260	2.243	0	Amino acid metabolism	O-acetylserine(thiol) lyase (OAS-TL)	4.00E-162
T2-L2	blue	Csa3G733350	2.317	0.00004435	Cell organization	RHO guanyl-nucleotide exchange factor 11 (ROPGEF11)	9.00E-41
T2-L2	blue	Csa2G004690	2.522	0.000004593	Transcription factor	Heat shock transcription factor C1 (HSFC1)	5.00E-67
T2-L2	blue	Csa7G387690	2.631	0.000001654	Transport	Sulfate transporter 3;1 (SULTR 3;1)	3.00E-307
T2-L2	blue	Csa1G008520	2.771	0.00804	Transport	Plasma membrane proton ATPase 4 (HA4)	0.00E+00
T2-L2	blue	Csa6G514850	3.048	0.00009803	Miscellaneous	Cytochrome P450 family 77 subfamily B polypeptide 1 (CYP77B1)	1.00E-241
T2-L2	blue	Csa5G604150	3.14	0.01	Redox	Thioredoxin 2 (TRX2)	
T2-L2	blue	Csa5G223070	3.216	0.003151	Transcription factor	WRKY DNA-binding protein 54 (WRKY54)	3.00E-96
T2-L2	blue	Csa2G359890	3.289	3.70E-03	Not assigned	unknown	
T2-L2	blue	Csa1G596520	3.4	0.0002165	Protein degradation	Cystein proteinases superfamily protein	2.00E-168
T2-L2	blue	Csa7G395800	3.415	0.001823	Miscellaneous	Glutathione S-transferase phi 8 (GSTF8)	3.00E-82
T2-L2	blue	Csa3G119700	3.575	0.00005376	Transcription factor	WRKY DNA-binding protein 53 (WRKY53)	1.00E-102
T2-L2	blue	Csa6G381850	3.831	0.001156	Transport	Amino acid permease 3 (AAP3)	2.00E-234
T2-L2	blue	Csa1G033160	4.495	0.006049	Miscellaneous	Glutathione S-transferase tau 9 (GSTU9)	3.00E-68
T2-L2	blue	Csa6G486960	4.496	0.000002502	Transcription factor	WRKY DNA-binding protein 51 (WRKY51)	1.00E-36
T2-L2	blue	Csa2G058620	5.345	0.003945	Abiotic stress	DNAJ heat shock N-terminal domain-containing protein	2.00E-137

Supplementary Table 1 (Continued)

T2-L2	blue	Csa1G574970	1.827	1.18E-05	Major CHO metabolism	Hexokinase 2 (HXK2)	7.00E-51
T2-L2	blue	Csa4G618520	1.837	0.0001765	Biodegradation of xenobiotic	UDP glycosyltransferase (UGT)	1.00E-132
T2-L2	blue	Csa2G003610	1.841	0.0003098	Lipid metabolism	Abnormal inflorescence meristem (AIM1)	3.00E-294
T2-L2	blue	Csa3G730930	1.847	0.00004243	Transport	Ammonium transporter 2 (AMT2)	8E-220
T2-L2	blue	Csa6G493850	1.86	1.50E-05	Not assigned	unknown	
T2-L2	blue	Csa3G159420	1.871	1.61E-03	Not assigned	unknown	
T2-L2	blue	Csa1G528610	1.874	0.00435	Lipid metabolism	Thylakoid membrane anion transporter 1 (ANTR1)	7.00E-236
T2-L2	blue	Csa1G181360	1.875	0.001947	Transport	non-intrinsic ABC protein 9 (NAP9)	3.00E-156
T2-L2	blue	Csa3G154410	2.05	1.63E-10	Not assigned	unknown	
T2-L2	blue	Csa3G183950	2.092	0.0004285	Abiotic stress	Heat shock protein 83	0.00E+00
T2-L2	blue	Csa2G296090	2.143	2.44E-04	Not assigned	unknown	
T2-L2	blue	Csa7G291720	2.169	0.006566	Lipid metabolism	Choline kinase	4.00E-133
T2-L2	blue	Csa2G427310	2.238	0.007581	Transcription factor	MYB domain protein 73 (MYB73)	1.00E-88
T2-L2	blue	Csa3G881750	2.24	0.009561	Lipid metabolism	PR5-like receptor kinase (PR5K)	4.00E-154
T2-L2	blue	Csa5G589260	2.243	0	Amino acid metabolism	O-acetylserine(thiol) lyase (OAS-TL)	4.00E-162
T2-L2	blue	Csa3G733350	2.317	0.00004435	Cell organization	RHO guanyl-nucleotide exchange factor 11 (ROPGEF11)	9.00E-41
T2-L2	blue	Csa2G004690	2.522	0.000004593	Transcription factor	Heat shock transcription factor C1 (HSFC1)	5.00E-67
T2-L2	blue	Csa7G387690	2.631	0.000001654	Transport	Sulfate transporter 3;1 (SULTR 3;1)	3.00E-307
T2-L2	blue	Csa1G008520	2.771	0.00804	Transport	Plasma membrane proton ATPase 4 (HA4)	0.00E+00
T2-L2	blue	Csa6G514850	3.048	0.00009803	Miscellaneous	Cytochrome P450 family 77 subfamily B polypeptide 1 (CYP77B1)	1.00E-241
T2-L2	blue	Csa5G604150	3.14	0.01	Redox	Thioredoxin 2 (TRX2)	
T2-L2	blue	Csa5G223070	3.216	0.003151	Transcription factor	WRKY DNA-binding protein 54 (WRKY54)	3.00E-96
T2-L2	blue	Csa2G359890	3.289	3.70E-03	Not assigned	unknown	
T2-L2	blue	Csa1G596520	3.4	0.0002165	Protein degradation	Cystein proteinases superfamily protein	2.00E-168
T2-L2	blue	Csa7G395800	3.415	0.001823	Miscellaneous	Glutathione S-transferase phi 8 (GSTF8)	3.00E-82
T2-L2	blue	Csa3G119700	3.575	0.00005376	Transcription factor	WRKY DNA-binding protein 53 (WRKY53)	1.00E-102
T2-L2	blue	Csa6G381850	3.831	0.001156	Transport	Amino acid permease 3 (AAP3)	2.00E-234
T2-L2	blue	Csa1G033160	4.495	0.006049	Miscellaneous	Glutathione S-transferase tau 9 (GSTU9)	3.00E-68
T2-L2	blue	Csa6G486960	4.496	0.000002502	Transcription factor	WRKY DNA-binding protein 51 (WRKY51)	1.00E-36
T2-L2	blue	Csa2G058620	5.345	0.003945	Abiotic stress	DNAJ heat shock N-terminal domain-containing protein	2.00E-137

Supplementary Table 1 (Continued)

T2-L2	blue	Csa1G574970	1.827	1.18E-05	Major CHO metabolism	Hexokinase 2 (HXK2)	7.00E-51
T2-L2	blue	Csa4G618520	1.837	0.0001765	Biodegradation of xenobiotic	UDP glycosyltransferase (UGT)	1.00E-132
T2-L2	blue	Csa2G003610	1.841	0.0003098	Lipid metabolism	Abnormal inflorescence meristem (AIM1)	3.00E-294
T2-L2	blue	Csa3G730930	1.847	0.00004243	Transport	Ammonium transporter 2 (AMT2)	8E-220
T2-L2	blue	Csa6G493850	1.86	1.50E-05	Not assigned	unknown	
T2-L2	blue	Csa3G159420	1.871	1.61E-03	Not assigned	unknown	
T2-L2	blue	Csa1G528610	1.874	0.00435	Lipid metabolism	Thylakoid membrane anion transporter 1 (ANTR1)	7.00E-236
T2-L2	blue	Csa1G181360	1.875	0.001947	Transport	non-intrinsic ABC protein 9 (NAP9)	3.00E-156
T2-L2	blue	Csa3G154410	2.05	1.63E-10	Not assigned	unknown	
T2-L2	blue	Csa3G183950	2.092	0.0004285	Abiotic stress	Heat shock protein 83	0.00E+00
T2-L2	blue	Csa2G296090	2.143	2.44E-04	Not assigned	unknown	
T2-L2	blue	Csa7G291720	2.169	0.006566	Lipid metabolism	Choline kinase	4.00E-133
T2-L2	blue	Csa2G427310	2.238	0.007581	Transcription factor	MYB domain protein 73 (MYB73)	1.00E-88
T2-L2	blue	Csa3G881750	2.24	0.009561	Lipid metabolism	PR5-like receptor kinase (PR5K)	4.00E-154
T2-L2	blue	Csa5G589260	2.243	0	Amino acid metabolism	O-acetylserine(thiol) lyase (OAS-TL)	4.00E-162
T2-L2	blue	Csa3G733350	2.317	0.00004435	Cell organization	RHO guanyl-nucleotide exchange factor 11 (ROPGEF11)	9.00E-41
T2-L2	blue	Csa2G004690	2.522	0.000004593	Transcription factor	Heat shock transcription factor C1 (HSFC1)	5.00E-67
T2-L2	blue	Csa7G387690	2.631	0.000001654	Transport	Sulfate transporter 3;1 (SULTR 3;1)	3.00E-307
T2-L2	blue	Csa1G008520	2.771	0.00804	Transport	Plasma membrane proton ATPase 4 (HA4)	0.00E+00
T2-L2	blue	Csa6G514850	3.048	0.00009803	Miscellaneous	Cytochrome P450 family 77 subfamily B polypeptide 1 (CYP77B1)	1.00E-241
T2-L2	blue	Csa5G604150	3.14	0.01	Redox	Thioredoxin 2 (TRX2)	
T2-L2	blue	Csa5G223070	3.216	0.003151	Transcription factor	WRKY DNA-binding protein 54 (WRKY54)	3.00E-96
T2-L2	blue	Csa2G359890	3.289	3.70E-03	Not assigned	unknown	
T2-L2	blue	Csa1G596520	3.4	0.0002165	Protein degradation	Cystein proteinases superfamily protein	2.00E-168
T2-L2	blue	Csa7G395800	3.415	0.001823	Miscellaneous	Glutathione S-transferase phi 8 (GSTF8)	3.00E-82
T2-L2	blue	Csa3G119700	3.575	0.00005376	Transcription factor	WRKY DNA-binding protein 53 (WRKY53)	1.00E-102
T2-L2	blue	Csa6G381850	3.831	0.001156	Transport	Amino acid permease 3 (AAP3)	2.00E-234
T2-L2	blue	Csa1G033160	4.495	0.006049	Miscellaneous	Glutathione S-transferase tau 9 (GSTU9)	3.00E-68
T2-L2	blue	Csa6G486960	4.496	0.000002502	Transcription factor	WRKY DNA-binding protein 51 (WRKY51)	1.00E-36
T2-L2	blue	Csa2G058620	5.345	0.003945	Abiotic stress	DNAJ heat shock N-terminal domain-containing protein	2.00E-137

Supplementary Table 1 (Continued)

T2-L2	blue	Csa1G574970	1.827	1.18E-05	Major CHO metabolism	Hexokinase 2 (HXK2)	7.00E-51
T2-L2	blue	Csa4G618520	1.837	0.0001765	Biodegradation of xenobiotic	UDP glycosyltransferase (UGT)	1.00E-132
T2-L2	blue	Csa2G003610	1.841	0.0003098	Lipid metabolism	Abnormal inflorescence meristem (AIM1)	3.00E-294
T2-L2	blue	Csa3G730930	1.847	0.00004243	Transport	Ammonium transporter 2 (AMT2)	8E-220
T2-L2	blue	Csa6G493850	1.86	1.50E-05	Not assigned	unknown	
T2-L2	blue	Csa3G159420	1.871	1.61E-03	Not assigned	unknown	
T2-L2	blue	Csa1G528610	1.874	0.00435	Lipid metabolism	Thylakoid membrane anion transporter 1 (ANTR1)	7.00E-236
T2-L2	blue	Csa1G181360	1.875	0.001947	Transport	non-intrinsic ABC protein 9 (NAP9)	3.00E-156
T2-L2	blue	Csa3G154410	2.05	1.63E-10	Not assigned	unknown	
T2-L2	blue	Csa3G183950	2.092	0.0004285	Abiotic stress	Heat shock protein 83	0.00E+00
T2-L2	blue	Csa2G296090	2.143	2.44E-04	Not assigned	unknown	
T2-L2	blue	Csa7G291720	2.169	0.006566	Lipid metabolism	Choline kinase	4.00E-133
T2-L2	blue	Csa2G427310	2.238	0.007581	Transcription factor	MYB domain protein 73 (MYB73)	1.00E-88
T2-L2	blue	Csa3G881750	2.24	0.009561	Lipid metabolism	PR5-like receptor kinase (PR5K)	4.00E-154
T2-L2	blue	Csa5G589260	2.243	0	Amino acid metabolism	O-acetylserine(thiol) lyase (OAS-TL)	4.00E-162
T2-L2	blue	Csa3G733350	2.317	0.00004435	Cell organization	RHO guanyl-nucleotide exchange factor 11 (ROPGEF11)	9.00E-41
T2-L2	blue	Csa2G004690	2.522	0.000004593	Transcription factor	Heat shock transcription factor C1 (HSFC1)	5.00E-67
T2-L2	blue	Csa7G387690	2.631	0.000001654	Transport	Sulfate transporter 3;1 (SULTR 3;1)	3.00E-307
T2-L2	blue	Csa1G008520	2.771	0.00804	Transport	Plasma membrane proton ATPase 4 (HA4)	0.00E+00
T2-L2	blue	Csa6G514850	3.048	0.00009803	Miscellaneous	Cytochrome P450 family 77 subfamily B polypeptide 1 (CYP77B1)	1.00E-241
T2-L2	blue	Csa5G604150	3.14	0.01	Redox	Thioredoxin 2 (TRX2)	
T2-L2	blue	Csa5G223070	3.216	0.003151	Transcription factor	WRKY DNA-binding protein 54 (WRKY54)	3.00E-96
T2-L2	blue	Csa2G359890	3.289	3.70E-03	Not assigned	unknown	
T2-L2	blue	Csa1G596520	3.4	0.0002165	Protein degradation	Cystein proteinases superfamily protein	2.00E-168
T2-L2	blue	Csa7G395800	3.415	0.001823	Miscellaneous	Glutathione S-transferase phi 8 (GSTF8)	3.00E-82
T2-L2	blue	Csa3G119700	3.575	0.00005376	Transcription factor	WRKY DNA-binding protein 53 (WRKY53)	1.00E-102
T2-L2	blue	Csa6G381850	3.831	0.001156	Transport	Amino acid permease 3 (AAP3)	2.00E-234
T2-L2	blue	Csa1G033160	4.495	0.006049	Miscellaneous	Glutathione S-transferase tau 9 (GSTU9)	3.00E-68
T2-L2	blue	Csa6G486960	4.496	0.000002502	Transcription factor	WRKY DNA-binding protein 51 (WRKY51)	1.00E-36
T2-L2	blue	Csa2G058620	5.345	0.003945	Abiotic stress	DNAJ heat shock N-terminal domain-containing protein	2.00E-137

Supplementary Table 1 (Continued)

T2-L2	blue	Csa1G574970	1.827	1.18E-05	Major CHO metabolism	Hexokinase 2 (HXK2)	7.00E-51
T2-L2	blue	Csa4G618520	1.837	0.0001765	Biodegradation of xenobiotic	UDP glycosyltransferase (UGT)	1.00E-132
T2-L2	blue	Csa2G003610	1.841	0.0003098	Lipid metabolism	Abnormal inflorescence meristem (AIM1)	3.00E-294
T2-L2	blue	Csa3G730930	1.847	0.00004243	Transport	Ammonium transporter 2 (AMT2)	8E-220
T2-L2	blue	Csa6G493850	1.86	1.50E-05	Not assigned	unknown	
T2-L2	blue	Csa3G159420	1.871	1.61E-03	Not assigned	unknown	
T2-L2	blue	Csa1G528610	1.874	0.00435	Lipid metabolism	Thylakoid membrane anion transporter 1 (ANTR1)	7.00E-236
T2-L2	blue	Csa1G181360	1.875	0.001947	Transport	non-intrinsic ABC protein 9 (NAP9)	3.00E-156
T2-L2	blue	Csa3G154410	2.05	1.63E-10	Not assigned	unknown	
T2-L2	blue	Csa3G183950	2.092	0.0004285	Abiotic stress	Heat shock protein 83	0.00E+00
T2-L2	blue	Csa2G296090	2.143	2.44E-04	Not assigned	unknown	
T2-L2	blue	Csa7G291720	2.169	0.006566	Lipid metabolism	Choline kinase	4.00E-133
T2-L2	blue	Csa2G427310	2.238	0.007581	Transcription factor	MYB domain protein 73 (MYB73)	1.00E-88
T2-L2	blue	Csa3G881750	2.24	0.009561	Lipid metabolism	PR5-like receptor kinase (PR5K)	4.00E-154
T2-L2	blue	Csa5G589260	2.243	0	Amino acid metabolism	O-acetylserine(thiol) lyase (OAS-TL)	4.00E-162
T2-L2	blue	Csa3G733350	2.317	0.00004435	Cell organization	RHO guanyl-nucleotide exchange factor 11 (ROPGEF11)	9.00E-41
T2-L2	blue	Csa2G004690	2.522	0.000004593	Transcription factor	Heat shock transcription factor C1 (HSFC1)	5.00E-67
T2-L2	blue	Csa7G387690	2.631	0.000001654	Transport	Sulfate transporter 3;1 (SULTR 3;1)	3.00E-307
T2-L2	blue	Csa1G008520	2.771	0.00804	Transport	Plasma membrane proton ATPase 4 (HA4)	0.00E+00
T2-L2	blue	Csa6G514850	3.048	0.00009803	Miscellaneous	Cytochrome P450 family 77 subfamily B polypeptide 1 (CYP77B1)	1.00E-241
T2-L2	blue	Csa5G604150	3.14	0.01	Redox	Thioredoxin 2 (TRX2)	
T2-L2	blue	Csa5G223070	3.216	0.003151	Transcription factor	WRKY DNA-binding protein 54 (WRKY54)	3.00E-96
T2-L2	blue	Csa2G359890	3.289	3.70E-03	Not assigned	unknown	
T2-L2	blue	Csa1G596520	3.4	0.0002165	Protein degradation	Cystein proteinases superfamily protein	2.00E-168
T2-L2	blue	Csa7G395800	3.415	0.001823	Miscellaneous	Glutathione S-transferase phi 8 (GSTF8)	3.00E-82
T2-L2	blue	Csa3G119700	3.575	0.00005376	Transcription factor	WRKY DNA-binding protein 53 (WRKY53)	1.00E-102
T2-L2	blue	Csa6G381850	3.831	0.001156	Transport	Amino acid permease 3 (AAP3)	2.00E-234
T2-L2	blue	Csa1G033160	4.495	0.006049	Miscellaneous	Glutathione S-transferase tau 9 (GSTU9)	3.00E-68
T2-L2	blue	Csa6G486960	4.496	0.000002502	Transcription factor	WRKY DNA-binding protein 51 (WRKY51)	1.00E-36
T2-L2	blue	Csa2G058620	5.345	0.003945	Abiotic stress	DNAJ heat shock N-terminal domain-containing protein	2.00E-137

Supplementary Table 1 (Continued)

T2-L2	blue	Csa1G574970	1.827	1.18E-05	Major CHO metabolism	Hexokinase 2 (HXK2)	7.00E-51
T2-L2	blue	Csa4G618520	1.837	0.0001765	Biodegradation of xenobiotic	UDP glycosyltransferase (UGT)	1.00E-132
T2-L2	blue	Csa2G003610	1.841	0.0003098	Lipid metabolism	Abnormal inflorescence meristem (AIM1)	3.00E-294
T2-L2	blue	Csa3G730930	1.847	0.00004243	Transport	Ammonium transporter 2 (AMT2)	8E-220
T2-L2	blue	Csa6G493850	1.86	1.50E-05	Not assigned	unknown	
T2-L2	blue	Csa3G159420	1.871	1.61E-03	Not assigned	unknown	
T2-L2	blue	Csa1G528610	1.874	0.00435	Lipid metabolism	Thylakoid membrane anion transporter 1 (ANTR1)	7.00E-236
T2-L2	blue	Csa1G181360	1.875	0.001947	Transport	non-intrinsic ABC protein 9 (NAP9)	3.00E-156
T2-L2	blue	Csa3G154410	2.05	1.63E-10	Not assigned	unknown	
T2-L2	blue	Csa3G183950	2.092	0.0004285	Abiotic stress	Heat shock protein 83	0.00E+00
T2-L2	blue	Csa2G296090	2.143	2.44E-04	Not assigned	unknown	
T2-L2	blue	Csa7G291720	2.169	0.006566	Lipid metabolism	Choline kinase	4.00E-133
T2-L2	blue	Csa2G427310	2.238	0.007581	Transcription factor	MYB domain protein 73 (MYB73)	1.00E-88
T2-L2	blue	Csa3G881750	2.24	0.009561	Lipid metabolism	PR5-like receptor kinase (PR5K)	4.00E-154
T2-L2	blue	Csa5G589260	2.243	0	Amino acid metabolism	O-acetylserine(thiol) lyase (OAS-TL)	4.00E-162
T2-L2	blue	Csa3G733350	2.317	0.00004435	Cell organization	RHO guanyl-nucleotide exchange factor 11 (ROPGEF11)	9.00E-41
T2-L2	blue	Csa2G004690	2.522	0.000004593	Transcription factor	Heat shock transcription factor C1 (HSFC1)	5.00E-67
T2-L2	blue	Csa7G387690	2.631	0.000001654	Transport	Sulfate transporter 3;1 (SULTR 3;1)	3.00E-307
T2-L2	blue	Csa1G008520	2.771	0.00804	Transport	Plasma membrane proton ATPase 4 (HA4)	0.00E+00
T2-L2	blue	Csa6G514850	3.048	0.00009803	Miscellaneous	Cytochrome P450 family 77 subfamily B polypeptide 1 (CYP77B1)	1.00E-241
T2-L2	blue	Csa5G604150	3.14	0.01	Redox	Thioredoxin 2 (TRX2)	
T2-L2	blue	Csa5G223070	3.216	0.003151	Transcription factor	WRKY DNA-binding protein 54 (WRKY54)	3.00E-96
T2-L2	blue	Csa2G359890	3.289	3.70E-03	Not assigned	unknown	
T2-L2	blue	Csa1G596520	3.4	0.0002165	Protein degradation	Cystein proteinases superfamily protein	2.00E-168
T2-L2	blue	Csa7G395800	3.415	0.001823	Miscellaneous	Glutathione S-transferase phi 8 (GSTF8)	3.00E-82
T2-L2	blue	Csa3G119700	3.575	0.00005376	Transcription factor	WRKY DNA-binding protein 53 (WRKY53)	1.00E-102
T2-L2	blue	Csa6G381850	3.831	0.001156	Transport	Amino acid permease 3 (AAP3)	2.00E-234
T2-L2	blue	Csa1G033160	4.495	0.006049	Miscellaneous	Glutathione S-transferase tau 9 (GSTU9)	3.00E-68
T2-L2	blue	Csa6G486960	4.496	0.000002502	Transcription factor	WRKY DNA-binding protein 51 (WRKY51)	1.00E-36
T2-L2	blue	Csa2G058620	5.345	0.003945	Abiotic stress	DNAJ heat shock N-terminal domain-containing protein	2.00E-137

Supplementary Table 1 (Continued)

T2-L2	blue	Csa1G574970	1.827	1.18E-05	Major CHO metabolism	Hexokinase 2 (HXK2)	7.00E-51
T2-L2	blue	Csa4G618520	1.837	0.0001765	Biodegradation of xenobiotic	UDP glycosyltransferase (UGT)	1.00E-132
T2-L2	blue	Csa2G003610	1.841	0.0003098	Lipid metabolism	Abnormal inflorescence meristem (AIM1)	3.00E-294
T2-L2	blue	Csa3G730930	1.847	0.00004243	Transport	Ammonium transporter 2 (AMT2)	8E-220
T2-L2	blue	Csa6G493850	1.86	1.50E-05	Not assigned	unknown	
T2-L2	blue	Csa3G159420	1.871	1.61E-03	Not assigned	unknown	
T2-L2	blue	Csa1G528610	1.874	0.00435	Lipid metabolism	Thylakoid membrane anion transporter 1 (ANTR1)	7.00E-236
T2-L2	blue	Csa1G181360	1.875	0.001947	Transport	non-intrinsic ABC protein 9 (NAP9)	3.00E-156
T2-L2	blue	Csa3G154410	2.05	1.63E-10	Not assigned	unknown	
T2-L2	blue	Csa3G183950	2.092	0.0004285	Abiotic stress	Heat shock protein 83	0.00E+00
T2-L2	blue	Csa2G296090	2.143	2.44E-04	Not assigned	unknown	
T2-L2	blue	Csa7G291720	2.169	0.006566	Lipid metabolism	Choline kinase	4.00E-133
T2-L2	blue	Csa2G427310	2.238	0.007581	Transcription factor	MYB domain protein 73 (MYB73)	1.00E-88
T2-L2	blue	Csa3G881750	2.24	0.009561	Lipid metabolism	PR5-like receptor kinase (PR5K)	4.00E-154
T2-L2	blue	Csa5G589260	2.243	0	Amino acid metabolism	O-acetylserine(thiol) lyase (OAS-TL)	4.00E-162
T2-L2	blue	Csa3G733350	2.317	0.00004435	Cell organization	RHO guanyl-nucleotide exchange factor 11 (ROPGEF11)	9.00E-41
T2-L2	blue	Csa2G004690	2.522	0.000004593	Transcription factor	Heat shock transcription factor C1 (HSFC1)	5.00E-67
T2-L2	blue	Csa7G387690	2.631	0.000001654	Transport	Sulfate transporter 3;1 (SULTR 3;1)	3.00E-307
T2-L2	blue	Csa1G008520	2.771	0.00804	Transport	Plasma membrane proton ATPase 4 (HA4)	0.00E+00
T2-L2	blue	Csa6G514850	3.048	0.00009803	Miscellaneous	Cytochrome P450 family 77 subfamily B polypeptide 1 (CYP77B1)	1.00E-241
T2-L2	blue	Csa5G604150	3.14	0.01	Redox	Thioredoxin 2 (TRX2)	
T2-L2	blue	Csa5G223070	3.216	0.003151	Transcription factor	WRKY DNA-binding protein 54 (WRKY54)	3.00E-96
T2-L2	blue	Csa2G359890	3.289	3.70E-03	Not assigned	unknown	
T2-L2	blue	Csa1G596520	3.4	0.0002165	Protein degradation	Cystein proteinases superfamily protein	2.00E-168
T2-L2	blue	Csa7G395800	3.415	0.001823	Miscellaneous	Glutathione S-transferase phi 8 (GSTF8)	3.00E-82
T2-L2	blue	Csa3G119700	3.575	0.00005376	Transcription factor	WRKY DNA-binding protein 53 (WRKY53)	1.00E-102
T2-L2	blue	Csa6G381850	3.831	0.001156	Transport	Amino acid permease 3 (AAP3)	2.00E-234
T2-L2	blue	Csa1G033160	4.495	0.006049	Miscellaneous	Glutathione S-transferase tau 9 (GSTU9)	3.00E-68
T2-L2	blue	Csa6G486960	4.496	0.000002502	Transcription factor	WRKY DNA-binding protein 51 (WRKY51)	1.00E-36
T2-L2	blue	Csa2G058620	5.345	0.003945	Abiotic stress	DNAJ heat shock N-terminal domain-containing protein	2.00E-137

Supplementary Table 1 (Continued)

T2-L2	blue	Csa1G574970	1.827	1.18E-05	Major CHO metabolism	Hexokinase 2 (HXK2)	7.00E-51
T2-L2	blue	Csa4G618520	1.837	0.0001765	Biodegradation of xenobiotic	UDP glycosyltransferase (UGT)	1.00E-132
T2-L2	blue	Csa2G003610	1.841	0.0003098	Lipid metabolism	Abnormal inflorescence meristem (AIM1)	3.00E-294
T2-L2	blue	Csa3G730930	1.847	0.00004243	Transport	Ammonium transporter 2 (AMT2)	8E-220
T2-L2	blue	Csa6G493850	1.86	1.50E-05	Not assigned	unknown	
T2-L2	blue	Csa3G159420	1.871	1.61E-03	Not assigned	unknown	
T2-L2	blue	Csa1G528610	1.874	0.00435	Lipid metabolism	Thylakoid membrane anion transporter 1 (ANTR1)	7.00E-236
T2-L2	blue	Csa1G181360	1.875	0.001947	Transport	non-intrinsic ABC protein 9 (NAP9)	3.00E-156
T2-L2	blue	Csa3G154410	2.05	1.63E-10	Not assigned	unknown	
T2-L2	blue	Csa3G183950	2.092	0.0004285	Abiotic stress	Heat shock protein 83	0.00E+00
T2-L2	blue	Csa2G296090	2.143	2.44E-04	Not assigned	unknown	
T2-L2	blue	Csa7G291720	2.169	0.006566	Lipid metabolism	Choline kinase	4.00E-133
T2-L2	blue	Csa2G427310	2.238	0.007581	Transcription factor	MYB domain protein 73 (MYB73)	1.00E-88
T2-L2	blue	Csa3G881750	2.24	0.009561	Lipid metabolism	PR5-like receptor kinase (PR5K)	4.00E-154
T2-L2	blue	Csa5G589260	2.243	0	Amino acid metabolism	O-acetylserine(thiol) lyase (OAS-TL)	4.00E-162
T2-L2	blue	Csa3G733350	2.317	0.00004435	Cell organization	RHO guanyl-nucleotide exchange factor 11 (ROPGEF11)	9.00E-41
T2-L2	blue	Csa2G004690	2.522	0.000004593	Transcription factor	Heat shock transcription factor C1 (HSFC1)	5.00E-67
T2-L2	blue	Csa7G387690	2.631	0.000001654	Transport	Sulfate transporter 3;1 (SULTR 3;1)	3.00E-307
T2-L2	blue	Csa1G008520	2.771	0.00804	Transport	Plasma membrane proton ATPase 4 (HA4)	0.00E+00
T2-L2	blue	Csa6G514850	3.048	0.00009803	Miscellaneous	Cytochrome P450 family 77 subfamily B polypeptide 1 (CYP77B1)	1.00E-241
T2-L2	blue	Csa5G604150	3.14	0.01	Redox	Thioredoxin 2 (TRX2)	
T2-L2	blue	Csa5G223070	3.216	0.003151	Transcription factor	WRKY DNA-binding protein 54 (WRKY54)	3.00E-96
T2-L2	blue	Csa2G359890	3.289	3.70E-03	Not assigned	unknown	
T2-L2	blue	Csa1G596520	3.4	0.0002165	Protein degradation	Cystein proteinases superfamily protein	2.00E-168
T2-L2	blue	Csa7G395800	3.415	0.001823	Miscellaneous	Glutathione S-transferase phi 8 (GSTF8)	3.00E-82
T2-L2	blue	Csa3G119700	3.575	0.00005376	Transcription factor	WRKY DNA-binding protein 53 (WRKY53)	1.00E-102
T2-L2	blue	Csa6G381850	3.831	0.001156	Transport	Amino acid permease 3 (AAP3)	2.00E-234
T2-L2	blue	Csa1G033160	4.495	0.006049	Miscellaneous	Glutathione S-transferase tau 9 (GSTU9)	3.00E-68
T2-L2	blue	Csa6G486960	4.496	0.000002502	Transcription factor	WRKY DNA-binding protein 51 (WRKY51)	1.00E-36
T2-L2	blue	Csa2G058620	5.345	0.003945	Abiotic stress	DNAJ heat shock N-terminal domain-containing protein	2.00E-137

Supplementary Table 1 (Continued)

T2-L2	blue	Csa1G574970	1.827	1.18E-05	Major CHO metabolism	Hexokinase 2 (HXK2)	7.00E-51
T2-L2	blue	Csa4G618520	1.837	0.0001765	Biodegradation of xenobiotic	UDP glycosyltransferase (UGT)	1.00E-132
T2-L2	blue	Csa2G003610	1.841	0.0003098	Lipid metabolism	Abnormal inflorescence meristem (AIM1)	3.00E-294
T2-L2	blue	Csa3G730930	1.847	0.00004243	Transport	Ammonium transporter 2 (AMT2)	8E-220
T2-L2	blue	Csa6G493850	1.86	1.50E-05	Not assigned	unknown	
T2-L2	blue	Csa3G159420	1.871	1.61E-03	Not assigned	unknown	
T2-L2	blue	Csa1G528610	1.874	0.00435	Lipid metabolism	Thylakoid membrane anion transporter 1 (ANTR1)	7.00E-236
T2-L2	blue	Csa1G181360	1.875	0.001947	Transport	non-intrinsic ABC protein 9 (NAP9)	3.00E-156
T2-L2	blue	Csa3G154410	2.05	1.63E-10	Not assigned	unknown	
T2-L2	blue	Csa3G183950	2.092	0.0004285	Abiotic stress	Heat shock protein 83	0.00E+00
T2-L2	blue	Csa2G296090	2.143	2.44E-04	Not assigned	unknown	
T2-L2	blue	Csa7G291720	2.169	0.006566	Lipid metabolism	Choline kinase	4.00E-133
T2-L2	blue	Csa2G427310	2.238	0.007581	Transcription factor	MYB domain protein 73 (MYB73)	1.00E-88
T2-L2	blue	Csa3G881750	2.24	0.009561	Lipid metabolism	PR5-like receptor kinase (PR5K)	4.00E-154
T2-L2	blue	Csa5G589260	2.243	0	Amino acid metabolism	O-acetylserine(thiol) lyase (OAS-TL)	4.00E-162
T2-L2	blue	Csa3G733350	2.317	0.00004435	Cell organization	RHO guanyl-nucleotide exchange factor 11 (ROPGEF11)	9.00E-41
T2-L2	blue	Csa2G004690	2.522	0.000004593	Transcription factor	Heat shock transcription factor C1 (HSFC1)	5.00E-67
T2-L2	blue	Csa7G387690	2.631	0.000001654	Transport	Sulfate transporter 3;1 (SULTR 3;1)	3.00E-307
T2-L2	blue	Csa1G008520	2.771	0.00804	Transport	Plasma membrane proton ATPase 4 (HA4)	0.00E+00
T2-L2	blue	Csa6G514850	3.048	0.00009803	Miscellaneous	Cytochrome P450 family 77 subfamily B polypeptide 1 (CYP77B1)	1.00E-241
T2-L2	blue	Csa5G604150	3.14	0.01	Redox	Thioredoxin 2 (TRX2)	
T2-L2	blue	Csa5G223070	3.216	0.003151	Transcription factor	WRKY DNA-binding protein 54 (WRKY54)	3.00E-96
T2-L2	blue	Csa2G359890	3.289	3.70E-03	Not assigned	unknown	
T2-L2	blue	Csa1G596520	3.4	0.0002165	Protein degradation	Cystein proteinases superfamily protein	2.00E-168
T2-L2	blue	Csa7G395800	3.415	0.001823	Miscellaneous	Glutathione S-transferase phi 8 (GSTF8)	3.00E-82
T2-L2	blue	Csa3G119700	3.575	0.00005376	Transcription factor	WRKY DNA-binding protein 53 (WRKY53)	1.00E-102
T2-L2	blue	Csa6G381850	3.831	0.001156	Transport	Amino acid permease 3 (AAP3)	2.00E-234
T2-L2	blue	Csa1G033160	4.495	0.006049	Miscellaneous	Glutathione S-transferase tau 9 (GSTU9)	3.00E-68
T2-L2	blue	Csa6G486960	4.496	0.000002502	Transcription factor	WRKY DNA-binding protein 51 (WRKY51)	1.00E-36
T2-L2	blue	Csa2G058620	5.345	0.003945	Abiotic stress	DNAJ heat shock N-terminal domain-containing protein	2.00E-137

Supplementary Table 1 (Continued)

T2-L2	blue	Csa1G574970	1.827	1.18E-05	Major CHO metabolism	Hexokinase 2 (HXK2)	7.00E-51
T2-L2	blue	Csa4G618520	1.837	0.0001765	Biodegradation of xenobiotic	UDP glycosyltransferase (UGT)	1.00E-132
T2-L2	blue	Csa2G003610	1.841	0.0003098	Lipid metabolism	Abnormal inflorescence meristem (AIM1)	3.00E-294
T2-L2	blue	Csa3G730930	1.847	0.00004243	Transport	Ammonium transporter 2 (AMT2)	8E-220
T2-L2	blue	Csa6G493850	1.86	1.50E-05	Not assigned	unknown	
T2-L2	blue	Csa3G159420	1.871	1.61E-03	Not assigned	unknown	
T2-L2	blue	Csa1G528610	1.874	0.00435	Lipid metabolism	Thylakoid membrane anion transporter 1 (ANTR1)	7.00E-236
T2-L2	blue	Csa1G181360	1.875	0.001947	Transport	non-intrinsic ABC protein 9 (NAP9)	3.00E-156
T2-L2	blue	Csa3G154410	2.05	1.63E-10	Not assigned	unknown	
T2-L2	blue	Csa3G183950	2.092	0.0004285	Abiotic stress	Heat shock protein 83	0.00E+00
T2-L2	blue	Csa2G296090	2.143	2.44E-04	Not assigned	unknown	
T2-L2	blue	Csa7G291720	2.169	0.006566	Lipid metabolism	Choline kinase	4.00E-133
T2-L2	blue	Csa2G427310	2.238	0.007581	Transcription factor	MYB domain protein 73 (MYB73)	1.00E-88
T2-L2	blue	Csa3G881750	2.24	0.009561	Lipid metabolism	PR5-like receptor kinase (PR5K)	4.00E-154
T2-L2	blue	Csa5G589260	2.243	0	Amino acid metabolism	O-acetylserine(thiol) lyase (OAS-TL)	4.00E-162
T2-L2	blue	Csa3G733350	2.317	0.00004435	Cell organization	RHO guanyl-nucleotide exchange factor 11 (ROPGEF11)	9.00E-41
T2-L2	blue	Csa2G004690	2.522	0.000004593	Transcription factor	Heat shock transcription factor C1 (HSFC1)	5.00E-67
T2-L2	blue	Csa7G387690	2.631	0.000001654	Transport	Sulfate transporter 3;1 (SULTR 3;1)	3.00E-307
T2-L2	blue	Csa1G008520	2.771	0.00804	Transport	Plasma membrane proton ATPase 4 (HA4)	0.00E+00
T2-L2	blue	Csa6G514850	3.048	0.00009803	Miscellaneous	Cytochrome P450 family 77 subfamily B polypeptide 1 (CYP77B1)	1.00E-241
T2-L2	blue	Csa5G604150	3.14	0.01	Redox	Thioredoxin 2 (TRX2)	
T2-L2	blue	Csa5G223070	3.216	0.003151	Transcription factor	WRKY DNA-binding protein 54 (WRKY54)	3.00E-96
T2-L2	blue	Csa2G359890	3.289	3.70E-03	Not assigned	unknown	
T2-L2	blue	Csa1G596520	3.4	0.0002165	Protein degradation	Cystein proteinases superfamily protein	2.00E-168
T2-L2	blue	Csa7G395800	3.415	0.001823	Miscellaneous	Glutathione S-transferase phi 8 (GSTF8)	3.00E-82
T2-L2	blue	Csa3G119700	3.575	0.00005376	Transcription factor	WRKY DNA-binding protein 53 (WRKY53)	1.00E-102
T2-L2	blue	Csa6G381850	3.831	0.001156	Transport	Amino acid permease 3 (AAP3)	2.00E-234
T2-L2	blue	Csa1G033160	4.495	0.006049	Miscellaneous	Glutathione S-transferase tau 9 (GSTU9)	3.00E-68
T2-L2	blue	Csa6G486960	4.496	0.000002502	Transcription factor	WRKY DNA-binding protein 51 (WRKY51)	1.00E-36
T2-L2	blue	Csa2G058620	5.345	0.003945	Abiotic stress	DNAJ heat shock N-terminal domain-containing protein	2.00E-137

Supplementary Table 1 (Continued)

T2-L2	blue	Csa1G574970	1.827	1.18E-05	Major CHO metabolism	Hexokinase 2 (HXK2)	7.00E-51
T2-L2	blue	Csa4G618520	1.837	0.0001765	Biodegradation of xenobiotic	UDP glycosyltransferase (UGT)	1.00E-132
T2-L2	blue	Csa2G003610	1.841	0.0003098	Lipid metabolism	Abnormal inflorescence meristem (AIM1)	3.00E-294
T2-L2	blue	Csa3G730930	1.847	0.00004243	Transport	Ammonium transporter 2 (AMT2)	8E-220
T2-L2	blue	Csa6G493850	1.86	1.50E-05	Not assigned	unknown	
T2-L2	blue	Csa3G159420	1.871	1.61E-03	Not assigned	unknown	
T2-L2	blue	Csa1G528610	1.874	0.00435	Lipid metabolism	Thylakoid membrane anion transporter 1 (ANTR1)	7.00E-236
T2-L2	blue	Csa1G181360	1.875	0.001947	Transport	non-intrinsic ABC protein 9 (NAP9)	3.00E-156
T2-L2	blue	Csa3G154410	2.05	1.63E-10	Not assigned	unknown	
T2-L2	blue	Csa3G183950	2.092	0.0004285	Abiotic stress	Heat shock protein 83	0.00E+00
T2-L2	blue	Csa2G296090	2.143	2.44E-04	Not assigned	unknown	
T2-L2	blue	Csa7G291720	2.169	0.006566	Lipid metabolism	Choline kinase	4.00E-133
T2-L2	blue	Csa2G427310	2.238	0.007581	Transcription factor	MYB domain protein 73 (MYB73)	1.00E-88
T2-L2	blue	Csa3G881750	2.24	0.009561	Lipid metabolism	PR5-like receptor kinase (PR5K)	4.00E-154
T2-L2	blue	Csa5G589260	2.243	0	Amino acid metabolism	O-acetylserine(thiol) lyase (OAS-TL)	4.00E-162
T2-L2	blue	Csa3G733350	2.317	0.00004435	Cell organization	RHO guanyl-nucleotide exchange factor 11 (ROPGEF11)	9.00E-41
T2-L2	blue	Csa2G004690	2.522	0.000004593	Transcription factor	Heat shock transcription factor C1 (HSFC1)	5.00E-67
T2-L2	blue	Csa7G387690	2.631	0.000001654	Transport	Sulfate transporter 3;1 (SULTR 3;1)	3.00E-307
T2-L2	blue	Csa1G008520	2.771	0.00804	Transport	Plasma membrane proton ATPase 4 (HA4)	0.00E+00
T2-L2	blue	Csa6G514850	3.048	0.00009803	Miscellaneous	Cytochrome P450 family 77 subfamily B polypeptide 1 (CYP77B1)	1.00E-241
T2-L2	blue	Csa5G604150	3.14	0.01	Redox	Thioredoxin 2 (TRX2)	
T2-L2	blue	Csa5G223070	3.216	0.003151	Transcription factor	WRKY DNA-binding protein 54 (WRKY54)	3.00E-96
T2-L2	blue	Csa2G359890	3.289	3.70E-03	Not assigned	unknown	
T2-L2	blue	Csa1G596520	3.4	0.0002165	Protein degradation	Cystein proteinases superfamily protein	2.00E-168
T2-L2	blue	Csa7G395800	3.415	0.001823	Miscellaneous	Glutathione S-transferase phi 8 (GSTF8)	3.00E-82
T2-L2	blue	Csa3G119700	3.575	0.00005376	Transcription factor	WRKY DNA-binding protein 53 (WRKY53)	1.00E-102
T2-L2	blue	Csa6G381850	3.831	0.001156	Transport	Amino acid permease 3 (AAP3)	2.00E-234
T2-L2	blue	Csa1G033160	4.495	0.006049	Miscellaneous	Glutathione S-transferase tau 9 (GSTU9)	3.00E-68
T2-L2	blue	Csa6G486960	4.496	0.000002502	Transcription factor	WRKY DNA-binding protein 51 (WRKY51)	1.00E-36
T2-L2	blue	Csa2G058620	5.345	0.003945	Abiotic stress	DNAJ heat shock N-terminal domain-containing protein	2.00E-137

Supplementary Table 1 (Continued)

T2-L2	blue	Csa1G574970	1.827	1.18E-05	Major CHO metabolism	Hexokinase 2 (HXK2)	7.00E-51
T2-L2	blue	Csa4G618520	1.837	0.0001765	Biodegradation of xenobiotic	UDP glycosyltransferase (UGT)	1.00E-132
T2-L2	blue	Csa2G003610	1.841	0.0003098	Lipid metabolism	Abnormal inflorescence meristem (AIM1)	3.00E-294
T2-L2	blue	Csa3G730930	1.847	0.00004243	Transport	Ammonium transporter 2 (AMT2)	8E-220
T2-L2	blue	Csa6G493850	1.86	1.50E-05	Not assigned	unknown	
T2-L2	blue	Csa3G159420	1.871	1.61E-03	Not assigned	unknown	
T2-L2	blue	Csa1G528610	1.874	0.00435	Lipid metabolism	Thylakoid membrane anion transporter 1 (ANTR1)	7.00E-236
T2-L2	blue	Csa1G181360	1.875	0.001947	Transport	non-intrinsic ABC protein 9 (NAP9)	3.00E-156
T2-L2	blue	Csa3G154410	2.05	1.63E-10	Not assigned	unknown	
T2-L2	blue	Csa3G183950	2.092	0.0004285	Abiotic stress	Heat shock protein 83	0.00E+00
T2-L2	blue	Csa2G296090	2.143	2.44E-04	Not assigned	unknown	
T2-L2	blue	Csa7G291720	2.169	0.006566	Lipid metabolism	Choline kinase	4.00E-133
T2-L2	blue	Csa2G427310	2.238	0.007581	Transcription factor	MYB domain protein 73 (MYB73)	1.00E-88
T2-L2	blue	Csa3G881750	2.24	0.009561	Lipid metabolism	PR5-like receptor kinase (PR5K)	4.00E-154
T2-L2	blue	Csa5G589260	2.243	0	Amino acid metabolism	O-acetylserine(thiol) lyase (OAS-TL)	4.00E-162
T2-L2	blue	Csa3G733350	2.317	0.00004435	Cell organization	RHO guanyl-nucleotide exchange factor 11 (ROPGEF11)	9.00E-41
T2-L2	blue	Csa2G004690	2.522	0.000004593	Transcription factor	Heat shock transcription factor C1 (HSFC1)	5.00E-67
T2-L2	blue	Csa7G387690	2.631	0.000001654	Transport	Sulfate transporter 3;1 (SULTR 3;1)	3.00E-307
T2-L2	blue	Csa1G008520	2.771	0.00804	Transport	Plasma membrane proton ATPase 4 (HA4)	0.00E+00
T2-L2	blue	Csa6G514850	3.048	0.00009803	Miscellaneous	Cytochrome P450 family 77 subfamily B polypeptide 1 (CYP77B1)	1.00E-241
T2-L2	blue	Csa5G604150	3.14	0.01	Redox	Thioredoxin 2 (TRX2)	
T2-L2	blue	Csa5G223070	3.216	0.003151	Transcription factor	WRKY DNA-binding protein 54 (WRKY54)	3.00E-96
T2-L2	blue	Csa2G359890	3.289	3.70E-03	Not assigned	unknown	
T2-L2	blue	Csa1G596520	3.4	0.0002165	Protein degradation	Cystein proteinases superfamily protein	2.00E-168
T2-L2	blue	Csa7G395800	3.415	0.001823	Miscellaneous	Glutathione S-transferase phi 8 (GSTF8)	3.00E-82
T2-L2	blue	Csa3G119700	3.575	0.00005376	Transcription factor	WRKY DNA-binding protein 53 (WRKY53)	1.00E-102
T2-L2	blue	Csa6G381850	3.831	0.001156	Transport	Amino acid permease 3 (AAP3)	2.00E-234
T2-L2	blue	Csa1G033160	4.495	0.006049	Miscellaneous	Glutathione S-transferase tau 9 (GSTU9)	3.00E-68
T2-L2	blue	Csa6G486960	4.496	0.000002502	Transcription factor	WRKY DNA-binding protein 51 (WRKY51)	1.00E-36
T2-L2	blue	Csa2G058620	5.345	0.003945	Abiotic stress	DNAJ heat shock N-terminal domain-containing protein	2.00E-137

Supplementary Table 1 (Continued)

T2-L2	blue	Csa1G574970	1.827	1.18E-05	Major CHO metabolism	Hexokinase 2 (HXK2)	7.00E-51
T2-L2	blue	Csa4G618520	1.837	0.0001765	Biodegradation of xenobiotic	UDP glycosyltransferase (UGT)	1.00E-132
T2-L2	blue	Csa2G003610	1.841	0.0003098	Lipid metabolism	Abnormal inflorescence meristem (AIM1)	3.00E-294
T2-L2	blue	Csa3G730930	1.847	0.00004243	Transport	Ammonium transporter 2 (AMT2)	8E-220
T2-L2	blue	Csa6G493850	1.86	1.50E-05	Not assigned	unknown	
T2-L2	blue	Csa3G159420	1.871	1.61E-03	Not assigned	unknown	
T2-L2	blue	Csa1G528610	1.874	0.00435	Lipid metabolism	Thylakoid membrane anion transporter 1 (ANTR1)	7.00E-236
T2-L2	blue	Csa1G181360	1.875	0.001947	Transport	non-intrinsic ABC protein 9 (NAP9)	3.00E-156
T2-L2	blue	Csa3G154410	2.05	1.63E-10	Not assigned	unknown	
T2-L2	blue	Csa3G183950	2.092	0.0004285	Abiotic stress	Heat shock protein 83	0.00E+00
T2-L2	blue	Csa2G296090	2.143	2.44E-04	Not assigned	unknown	
T2-L2	blue	Csa7G291720	2.169	0.006566	Lipid metabolism	Choline kinase	4.00E-133
T2-L2	blue	Csa2G427310	2.238	0.007581	Transcription factor	MYB domain protein 73 (MYB73)	1.00E-88
T2-L2	blue	Csa3G881750	2.24	0.009561	Lipid metabolism	PR5-like receptor kinase (PR5K)	4.00E-154
T2-L2	blue	Csa5G589260	2.243	0	Amino acid metabolism	O-acetylserine(thiol) lyase (OAS-TL)	4.00E-162
T2-L2	blue	Csa3G733350	2.317	0.00004435	Cell organization	RHO guanyl-nucleotide exchange factor 11 (ROPGEF11)	9.00E-41
T2-L2	blue	Csa2G004690	2.522	0.000004593	Transcription factor	Heat shock transcription factor C1 (HSFC1)	5.00E-67
T2-L2	blue	Csa7G387690	2.631	0.000001654	Transport	Sulfate transporter 3;1 (SULTR 3;1)	3.00E-307
T2-L2	blue	Csa1G008520	2.771	0.00804	Transport	Plasma membrane proton ATPase 4 (HA4)	0.00E+00
T2-L2	blue	Csa6G514850	3.048	0.00009803	Miscellaneous	Cytochrome P450 family 77 subfamily B polypeptide 1 (CYP77B1)	1.00E-241
T2-L2	blue	Csa5G604150	3.14	0.01	Redox	Thioredoxin 2 (TRX2)	
T2-L2	blue	Csa5G223070	3.216	0.003151	Transcription factor	WRKY DNA-binding protein 54 (WRKY54)	3.00E-96
T2-L2	blue	Csa2G359890	3.289	3.70E-03	Not assigned	unknown	
T2-L2	blue	Csa1G596520	3.4	0.0002165	Protein degradation	Cystein proteinases superfamily protein	2.00E-168
T2-L2	blue	Csa7G395800	3.415	0.001823	Miscellaneous	Glutathione S-transferase phi 8 (GSTF8)	3.00E-82
T2-L2	blue	Csa3G119700	3.575	0.00005376	Transcription factor	WRKY DNA-binding protein 53 (WRKY53)	1.00E-102
T2-L2	blue	Csa6G381850	3.831	0.001156	Transport	Amino acid permease 3 (AAP3)	2.00E-234
T2-L2	blue	Csa1G033160	4.495	0.006049	Miscellaneous	Glutathione S-transferase tau 9 (GSTU9)	3.00E-68
T2-L2	blue	Csa6G486960	4.496	0.000002502	Transcription factor	WRKY DNA-binding protein 51 (WRKY51)	1.00E-36
T2-L2	blue	Csa2G058620	5.345	0.003945	Abiotic stress	DNAJ heat shock N-terminal domain-containing protein	2.00E-137

Supplementary Table 1 (Continued)

T2-L2	blue	Csa1G574970	1.827	1.18E-05	Major CHO metabolism	Hexokinase 2 (HXK2)	7.00E-51
T2-L2	blue	Csa4G618520	1.837	0.0001765	Biodegradation of xenobiotic	UDP glycosyltransferase (UGT)	1.00E-132
T2-L2	blue	Csa2G003610	1.841	0.0003098	Lipid metabolism	Abnormal inflorescence meristem (AIM1)	3.00E-294
T2-L2	blue	Csa3G730930	1.847	0.00004243	Transport	Ammonium transporter 2 (AMT2)	8E-220
T2-L2	blue	Csa6G493850	1.86	1.50E-05	Not assigned	unknown	
T2-L2	blue	Csa3G159420	1.871	1.61E-03	Not assigned	unknown	
T2-L2	blue	Csa1G528610	1.874	0.00435	Lipid metabolism	Thylakoid membrane anion transporter 1 (ANTR1)	7.00E-236
T2-L2	blue	Csa1G181360	1.875	0.001947	Transport	non-intrinsic ABC protein 9 (NAP9)	3.00E-156
T2-L2	blue	Csa3G154410	2.05	1.63E-10	Not assigned	unknown	
T2-L2	blue	Csa3G183950	2.092	0.0004285	Abiotic stress	Heat shock protein 83	0.00E+00
T2-L2	blue	Csa2G296090	2.143	2.44E-04	Not assigned	unknown	
T2-L2	blue	Csa7G291720	2.169	0.006566	Lipid metabolism	Choline kinase	4.00E-133
T2-L2	blue	Csa2G427310	2.238	0.007581	Transcription factor	MYB domain protein 73 (MYB73)	1.00E-88
T2-L2	blue	Csa3G881750	2.24	0.009561	Lipid metabolism	PR5-like receptor kinase (PR5K)	4.00E-154
T2-L2	blue	Csa5G589260	2.243	0	Amino acid metabolism	O-acetylserine(thiol) lyase (OAS-TL)	4.00E-162
T2-L2	blue	Csa3G733350	2.317	0.00004435	Cell organization	RHO guanyl-nucleotide exchange factor 11 (ROPGEF11)	9.00E-41
T2-L2	blue	Csa2G004690	2.522	0.000004593	Transcription factor	Heat shock transcription factor C1 (HSFC1)	5.00E-67
T2-L2	blue	Csa7G387690	2.631	0.000001654	Transport	Sulfate transporter 3;1 (SULTR 3;1)	3.00E-307
T2-L2	blue	Csa1G008520	2.771	0.00804	Transport	Plasma membrane proton ATPase 4 (HA4)	0.00E+00
T2-L2	blue	Csa6G514850	3.048	0.00009803	Miscellaneous	Cytochrome P450 family 77 subfamily B polypeptide 1 (CYP77B1)	1.00E-241
T2-L2	blue	Csa5G604150	3.14	0.01	Redox	Thioredoxin 2 (TRX2)	
T2-L2	blue	Csa5G223070	3.216	0.003151	Transcription factor	WRKY DNA-binding protein 54 (WRKY54)	3.00E-96
T2-L2	blue	Csa2G359890	3.289	3.70E-03	Not assigned	unknown	
T2-L2	blue	Csa1G596520	3.4	0.0002165	Protein degradation	Cystein proteinases superfamily protein	2.00E-168
T2-L2	blue	Csa7G395800	3.415	0.001823	Miscellaneous	Glutathione S-transferase phi 8 (GSTF8)	3.00E-82
T2-L2	blue	Csa3G119700	3.575	0.00005376	Transcription factor	WRKY DNA-binding protein 53 (WRKY53)	1.00E-102
T2-L2	blue	Csa6G381850	3.831	0.001156	Transport	Amino acid permease 3 (AAP3)	2.00E-234
T2-L2	blue	Csa1G033160	4.495	0.006049	Miscellaneous	Glutathione S-transferase tau 9 (GSTU9)	3.00E-68
T2-L2	blue	Csa6G486960	4.496	0.000002502	Transcription factor	WRKY DNA-binding protein 51 (WRKY51)	1.00E-36
T2-L2	blue	Csa2G058620	5.345	0.003945	Abiotic stress	DNAJ heat shock N-terminal domain-containing protein	2.00E-137

Supplementary Table 1 (Continued)

T2-L2	blue	Csa1G574970	1.827	1.18E-05	Major CHO metabolism	Hexokinase 2 (HXK2)	7.00E-51
T2-L2	blue	Csa4G618520	1.837	0.0001765	Biodegradation of xenobiotic	UDP glycosyltransferase (UGT)	1.00E-132
T2-L2	blue	Csa2G003610	1.841	0.0003098	Lipid metabolism	Abnormal inflorescence meristem (AIM1)	3.00E-294
T2-L2	blue	Csa3G730930	1.847	0.00004243	Transport	Ammonium transporter 2 (AMT2)	8E-220
T2-L2	blue	Csa6G493850	1.86	1.50E-05	Not assigned	unknown	
T2-L2	blue	Csa3G159420	1.871	1.61E-03	Not assigned	unknown	
T2-L2	blue	Csa1G528610	1.874	0.00435	Lipid metabolism	Thylakoid membrane anion transporter 1 (ANTR1)	7.00E-236
T2-L2	blue	Csa1G181360	1.875	0.001947	Transport	non-intrinsic ABC protein 9 (NAP9)	3.00E-156
T2-L2	blue	Csa3G154410	2.05	1.63E-10	Not assigned	unknown	
T2-L2	blue	Csa3G183950	2.092	0.0004285	Abiotic stress	Heat shock protein 83	0.00E+00
T2-L2	blue	Csa2G296090	2.143	2.44E-04	Not assigned	unknown	
T2-L2	blue	Csa7G291720	2.169	0.006566	Lipid metabolism	Choline kinase	4.00E-133
T2-L2	blue	Csa2G427310	2.238	0.007581	Transcription factor	MYB domain protein 73 (MYB73)	1.00E-88
T2-L2	blue	Csa3G881750	2.24	0.009561	Lipid metabolism	PR5-like receptor kinase (PR5K)	4.00E-154
T2-L2	blue	Csa5G589260	2.243	0	Amino acid metabolism	O-acetylserine(thiol) lyase (OAS-TL)	4.00E-162
T2-L2	blue	Csa3G733350	2.317	0.00004435	Cell organization	RHO guanyl-nucleotide exchange factor 11 (ROPGEF11)	9.00E-41
T2-L2	blue	Csa2G004690	2.522	0.000004593	Transcription factor	Heat shock transcription factor C1 (HSFC1)	5.00E-67
T2-L2	blue	Csa7G387690	2.631	0.000001654	Transport	Sulfate transporter 3;1 (SULTR 3;1)	3.00E-307
T2-L2	blue	Csa1G008520	2.771	0.00804	Transport	Plasma membrane proton ATPase 4 (HA4)	0.00E+00
T2-L2	blue	Csa6G514850	3.048	0.00009803	Miscellaneous	Cytochrome P450 family 77 subfamily B polypeptide 1 (CYP77B1)	1.00E-241
T2-L2	blue	Csa5G604150	3.14	0.01	Redox	Thioredoxin 2 (TRX2)	
T2-L2	blue	Csa5G223070	3.216	0.003151	Transcription factor	WRKY DNA-binding protein 54 (WRKY54)	3.00E-96
T2-L2	blue	Csa2G359890	3.289	3.70E-03	Not assigned	unknown	
T2-L2	blue	Csa1G596520	3.4	0.0002165	Protein degradation	Cystein proteinases superfamily protein	2.00E-168
T2-L2	blue	Csa7G395800	3.415	0.001823	Miscellaneous	Glutathione S-transferase phi 8 (GSTF8)	3.00E-82
T2-L2	blue	Csa3G119700	3.575	0.00005376	Transcription factor	WRKY DNA-binding protein 53 (WRKY53)	1.00E-102
T2-L2	blue	Csa6G381850	3.831	0.001156	Transport	Amino acid permease 3 (AAP3)	2.00E-234
T2-L2	blue	Csa1G033160	4.495	0.006049	Miscellaneous	Glutathione S-transferase tau 9 (GSTU9)	3.00E-68
T2-L2	blue	Csa6G486960	4.496	0.000002502	Transcription factor	WRKY DNA-binding protein 51 (WRKY51)	1.00E-36
T2-L2	blue	Csa2G058620	5.345	0.003945	Abiotic stress	DNAJ heat shock N-terminal domain-containing protein	2.00E-137

Supplementary Table 1 (Continued)

T2-L2	blue	Csa1G574970	1.827	1.18E-05	Major CHO metabolism	Hexokinase 2 (HXK2)	7.00E-51
T2-L2	blue	Csa4G618520	1.837	0.0001765	Biodegradation of xenobiotic	UDP glycosyltransferase (UGT)	1.00E-132
T2-L2	blue	Csa2G003610	1.841	0.0003098	Lipid metabolism	Abnormal inflorescence meristem (AIM1)	3.00E-294
T2-L2	blue	Csa3G730930	1.847	0.00004243	Transport	Ammonium transporter 2 (AMT2)	8E-220
T2-L2	blue	Csa6G493850	1.86	1.50E-05	Not assigned	unknown	
T2-L2	blue	Csa3G159420	1.871	1.61E-03	Not assigned	unknown	
T2-L2	blue	Csa1G528610	1.874	0.00435	Lipid metabolism	Thylakoid membrane anion transporter 1 (ANTR1)	7.00E-236
T2-L2	blue	Csa1G181360	1.875	0.001947	Transport	non-intrinsic ABC protein 9 (NAP9)	3.00E-156
T2-L2	blue	Csa3G154410	2.05	1.63E-10	Not assigned	unknown	
T2-L2	blue	Csa3G183950	2.092	0.0004285	Abiotic stress	Heat shock protein 83	0.00E+00
T2-L2	blue	Csa2G296090	2.143	2.44E-04	Not assigned	unknown	
T2-L2	blue	Csa7G291720	2.169	0.006566	Lipid metabolism	Choline kinase	4.00E-133
T2-L2	blue	Csa2G427310	2.238	0.007581	Transcription factor	MYB domain protein 73 (MYB73)	1.00E-88
T2-L2	blue	Csa3G881750	2.24	0.009561	Lipid metabolism	PR5-like receptor kinase (PR5K)	4.00E-154
T2-L2	blue	Csa5G589260	2.243	0	Amino acid metabolism	O-acetylserine(thiol) lyase (OAS-TL)	4.00E-162
T2-L2	blue	Csa3G733350	2.317	0.00004435	Cell organization	RHO guanyl-nucleotide exchange factor 11 (ROPGEF11)	9.00E-41
T2-L2	blue	Csa2G004690	2.522	0.000004593	Transcription factor	Heat shock transcription factor C1 (HSFC1)	5.00E-67
T2-L2	blue	Csa7G387690	2.631	0.000001654	Transport	Sulfate transporter 3;1 (SULTR 3;1)	3.00E-307
T2-L2	blue	Csa1G008520	2.771	0.00804	Transport	Plasma membrane proton ATPase 4 (HA4)	0.00E+00
T2-L2	blue	Csa6G514850	3.048	0.00009803	Miscellaneous	Cytochrome P450 family 77 subfamily B polypeptide 1 (CYP77B1)	1.00E-241
T2-L2	blue	Csa5G604150	3.14	0.01	Redox	Thioredoxin 2 (TRX2)	
T2-L2	blue	Csa5G223070	3.216	0.003151	Transcription factor	WRKY DNA-binding protein 54 (WRKY54)	3.00E-96
T2-L2	blue	Csa2G359890	3.289	3.70E-03	Not assigned	unknown	
T2-L2	blue	Csa1G596520	3.4	0.0002165	Protein degradation	Cystein proteinases superfamily protein	2.00E-168
T2-L2	blue	Csa7G395800	3.415	0.001823	Miscellaneous	Glutathione S-transferase phi 8 (GSTF8)	3.00E-82
T2-L2	blue	Csa3G119700	3.575	0.00005376	Transcription factor	WRKY DNA-binding protein 53 (WRKY53)	1.00E-102
T2-L2	blue	Csa6G381850	3.831	0.001156	Transport	Amino acid permease 3 (AAP3)	2.00E-234
T2-L2	blue	Csa1G033160	4.495	0.006049	Miscellaneous	Glutathione S-transferase tau 9 (GSTU9)	3.00E-68
T2-L2	blue	Csa6G486960	4.496	0.000002502	Transcription factor	WRKY DNA-binding protein 51 (WRKY51)	1.00E-36
T2-L2	blue	Csa2G058620	5.345	0.003945	Abiotic stress	DNAJ heat shock N-terminal domain-containing protein	2.00E-137

Supplementary Table 1 (Continued)

T2-L2	blue	Csa1G574970	1.827	1.18E-05	Major CHO metabolism	Hexokinase 2 (HXK2)	7.00E-51
T2-L2	blue	Csa4G618520	1.837	0.0001765	Biodegradation of xenobiotic	UDP glycosyltransferase (UGT)	1.00E-132
T2-L2	blue	Csa2G003610	1.841	0.0003098	Lipid metabolism	Abnormal inflorescence meristem (AIM1)	3.00E-294
T2-L2	blue	Csa3G730930	1.847	0.00004243	Transport	Ammonium transporter 2 (AMT2)	8E-220
T2-L2	blue	Csa6G493850	1.86	1.50E-05	Not assigned	unknown	
T2-L2	blue	Csa3G159420	1.871	1.61E-03	Not assigned	unknown	
T2-L2	blue	Csa1G528610	1.874	0.00435	Lipid metabolism	Thylakoid membrane anion transporter 1 (ANTR1)	7.00E-236
T2-L2	blue	Csa1G181360	1.875	0.001947	Transport	non-intrinsic ABC protein 9 (NAP9)	3.00E-156
T2-L2	blue	Csa3G154410	2.05	1.63E-10	Not assigned	unknown	
T2-L2	blue	Csa3G183950	2.092	0.0004285	Abiotic stress	Heat shock protein 83	0.00E+00
T2-L2	blue	Csa2G296090	2.143	2.44E-04	Not assigned	unknown	
T2-L2	blue	Csa7G291720	2.169	0.006566	Lipid metabolism	Choline kinase	4.00E-133
T2-L2	blue	Csa2G427310	2.238	0.007581	Transcription factor	MYB domain protein 73 (MYB73)	1.00E-88
T2-L2	blue	Csa3G881750	2.24	0.009561	Lipid metabolism	PR5-like receptor kinase (PR5K)	4.00E-154
T2-L2	blue	Csa5G589260	2.243	0	Amino acid metabolism	O-acetylserine(thiol) lyase (OAS-TL)	4.00E-162
T2-L2	blue	Csa3G733350	2.317	0.00004435	Cell organization	RHO guanyl-nucleotide exchange factor 11 (ROPGEF11)	9.00E-41
T2-L2	blue	Csa2G004690	2.522	0.000004593	Transcription factor	Heat shock transcription factor C1 (HSFC1)	5.00E-67
T2-L2	blue	Csa7G387690	2.631	0.000001654	Transport	Sulfate transporter 3;1 (SULTR 3;1)	3.00E-307
T2-L2	blue	Csa1G008520	2.771	0.00804	Transport	Plasma membrane proton ATPase 4 (HA4)	0.00E+00
T2-L2	blue	Csa6G514850	3.048	0.00009803	Miscellaneous	Cytochrome P450 family 77 subfamily B polypeptide 1 (CYP77B1)	1.00E-241
T2-L2	blue	Csa5G604150	3.14	0.01	Redox	Thioredoxin 2 (TRX2)	
T2-L2	blue	Csa5G223070	3.216	0.003151	Transcription factor	WRKY DNA-binding protein 54 (WRKY54)	3.00E-96
T2-L2	blue	Csa2G359890	3.289	3.70E-03	Not assigned	unknown	
T2-L2	blue	Csa1G596520	3.4	0.0002165	Protein degradation	Cystein proteinases superfamily protein	2.00E-168
T2-L2	blue	Csa7G395800	3.415	0.001823	Miscellaneous	Glutathione S-transferase phi 8 (GSTF8)	3.00E-82
T2-L2	blue	Csa3G119700	3.575	0.00005376	Transcription factor	WRKY DNA-binding protein 53 (WRKY53)	1.00E-102
T2-L2	blue	Csa6G381850	3.831	0.001156	Transport	Amino acid permease 3 (AAP3)	2.00E-234
T2-L2	blue	Csa1G033160	4.495	0.006049	Miscellaneous	Glutathione S-transferase tau 9 (GSTU9)	3.00E-68
T2-L2	blue	Csa6G486960	4.496	0.000002502	Transcription factor	WRKY DNA-binding protein 51 (WRKY51)	1.00E-36
T2-L2	blue	Csa2G058620	5.345	0.003945	Abiotic stress	DNAJ heat shock N-terminal domain-containing protein	2.00E-137

Supplementary Table 1 (Continued)

T2-L2	blue	Csa1G574970	1.827	1.18E-05	Major CHO metabolism	Hexokinase 2 (HXK2)	7.00E-51
T2-L2	blue	Csa4G618520	1.837	0.0001765	Biodegradation of xenobiotic	UDP glycosyltransferase (UGT)	1.00E-132
T2-L2	blue	Csa2G003610	1.841	0.0003098	Lipid metabolism	Abnormal inflorescence meristem (AIM1)	3.00E-294
T2-L2	blue	Csa3G730930	1.847	0.00004243	Transport	Ammonium transporter 2 (AMT2)	8E-220
T2-L2	blue	Csa6G493850	1.86	1.50E-05	Not assigned	unknown	
T2-L2	blue	Csa3G159420	1.871	1.61E-03	Not assigned	unknown	
T2-L2	blue	Csa1G528610	1.874	0.00435	Lipid metabolism	Thylakoid membrane anion transporter 1 (ANTR1)	7.00E-236
T2-L2	blue	Csa1G181360	1.875	0.001947	Transport	non-intrinsic ABC protein 9 (NAP9)	3.00E-156
T2-L2	blue	Csa3G154410	2.05	1.63E-10	Not assigned	unknown	
T2-L2	blue	Csa3G183950	2.092	0.0004285	Abiotic stress	Heat shock protein 83	0.00E+00
T2-L2	blue	Csa2G296090	2.143	2.44E-04	Not assigned	unknown	
T2-L2	blue	Csa7G291720	2.169	0.006566	Lipid metabolism	Choline kinase	4.00E-133
T2-L2	blue	Csa2G427310	2.238	0.007581	Transcription factor	MYB domain protein 73 (MYB73)	1.00E-88
T2-L2	blue	Csa3G881750	2.24	0.009561	Lipid metabolism	PR5-like receptor kinase (PR5K)	4.00E-154
T2-L2	blue	Csa5G589260	2.243	0	Amino acid metabolism	O-acetylserine(thiol) lyase (OAS-TL)	4.00E-162
T2-L2	blue	Csa3G733350	2.317	0.00004435	Cell organization	RHO guanyl-nucleotide exchange factor 11 (ROPGEF11)	9.00E-41
T2-L2	blue	Csa2G004690	2.522	0.000004593	Transcription factor	Heat shock transcription factor C1 (HSFC1)	5.00E-67
T2-L2	blue	Csa7G387690	2.631	0.000001654	Transport	Sulfate transporter 3;1 (SULTR 3;1)	3.00E-307
T2-L2	blue	Csa1G008520	2.771	0.00804	Transport	Plasma membrane proton ATPase 4 (HA4)	0.00E+00
T2-L2	blue	Csa6G514850	3.048	0.00009803	Miscellaneous	Cytochrome P450 family 77 subfamily B polypeptide 1 (CYP77B1)	1.00E-241
T2-L2	blue	Csa5G604150	3.14	0.01	Redox	Thioredoxin 2 (TRX2)	
T2-L2	blue	Csa5G223070	3.216	0.003151	Transcription factor	WRKY DNA-binding protein 54 (WRKY54)	3.00E-96
T2-L2	blue	Csa2G359890	3.289	3.70E-03	Not assigned	unknown	
T2-L2	blue	Csa1G596520	3.4	0.0002165	Protein degradation	Cystein proteinases superfamily protein	2.00E-168
T2-L2	blue	Csa7G395800	3.415	0.001823	Miscellaneous	Glutathione S-transferase phi 8 (GSTF8)	3.00E-82
T2-L2	blue	Csa3G119700	3.575	0.00005376	Transcription factor	WRKY DNA-binding protein 53 (WRKY53)	1.00E-102
T2-L2	blue	Csa6G381850	3.831	0.001156	Transport	Amino acid permease 3 (AAP3)	2.00E-234
T2-L2	blue	Csa1G033160	4.495	0.006049	Miscellaneous	Glutathione S-transferase tau 9 (GSTU9)	3.00E-68
T2-L2	blue	Csa6G486960	4.496	0.000002502	Transcription factor	WRKY DNA-binding protein 51 (WRKY51)	1.00E-36
T2-L2	blue	Csa2G058620	5.345	0.003945	Abiotic stress	DNAJ heat shock N-terminal domain-containing protein	2.00E-137

Supplementary Table 1 (Continued)

T2-L2	blue	Csa1G574970	1.827	1.18E-05	Major CHO metabolism	Hexokinase 2 (HXK2)	7.00E-51
T2-L2	blue	Csa4G618520	1.837	0.0001765	Biodegradation of xenobiotic	UDP glycosyltransferase (UGT)	1.00E-132
T2-L2	blue	Csa2G003610	1.841	0.0003098	Lipid metabolism	Abnormal inflorescence meristem (AIM1)	3.00E-294
T2-L2	blue	Csa3G730930	1.847	0.00004243	Transport	Ammonium transporter 2 (AMT2)	8E-220
T2-L2	blue	Csa6G493850	1.86	1.50E-05	Not assigned	unknown	
T2-L2	blue	Csa3G159420	1.871	1.61E-03	Not assigned	unknown	
T2-L2	blue	Csa1G528610	1.874	0.00435	Lipid metabolism	Thylakoid membrane anion transporter 1 (ANTR1)	7.00E-236
T2-L2	blue	Csa1G181360	1.875	0.001947	Transport	non-intrinsic ABC protein 9 (NAP9)	3.00E-156
T2-L2	blue	Csa3G154410	2.05	1.63E-10	Not assigned	unknown	
T2-L2	blue	Csa3G183950	2.092	0.0004285	Abiotic stress	Heat shock protein 83	0.00E+00
T2-L2	blue	Csa2G296090	2.143	2.44E-04	Not assigned	unknown	
T2-L2	blue	Csa7G291720	2.169	0.006566	Lipid metabolism	Choline kinase	4.00E-133
T2-L2	blue	Csa2G427310	2.238	0.007581	Transcription factor	MYB domain protein 73 (MYB73)	1.00E-88
T2-L2	blue	Csa3G881750	2.24	0.009561	Lipid metabolism	PR5-like receptor kinase (PR5K)	4.00E-154
T2-L2	blue	Csa5G589260	2.243	0	Amino acid metabolism	O-acetylserine(thiol) lyase (OAS-TL)	4.00E-162
T2-L2	blue	Csa3G733350	2.317	0.00004435	Cell organization	RHO guanyl-nucleotide exchange factor 11 (ROPGEF11)	9.00E-41
T2-L2	blue	Csa2G004690	2.522	0.000004593	Transcription factor	Heat shock transcription factor C1 (HSFC1)	5.00E-67
T2-L2	blue	Csa7G387690	2.631	0.000001654	Transport	Sulfate transporter 3;1 (SULTR 3;1)	3.00E-307
T2-L2	blue	Csa1G008520	2.771	0.00804	Transport	Plasma membrane proton ATPase 4 (HA4)	0.00E+00
T2-L2	blue	Csa6G514850	3.048	0.00009803	Miscellaneous	Cytochrome P450 family 77 subfamily B polypeptide 1 (CYP77B1)	1.00E-241
T2-L2	blue	Csa5G604150	3.14	0.01	Redox	Thioredoxin 2 (TRX2)	
T2-L2	blue	Csa5G223070	3.216	0.003151	Transcription factor	WRKY DNA-binding protein 54 (WRKY54)	3.00E-96
T2-L2	blue	Csa2G359890	3.289	3.70E-03	Not assigned	unknown	
T2-L2	blue	Csa1G596520	3.4	0.0002165	Protein degradation	Cystein proteinases superfamily protein	2.00E-168
T2-L2	blue	Csa7G395800	3.415	0.001823	Miscellaneous	Glutathione S-transferase phi 8 (GSTF8)	3.00E-82
T2-L2	blue	Csa3G119700	3.575	0.00005376	Transcription factor	WRKY DNA-binding protein 53 (WRKY53)	1.00E-102
T2-L2	blue	Csa6G381850	3.831	0.001156	Transport	Amino acid permease 3 (AAP3)	2.00E-234
T2-L2	blue	Csa1G033160	4.495	0.006049	Miscellaneous	Glutathione S-transferase tau 9 (GSTU9)	3.00E-68
T2-L2	blue	Csa6G486960	4.496	0.000002502	Transcription factor	WRKY DNA-binding protein 51 (WRKY51)	1.00E-36
T2-L2	blue	Csa2G058620	5.345	0.003945	Abiotic stress	DNAJ heat shock N-terminal domain-containing protein	2.00E-137

Supplementary Table 1 (Continued)

T2-L2	blue	Csa1G574970	1.827	1.18E-05	Major CHO metabolism	Hexokinase 2 (HXK2)	7.00E-51
T2-L2	blue	Csa4G618520	1.837	0.0001765	Biodegradation of xenobiotic	UDP glycosyltransferase (UGT)	1.00E-132
T2-L2	blue	Csa2G003610	1.841	0.0003098	Lipid metabolism	Abnormal inflorescence meristem (AIM1)	3.00E-294
T2-L2	blue	Csa3G730930	1.847	0.00004243	Transport	Ammonium transporter 2 (AMT2)	8E-220
T2-L2	blue	Csa6G493850	1.86	1.50E-05	Not assigned	unknown	
T2-L2	blue	Csa3G159420	1.871	1.61E-03	Not assigned	unknown	
T2-L2	blue	Csa1G528610	1.874	0.00435	Lipid metabolism	Thylakoid membrane anion transporter 1 (ANTR1)	7.00E-236
T2-L2	blue	Csa1G181360	1.875	0.001947	Transport	non-intrinsic ABC protein 9 (NAP9)	3.00E-156
T2-L2	blue	Csa3G154410	2.05	1.63E-10	Not assigned	unknown	
T2-L2	blue	Csa3G183950	2.092	0.0004285	Abiotic stress	Heat shock protein 83	0.00E+00
T2-L2	blue	Csa2G296090	2.143	2.44E-04	Not assigned	unknown	
T2-L2	blue	Csa7G291720	2.169	0.006566	Lipid metabolism	Choline kinase	4.00E-133
T2-L2	blue	Csa2G427310	2.238	0.007581	Transcription factor	MYB domain protein 73 (MYB73)	1.00E-88
T2-L2	blue	Csa3G881750	2.24	0.009561	Lipid metabolism	PR5-like receptor kinase (PR5K)	4.00E-154
T2-L2	blue	Csa5G589260	2.243	0	Amino acid metabolism	O-acetylserine(thiol) lyase (OAS-TL)	4.00E-162
T2-L2	blue	Csa3G733350	2.317	0.00004435	Cell organization	RHO guanyl-nucleotide exchange factor 11 (ROPGEF11)	9.00E-41
T2-L2	blue	Csa2G004690	2.522	0.000004593	Transcription factor	Heat shock transcription factor C1 (HSFC1)	5.00E-67
T2-L2	blue	Csa7G387690	2.631	0.000001654	Transport	Sulfate transporter 3;1 (SULTR 3;1)	3.00E-307
T2-L2	blue	Csa1G008520	2.771	0.00804	Transport	Plasma membrane proton ATPase 4 (HA4)	0.00E+00
T2-L2	blue	Csa6G514850	3.048	0.00009803	Miscellaneous	Cytochrome P450 family 77 subfamily B polypeptide 1 (CYP77B1)	1.00E-241
T2-L2	blue	Csa5G604150	3.14	0.01	Redox	Thioredoxin 2 (TRX2)	
T2-L2	blue	Csa5G223070	3.216	0.003151	Transcription factor	WRKY DNA-binding protein 54 (WRKY54)	3.00E-96
T2-L2	blue	Csa2G359890	3.289	3.70E-03	Not assigned	unknown	
T2-L2	blue	Csa1G596520	3.4	0.0002165	Protein degradation	Cystein proteinases superfamily protein	2.00E-168
T2-L2	blue	Csa7G395800	3.415	0.001823	Miscellaneous	Glutathione S-transferase phi 8 (GSTF8)	3.00E-82
T2-L2	blue	Csa3G119700	3.575	0.00005376	Transcription factor	WRKY DNA-binding protein 53 (WRKY53)	1.00E-102
T2-L2	blue	Csa6G381850	3.831	0.001156	Transport	Amino acid permease 3 (AAP3)	2.00E-234
T2-L2	blue	Csa1G033160	4.495	0.006049	Miscellaneous	Glutathione S-transferase tau 9 (GSTU9)	3.00E-68
T2-L2	blue	Csa6G486960	4.496	0.000002502	Transcription factor	WRKY DNA-binding protein 51 (WRKY51)	1.00E-36
T2-L2	blue	Csa2G058620	5.345	0.003945	Abiotic stress	DNAJ heat shock N-terminal domain-containing protein	2.00E-137

Supplementary Table 1 (Continued)

T2-L2	blue	Csa1G574970	1.827	1.18E-05	Major CHO metabolism	Hexokinase 2 (HXK2)	7.00E-51
T2-L2	blue	Csa4G618520	1.837	0.0001765	Biodegradation of xenobiotic	UDP glycosyltransferase (UGT)	1.00E-132
T2-L2	blue	Csa2G003610	1.841	0.0003098	Lipid metabolism	Abnormal inflorescence meristem (AIM1)	3.00E-294
T2-L2	blue	Csa3G730930	1.847	0.00004243	Transport	Ammonium transporter 2 (AMT2)	8E-220
T2-L2	blue	Csa6G493850	1.86	1.50E-05	Not assigned	unknown	
T2-L2	blue	Csa3G159420	1.871	1.61E-03	Not assigned	unknown	
T2-L2	blue	Csa1G528610	1.874	0.00435	Lipid metabolism	Thylakoid membrane anion transporter 1 (ANTR1)	7.00E-236
T2-L2	blue	Csa1G181360	1.875	0.001947	Transport	non-intrinsic ABC protein 9 (NAP9)	3.00E-156
T2-L2	blue	Csa3G154410	2.05	1.63E-10	Not assigned	unknown	
T2-L2	blue	Csa3G183950	2.092	0.0004285	Abiotic stress	Heat shock protein 83	0.00E+00
T2-L2	blue	Csa2G296090	2.143	2.44E-04	Not assigned	unknown	
T2-L2	blue	Csa7G291720	2.169	0.006566	Lipid metabolism	Choline kinase	4.00E-133
T2-L2	blue	Csa2G427310	2.238	0.007581	Transcription factor	MYB domain protein 73 (MYB73)	1.00E-88
T2-L2	blue	Csa3G881750	2.24	0.009561	Lipid metabolism	PR5-like receptor kinase (PR5K)	4.00E-154
T2-L2	blue	Csa5G589260	2.243	0	Amino acid metabolism	O-acetylserine(thiol) lyase (OAS-TL)	4.00E-162
T2-L2	blue	Csa3G733350	2.317	0.00004435	Cell organization	RHO guanyl-nucleotide exchange factor 11 (ROPGEF11)	9.00E-41
T2-L2	blue	Csa2G004690	2.522	0.000004593	Transcription factor	Heat shock transcription factor C1 (HSFC1)	5.00E-67
T2-L2	blue	Csa7G387690	2.631	0.000001654	Transport	Sulfate transporter 3;1 (SULTR 3;1)	3.00E-307
T2-L2	blue	Csa1G008520	2.771	0.00804	Transport	Plasma membrane proton ATPase 4 (HA4)	0.00E+00
T2-L2	blue	Csa6G514850	3.048	0.00009803	Miscellaneous	Cytochrome P450 family 77 subfamily B polypeptide 1 (CYP77B1)	1.00E-241
T2-L2	blue	Csa5G604150	3.14	0.01	Redox	Thioredoxin 2 (TRX2)	
T2-L2	blue	Csa5G223070	3.216	0.003151	Transcription factor	WRKY DNA-binding protein 54 (WRKY54)	3.00E-96
T2-L2	blue	Csa2G359890	3.289	3.70E-03	Not assigned	unknown	
T2-L2	blue	Csa1G596520	3.4	0.0002165	Protein degradation	Cystein proteinases superfamily protein	2.00E-168
T2-L2	blue	Csa7G395800	3.415	0.001823	Miscellaneous	Glutathione S-transferase phi 8 (GSTF8)	3.00E-82
T2-L2	blue	Csa3G119700	3.575	0.00005376	Transcription factor	WRKY DNA-binding protein 53 (WRKY53)	1.00E-102
T2-L2	blue	Csa6G381850	3.831	0.001156	Transport	Amino acid permease 3 (AAP3)	2.00E-234
T2-L2	blue	Csa1G033160	4.495	0.006049	Miscellaneous	Glutathione S-transferase tau 9 (GSTU9)	3.00E-68
T2-L2	blue	Csa6G486960	4.496	0.000002502	Transcription factor	WRKY DNA-binding protein 51 (WRKY51)	1.00E-36
T2-L2	blue	Csa2G058620	5.345	0.003945	Abiotic stress	DNAJ heat shock N-terminal domain-containing protein	2.00E-137

Supplementary Table 1 (Continued)

T2-L2	blue	Csa1G574970	1.827	1.18E-05	Major CHO metabolism	Hexokinase 2 (HXK2)	7.00E-51
T2-L2	blue	Csa4G618520	1.837	0.0001765	Biodegradation of xenobiotic	UDP glycosyltransferase (UGT)	1.00E-132
T2-L2	blue	Csa2G003610	1.841	0.0003098	Lipid metabolism	Abnormal inflorescence meristem (AIM1)	3.00E-294
T2-L2	blue	Csa3G730930	1.847	0.00004243	Transport	Ammonium transporter 2 (AMT2)	8E-220
T2-L2	blue	Csa6G493850	1.86	1.50E-05	Not assigned	unknown	
T2-L2	blue	Csa3G159420	1.871	1.61E-03	Not assigned	unknown	
T2-L2	blue	Csa1G528610	1.874	0.00435	Lipid metabolism	Thylakoid membrane anion transporter 1 (ANTR1)	7.00E-236
T2-L2	blue	Csa1G181360	1.875	0.001947	Transport	non-intrinsic ABC protein 9 (NAP9)	3.00E-156
T2-L2	blue	Csa3G154410	2.05	1.63E-10	Not assigned	unknown	
T2-L2	blue	Csa3G183950	2.092	0.0004285	Abiotic stress	Heat shock protein 83	0.00E+00
T2-L2	blue	Csa2G296090	2.143	2.44E-04	Not assigned	unknown	
T2-L2	blue	Csa7G291720	2.169	0.006566	Lipid metabolism	Choline kinase	4.00E-133
T2-L2	blue	Csa2G427310	2.238	0.007581	Transcription factor	MYB domain protein 73 (MYB73)	1.00E-88
T2-L2	blue	Csa3G881750	2.24	0.009561	Lipid metabolism	PR5-like receptor kinase (PR5K)	4.00E-154
T2-L2	blue	Csa5G589260	2.243	0	Amino acid metabolism	O-acetylserine(thiol) lyase (OAS-TL)	4.00E-162
T2-L2	blue	Csa3G733350	2.317	0.00004435	Cell organization	RHO guanyl-nucleotide exchange factor 11 (ROPGEF11)	9.00E-41
T2-L2	blue	Csa2G004690	2.522	0.000004593	Transcription factor	Heat shock transcription factor C1 (HSFC1)	5.00E-67
T2-L2	blue	Csa7G387690	2.631	0.000001654	Transport	Sulfate transporter 3;1 (SULTR 3;1)	3.00E-307
T2-L2	blue	Csa1G008520	2.771	0.00804	Transport	Plasma membrane proton ATPase 4 (HA4)	0.00E+00
T2-L2	blue	Csa6G514850	3.048	0.00009803	Miscellaneous	Cytochrome P450 family 77 subfamily B polypeptide 1 (CYP77B1)	1.00E-241
T2-L2	blue	Csa5G604150	3.14	0.01	Redox	Thioredoxin 2 (TRX2)	
T2-L2	blue	Csa5G223070	3.216	0.003151	Transcription factor	WRKY DNA-binding protein 54 (WRKY54)	3.00E-96
T2-L2	blue	Csa2G359890	3.289	3.70E-03	Not assigned	unknown	
T2-L2	blue	Csa1G596520	3.4	0.0002165	Protein degradation	Cystein proteinases superfamily protein	2.00E-168
T2-L2	blue	Csa7G395800	3.415	0.001823	Miscellaneous	Glutathione S-transferase phi 8 (GSTF8)	3.00E-82
T2-L2	blue	Csa3G119700	3.575	0.00005376	Transcription factor	WRKY DNA-binding protein 53 (WRKY53)	1.00E-102
T2-L2	blue	Csa6G381850	3.831	0.001156	Transport	Amino acid permease 3 (AAP3)	2.00E-234
T2-L2	blue	Csa1G033160	4.495	0.006049	Miscellaneous	Glutathione S-transferase tau 9 (GSTU9)	3.00E-68
T2-L2	blue	Csa6G486960	4.496	0.000002502	Transcription factor	WRKY DNA-binding protein 51 (WRKY51)	1.00E-36
T2-L2	blue	Csa2G058620	5.345	0.003945	Abiotic stress	DNAJ heat shock N-terminal domain-containing protein	2.00E-137

Supplementary Table 1 (Continued)

T2-L2	blue	Csa1G574970	1.827	1.18E-05	Major CHO metabolism	Hexokinase 2 (HXK2)	7.00E-51
T2-L2	blue	Csa4G618520	1.837	0.0001765	Biodegradation of xenobiotic	UDP glycosyltransferase (UGT)	1.00E-132
T2-L2	blue	Csa2G003610	1.841	0.0003098	Lipid metabolism	Abnormal inflorescence meristem (AIM1)	3.00E-294
T2-L2	blue	Csa3G730930	1.847	0.00004243	Transport	Ammonium transporter 2 (AMT2)	8E-220
T2-L2	blue	Csa6G493850	1.86	1.50E-05	Not assigned	unknown	
T2-L2	blue	Csa3G159420	1.871	1.61E-03	Not assigned	unknown	
T2-L2	blue	Csa1G528610	1.874	0.00435	Lipid metabolism	Thylakoid membrane anion transporter 1 (ANTR1)	7.00E-236
T2-L2	blue	Csa1G181360	1.875	0.001947	Transport	non-intrinsic ABC protein 9 (NAP9)	3.00E-156
T2-L2	blue	Csa3G154410	2.05	1.63E-10	Not assigned	unknown	
T2-L2	blue	Csa3G183950	2.092	0.0004285	Abiotic stress	Heat shock protein 83	0.00E+00
T2-L2	blue	Csa2G296090	2.143	2.44E-04	Not assigned	unknown	
T2-L2	blue	Csa7G291720	2.169	0.006566	Lipid metabolism	Choline kinase	4.00E-133
T2-L2	blue	Csa2G427310	2.238	0.007581	Transcription factor	MYB domain protein 73 (MYB73)	1.00E-88
T2-L2	blue	Csa3G881750	2.24	0.009561	Lipid metabolism	PR5-like receptor kinase (PR5K)	4.00E-154
T2-L2	blue	Csa5G589260	2.243	0	Amino acid metabolism	O-acetylserine(thiol) lyase (OAS-TL)	4.00E-162
T2-L2	blue	Csa3G733350	2.317	0.00004435	Cell organization	RHO guanyl-nucleotide exchange factor 11 (ROPGEF11)	9.00E-41
T2-L2	blue	Csa2G004690	2.522	0.000004593	Transcription factor	Heat shock transcription factor C1 (HSFC1)	5.00E-67
T2-L2	blue	Csa7G387690	2.631	0.000001654	Transport	Sulfate transporter 3;1 (SULTR 3;1)	3.00E-307
T2-L2	blue	Csa1G008520	2.771	0.00804	Transport	Plasma membrane proton ATPase 4 (HA4)	0.00E+00
T2-L2	blue	Csa6G514850	3.048	0.00009803	Miscellaneous	Cytochrome P450 family 77 subfamily B polypeptide 1 (CYP77B1)	1.00E-241
T2-L2	blue	Csa5G604150	3.14	0.01	Redox	Thioredoxin 2 (TRX2)	
T2-L2	blue	Csa5G223070	3.216	0.003151	Transcription factor	WRKY DNA-binding protein 54 (WRKY54)	3.00E-96
T2-L2	blue	Csa2G359890	3.289	3.70E-03	Not assigned	unknown	
T2-L2	blue	Csa1G596520	3.4	0.0002165	Protein degradation	Cystein proteinases superfamily protein	2.00E-168
T2-L2	blue	Csa7G395800	3.415	0.001823	Miscellaneous	Glutathione S-transferase phi 8 (GSTF8)	3.00E-82
T2-L2	blue	Csa3G119700	3.575	0.00005376	Transcription factor	WRKY DNA-binding protein 53 (WRKY53)	1.00E-102
T2-L2	blue	Csa6G381850	3.831	0.001156	Transport	Amino acid permease 3 (AAP3)	2.00E-234
T2-L2	blue	Csa1G033160	4.495	0.006049	Miscellaneous	Glutathione S-transferase tau 9 (GSTU9)	3.00E-68
T2-L2	blue	Csa6G486960	4.496	0.000002502	Transcription factor	WRKY DNA-binding protein 51 (WRKY51)	1.00E-36
T2-L2	blue	Csa2G058620	5.345	0.003945	Abiotic stress	DNAJ heat shock N-terminal domain-containing protein	2.00E-137

Supplementary Table 1 (Continued)

T2-L2	blue	Csa1G574970	1.827	1.18E-05	Major CHO metabolism	Hexokinase 2 (HXK2)	7.00E-51
T2-L2	blue	Csa4G618520	1.837	0.0001765	Biodegradation of xenobiotic	UDP glycosyltransferase (UGT)	1.00E-132
T2-L2	blue	Csa2G003610	1.841	0.0003098	Lipid metabolism	Abnormal inflorescence meristem (AIM1)	3.00E-294
T2-L2	blue	Csa3G730930	1.847	0.00004243	Transport	Ammonium transporter 2 (AMT2)	8E-220
T2-L2	blue	Csa6G493850	1.86	1.50E-05	Not assigned	unknown	
T2-L2	blue	Csa3G159420	1.871	1.61E-03	Not assigned	unknown	
T2-L2	blue	Csa1G528610	1.874	0.00435	Lipid metabolism	Thylakoid membrane anion transporter 1 (ANTR1)	7.00E-236
T2-L2	blue	Csa1G181360	1.875	0.001947	Transport	non-intrinsic ABC protein 9 (NAP9)	3.00E-156
T2-L2	blue	Csa3G154410	2.05	1.63E-10	Not assigned	unknown	
T2-L2	blue	Csa3G183950	2.092	0.0004285	Abiotic stress	Heat shock protein 83	0.00E+00
T2-L2	blue	Csa2G296090	2.143	2.44E-04	Not assigned	unknown	
T2-L2	blue	Csa7G291720	2.169	0.006566	Lipid metabolism	Choline kinase	4.00E-133
T2-L2	blue	Csa2G427310	2.238	0.007581	Transcription factor	MYB domain protein 73 (MYB73)	1.00E-88
T2-L2	blue	Csa3G881750	2.24	0.009561	Lipid metabolism	PR5-like receptor kinase (PR5K)	4.00E-154
T2-L2	blue	Csa5G589260	2.243	0	Amino acid metabolism	O-acetylserine(thiol) lyase (OAS-TL)	4.00E-162
T2-L2	blue	Csa3G733350	2.317	0.00004435	Cell organization	RHO guanyl-nucleotide exchange factor 11 (ROPGEF11)	9.00E-41
T2-L2	blue	Csa2G004690	2.522	0.000004593	Transcription factor	Heat shock transcription factor C1 (HSFC1)	5.00E-67
T2-L2	blue	Csa7G387690	2.631	0.000001654	Transport	Sulfate transporter 3;1 (SULTR 3;1)	3.00E-307
T2-L2	blue	Csa1G008520	2.771	0.00804	Transport	Plasma membrane proton ATPase 4 (HA4)	0.00E+00
T2-L2	blue	Csa6G514850	3.048	0.00009803	Miscellaneous	Cytochrome P450 family 77 subfamily B polypeptide 1 (CYP77B1)	1.00E-241
T2-L2	blue	Csa5G604150	3.14	0.01	Redox	Thioredoxin 2 (TRX2)	
T2-L2	blue	Csa5G223070	3.216	0.003151	Transcription factor	WRKY DNA-binding protein 54 (WRKY54)	3.00E-96
T2-L2	blue	Csa2G359890	3.289	3.70E-03	Not assigned	unknown	
T2-L2	blue	Csa1G596520	3.4	0.0002165	Protein degradation	Cystein proteinases superfamily protein	2.00E-168
T2-L2	blue	Csa7G395800	3.415	0.001823	Miscellaneous	Glutathione S-transferase phi 8 (GSTF8)	3.00E-82
T2-L2	blue	Csa3G119700	3.575	0.00005376	Transcription factor	WRKY DNA-binding protein 53 (WRKY53)	1.00E-102
T2-L2	blue	Csa6G381850	3.831	0.001156	Transport	Amino acid permease 3 (AAP3)	2.00E-234
T2-L2	blue	Csa1G033160	4.495	0.006049	Miscellaneous	Glutathione S-transferase tau 9 (GSTU9)	3.00E-68
T2-L2	blue	Csa6G486960	4.496	0.000002502	Transcription factor	WRKY DNA-binding protein 51 (WRKY51)	1.00E-36
T2-L2	blue	Csa2G058620	5.345	0.003945	Abiotic stress	DNAJ heat shock N-terminal domain-containing protein	2.00E-137

Supplementary Table 1 (Continued)

T2-L2	blue	Csa1G574970	1.827	1.18E-05	Major CHO metabolism	Hexokinase 2 (HXK2)	7.00E-51
T2-L2	blue	Csa4G618520	1.837	0.0001765	Biodegradation of xenobiotic	UDP glycosyltransferase (UGT)	1.00E-132
T2-L2	blue	Csa2G003610	1.841	0.0003098	Lipid metabolism	Abnormal inflorescence meristem (AIM1)	3.00E-294
T2-L2	blue	Csa3G730930	1.847	0.00004243	Transport	Ammonium transporter 2 (AMT2)	8E-220
T2-L2	blue	Csa6G493850	1.86	1.50E-05	Not assigned	unknown	
T2-L2	blue	Csa3G159420	1.871	1.61E-03	Not assigned	unknown	
T2-L2	blue	Csa1G528610	1.874	0.00435	Lipid metabolism	Thylakoid membrane anion transporter 1 (ANTR1)	7.00E-236
T2-L2	blue	Csa1G181360	1.875	0.001947	Transport	non-intrinsic ABC protein 9 (NAP9)	3.00E-156
T2-L2	blue	Csa3G154410	2.05	1.63E-10	Not assigned	unknown	
T2-L2	blue	Csa3G183950	2.092	0.0004285	Abiotic stress	Heat shock protein 83	0.00E+00
T2-L2	blue	Csa2G296090	2.143	2.44E-04	Not assigned	unknown	
T2-L2	blue	Csa7G291720	2.169	0.006566	Lipid metabolism	Choline kinase	4.00E-133
T2-L2	blue	Csa2G427310	2.238	0.007581	Transcription factor	MYB domain protein 73 (MYB73)	1.00E-88
T2-L2	blue	Csa3G881750	2.24	0.009561	Lipid metabolism	PR5-like receptor kinase (PR5K)	4.00E-154
T2-L2	blue	Csa5G589260	2.243	0	Amino acid metabolism	O-acetylserine(thiol) lyase (OAS-TL)	4.00E-162
T2-L2	blue	Csa3G733350	2.317	0.00004435	Cell organization	RHO guanyl-nucleotide exchange factor 11 (ROPGEF11)	9.00E-41
T2-L2	blue	Csa2G004690	2.522	0.000004593	Transcription factor	Heat shock transcription factor C1 (HSFC1)	5.00E-67
T2-L2	blue	Csa7G387690	2.631	0.000001654	Transport	Sulfate transporter 3;1 (SULTR 3;1)	3.00E-307
T2-L2	blue	Csa1G008520	2.771	0.00804	Transport	Plasma membrane proton ATPase 4 (HA4)	0.00E+00
T2-L2	blue	Csa6G514850	3.048	0.00009803	Miscellaneous	Cytochrome P450 family 77 subfamily B polypeptide 1 (CYP77B1)	1.00E-241
T2-L2	blue	Csa5G604150	3.14	0.01	Redox	Thioredoxin 2 (TRX2)	
T2-L2	blue	Csa5G223070	3.216	0.003151	Transcription factor	WRKY DNA-binding protein 54 (WRKY54)	3.00E-96
T2-L2	blue	Csa2G359890	3.289	3.70E-03	Not assigned	unknown	
T2-L2	blue	Csa1G596520	3.4	0.0002165	Protein degradation	Cystein proteinases superfamily protein	2.00E-168
T2-L2	blue	Csa7G395800	3.415	0.001823	Miscellaneous	Glutathione S-transferase phi 8 (GSTF8)	3.00E-82
T2-L2	blue	Csa3G119700	3.575	0.00005376	Transcription factor	WRKY DNA-binding protein 53 (WRKY53)	1.00E-102
T2-L2	blue	Csa6G381850	3.831	0.001156	Transport	Amino acid permease 3 (AAP3)	2.00E-234
T2-L2	blue	Csa1G033160	4.495	0.006049	Miscellaneous	Glutathione S-transferase tau 9 (GSTU9)	3.00E-68
T2-L2	blue	Csa6G486960	4.496	0.000002502	Transcription factor	WRKY DNA-binding protein 51 (WRKY51)	1.00E-36
T2-L2	blue	Csa2G058620	5.345	0.003945	Abiotic stress	DNAJ heat shock N-terminal domain-containing protein	2.00E-137

Supplementary Table 1 (Continued)

T2-L2	blue	Csa1G574970	1.827	1.18E-05	Major CHO metabolism	Hexokinase 2 (HXK2)	7.00E-51
T2-L2	blue	Csa4G618520	1.837	0.0001765	Biodegradation of xenobiotic	UDP glycosyltransferase (UGT)	1.00E-132
T2-L2	blue	Csa2G003610	1.841	0.0003098	Lipid metabolism	Abnormal inflorescence meristem (AIM1)	3.00E-294
T2-L2	blue	Csa3G730930	1.847	0.00004243	Transport	Ammonium transporter 2 (AMT2)	8E-220
T2-L2	blue	Csa6G493850	1.86	1.50E-05	Not assigned	unknown	
T2-L2	blue	Csa3G159420	1.871	1.61E-03	Not assigned	unknown	
T2-L2	blue	Csa1G528610	1.874	0.00435	Lipid metabolism	Thylakoid membrane anion transporter 1 (ANTR1)	7.00E-236
T2-L2	blue	Csa1G181360	1.875	0.001947	Transport	non-intrinsic ABC protein 9 (NAP9)	3.00E-156
T2-L2	blue	Csa3G154410	2.05	1.63E-10	Not assigned	unknown	
T2-L2	blue	Csa3G183950	2.092	0.0004285	Abiotic stress	Heat shock protein 83	0.00E+00
T2-L2	blue	Csa2G296090	2.143	2.44E-04	Not assigned	unknown	
T2-L2	blue	Csa7G291720	2.169	0.006566	Lipid metabolism	Choline kinase	4.00E-133
T2-L2	blue	Csa2G427310	2.238	0.007581	Transcription factor	MYB domain protein 73 (MYB73)	1.00E-88
T2-L2	blue	Csa3G881750	2.24	0.009561	Lipid metabolism	PR5-like receptor kinase (PR5K)	4.00E-154
T2-L2	blue	Csa5G589260	2.243	0	Amino acid metabolism	O-acetylserine(thiol) lyase (OAS-TL)	4.00E-162
T2-L2	blue	Csa3G733350	2.317	0.00004435	Cell organization	RHO guanyl-nucleotide exchange factor 11 (ROPGEF11)	9.00E-41
T2-L2	blue	Csa2G004690	2.522	0.000004593	Transcription factor	Heat shock transcription factor C1 (HSFC1)	5.00E-67
T2-L2	blue	Csa7G387690	2.631	0.000001654	Transport	Sulfate transporter 3;1 (SULTR 3;1)	3.00E-307
T2-L2	blue	Csa1G008520	2.771	0.00804	Transport	Plasma membrane proton ATPase 4 (HA4)	0.00E+00
T2-L2	blue	Csa6G514850	3.048	0.00009803	Miscellaneous	Cytochrome P450 family 77 subfamily B polypeptide 1 (CYP77B1)	1.00E-241
T2-L2	blue	Csa5G604150	3.14	0.01	Redox	Thioredoxin 2 (TRX2)	
T2-L2	blue	Csa5G223070	3.216	0.003151	Transcription factor	WRKY DNA-binding protein 54 (WRKY54)	3.00E-96
T2-L2	blue	Csa2G359890	3.289	3.70E-03	Not assigned	unknown	
T2-L2	blue	Csa1G596520	3.4	0.0002165	Protein degradation	Cystein proteinases superfamily protein	2.00E-168
T2-L2	blue	Csa7G395800	3.415	0.001823	Miscellaneous	Glutathione S-transferase phi 8 (GSTF8)	3.00E-82
T2-L2	blue	Csa3G119700	3.575	0.00005376	Transcription factor	WRKY DNA-binding protein 53 (WRKY53)	1.00E-102
T2-L2	blue	Csa6G381850	3.831	0.001156	Transport	Amino acid permease 3 (AAP3)	2.00E-234
T2-L2	blue	Csa1G033160	4.495	0.006049	Miscellaneous	Glutathione S-transferase tau 9 (GSTU9)	3.00E-68
T2-L2	blue	Csa6G486960	4.496	0.000002502	Transcription factor	WRKY DNA-binding protein 51 (WRKY51)	1.00E-36
T2-L2	blue	Csa2G058620	5.345	0.003945	Abiotic stress	DNAJ heat shock N-terminal domain-containing protein	2.00E-137

Supplementary Table 1 (Continued)

T2-L2	blue	Csa1G574970	1.827	1.18E-05	Major CHO metabolism	Hexokinase 2 (HXK2)	7.00E-51
T2-L2	blue	Csa4G618520	1.837	0.0001765	Biodegradation of xenobiotic	UDP glycosyltransferase (UGT)	1.00E-132
T2-L2	blue	Csa2G003610	1.841	0.0003098	Lipid metabolism	Abnormal inflorescence meristem (AIM1)	3.00E-294
T2-L2	blue	Csa3G730930	1.847	0.00004243	Transport	Ammonium transporter 2 (AMT2)	8E-220
T2-L2	blue	Csa6G493850	1.86	1.50E-05	Not assigned	unknown	
T2-L2	blue	Csa3G159420	1.871	1.61E-03	Not assigned	unknown	
T2-L2	blue	Csa1G528610	1.874	0.00435	Lipid metabolism	Thylakoid membrane anion transporter 1 (ANTR1)	7.00E-236
T2-L2	blue	Csa1G181360	1.875	0.001947	Transport	non-intrinsic ABC protein 9 (NAP9)	3.00E-156
T2-L2	blue	Csa3G154410	2.05	1.63E-10	Not assigned	unknown	
T2-L2	blue	Csa3G183950	2.092	0.0004285	Abiotic stress	Heat shock protein 83	0.00E+00
T2-L2	blue	Csa2G296090	2.143	2.44E-04	Not assigned	unknown	
T2-L2	blue	Csa7G291720	2.169	0.006566	Lipid metabolism	Choline kinase	4.00E-133
T2-L2	blue	Csa2G427310	2.238	0.007581	Transcription factor	MYB domain protein 73 (MYB73)	1.00E-88
T2-L2	blue	Csa3G881750	2.24	0.009561	Lipid metabolism	PR5-like receptor kinase (PR5K)	4.00E-154
T2-L2	blue	Csa5G589260	2.243	0	Amino acid metabolism	O-acetylserine(thiol) lyase (OAS-TL)	4.00E-162
T2-L2	blue	Csa3G733350	2.317	0.00004435	Cell organization	RHO guanyl-nucleotide exchange factor 11 (ROPGEF11)	9.00E-41
T2-L2	blue	Csa2G004690	2.522	0.000004593	Transcription factor	Heat shock transcription factor C1 (HSFC1)	5.00E-67
T2-L2	blue	Csa7G387690	2.631	0.000001654	Transport	Sulfate transporter 3;1 (SULTR 3;1)	3.00E-307
T2-L2	blue	Csa1G008520	2.771	0.00804	Transport	Plasma membrane proton ATPase 4 (HA4)	0.00E+00
T2-L2	blue	Csa6G514850	3.048	0.00009803	Miscellaneous	Cytochrome P450 family 77 subfamily B polypeptide 1 (CYP77B1)	1.00E-241
T2-L2	blue	Csa5G604150	3.14	0.01	Redox	Thioredoxin 2 (TRX2)	
T2-L2	blue	Csa5G223070	3.216	0.003151	Transcription factor	WRKY DNA-binding protein 54 (WRKY54)	3.00E-96
T2-L2	blue	Csa2G359890	3.289	3.70E-03	Not assigned	unknown	
T2-L2	blue	Csa1G596520	3.4	0.0002165	Protein degradation	Cystein proteinases superfamily protein	2.00E-168
T2-L2	blue	Csa7G395800	3.415	0.001823	Miscellaneous	Glutathione S-transferase phi 8 (GSTF8)	3.00E-82
T2-L2	blue	Csa3G119700	3.575	0.00005376	Transcription factor	WRKY DNA-binding protein 53 (WRKY53)	1.00E-102
T2-L2	blue	Csa6G381850	3.831	0.001156	Transport	Amino acid permease 3 (AAP3)	2.00E-234
T2-L2	blue	Csa1G033160	4.495	0.006049	Miscellaneous	Glutathione S-transferase tau 9 (GSTU9)	3.00E-68
T2-L2	blue	Csa6G486960	4.496	0.000002502	Transcription factor	WRKY DNA-binding protein 51 (WRKY51)	1.00E-36
T2-L2	blue	Csa2G058620	5.345	0.003945	Abiotic stress	DNAJ heat shock N-terminal domain-containing protein	2.00E-137

Supplementary Table 1 (Continued)

T2-L2	blue	Csa1G574970	1.827	1.18E-05	Major CHO metabolism	Hexokinase 2 (HXK2)	7.00E-51
T2-L2	blue	Csa4G618520	1.837	0.0001765	Biodegradation of xenobiotic	UDP glycosyltransferase (UGT)	1.00E-132
T2-L2	blue	Csa2G003610	1.841	0.0003098	Lipid metabolism	Abnormal inflorescence meristem (AIM1)	3.00E-294
T2-L2	blue	Csa3G730930	1.847	0.00004243	Transport	Ammonium transporter 2 (AMT2)	8E-220
T2-L2	blue	Csa6G493850	1.86	1.50E-05	Not assigned	unknown	
T2-L2	blue	Csa3G159420	1.871	1.61E-03	Not assigned	unknown	
T2-L2	blue	Csa1G528610	1.874	0.00435	Lipid metabolism	Thylakoid membrane anion transporter 1 (ANTR1)	7.00E-236
T2-L2	blue	Csa1G181360	1.875	0.001947	Transport	non-intrinsic ABC protein 9 (NAP9)	3.00E-156
T2-L2	blue	Csa3G154410	2.05	1.63E-10	Not assigned	unknown	
T2-L2	blue	Csa3G183950	2.092	0.0004285	Abiotic stress	Heat shock protein 83	0.00E+00
T2-L2	blue	Csa2G296090	2.143	2.44E-04	Not assigned	unknown	
T2-L2	blue	Csa7G291720	2.169	0.006566	Lipid metabolism	Choline kinase	4.00E-133
T2-L2	blue	Csa2G427310	2.238	0.007581	Transcription factor	MYB domain protein 73 (MYB73)	1.00E-88
T2-L2	blue	Csa3G881750	2.24	0.009561	Lipid metabolism	PR5-like receptor kinase (PR5K)	4.00E-154
T2-L2	blue	Csa5G589260	2.243	0	Amino acid metabolism	O-acetylserine(thiol) lyase (OAS-TL)	4.00E-162
T2-L2	blue	Csa3G733350	2.317	0.00004435	Cell organization	RHO guanyl-nucleotide exchange factor 11 (ROPGEF11)	9.00E-41
T2-L2	blue	Csa2G004690	2.522	0.000004593	Transcription factor	Heat shock transcription factor C1 (HSFC1)	5.00E-67
T2-L2	blue	Csa7G387690	2.631	0.000001654	Transport	Sulfate transporter 3;1 (SULTR 3;1)	3.00E-307
T2-L2	blue	Csa1G008520	2.771	0.00804	Transport	Plasma membrane proton ATPase 4 (HA4)	0.00E+00
T2-L2	blue	Csa6G514850	3.048	0.00009803	Miscellaneous	Cytochrome P450 family 77 subfamily B polypeptide 1 (CYP77B1)	1.00E-241
T2-L2	blue	Csa5G604150	3.14	0.01	Redox	Thioredoxin 2 (TRX2)	
T2-L2	blue	Csa5G223070	3.216	0.003151	Transcription factor	WRKY DNA-binding protein 54 (WRKY54)	3.00E-96
T2-L2	blue	Csa2G359890	3.289	3.70E-03	Not assigned	unknown	
T2-L2	blue	Csa1G596520	3.4	0.0002165	Protein degradation	Cystein proteinases superfamily protein	2.00E-168
T2-L2	blue	Csa7G395800	3.415	0.001823	Miscellaneous	Glutathione S-transferase phi 8 (GSTF8)	3.00E-82
T2-L2	blue	Csa3G119700	3.575	0.00005376	Transcription factor	WRKY DNA-binding protein 53 (WRKY53)	1.00E-102
T2-L2	blue	Csa6G381850	3.831	0.001156	Transport	Amino acid permease 3 (AAP3)	2.00E-234
T2-L2	blue	Csa1G033160	4.495	0.006049	Miscellaneous	Glutathione S-transferase tau 9 (GSTU9)	3.00E-68
T2-L2	blue	Csa6G486960	4.496	0.000002502	Transcription factor	WRKY DNA-binding protein 51 (WRKY51)	1.00E-36
T2-L2	blue	Csa2G058620	5.345	0.003945	Abiotic stress	DNAJ heat shock N-terminal domain-containing protein	2.00E-137

Supplementary Table 1 (Continued)

T2-L2	blue	Csa1G574970	1.827	1.18E-05	Major CHO metabolism	Hexokinase 2 (HXK2)	7.00E-51
T2-L2	blue	Csa4G618520	1.837	0.0001765	Biodegradation of xenobiotic	UDP glycosyltransferase (UGT)	1.00E-132
T2-L2	blue	Csa2G003610	1.841	0.0003098	Lipid metabolism	Abnormal inflorescence meristem (AIM1)	3.00E-294
T2-L2	blue	Csa3G730930	1.847	0.00004243	Transport	Ammonium transporter 2 (AMT2)	8E-220
T2-L2	blue	Csa6G493850	1.86	1.50E-05	Not assigned	unknown	
T2-L2	blue	Csa3G159420	1.871	1.61E-03	Not assigned	unknown	
T2-L2	blue	Csa1G528610	1.874	0.00435	Lipid metabolism	Thylakoid membrane anion transporter 1 (ANTR1)	7.00E-236
T2-L2	blue	Csa1G181360	1.875	0.001947	Transport	non-intrinsic ABC protein 9 (NAP9)	3.00E-156
T2-L2	blue	Csa3G154410	2.05	1.63E-10	Not assigned	unknown	
T2-L2	blue	Csa3G183950	2.092	0.0004285	Abiotic stress	Heat shock protein 83	0.00E+00
T2-L2	blue	Csa2G296090	2.143	2.44E-04	Not assigned	unknown	
T2-L2	blue	Csa7G291720	2.169	0.006566	Lipid metabolism	Choline kinase	4.00E-133
T2-L2	blue	Csa2G427310	2.238	0.007581	Transcription factor	MYB domain protein 73 (MYB73)	1.00E-88
T2-L2	blue	Csa3G881750	2.24	0.009561	Lipid metabolism	PR5-like receptor kinase (PR5K)	4.00E-154
T2-L2	blue	Csa5G589260	2.243	0	Amino acid metabolism	O-acetylserine(thiol) lyase (OAS-TL)	4.00E-162
T2-L2	blue	Csa3G733350	2.317	0.00004435	Cell organization	RHO guanyl-nucleotide exchange factor 11 (ROPGEF11)	9.00E-41
T2-L2	blue	Csa2G004690	2.522	0.000004593	Transcription factor	Heat shock transcription factor C1 (HSFC1)	5.00E-67
T2-L2	blue	Csa7G387690	2.631	0.000001654	Transport	Sulfate transporter 3;1 (SULTR 3;1)	3.00E-307
T2-L2	blue	Csa1G008520	2.771	0.00804	Transport	Plasma membrane proton ATPase 4 (HA4)	0.00E+00
T2-L2	blue	Csa6G514850	3.048	0.00009803	Miscellaneous	Cytochrome P450 family 77 subfamily B polypeptide 1 (CYP77B1)	1.00E-241
T2-L2	blue	Csa5G604150	3.14	0.01	Redox	Thioredoxin 2 (TRX2)	
T2-L2	blue	Csa5G223070	3.216	0.003151	Transcription factor	WRKY DNA-binding protein 54 (WRKY54)	3.00E-96
T2-L2	blue	Csa2G359890	3.289	3.70E-03	Not assigned	unknown	
T2-L2	blue	Csa1G596520	3.4	0.0002165	Protein degradation	Cystein proteinases superfamily protein	2.00E-168
T2-L2	blue	Csa7G395800	3.415	0.001823	Miscellaneous	Glutathione S-transferase phi 8 (GSTF8)	3.00E-82
T2-L2	blue	Csa3G119700	3.575	0.00005376	Transcription factor	WRKY DNA-binding protein 53 (WRKY53)	1.00E-102
T2-L2	blue	Csa6G381850	3.831	0.001156	Transport	Amino acid permease 3 (AAP3)	2.00E-234
T2-L2	blue	Csa1G033160	4.495	0.006049	Miscellaneous	Glutathione S-transferase tau 9 (GSTU9)	3.00E-68
T2-L2	blue	Csa6G486960	4.496	0.000002502	Transcription factor	WRKY DNA-binding protein 51 (WRKY51)	1.00E-36
T2-L2	blue	Csa2G058620	5.345	0.003945	Abiotic stress	DNAJ heat shock N-terminal domain-containing protein	2.00E-137

Supplementary Table 1 (Continued)

T2-L2	blue	Csa1G574970	1.827	1.18E-05	Major CHO metabolism	Hexokinase 2 (HXK2)	7.00E-51
T2-L2	blue	Csa4G618520	1.837	0.0001765	Biodegradation of xenobiotic	UDP glycosyltransferase (UGT)	1.00E-132
T2-L2	blue	Csa2G003610	1.841	0.0003098	Lipid metabolism	Abnormal inflorescence meristem (AIM1)	3.00E-294
T2-L2	blue	Csa3G730930	1.847	0.00004243	Transport	Ammonium transporter 2 (AMT2)	8E-220
T2-L2	blue	Csa6G493850	1.86	1.50E-05	Not assigned	unknown	
T2-L2	blue	Csa3G159420	1.871	1.61E-03	Not assigned	unknown	
T2-L2	blue	Csa1G528610	1.874	0.00435	Lipid metabolism	Thylakoid membrane anion transporter 1 (ANTR1)	7.00E-236
T2-L2	blue	Csa1G181360	1.875	0.001947	Transport	non-intrinsic ABC protein 9 (NAP9)	3.00E-156
T2-L2	blue	Csa3G154410	2.05	1.63E-10	Not assigned	unknown	
T2-L2	blue	Csa3G183950	2.092	0.0004285	Abiotic stress	Heat shock protein 83	0.00E+00
T2-L2	blue	Csa2G296090	2.143	2.44E-04	Not assigned	unknown	
T2-L2	blue	Csa7G291720	2.169	0.006566	Lipid metabolism	Choline kinase	4.00E-133
T2-L2	blue	Csa2G427310	2.238	0.007581	Transcription factor	MYB domain protein 73 (MYB73)	1.00E-88
T2-L2	blue	Csa3G881750	2.24	0.009561	Lipid metabolism	PR5-like receptor kinase (PR5K)	4.00E-154
T2-L2	blue	Csa5G589260	2.243	0	Amino acid metabolism	O-acetylserine(thiol) lyase (OAS-TL)	4.00E-162
T2-L2	blue	Csa3G733350	2.317	0.00004435	Cell organization	RHO guanyl-nucleotide exchange factor 11 (ROPGEF11)	9.00E-41
T2-L2	blue	Csa2G004690	2.522	0.000004593	Transcription factor	Heat shock transcription factor C1 (HSFC1)	5.00E-67
T2-L2	blue	Csa7G387690	2.631	0.000001654	Transport	Sulfate transporter 3;1 (SULTR 3;1)	3.00E-307
T2-L2	blue	Csa1G008520	2.771	0.00804	Transport	Plasma membrane proton ATPase 4 (HA4)	0.00E+00
T2-L2	blue	Csa6G514850	3.048	0.00009803	Miscellaneous	Cytochrome P450 family 77 subfamily B polypeptide 1 (CYP77B1)	1.00E-241
T2-L2	blue	Csa5G604150	3.14	0.01	Redox	Thioredoxin 2 (TRX2)	
T2-L2	blue	Csa5G223070	3.216	0.003151	Transcription factor	WRKY DNA-binding protein 54 (WRKY54)	3.00E-96
T2-L2	blue	Csa2G359890	3.289	3.70E-03	Not assigned	unknown	
T2-L2	blue	Csa1G596520	3.4	0.0002165	Protein degradation	Cystein proteinases superfamily protein	2.00E-168
T2-L2	blue	Csa7G395800	3.415	0.001823	Miscellaneous	Glutathione S-transferase phi 8 (GSTF8)	3.00E-82
T2-L2	blue	Csa3G119700	3.575	0.00005376	Transcription factor	WRKY DNA-binding protein 53 (WRKY53)	1.00E-102
T2-L2	blue	Csa6G381850	3.831	0.001156	Transport	Amino acid permease 3 (AAP3)	2.00E-234
T2-L2	blue	Csa1G033160	4.495	0.006049	Miscellaneous	Glutathione S-transferase tau 9 (GSTU9)	3.00E-68
T2-L2	blue	Csa6G486960	4.496	0.000002502	Transcription factor	WRKY DNA-binding protein 51 (WRKY51)	1.00E-36
T2-L2	blue	Csa2G058620	5.345	0.003945	Abiotic stress	DNAJ heat shock N-terminal domain-containing protein	2.00E-137

Supplementary Table 1 (Continued)

T2-L2	blue	Csa3G064240	5.658	3.97E-13	Not assigned	unknown	
T2-L2	blue	Csa3G727990	6.451	4.501E-07	Transcription factor	WRKY DNA-binding protein 70 (WRKY70)	4.00E-166
T2-L2	blue	Csa6G093640	7.096	0.003228	Transcription factor	PCF (TCP)-domain family protein 20 (TCP20)	6.00E-72
T2-L2	blue	Csa3G825080	7.272	0.003456	Miscellaneous	Cytochrome P450 family 72 subfamily A polypeptide 8 (CYP72A8)	5.00E-38
T2-L2	blue	Csa1G002860	8.184	0.002006	Signaling	Suppressor of BIR1 (SOBIR1)	9.00E-255
T2-L2	blue	Csa2G297180	12.788	0.0003204	Transcription factor	WRKY DNA-binding protein 51 (WRKY51)	3.00E-51
T2-L2	blue	Csa7G037610	14.168	7.21E-03	Not assigned	unknown	
T2-L2	blue	Csa7G414490	17.197	0.001714	Miscellaneous	NAD(P)-binding Rossmann-fold superfamily protein	1.00E-84
T2-L2	blue	Csa1G007850	1.783	0.026	Transcription factor	MYB-like DNA-binding domain protein	7.00E-61
T2-L2	blue	Csa1G022520	3.614	0.025	Not assigned	unknown	2.00E-30
T2-L2	blue	Csa1G043040	1.212	0.027	Transcription factor	Homeobox protein 22 (HB22)	3.00E-22
T2-L2	blue	Csa1G045650	-3.2	1.04E-07	Protein degradation	AAA-ATPase 1 (AATP1)	1.00E-149
T2-L2	blue	Csa1G132110	1.644	0.015	Not assigned	unknown	1.00E-43
T2-L2	blue	Csa1G164660	-1.478	0.04	Miscellaneous	Glycosyl hydrolase superfamily protein	6.00E-149
T2-L2	blue	Csa1G165230	1.466	0.016	Transcription factor	NAC domain containing protein 90 (NAC090)	8.00E-62
T2-L2	blue	Csa1G212830	1.554	0.025	Secondary metabolism	2-oxoglutarase (2OG) and Fe(II)-dependent oxygenase superfamily protein	6.00E-92
T2-L2	blue	Csa1G523610	1.418	0.031	Transcription factor	GRAS family transcription factor	1.00E-144
T2-L2	blue	Csa1G560830	-4.342	1.61E-05	RNA processing	RNA helicase 2	2.00E-282
T2-L2	blue	Csa1G575160	1.173	0.027	Transcription factor	SNF7 family protein	7.00E-104
T2-L2	blue	Csa1G611290	1.417	0.014	Miscellaneous	Beta glucosidase 15 (BGLU15)	9.00E-159
T2-L2	blue	Csa2G163170	1.75	0.014	Transport	Ammonium transporter 1;1 (AMT1;1)	2.00E-241
T2-L2	blue	Csa2G287040	1.909	0.032	Cell organization	Ankyrin repeat family protein	5.00E-14
T2-L2	blue	Csa2G345990	1.632	0.019	Not assigned	Thioredoxin superfamily protein	5.00E-67
T2-L2	blue	Csa2G351820	1.889	0.031	Protein targeting	Vacuolar-processing enzyme precursor (VPE)	2.00E-219
T2-L2	blue	Csa2G358860	1.58	0.049	Not assigned	unknown	
T2-L2	blue	Csa2G359940	1.788	0	Not assigned	unknown	
T2-L2	blue	Csa2G360750	1.962	6.24E-03	Not assigned	Transducing/WD40 repeat-like superfamily protein	1.00E-141
T2-L2	blue	Csa2G423560	1.685	0.012	Transcription factor	B-box type zinc finger family protein	5.00E-21
T2-L2	blue	Csa3G035840	-3.171	3.41E-03	Miscellaneous	receptor serine/threonine kinase	4.00E-19
T2-L2	blue	Csa3G061000	5.308	0.016	Protein postranslational modification	Calcium-binding EF-hand family protein	1.00E-85

Supplementary Table 1 (Continued)

T2-L2	blue	Csa1G574970	1.827	1.18E-05	Major CHO metabolism	Hexokinase 2 (HXK2)	7.00E-51
T2-L2	blue	Csa4G618520	1.837	0.0001765	Biodegradation of xenobiotic	UDP glycosyltransferase (UGT)	1.00E-132
T2-L2	blue	Csa2G003610	1.841	0.0003098	Lipid metabolism	Abnormal inflorescence meristem (AIM1)	3.00E-294
T2-L2	blue	Csa3G730930	1.847	0.00004243	Transport	Ammonium transporter 2 (AMT2)	8E-220
T2-L2	blue	Csa6G493850	1.86	1.50E-05	Not assigned	unknown	
T2-L2	blue	Csa3G159420	1.871	1.61E-03	Not assigned	unknown	
T2-L2	blue	Csa1G528610	1.874	0.00435	Lipid metabolism	Thylakoid membrane anion transporter 1 (ANTR1)	7.00E-236
T2-L2	blue	Csa1G181360	1.875	0.001947	Transport	non-intrinsic ABC protein 9 (NAP9)	3.00E-156
T2-L2	blue	Csa3G154410	2.05	1.63E-10	Not assigned	unknown	
T2-L2	blue	Csa3G183950	2.092	0.0004285	Abiotic stress	Heat shock protein 83	0.00E+00
T2-L2	blue	Csa2G296090	2.143	2.44E-04	Not assigned	unknown	
T2-L2	blue	Csa7G291720	2.169	0.006566	Lipid metabolism	Choline kinase	4.00E-133
T2-L2	blue	Csa2G427310	2.238	0.007581	Transcription factor	MYB domain protein 73 (MYB73)	1.00E-88
T2-L2	blue	Csa3G881750	2.24	0.009561	Lipid metabolism	PR5-like receptor kinase (PR5K)	4.00E-154
T2-L2	blue	Csa5G589260	2.243	0	Amino acid metabolism	O-acetylserine(thiol) lyase (OAS-TL)	4.00E-162
T2-L2	blue	Csa3G733350	2.317	0.00004435	Cell organization	RHO guanyl-nucleotide exchange factor 11 (ROPGEF11)	9.00E-41
T2-L2	blue	Csa2G004690	2.522	0.000004593	Transcription factor	Heat shock transcription factor C1 (HSFC1)	5.00E-67
T2-L2	blue	Csa7G387690	2.631	0.000001654	Transport	Sulfate transporter 3;1 (SULTR 3;1)	3.00E-307
T2-L2	blue	Csa1G008520	2.771	0.00804	Transport	Plasma membrane proton ATPase 4 (HA4)	0.00E+00
T2-L2	blue	Csa6G514850	3.048	0.00009803	Miscellaneous	Cytochrome P450 family 77 subfamily B polypeptide 1 (CYP77B1)	1.00E-241
T2-L2	blue	Csa5G604150	3.14	0.01	Redox	Thioredoxin 2 (TRX2)	
T2-L2	blue	Csa5G223070	3.216	0.003151	Transcription factor	WRKY DNA-binding protein 54 (WRKY54)	3.00E-96
T2-L2	blue	Csa2G359890	3.289	3.70E-03	Not assigned	unknown	
T2-L2	blue	Csa1G596520	3.4	0.0002165	Protein degradation	Cystein proteinases superfamily protein	2.00E-168
T2-L2	blue	Csa7G395800	3.415	0.001823	Miscellaneous	Glutathione S-transferase phi 8 (GSTF8)	3.00E-82
T2-L2	blue	Csa3G119700	3.575	0.00005376	Transcription factor	WRKY DNA-binding protein 53 (WRKY53)	1.00E-102
T2-L2	blue	Csa6G381850	3.831	0.001156	Transport	Amino acid permease 3 (AAP3)	2.00E-234
T2-L2	blue	Csa1G033160	4.495	0.006049	Miscellaneous	Glutathione S-transferase tau 9 (GSTU9)	3.00E-68
T2-L2	blue	Csa6G486960	4.496	0.000002502	Transcription factor	WRKY DNA-binding protein 51 (WRKY51)	1.00E-36
T2-L2	blue	Csa2G058620	5.345	0.003945	Abiotic stress	DNAJ heat shock N-terminal domain-containing protein	2.00E-137

Supplementary Table 1 (Continued)

T2-L2	blue	Csa3G099650	1.434	3.40E-03	Abiotic stress	Wound-responsive family protein	1.00E-17
T2-L2	blue	Csa3G145780	10.118	0.012	Signaling	Early light-inducible protein (ELIP1)	7.00E-55
T2-L2	blue	Csa3G146300	1.261	3.15E-05	Metal handling	Acireductone dioxygenase 2 (ARD2)	1.00E-92
T2-L2	blue	Csa3G171820	1.309	0.023	Hormone metabolism	SAUR-like auxin-responsive protein family	1.00E-45
T2-L2	blue	Csa3G178520	1.315	3.66E-03	Protein degradation	Subtilase 1.3 (SBT1.3)	0
T2-L2	blue	Csa3G181940	1.399	0.012	Abiotic stress	Heat stress transcription factor (Hsf) family	3.00E-55
T2-L2	blue	Csa3G182150	-1.65	9.72E-06	Signaling	PAR1 protein	3.00E-55
T2-L2	blue	Csa3G239250	-2.005	1.63E-04	Not assigned	unknown	
T2-L2	blue	Csa3G316280	2.061	7.95E-07	Transcription factor	Calcium-dependent lipid-binding (CaLB domain) family protein	4.00E-64
T2-L2	blue	Csa3G319290	1.31	0.04	Biotic stress	Polyketide cyclase/dehydrase and lipid transport superfamily protein	3.00E-20
T2-L2	blue	Csa3G333840	3.405	6.16E-05	Biotic stress	MLP-like protein 34 (MLP34)	3.00E-13
T2-L2	blue	Csa3G588450	1.584	0.047	Protein synthesis	Glucose hypersensitive 1 (GHS1)	3.00E-45
T2-L2	blue	Csa3G646010	1.758	0.013	Hormone metabolism	Dormancy/auxin associated family protein	3.00E-15
T2-L2	blue	Csa3G646660	2.135	0.048	Not assigned	unknown	
T2-L2	blue	Csa3G732460	1.343	0.046	Lipid metabolism	Sulfoquinovosyldiacylglycerol 2 (SQD2)	7.00E-209
T2-L2	blue	Csa3G733230	-2.994	0.046	Abiotic stress	Heat shock protein 20 (HSP20)	2.00E-13
T2-L2	blue	Csa3G776960	3.355	0.014	Not assigned	Thioredoxin family protein	4.00E-81
T2-L2	blue	Csa3G820480	1.617	0.026	Protein targeting	Sigma factor binding protein 1 (SIB1)	1.00E-10
T2-L2	blue	Csa3G848170	1.744	0.024	Protein degradation	Amino peptidase M1 (APM1)	3.00E-284
T2-L2	blue	Csa3G848300	-2.377	0.021	Miscellaneous	nitrile lyases, berberine bridge enzymes, reticuline oxidases, troponine reductases	9.00E-238
T2-L2	blue	Csa3G859670	1.268	0.024	Not assigned	unknown	4.00E-10
T2-L2	blue	Csa3G860260	4.271	0.028	Abiotic stress	Wound-responsive family protein	1.00E-127
T2-L2	blue	Csa3G872070	-1.57	0.046	Cell wall	Glycosyl hydrolase 9B5 (GH9B5)	2.00E-228
T2-L2	blue	Csa3G873260	2.16	0.026	Not assigned	unknown	1.00E-06
T2-L2	blue	Csa3G878740	3.003	0.014	Development	DNAJ/HSP40 cystein-rich domain superfamily protein	6.00E-32
T2-L2	blue	Csa4G011770	3.01	0.015	Transcription factor	NAC domain containing protein 83 (NAC083)	4.00E-62
T2-L2	blue	Csa4G025120	-1.865	0.027	Not assigned	unknown	4.00E-44
T2-L2	blue	Csa4G052080	3.146	0.035	Not assigned	unknown	
T2-L2	blue	Csa4G056640	1.53	0.011	Hormone metabolism	Nine-cis-epoxycarotenoid dioxygenase 4 (NCED4)	3.00E-226
T2-L2	blue	Csa4G063480	1.772	0.028	Biotic stress	NPR1-like protein 3	2.00E-37

Supplementary Table 1 (Continued)

T2-L2	blue	Csa4G091870	3.269	1.43E-04	Secondary metabolism	Downy mildew resistant 6 (DMR6)	5.00E-140
T2-L2	blue	Csa4G269770	2.17	0.047	Protein postranslational modification	Protein kinase 2B (APK2B)	2.00E-125
T2-L2	blue	Csa4G361820	3.071	0.03	Development	NAC domain containing protein 32 (NAC32)	2.00E-88
T2-L2	blue	Csa4G630010	-1.901	0.039	Hormone metabolism	Ethylene response factor 8 (ERF8)	4.00E-32
T2-L2	blue	Csa4G646340	1.828	0.013	Abiotic stress	Heat shock protein 17.6A (HSP17.6II)	1.00E-49
T2-L2	blue	Csa4G651750	-3.219	0.02	Transcription factor	Aspartyl protease family protein	4.00E-136
T2-L2	blue	Csa5G011650	1.376	0.021	Miscellaneous	NAD(P)-binding Rossmann-fold superfamily protein	3.00E-65
T2-L2	blue	Csa5G097460	-1.366	1.15E-06	Biotic stress	P-loop containing nucleotide triphosphate hydrolases superfamily protein	1.00E-65
T2-L2	blue	Csa5G140450	7.962	0.02	DNA repair	Heavy metal transport/detoxification superfamily protein	3.00E-24
T2-L2	blue	Csa5G150420	2.011	0.048	Transcription factor	Apetala2/Ethylene-responsive element binding protein family	1.00E-48
T2-L2	blue	Csa5G152250	1.603	2.95E-05	DNA repair	Heavy metal transport/detoxification superfamily protein	2.00E-27
T2-L2	blue	Csa5G152260	-1.607	0.016	DNA repair	Heavy metal transport/detoxification superfamily protein	3.00E-27
T2-L2	blue	Csa5G153020	1.177	0.016	Transport	Plasma membrane intrinsic protein 1C (PIP1C)	4.00E-149
T2-L2	blue	Csa5G158570	1.562	3.77E-09	Miscellaneous	Copper amine oxidase family protein	2.00E-113
T2-L2	blue	Csa5G167120	1.63	0.019	Hormone metabolism	Ethylene response factor 104 (ERF104)	4.00E-45
T2-L2	blue	Csa5G168910	-1.606	0.021	Not assigned	unknown	
T2-L2	blue	Csa5G168920	2.675	0.026	Signaling	Leucine-rich repeat receptor -like protein kinase family protein	2.00E-76
T2-L2	blue	Csa5G179220	1.348	0.015	Lipid metabolism	Alpha/beta-hydrolases superfamily protein	1.00E-131
T2-L2	blue	Csa5G207940	2.293	3.45E-10	Transcription factor	Salt-inducible zinc finger 1 (SZF1)	2.00E-44
T2-L2	blue	Csa5G547610	-1.559	0.04	Nucleotide metabolism	Nudix hydrolase homolog 17 (NUDT17)	2.00E-67
T2-L2	blue	Csa5G610370	4.368	0.034	Lipid metabolism	PR5-like receptor kinase (PR5K)	1.00E-98
T2-L2	blue	Csa5G611750	1.952	0.016	Metal handling	Heavy metal transport/detoxification superfamily protein	3.00E-49
T2-L2	blue	Csa5G628650	7.218	0.02	Hormone metabolism	Jasmonate-zim-domain protein 3 (JAZ3)	4.00E-18
T2-L2	blue	Csa5G630800	-1.502	0.035	Miscellaneous	Oxidoreductase, zinc-binding dehydrogenase family protein	2.00E-111
T2-L2	blue	Csa6G084580	3.26	1.04E-10	Biotic stress	Disease resistance-responsive (dirigent-like protein) family protein	7.00E-47

Supplementary Table 1 (Continued)

T2-L2	blue	Csa6G106800	2.214	3.81E-05	Signaling	Calcium-binding EF-hand family protein	3.00E-41
T2-L2	blue	Csa6G127320	1.484	0.019	Development	NAC domain containing protein 36 (NAC036)	3.00E-73
T2-L2	blue	Csa6G133770	2.243	1.52E-07	Hormone metabolism	Cytokinin response factor 4 (CRF4)	2.00E-34
T2-L2	blue	Csa6G404210	10.38	0.029	Not assigned	SPFH/Band 7/ PHB domain-containing membrane-associated protein family	5.00E-143
T2-L2	blue	Csa6G445150	-1.623	4.96E-04	Not assigned	Aquaporin 2	2.00E-118
T2-L2	blue	Csa6G486670	1.638	0.024	Transcription factor	Tonoplast intrinsic protein 2 (TIP2)	2.00E-101
T2-L2	blue	Csa6G490900	3.584	0.021	Transcription factor	Remorin family protein	8.00E-35
T2-L2	blue	Csa6G495000	-3.252	0.027	Miscellaneous	Root hair specific 19 (RHS19)	5.00E-138
T2-L2	blue	Csa6G505280	1.841	9.08E-06	DNA synthesis	Histone H1-3 (HIS1-3)	4.00E-34
T2-L2	blue	Csa6G513580	1.269	2.01E-10	Protein targeting	Peroxisomal membrane 22kDa family protein	7.00E-64
T2-L2	blue	Csa6G514860	1.318	0.033	Transport	Glucose-6-phosphate/phosphate translocator 2 (GPT2)	4.00E-162
T2-L2	blue	Csa6G516580	1.417	0.044	Signaling	Cysteine-rich receptor-like protein kinase 29 (CRK29)	1.00E-55
T2-L2	blue	Csa6G538630	1.859	0.015	Co-factor and vitamin metabolism	Molybdenum cofactor sulfurase family protein	9.00E-120
T2-L2	blue	Csa7G037660	1.854	0.036	Protein synthesis	Nucleic acid-binding, OB-fold-like protein	1.00E-76
T2-L2	blue	Csa7G049260	2.259	0.022	Protein postranslational modification	Protein phosphatase 2C family protein	1.00E-109
T2-L2	blue	Csa7G230940	1.146	0.023	Redox	Glutaredoxin family protein	2.00E-30
T2-L2	blue	Csa7G253780	1.374	0.045	Transport	Polyol/monosaccharide transporter 1 (PMT1)	2.00E-190
T2-L2	blue	Csa7G318990	1.676	0.023	Biotic stress	Pathogenesis-related family protein	4.00E-62
T2-L2	blue	Csa7G352410	-1.471	0.026	Not assigned	unknown	6.00E-71
T2-L2	blue	Csa7G372360	-4.168	2.77E-06	Miscellaneous	Defective in induced resistance 1 (DIR1)	6.00E-21
T2-L2	blue	Csa7G429630	1.754	0.017	Not assigned	unknown	5.00E-35
T2-L2	blue	Csa7G432550	1.637	0.013	Not assigned	Tetratricopeptide repeat (TPR)-like superfamily protein	3.00E-50
T2-L2	brown	Csa4G297410	-4.596	1.61E-05	Abiotic stress	Pollen Ole e 1 allergen and extensin family protein	4.00E-62
T2-L2	brown	Csa3G179180	-4.461	1.86E-10	Not assigned	unknown	
T2-L2	brown	Csa7G390010	-3.971	1.12E-05	Not assigned	unknown	
T2-L2	brown	Csa1G062340	-2.872	2.35E-04	Miscellaneous	GDSL-like Lipase/Acylhydrolase superfamily protein	3.00E-171
T2-L2	brown	Csa6G139210	-2.397	2.49E-03	Miscellaneous	Indole-3-acetate beta-D-glucosyltransferase (IAGLU)	1.00E-27

Supplementary Table 1 (Continued)

T2-L2	brown	Csa5G642130	-1.918	1.35E-03	Miscellaneous	Peroxidase superfamily protein	1.00E-98
T2-L2	brown	Csa3G035890	-1.737	1.64E-04	Transcription factor	WRKY DNA-binding protein 40 (WRKY40)	2.00E-32
T2-L2	brown	Csa6G148170	-1.707	9.44E-13	Photosynthesis	Chlorophyll A/B binding protein 1 (CAB1)	6.00E-11
T2-L2	brown	Csa3G239280	-1.465	7.68E-03	Miscellaneous	Peroxidase superfamily protein	8.00E-14
T2-L2	brown	Csa4G126430	-1.413	1.80E-03	development	flowering-time gene CONSTANS (CO) encoding zinc-finger proteins	3.00E-86
T2-L2	brown	Csa1G066480	-1.393	7.30E-03	Not assigned	unknown	
T2-L2	brown	Csa2G369210	-1.274	3.12E-03	Not assigned	unknown	
T2-L2	brown	Csa7G451340	1	0	Miscellaneous	Nitrile lyases, berberine bridge enzymes, reticuline oxidases, troponine reductases	8.00E-21
T2-L2	brown	Csa6G109750	1.185	0.046	Miscellaneous	Bif-unctional inhibitor/lipid-transfer protein/seed storage 2S albumin superfamily protein	6.00E-41
T2-L2	brown	Csa6G318680	1.312	0.0007313	Protein degradation	Small ubiquitin-like modifier 1 (SUMO1)	1.00E-45
T2-L2	brown	Csa6G446450	1.369	4.52E-03	biotic stress	Late embryogenesis abundant (LEA) hydroxyproline-rich glycoprotein family	2.00E-65
T2-L2	brown	Csa3G150800	1.389	1.57E-03	not assigned	unknown	
T2-L2	brown	Csa6G150550	1.445	0.000008129	Redox	Copper/zinc superoxide dismutase 2 (CSD2)	1.00E-89
T2-L2	brown	Csa7G024130	1.47	2.38E-03	Not assigned	unknown	
T2-L2	brown	Csa2G336130	1.485	0.011	RNA binding	glycine-rich RNA binding protein (GLYCINE RICH PROTEIN 7)	1.00E-58
T2-L2	brown	Csa5G171740	1.562	3.769E-09	Miscellaneous	Copper amine oxidase family protein	4.00E-11
T2-L2	brown	Csa1G212840	1.58	0	Protein degradation	Cystatin/monellin superfamily protein	5.00E-07
T2-L2	brown	Csa7G452200	1.581	0.024	transport	Delta tonoplast intrinsic protein, functions as a water channel and ammonium (NH3) transporter	4.00E-11
T2-L2	brown	Csa6G521090	1.68	0.000001159	Miscellaneous	alpha/beta-hydrolases superfamily protein	1.00E-12
T2-L2	brown	Csa3G710200	1.705	3.66E-03	Not assigned	unknown	
T2-L2	brown	Csa7G025720	1.772	0.0001424	Protein postranslational modification	Protein kinase superfamily protein	4.00E-16
T2-L2	brown	Csa5G631510	1.776	2.25E-03	Not assigned	unknown	
T2-L2	brown	Csa2G009470	1.841	0.0003098	Lipid metabolism	Abnormal inflorescence meristem (AIM1)	3.00E-29
T2-L2	brown	Csa2G351840	2.309	0.00001605	Miscellaneous	Glutathione S-transferase zeta 1 (GSTZ1)	9.00E-78
T2-L2	brown	Csa4G001980	2.335	7.43E-04	not assigned	unknown	
T2-L2	brown	Csa3G736660	2.599	2.35E-04	Not assigned	unknown	
T2-L2	brown	Csa3G778440	2.637	0.001429	Miscellaneous	Glutathione S-transferase L3 (GSTL3)	1.00E-99

Supplementary Table 1 (Continued)

T2-L2	brown	Csa1G228960	3.136	0.00003412	Transport	Pyrophosphate-energized vacuolar membrane proton pump	0.00E+00
T2-L2	brown	Csa5G319910	3.216	0.003151	Transcription factor	WRKY DNA-binding protein 54 (WRKY54)	3.00E-96
T2-L2	brown	Csa6G148290	3.755	1.73E-05	Not assigned	unknown	
T2-L2	brown	Csa2G361590	5.96	1.72E-06	Not assigned	unknown	
T2-L2	brown	Csa7G447100	8		Transport	Tonoplast intrinsic protein 2;3 (TIP 2;3)	5.00E-107
T2-L2	brown	Csa6G356490	9.406	7.34E-04	Not assigned	unknown	
T2-L2	brown	Csa5G547610	9.656	0.0000344	Lipid metabolism	AMP-dependent synthetase and ligase family protein	6.00E-236
T2-L2	brown	Csa7G037660	14.168	7.21E-03	Not assigned	unknown	
T2-L2	brown	Csa1G109330	-3.47	0.013	Protein degradation	Serine carboxypeptidase-like 48 (SCPL48)	3.00E-109
T2-L2	brown	Csa1G123470	1.58	0	Protein degradation	Cystatin/monellin superfamily protein	
T2-L2	brown	Csa2G003610	1.841	3.10E-04	Lipid metabolism	Abnormal inflorescence meristem (AIM1)	3.00E-294
T2-L2	brown	Csa2G120410	1.347	3.65E-09	Protein synthesis	Zinc-binding ribosomal protein family protein	7.00E-47
T2-L2	brown	Csa2G382650	-1.737	1.64E-04	Transcription factor	WRKY DNA-binding protein 40 (WRKY40)	8.00E-147
T2-L2	brown	Csa3G119700	3.575	5.38E-05	Transcription factor	WRKY DNA-binding protein 53 (WRKY53)	1.00E-102
T2-L2	brown	Csa3G600020	-2.306	4.96E-04	Secondary metabolism	Chalcone synthase 2 (CHS2)	5.00E-212
T2-L2	brown	Csa3G734160	2.599	2.35E-04	Not assigned	Unknown	2.00E-17
T2-L2	brown	Csa3G814360	2.296	0.013	Not assigned	Unknown	8.00E-26
T2-L2	brown	Csa3G903520	-1.154	0.029	Photosynthesis	Glycine decarboxylase complex H (GDCH)	2.00E-78
T2-L2	brown	Csa3G911280	1.219	3.02E-03	Development	DUF581	2.00E-44
T2-L2	brown	Csa4G011770	3.01	0.015	Transcription factor	NAC domain containing protein 83 (NAC083)	3.00E-81
T2-L2	brown	Csa4G062920	1.404	6.20E-06	Miscellaneous	Polyamine oxisase 2 (PAO2)	3.00E-228
T2-L2	brown	Csa4G361820	3.071	0.03	Development	NAC domain containing protein 32 (NAC32)	5.00E-120
T2-L2	brown	Csa4G630010	-1.901	0.039	Hormone metabolism	Ethylene response factor 8 (ERF8)	2.00E-34
T2-L2	brown	Csa4G651750	-3.219	0.02	Transcription factor	Aspartyl protease family protein	2.00E-142
T2-L2	brown	Csa5G140450	7.962	0.02	DNA repair	Heavy metal transport/detoxification superfamily protein	1.00E-61
T2-L2	brown	Csa5G158570	1.562	3.77E-09	Miscellaneous	Copper amine oxidase family protein	4.00E-114
T2-L2	brown	Csa5G223070	3.216	3.15E-03	Transcription factor	WRKY DNA-binding protein 54 (WRKY54)	3.00E-96
T2-L2	brown	Csa5G605150	1.346	0.021	RNA processing	Ribonuclease T2	6.00E-105
T2-L2	brown	Csa5G608610	1.776	2.25E-03	Not assigned	Unknown	4.00E-31
T2-L2	brown	Csa5G650560	6.11	0.037	Miscellaneous	GDSL-like Lipase/Acylhydrolase superfamily protein	2.00E-153

Supplementary Table 1 (Continued)

T2-L2	brown	Csa6G014540	1.836	0.019	Cell wall	Expansin A8 (EXPA8)	6.00E-125
T2-L2	brown	Csa6G057170	-1.227	0.027	Photosynthesis	Light harvesting complex photosystem II subunit 6 (LHCB6)	3.00E-139
T2-L2	brown	Csa6G139750	-1.707	9.44E-13	Photosynthesis	Chlorophyll A/B binding protein 1 (CAB1)	1.00E-149
T2-L2	brown	Csa6G152330	1.508	2.61E-03	Protein synthesis	Ribosomal protein L1p/L10e family	1.00E-111
T2-L2	brown	Csa6G157680	1.312	7.31E-04	Protein degradation	Small ubiquitin-like modifier 2 (SUMO2)	1.00E-45
T2-L2	brown	Csa6G404210	10.38	0.029	Not assigned	SPFH/Band 7/ PHB domain-containing membrane-associated protein family	2.00E-155
T2-L2	brown	Csa6G404270	1.408	0.049	Abiotic stress	Germin-like protein 7 (GLP7)	7.00E-71
T2-L2	brown	Csa6G405340	-3.365	0.028	Cell wall	Glycosyl hydrolase superfamily protein	1.00E-205
T2-L2	brown	Csa6G517180	1.68	1.16E-06	Miscellaneous	Alpha/beta-hydrolases superfamily protein	1.00E-128
T2-L2	brown	Csa6G538750	1.47	2.38E-03	Not assigned	DUF23	2.00E-200
T2-L2	brown	Csa7G037610	14.168	7.21E-03	Not assigned	Unknown	2.00E-45
T2-L2	brown	Csa7G066240	3.449	0.036	Not assigned	Unknown	
T2-L2	brown	Csa7G071460	1.556	1.42E-04	Protein synthesis	Ribosomal protein S14p/S29e family protein	1.00E-28
T2-L2	brown	Csa7G073430	-3.971	1.12E-05	Not assigned	DUF579	3.00E-78
T2-L2	brown	Csa7G428940	1.343	0.033	Protein synthesis	Ribosomal protein L6 family protein	2.00E-115
T2-L2	brown	Csa7G430130	-1.498	0.047	Lipid metabolism	Bi-functional inhibitor/lipid-transfer protein/seed storage 2S albumin superfamily protein	8.00E-48
T2-L2	grey	Csa6G407650	1.837	0.0001765	Biodegradation of xenobiotic	UDP glycosyltransferase (UGT)	1.00E-132
T2-L2	grey	Csa1G050250	1.106	6.41E-03	Glycolysis	Glyceraldehyde-3-phosphate dehydrogenase	2.00E-188
T2-L2	grey	Csa3G680120	1.221	0.031	Minor CHO metabolism	Galactinol synthase 1 (GolS1)	1.00E-156
T2-L2	grey	Csa4G618520	1.837	1.77E-04	Hormone metabolism	UDP-Glycosyltransferase superfamily protein	1.00E-132
T2-L2	turquoise	Csa3G122470	-6.667	5.69E-04	Not assigned	unknown	
T2-L2	turquoise	Csa4G167980	-4.114	1.13E-06	Not assigned	unknown	
T2-L2	turquoise	Csa5G152260	-4.109	7.06E-04	Cell wall	Pectin lyase-like superfamily protein	1.00E-79
T2-L2	turquoise	Csa5G139600	-3.252	4.53E-05	Protein degradation	Prolyl oligopeptidase family protein	3.00E-69
T2-L2	turquoise	Csa4G000930	-3.167	4.50E-04	Not assigned	unknown	
T2-L2	turquoise	Csa7G387690	-3.105	5.63E-05	Not assigned	unknown	
T2-L2	turquoise	Csa3G849920	-2.921	6.11E-16	Transcription factor	MYB domain protein 30 (MYB30)	7.00E-209
T2-L2	turquoise	Csa6G190340	-2.765	8.71E-05	Miscellaneous	UDP-Glycosyltransferase superfamily protein	3.00E-84
T2-L2	turquoise	Csa6G518250	-2.436	7.19E-06	Development	Squamosa promoter binding protein-like 8 (SPL8)	4.00E-162
T2-L2	turquoise	Csa7G450510	-2.276	9.15E-03	Secondary metabolism	Cinnamoyl Coenzyme A reductase 1 (CCR1)	5.00E-152

Supplementary Table 1 (Continued)

T2-L2	turquoise	Csa5G505200	-2.248	1.07E-03	Protein degradation	Anaphase-promoting complex/cyclosome 2 (APC2)	8.00E-148
T2-L2	turquoise	Csa4G416430	-2.238	1.75E-03	Lipid metabolism	Nonspecific lipid-transfer protein precursor (LTP)	1.00E-109
T2-L2	turquoise	Csa7G049260	-1.969	6.68E-05	Hormone metabolism	Ethylene responsive element binding factor 4 (ERF4)	7.00E-113
T2-L2	turquoise	Csa3G202730	-1.907	1.38E-12	Secondary metabolism	Beta-hydroxylase 1 (BETA_OHASE1)	3.00E-89
T2-L2	turquoise	Csa1G420360	-1.884	5.86E-03	Not assigned	unknown	
T2-L2	turquoise	Csa7G043640	-1.87	9.28E-04	Protein degradation	Xylem cysteine peptidase 1 (XCP1)	7.00E-150
T2-L2	turquoise	Csa4G639870	-1.85	6.66E-03	Not assigned	unknown	
T2-L2	turquoise	Csa6G309950	-1.797	8.01E-05	Light signaling	Phototropin 1 (PHO1)	3.00E-45
T2-L2	turquoise	Csa1G050420	-1.733	4.47E-06	Not assigned	unknown	
T2-L2	turquoise	Csa3G252490	-1.725	6.57E-03	Not assigned	unknown	
T2-L2	turquoise	Csa6G109650	-1.652	7.76E-03	Secondary metabolism	Flavanone 3-hydroxylase (F3H)	7.00E-156
T2-L2	turquoise	Csa7G230940	-1.647	9.38E-06	Cell wall	Fasciclin-like arabinoglaaractan protein 1 (FLA1)	9.00E-70
T2-L2	turquoise	Csa2G000230	-1.618	1.21E-03	Cell wall	Fasciclin-like arabinogalaaractan protein 17 precursor (FLA17)	2.00E-119
T2-L2	turquoise	Csa1G462020	-1.607	6.30E-03	Biotic stress	Cystein-rich secretory proteins, Antigen 5, and Pathogenesis-related 1 protein	
T2-L2	turquoise	Csa6G517960	-1.596	8.96E-03	Development	Senescence 1 (SEN1)	5.00E-138
T2-L2	turquoise	Csa2G173070	-1.522	4.76E-03	Photosynthesis	Serine hydroxymethyltransferase 4 (SHM4)	3.00E-137
T2-L2	turquoise	Csa5G207940	-1.5	5.58E-08	Photosynthesis	RuBisCO activase (RA)	
T2-L2	turquoise	Csa3G182240	-1.494	5.11E-03	Secondary metabolism	2-oxoglutarate (2OG) and Fe(II)-dependent oxygenase superfamily protein	7.00E-93
T2-L2	turquoise	Csa5G162630	-1.494	0.021	miscellaneous	extensin-like protein (ELP) (Arabidopsis thaliana)	3.00E-52
T2-L2	turquoise	Csa3G563300	-1.485	7.90E-03	Lipid metabolism	Lysophospholipase 2 (LysoPL2)	1.00E-126
T2-L2	turquoise	Csa3G035840	-1.464	0.021	miscellaneous	Bi-functional inhibitor/lipid-transfer protein/seed storage 2S albumin superfamily protein (Arabidopsis thaliana)	5.00E-10
T2-L2	turquoise	Csa5G174640	-1.419	9.38E-06	Abiotic stress	Bcl2-associated anthanogene (ABG) protein	3.00E-77
T2-L2	turquoise	Csa7G336450	-1.375	6.08E-03	Photosynthesis	Non-photochemical quenching 4 (NPQ4)	4.00E-107
T2-L2	turquoise	Csa6G013960	-1.334	5.05E-03	Photosynthesis	RuBisCO activase (RA)	1.00E-69
T2-L2	turquoise	Csa3G539670	-1.282	7.34E-04	Photosynthesis	Photosystem I subunit H2 (PSAH2)	0
T2-L2	turquoise	Csa7G398090	-1.261	7.43E-04	development	Late embryogenesis abundant 3 (LEA3) family protein	2.00E-20
T2-L2	turquoise	Csa5G171730	-1.229	1.50E-04	not assigned	unknown	
T2-L2	turquoise	Csa3G826640	-1.228	1.14E-04	Lipid metabolism	Fatty acid desaturase 3	2.00E-108
T2-L2	turquoise	Csa3G874340	-1.221	3.64E-04	protein folding	DnaJ/Hsp40 cysteine-rich domain superfamily protein	6.00E-73

Supplementary Table 1 (Continued)

T2-L2	turquoise	Csa5G212590	-1.194	0.012	lipid metabolism	Omega-3 fatty acid desaturase, chloroplast precursor	1.00E-274
T2-L2	turquoise	Csa3G134920	1.136	6.18E-03	Not assigned	unknown	
T2-L2	turquoise	Csa6G381850	1.251	1.62E-03	Not assigned	unknown	
T2-L2	turquoise	Csa1G569380	1.278	2.06E-04	Abiotic stress	A member of heat shock protein 90 (HSP90) gene family (Arabidopsis thaliana)	3.00E-270
T2-L2	turquoise	Csa1G043180	1.285	0.00657	Hormone metabolism	Jasmonate-zim-domain protein 3 (JAZ3)	0.00E+00
T2-L2	turquoise	Csa6G289760	1.294	0.000364	Photosynthesis	ATP synthase protein 1-related	6.00E-131
T2-L2	turquoise	Csa5G179220	1.295	0.009199	RNA Processing	Fibrillarin 2 (FIB2)	1.00E-151
T2-L2	turquoise	Csa1G046840	1.3	6.55E-05	Not assigned	unknown	
T2-L2	turquoise	Csa6G151730	1.311	4.19E-04	Hormone metabolism	Multiprotein bridging factor 1B (MBF1B)	2.00E-42
T2-L2	turquoise	Csa3G483790	1.33	0.006473	RNA binding	RNA-binding (RRM/RBD/RNP motifs) family protein	7.00E-46
T2-L2	turquoise	Csa2G247040	1.332	1.62E-03	Not assigned	unknown	
T2-L2	turquoise	Csa1G039240	1.338	0.004886	RNA binding	Glycine-rich RNA-binding protein 3 (GR-RBP3)	3.00E-62
T2-L2	turquoise	Csa3G826690	1.409	6.998E-11	Transport	Silicon transpoter LSI1	3.00E-145
T2-L2	turquoise	Csa3G101810	1.426	0.003945	Protein synthesis	Translation initiation factor SUI family protein	9.00E-54
T2-L2	turquoise	Csa3G110620	1.426	0.002703	Transcription factor	NAC domain containing protein 3 (NAC3)	7.00E-102
T2-L2	turquoise	Csa4G000800	1.451	0.001625	RNA binding	Hyaluronan/mRNA binding family	2.00E-128
T2-L2	turquoise	Csa2G258680	1.466	0.01	Hormone metabolism	SAUR-like auxin-responsive protein family	0.00E+00
T2-L2	turquoise	Csa6G151110	1.503	9.23E-03	cell wall	cellulose synthase-like A02 (CSLA02) (Arabidopsis thaliana)	5.00E-251
T2-L2	turquoise	Csa3G153170	1.515	0.00005673	Redox	Thioredoxin superfamily protein	2.00E-39
T2-L2	turquoise	Csa6G421800	1.523	0.0007678	Protein degradation	Ubiquitin-like protein 5 (UBL5)	2.00E-35
T2-L2	turquoise	Csa4G651760	1.53	0.01	Transcription factor	MYB domain protein R1 (MYBR1)	
T2-L2	turquoise	Csa3G118080	1.532	9.679E-12	Development	Squamosa promoter binding protein-like 10 (SPL10)	1.00E-95
T2-L2	turquoise	Csa6G046270	1.562	0.0006147	Transcription factor	Constants-like zinc finger family	2.00E-186
T2-L2	turquoise	Csa1G041530	1.567	2.71E-03	Not assigned	unknown	
T2-L2	turquoise	Csa6G088070	1.582	1.49E-06	Not assigned	unknown	
T2-L2	turquoise	Csa3G808360	1.638	0.0002177	TCA	Putative alpha-carbonic anhydrase (CAH1)	4.00E-81
T2-L2	turquoise	Csa5G140480	1.639	0.00005501	Development	Nodulin MtN3 family	5.00E-85
T2-L2	turquoise	Csa3G127210	1.687	1.29E-03	Not assigned	unknown	
T2-L2	turquoise	Csa7G049230	1.702	0.036	not assigned	unknown	
T2-L2	turquoise	Csa2G368880	1.706	0.003583	Redox	Thioredoxin H-type 1 (TRX1)	1.00E-32
T2-L2	turquoise	Csa4G017110	1.754	4.35E-04	Not assigned	unknown	
T2-L2	turquoise	Csa1G569250	1.778	9.18E-03	Not assigned	unknown	

Supplementary Table 1 (Continued)

T2-L2	turquoise	Csa2G362490	1.788	0	Not assigned	unknown	
T2-L2	turquoise	Csa1G021950	1.803	0	RNA binding	Glycine-rich RNA-binding protein 3 (GR-RBP3)	6.00E-74
T2-L2	turquoise	Csa2G006870	1.822	0.000168	Cell organization	Profillin 3	3.00E-68
T2-L2	turquoise	Csa2G359940	1.869	0.027	RNA regulation of transcription	Member of the R2R3 factor MYB gene family; myb domain protein r1 (MYBR1)	4.00E-115
T2-L2	turquoise	Csa2G012120	1.884	0	Miscellaneous	Uclacyanin 1 (UCC1)	3.00E-38
T2-L2	turquoise	Csa2G028480	1.908	9.15E-03	hormone metabolism	Lipoxygenase 1 (EC 1.13.11.12) - (Solanum tuberosum)	2.00E-25
T2-L2	turquoise	Csa6G242200	1.979	2.91E-03	signaling	Rapid Alkalinization Factor (RALF) 23	4.00E-32
T2-L2	turquoise	Csa2G258610	1.987	0.0009968	RNA binding	Glycine rich protein 7 (GRP7)	1.00E-58
T2-L2	turquoise	Csa3G739040	2.068	0.00483	Biodegradation of xenobiotic	Gloxal oxidase-related protein	1.00E-258
T2-L2	turquoise	Csa2G431070	2.23	0.000006487	Abiotic stress	SHEPHERD (SHD)	0.00E+00
T2-L2	turquoise	Csa3G129540	2.231	0.021	protein synthesis	chloroplast ribosomal protein S7 (Arabidopsis thaliana)	3.00E-81
T2-L2	turquoise	Csa5G604980	2.243	0	Amino acid metabolism	O-acetylserine(thiol) lyase (OAS-TL)	4.00E-162
T2-L2	turquoise	Csa1G024920	2.253	0.007294	Protein postranslational modification	RACK1B	7.00E-174
T2-L2	turquoise	Csa1G386590	2.258	3.86E-04	not assigned	unknown	
T2-L2	turquoise	Csa1G587400	2.496	0.00002831	Protein degradation	Metallopeptidase M24 family protein	3.00E-172
T2-L2	turquoise	Csa7G395800	2.631	0.000001654	Transport	Sulfate transporter 3;1 (SULTR 3;1)	3.00E-307
T2-L2	turquoise	Csa1G600980	2.792	0.00737	Protein postranslational modification	AGC (cAMP-dependent, cGMP-dependent and protein kinase C) kinase family protein	2.00E-235
T2-L2	turquoise	Csa5G589260	2.953	0.000001814	Miscellaneous	Plant invertase/pectin methylesterase inhibitor superfamily protein	5.00E-62
T2-L2	turquoise	Csa5G139420	3.156	6.39E-05	Not assigned	unknown	
T2-L2	turquoise	Csa5G615230	3.177	2.18E-03	Not assigned	unknown	
T2-L2	turquoise	Csa4G620640	3.195	0	Protein targeting	Sigma factor binding protein 1 (SIB1)	2.00E-14
T2-L2	turquoise	Csa4G420110	3.334	0	Protein degradation	Metacaspase 9 (MC9)	1.00E-120
T2-L2	turquoise	Csa7G397040	3.415	0.001823	Miscellaneous	Glutathione S-transferase phi 8 (GSTF8)	3.00E-82
T2-L2	turquoise	Csa6G445040	3.81	6.53E-03	Not assigned	unknown	
T2-L2	turquoise	Csa6G382970	3.831	0.001156	Transport	Amino acid permease 3 (AAP3)	2.00E-234
T2-L2	turquoise	Csa1G039120	4.104	0.00004243	Development	Nascent polypeptide-associated complex NAC	4.00E-68
T2-L2	turquoise	Csa2G072490	4.767	5.874E-08	Protein amino acid activation	Glycyl-tRNA synthetase	0.00E+00

Supplementary Table 1 (Continued)

T2-L2	turquoise	Csa3G026690	4.954	0.006704	Lipid metabolism	Palmitoy (protein) hydrolase	5.00E-77
T2-L2	turquoise	Csa5G567800	5.13	0.00002257	Redox	Glutaredoxin	9.00E-41
T2-L2	turquoise	Csa2G070890	5.345	0.003945	Abiotic stress	DNAJ heat shock N-terminal domain-containing protein	2.00E-137
T2-L2	turquoise	Csa3G002850	5.719	0.00004243	Biotic stress	Thaumatin-like protein 1	2.00E-166
T2-L2	turquoise	Csa7G429630	9.428	8.95E-05	Not assigned	unknown	
T2-L2	turquoise	Csa6G008680	9.581	0.004781	Protein postranslational modification	CBL-interacting protein kinase 1 (CIPK1)	3.00E-169
T2-L2	turquoise	Csa5G470570	14.58	0.00536	Transcription factor	Response regulator 9 (ARR9)	7.00E-57
T2-L2	turquoise	Csa4G639890	15.851	0.00001712	Lipid metabolism	Triacylglycerol lipase like 1 (TLL1)	2.00E-131
T2-L2	turquoise	Csa7G009740	125.334	0.009597	Transcription factor	Cryptochrome-interacting basic-helix-loop-helix (CIB1)	1.00E-115
T2-L2	turquoise	Csa1G000010	1.357	0.029	Protein synthesis	60S acidic ribosomal protein family	5.00E-36
T2-L2	turquoise	Csa1G009690	1.458	3.16E-08	Protein synthesis	Ribosomal protein L34	1.00E-59
T2-L2	turquoise	Csa1G013160	1.322	0.01	Not assigned	Unknown	3.00E-42
T2-L2	turquoise	Csa1G013770	1.803	0	RNA binding	Glycine rich RNA-binding protein 5 (GR-RBP5)	6.00E-74
T2-L2	turquoise	Csa1G024170	1.522	3.55E-06	Protein synthesis	Ribosomal protein S7e family protein	2.00E-92
T2-L2	turquoise	Csa1G024830	2.253	7.29E-03	Protein postranslational modification	Receptor for activated C kinase 1B (RACK1B)	7.00E-174
T2-L2	turquoise	Csa1G031860	4.104	4.24E-05	Transcription factor	Basic transcription factor 3	4.00E-68
T2-L2	turquoise	Csa1G039190	1.338	4.89E-03	RNA binding	Glycine rich RNA-binding protein 5 (GR-RBP5)	3.00E-62
T2-L2	turquoise	Csa1G039250	1.567	2.71E-03	Not assigned	DUF1118	7.00E-70
T2-L2	turquoise	Csa1G042360	1.281	2.35E-03	Protein synthesis	Ribosomal protein S7e family protein	2.00E-91
T2-L2	turquoise	Csa1G042920	1.285	6.57E-03	Hormone metabolism	Jasmonate-zim-domain protein 3 (JAZ3)	2.00E-37
T2-L2	turquoise	Csa1G045540	1.3	6.55E-05	Not assigned	DUF1118	5.00E-67
T2-L2	turquoise	Csa1G050240	-1.256	0.029	Glycolysis	Glyceraldehyde-3-phosphate dehydrogenase (GAP-DH)	4.00E-189
T2-L2	turquoise	Csa1G050270	-1.733	4.47E-06	Not assigned	Unknown	1.00E-149
T2-L2	turquoise	Csa1G051810	1.38	0.012	Signaling	Calnexin 1 (CNX1)	4.00E-256
T2-L2	turquoise	Csa1G059750	2.634	0.024	Not assigned	Unknown	
T2-L2	turquoise	Csa1G063560	1.22	0.043	Abiotic stress	Heat shock protein 20 (HSP20)	1.00E-34
T2-L2	turquoise	Csa1G163140	-1.401	8.89E-03	Protein synthesis	Ribosomal protein L1p/L10e family	4.00E-111
T2-L2	turquoise	Csa1G168900	1.214	0.028	Protein synthesis	Ribosomal protein 5A	3.00E-100
T2-L2	turquoise	Csa1G479110	1.778	9.18E-03	Not assigned	Unknown	
T2-L2	turquoise	Csa1G570180	3.012	5.39E-05	Transport	Heavy metal transport/detoxification superfamily protein	1.00E-47
T2-L2	turquoise	Csa1G573590	1.361	3.54E-05	Protein synthesis	Ribosomal protein S25 family protein	7.00E-51

Supplementary Table 1 (Continued)

T2-L2	turquoise	Csa1G574950	1.58	0.014	Biotic stress	Receptor like protein 52	4.00E-80
T2-L2	turquoise	Csa1G575160	1.173	0.027	Transcription factor	SNF7 family protein	1.00E-105
T2-L2	turquoise	Csa1G586750	2.496	2.83E-05	Protein degradation	Metallopeptidase M24 family protein	3.00E-172
T2-L2	turquoise	Csa1G589720	2.792	7.37E-03	Protein postranslational modification	AGC (cAMP-dependent, cGMP-dependent and protein kinase C) kinase family protein	2.00E-235
T2-L2	turquoise	Csa1G604040	2.041	0.037	Redox	L-ascorbate oxidase precursor (ASO)	0
T2-L2	turquoise	Csa1G611290	1.417	0.014	Miscellaneous	Beta glucosidase 15 (BGLU15)	2.00E-187
T2-L2	turquoise	Csa1G629740	-1.618	1.21E-03	Cell wall	fasciclin-like arabinogalactan-protein precursor 17 (Fla17)	3.00E-199
T2-L2	turquoise	Csa2G000570	1.314	6.05E-03	Protein synthesis	Ribosomal L28e protein family	1.00E-65
T2-L2	turquoise	Csa2G000600	1.391	6.47E-04	Protein synthesis	Ribosomal protein L31e family protein	4.00E-59
T2-L2	turquoise	Csa2G000770	1.822	1.68E-04	Cell organization	Profilin 3 (PRF3)	3.00E-68
T2-L2	turquoise	Csa2G012710	1.521	3.62E-04	Transcription factor	NOP56-like pre RNA processing ribonucleoprotein	1.00E-290
T2-L2	turquoise	Csa2G013290	3.167	0.042	Lipid metabolism	Acyl carrier protein 4 (ACP4)	1.00E-37
T2-L2	turquoise	Csa2G033940	1.812	3.15E-05	Signaling	Calreticulin 1a (CRT1a)	4.00E-216
T2-L2	turquoise	Csa2G036680	-1.209	1.49E-03	Polyamine metabolism	S-adenosylmethionine decarboxylase proenzyme	7.00E-136
T2-L2	turquoise	Csa2G145880	-1.522	4.76E-03	Photosynthesis	Serine hydroxymethyltransferase 4 (SHM4)	2.00E-263
T2-L2	turquoise	Csa2G193310	1.498	6.85E-06	Protein synthesis	Ribosomal protein S15	1.00E-75
T2-L2	turquoise	Csa2G222050	1.332	1.62E-03	Not assigned	RmlC-like cupins superfamily protein	2.00E-88
T2-L2	turquoise	Csa2G285350	1.262	0.046	Protein synthesis	Translation protein SH3-like family protein	5.00E-71
T2-L2	turquoise	Csa2G302060	1.523	4.38E-07	Protein synthesis	Ribosomal protein L10 protein	9.00E-82
T2-L2	turquoise	Csa2G433350	-2.293	0.043	Secondary metabolism	4-coumarate-CoA ligase 2 (4CL2)	1.00E-187
T2-L2	turquoise	Csa3G002400	7.592	0.017	Lipid metabolism	AMP-dependent synthetase and ligase family protein	4.00E-137
T2-L2	turquoise	Csa3G002750	5.719	4.24E-05	Biotic stress	Thaumatin-like protein 1	2.00E-166
T2-L2	turquoise	Csa3G019340	4.954	6.70E-03	Lipid metabolism	Alpha/beta-hydrolases superfamily protein	5.00E-77
T2-L2	turquoise	Csa3G044520	-1.737	0.015	C1-metabolism	Ribonuclease E inhibitor RraA/Dimethylmenaquinone methyltransferase	2.00E-72
T2-L2	turquoise	Csa3G077610	1.426	3.95E-03	Protein synthesis	Translation initiation factor SUI family protein	9.00E-54
T2-L2	turquoise	Csa3G117960	1.532	9.68E-12	Development	Squamosa promoter binding protein-like 10 (SPL10)	1.00E-95
T2-L2	turquoise	Csa3G118140	1.413	9.80E-05	Protein synthesis	Ribosomal protein S6	7.00E-134
T2-L2	turquoise	Csa3G122460	-6.667	5.69E-04	Not assigned	Unknown	5.00E-18
T2-L2	turquoise	Csa3G134910	1.136	6.18E-03	Not assigned	Unknown	3.00E-46
T2-L2	turquoise	Csa3G134930	1.756	1.64E-04	Protein synthesis	Ribosomal protein L14	4.00E-63
T2-L2	turquoise	Csa3G135010	1.319	0.028	Protein synthesis	Ribosomal protein L30/L7 family protein	6.00E-125

Supplementary Table 1 (Continued)

T2-L2	turquoise	Csa3G149910	-2.523	0.033	Not assigned	DUF679	6.00E-20
T2-L2	turquoise	Csa3G152090	1.515	5.67E-05	Redox	Thioredoxin superfamily protein	2.00E-39
T2-L2	turquoise	Csa3G183920	-1.907	1.38E-12	Secondary metabolism	Beta-hydroxylase 1 (BETA-OHASE1)	4.00E-161
T2-L2	turquoise	Csa3G214020	-1.725	6.57E-03	Not assigned	Maternal effect embryo arrest 9 (MEE9)	1.00E-56
T2-L2	turquoise	Csa3G403980	1.33	6.47E-03	RNA binding	RNA-binding (RRM/RBD/RNP motifs) family protein	7.00E-46
T2-L2	turquoise	Csa3G483830	-1.282	7.34E-04	Photosynthesis	Photosystem I subunit H-1	6.00E-67
T2-L2	turquoise	Csa3G639040	1.71	0.016	Protein synthesis	Stress response suppressor 1 (STRS1)	1.00E-213
T2-L2	turquoise	Csa3G653380	1.422	5.67E-08	Protein synthesis	Zinc-binding ribosomal protein family protein	2.00E-57
T2-L2	turquoise	Csa3G653460	1.642	1.62E-03	Protein synthesis	Nucleolin-like 2 (NUC-L2)	1.00E-162
T2-L2	turquoise	Csa3G698560	1.52	5.30E-04	Protein synthesis	Ribosomal protein L6 family protein	3.00E-111
T2-L2	turquoise	Csa3G710180	2.47	0.02	Not assigned	Unknown	1.00E-37
T2-L2	turquoise	Csa3G733320	2.068	4.83E-03	Biodegradation of xenobiotic	Glyoxal oxidase-related protein	1.00E-258
T2-L2	turquoise	Csa3G748210	1.46	9.83E-09	Protein synthesis	Ribosomal protein L24e family protein	3.00E-82
T2-L2	turquoise	Csa3G776970	1.638	2.18E-04	TCA	Alpha carbonic anhydrase 1 (CAH1)	4.00E-81
T2-L2	turquoise	Csa3G855430	1.847	0.019	Miscellaneous	O-Glycosyl hydrolases family 17 protein	1.00E-162
T2-L2	turquoise	Csa3G895680	3.397	0.026	Transcription factor	Apetala2/Ethylene-responsive element binding protein family	2.00E-56
T2-L2	turquoise	Csa3G914560	1.451	1.63E-03	RNA binding	Hyaluronan/mRNA binding family	2.00E-128
T2-L2	turquoise	Csa4G004870	1.754	4.35E-04	Not assigned	Unknown	8.00E-34
T2-L2	turquoise	Csa4G025120	-1.865	0.027	Not assigned	Unknown	3.00E-50
T2-L2	turquoise	Csa4G052080	3.146	0.035	Not assigned	Unknown	1.00E-08
T2-L2	turquoise	Csa4G091870	3.269	1.43E-04	Secondary metabolism	2-oxoglutarase (2OG) and Fe(II)-dependent oxygenase superfamily protein	1.00E-144
T2-L2	turquoise	Csa4G152270	-4.114	1.13E-06	Not assigned	Unknown	
T2-L2	turquoise	Csa4G269770	2.17	0.047	Protein postranslational modification	Protein kinase 2B (APK2B)	1.00E-153
T2-L2	turquoise	Csa4G290160	1.607	0.011	Protein postranslational modification	Receptor-like protein kinase 1 (RLK1)	4.00E-27
T2-L2	turquoise	Csa4G297420	2.307	0.037	Not assigned	Cysteine/Histidine-rich C1 domain family protein	4.00E-47
T2-L2	turquoise	Csa4G307390	-2.045	0.022	Cell organization	Tubulin beta chain 3	5.00E-256
T2-L2	turquoise	Csa4G333620	-2.238	1.75E-03	Lipid metabolism	Lipid-transfer protein 6 (LTP6)	2.00E-29
T2-L2	turquoise	Csa4G431960	3.195	0	Protein targeting	Sigma factor binding protein 1 (SIB1)	2.00E-14

Supplementary Table 1 (Continued)

T2-L2	turquoise	Csa4G641690	1.538	0.01	Transcription factor	MYB domain protein 77 (MYB77)	1.00E-72
T2-L2	turquoise	Csa4G651970	1.399	3.03E-08	Protein synthesis	Eukaryotic translation initiation factor 5A-2 (elF-5A-2)	2.00E-85
T2-L2	turquoise	Csa5G097970	3.156	6.39E-05	Not assigned	Unknown	5.00E-12
T2-L2	turquoise	Csa5G153020	1.177	0.016	Transport	Plasma membrane intrinsic protein 1;4 (PIP1;4)	2.00E-147
T2-L2	turquoise	Csa5G160150	-2.457	1.80E-06	Miscellaneous	Plant invertase/pectin methylesterase inhibitor superfamily protein	1.00E-102
T2-L2	turquoise	Csa5G168920	2.675	0.026	Signaling	Leucine-rich repeat receptor -like protein kinase family protein	3.00E-93
T2-L2	turquoise	Csa5G171160	1.599	1.44E-04	Protein synthesis	Ribosomal protein S10p/S20e family protein	2.00E-59
T2-L2	turquoise	Csa5G174560	-1.419	9.38E-06	Abiotic stress	BCL-2-associated athaogene 3 (BAG3)	3.00E-93
T2-L2	turquoise	Csa5G180850	1.312	0.05	Protein synthesis	Ribosomal protein L23AA	6.00E-73
T2-L2	turquoise	Csa5G182730	-1.5	5.58E-08	Photosynthesis	RuBisCO activase (RA)	1.00E-204
T2-L2	turquoise	Csa5G320430	-2.045	3.44E-07	Secondary metabolism	Phytoene synthase (PSY)	5.00E-232
T2-L2	turquoise	Csa5G409620	1.474	2.76E-05	Protein synthesis	Ribosomal protein L7Ae/L30e/S12e/Gadd45 family protein	9.00E-54
T2-L2	turquoise	Csa5G434550	14.58	5.36E-03	Transcription factor	Response regulator 3	7.00E-57
T2-L2	turquoise	Csa5G523190	5.13	2.26E-05	Redox	Glutaredoxin family protein	9.00E-41
T2-L2	turquoise	Csa5G606660	1.869	0.037	Miscellaneous	Carbohydrate-binding X8 domain superfamily protein	9.00E-53
T2-L2	turquoise	Csa5G606810	2.66	0.024	Not assigned	Unknown	3.00E-06
T2-L2	turquoise	Csa5G610410	3.177	2.18E-03	Not assigned	Unknown	1.00E-08
T2-L2	turquoise	Csa5G638420	1.444	6.70E-03	Protein synthesis	Ribosomal protein S19e family protein	3.00E-73
T2-L2	turquoise	Csa5G645120	1.554	2.58E-04	Protein synthesis	Ribosomal protein L16p/L10e family protein	2.00E-74
T2-L2	turquoise	Csa5G649850	1.378	6.08E-03	Protein synthesis	Ribosomal protein S4	4.00E-106
T2-L2	turquoise	Csa5G652280	1.316	0.029	Transcription factor	MYB domain protein 6 (MYB6)	4.00E-84
T2-L2	turquoise	Csa6G001220	9.581	4.78E-03	Protein postranslational modification	CBL-interacting protein kinase 1 (CIPK1)	3.00E-169
T2-L2	turquoise	Csa6G014790	1.439	2.35E-04	Protein synthesis	R-protein L3B (RPL3B)	5.00E-214
T2-L2	turquoise	Csa6G039540	1.562	6.15E-04	Transcription factor	Constant-like zinc finger family	2.00E-186
T2-L2	turquoise	Csa6G055370	1.611	1.54E-06	Protein synthesis	Ribosomal protein S18C	2.00E-82
T2-L2	turquoise	Csa6G077430	1.669	2.33E-03	Protein synthesis	Ribosomal protein S13	1.00E-78
T2-L2	turquoise	Csa6G087710	1.582	1.49E-06	Not assigned	Ribosome associated membrane RAMP4	1.00E-26
T2-L2	turquoise	Csa6G091290	1.404	7.56E-03	Protein synthesis	Ribosomal protein S27	7.00E-45
T2-L2	turquoise	Csa6G108510	-1.652	7.76E-03	Secondary metabolism	Flavanone 3-hydroxylase (F3H)	4.00E-118
T2-L2	turquoise	Csa6G133770	2.243	1.52E-07	Hormone metabolism	Cytokinin response factor 4 (CRF4)	9.00E-38
T2-L2	turquoise	Csa6G151720	1.43	2.74E-09	Protein synthesis	60S acidic ribosomal protein family	

Supplementary Table 1 (Continued)

T2-L2	turquoise	Csa6G181560	-2.765	8.71E-05	Miscellaneous	UDP-Glycosyltransferase superfamily protein	6.00E-145
T2-L2	turquoise	Csa6G301020	-1.797	8.01E-05	Signaling	Phototropin 2 (PHOT2)	0
T2-L2	turquoise	Csa6G365120	1.251	1.62E-03	Not assigned	Unknown	5.00E-45
T2-L2	turquoise	Csa6G405880	1.523	7.68E-04	Protein degradation	Ubiquitin-like protein 5 (UBL5)	2.00E-35
T2-L2	turquoise	Csa6G507370	-1.596	8.96E-03	Development	Senescence 1 (SEN1)	2.00E-80
T2-L2	turquoise	Csa6G519680	1.421	2.95E-06	Protein synthesis	Ribosomal protein L16B	8.00E-96
T2-L2	turquoise	Csa6G538630	1.859	0.015	Co-factor and vitamin metabolism	Molybdenum cofactor sulfurase family protein	2.00E-128
T2-L2	turquoise	Csa7G000520	125.334	9.60E-03	Transcription factor	Cryptochrome-interacting basic-helix-loop-helix (CIB1)	1.00E-115
T2-L2	turquoise	Csa7G027790	-1.87	9.28E-04	Protein degradation	Xylem cysteine peptidase 1 (XCP1)	1.00E-162
T2-L2	turquoise	Csa7G108300	-1.647	9.38E-06	Cell wall	Fasciclin-like arabinogalactan-protein 1 (FLA1)	2.00E-153
T2-L2	turquoise	Csa7G267900	-1.375	6.08E-03	Photosynthesis	Photosystem II 22kDa protein chloroplast precursor (CP22)	3.00E-110
T2-L2	turquoise	Csa7G397560	1.605	7.19E-06	Protein synthesis	Ribosomal L22e protein family	2.00E-58
T2-L2	turquoise	Csa7G414420	9.428	8.95E-05	Not assigned	Unknown	
T2-L2	turquoise	Csa7G432010	-2.276	9.15E-03	Secondary metabolism	Cinnamoyl CoA reductase	4.00E-168
T2-L2	turquoise	Csa7G451340	2.619	1.72E-06	Miscellaneous	Nitrile lyases, berberine bridge enzymes, reticuline oxidases, troponine reductases	0
T2-L2	yellow	Csa5G011650	-71.384	1.63E-03	Biotic stress	ADR1-like 1 (ADR1-L1)	8.00E-257
T2-L2	yellow	Csa5G603370	-4.312	2.12E-03	Cell wall	RXF12	1.00E-144
T2-L2	yellow	Csa3G859730	-3.412	9.15E-03	Miscellaneous	Cytochrome P450, family 82, subfamily C, polypeptide 4 (CYP82C4)	7.00E-83
T2-L2	yellow	Csa1G033120	-3.352	4.23E-03	Transcription factor	Aspartyl protease family protein	6.00E-130
T2-L2	yellow	Csa6G312550	-2.678	1.24E-03	Secondary metabolism	Phenylalanine ammonia-lyase 1 (PAL2)	9.00E-30
T2-L2	yellow	Csa3G300600	-1.888	2.47E-04	Miscellaneous	Plant invertase/pectin methylesterase inhibitor superfamily protein	4.00E-52
T2-L2	yellow	Csa5G623670	-1.447	0.018	not assigned	unknown	
T2-L2	yellow	Csa2G028500	1.232	7.93E-03	Hormone metabolism	Lipoxygenase 1 (LOX1)	0.00E+00
T2-L2	yellow	Csa6G324880	1.335	0.0001866	Protein targeting	Importing alpha isoform 2	2.00E-281
T2-L2	yellow	Csa7G398150	1.351	0.003881	Cell wall	Expansin B3 (EXPB3)	2.00E-117
T2-L2	yellow	Csa4G292970	1.368	4.84E-03	Not assigned	unknown	
T2-L2	yellow	Csa6G445150	1.382	7.92E-04	Not assigned	unknown	
T2-L2	yellow	Csa7G047420	1.409	0.031	cell wall	fasciclin-like arabinogalactan-protein 6 (Fla6)	7.00E-49
T2-L2	yellow	Csa6G007450	1.436	0.003383	Cell wall	MYB domain protein 6 (MYB6)	4.00E-80
T2-L2	yellow	Csa3G643770	1.534	0.001616	Transport	Lysine histidine transporter 2 (LHT2)	5.00E-213

Supplementary Table 1 (Continued)

T2-L2	yellow	Csa5G609110	1.732	0.004961	Hormone metabolism	IAA-Alanine resistant 3 (IAR3)	2.00E-173
T2-L2	yellow	Csa1G590300	1.827	1.18E-05	Major CHO metabolism	Hexokinase 2 (HXK2)	7.00E-51
T2-L2	yellow	Csa6G121970	1.841	0.02	hormone metabolism	EXORDIUM like 5 (EXL5)	4.00E-159
T2-L2	yellow	Csa6G495000	1.86	1.50E-05	Not assigned	unknown	
T2-L2	yellow	Csa3G002330	2.238	0.007581	Transcription factor	MYB domain protein 73 (MYB73)	1.00E-88
T2-L2	yellow	Csa3G481240	2.483	0.000002215	Cell wall	Maternal effect embryo arrest 31 (MEE31)	4.00E-159
T2-L2	yellow	Csa1G003490	2.619	0.000001719	Miscellaneous	nitrile lyases, berberine bridge enzymes, reticuline oxidases, troponine reductases	0.00E+00
T2-L2	yellow	Csa4G000030	2.908	0.00547	Hormone metabolism	S-adenosyl-L-methionine: jasmonic acid carboxyl methyltransferase	0
T2-L2	yellow	Csa3G850600	2.992	2.07E-03	Not assigned	unknown	
T2-L2	yellow	Csa1G066560	3.395	0.0001601	Signaling	Cystine-rich RLK 29 (CRK29)	8.00E-79
T2-L2	yellow	Csa2G028490	3.4	0.0002165	Protein degradation	Cystein proteinases superfamily protein	2.00E-168
T2-L2	yellow	Csa3G665100	6.455	2.944E-09	Secondary metabolism	Transparent testa 4 (TT7)	9.00E-146
T2-L2	yellow	Csa1G073810	6.575	0.002475	Secondary metabolism	Terpene synthase 14 (TPS14)	2.00E-144
T2-L2	yellow	Csa1G596520	6.671	1.497E-07	Glycolysis	Phenylalanine ammonia-lyase 2 (PAL2)	0.00E+00
T2-L2	yellow	Csa3G852600	8.469	0	Miscellaneous	Cytochrome P450 family 82 subfamily C polypeptide 4 (CYP82C4)	8.00E-145
T2-L2	yellow	Csa2G033340	9.201	6.89E-03	Not assigned	unknown	
T2-L2	yellow	Csa6G127320	9.393	0.000009377	Secondary metabolism	MYB domain protein 10 (MYB10)	7.00E-51
T2-L2	yellow	Csa1G066570	11.211	4.726E-08	Secondary metabolism	Terpene synthase 14 (TPS14)	2.00E-133
T2-L2	yellow	Csa3G589590	12.087	1.93E-05	Not assigned	unknown	
T2-L2	yellow	Csa6G057160	15.701	0.00008718	Hormone metabolism	UDP-glucosyltransferase 75B1 (UGT75B1)	3.00E-128
T2-L2	yellow	Csa1G068570	26.653	0.001096	Secondary metabolism	Terpene synthase 14 (TPS14)	8.00E-12
T2-L2	yellow	Csa1G006320	2.533	2.29E-05	Biotic stress	Enhanced disease susceptibility 1 (EDS1)	2.00E-159
T2-L2	yellow	Csa1G022490	-3.352	4.23E-03	Transcription factor	Aspartyl protease family protein	3.00E-135

Supplementary Table 1 (Continued)

T2-L2	yellow	Csa1G064830	3.424	0.029	Signaling	Cystein-rich receptor like kinase 10 (CRK10)	
T2-L2	yellow	Csa1G065930	3.395	1.60E-04	Signaling	Cystein-rich receptor like kinase 29 (CRK29)	
T2-L2	yellow	Csa1G085390	1.609	5.43E-03	Transcription factor	C2H2-like zinc finger protein	
T2-L2	yellow	Csa1G574970	1.827	1.18E-05	Major CHO metabolism	Hexokinase 2 (HXK2)	
T2-L2	yellow	Csa2G351740	1.399	0.013	Transcription factor	MYB-like transcription factor family protein	8.00E-51
T2-L2	yellow	Csa2G369060	1.582	6.50E-03	Protein synthesis	Ribosomal protein S6	5.00E-133
T2-L2	yellow	Csa2G369220	3.723	0.04	Not assigned	ENTH/ANTH/VHS superfamily protein	1.00E-72
T2-L2	yellow	Csa2G427310	2.238	7.58E-03	Transcription factor	MYB domain protein 73 (MYB73)	1.00E-88
T2-L2	yellow	Csa3G016990	1.432	6.40E-03	Development	DUF581	7.00E-26
T2-L2	yellow	Csa3G166350	-3.315	0.017	Not assigned	Unknown	2.00E-116
T2-L2	yellow	Csa3G176260	-1.888	2.47E-04	Miscellaneous	Plant invertase/pectin methylesterase inhibitor superfamily protein	5.00E-63
T2-L2	yellow	Csa3G852590	8.469	0	Miscellaneous	Cytochrome P450 family 82 subfamily C polypeptide 4 (CYP82C4)	8.00E-145
T2-L2	yellow	Csa4G056640	1.53	0.011	Hormone metabolism	Nine-cis-epoxycarotenoid dioxygenase 4 (NCED4)	1.00E-249
T2-L2	yellow	Csa4G280660	1.368	4.84E-03	Not assigned	Unknown	3.00E-31
T2-L2	yellow	Csa4G638480	-71.384	1.63E-03	Biotic stress	Apoptosis defense response1-like 1 (ADR1-L1)	3.00E-292
T2-L2	yellow	Csa5G097460	-1.366	1.15E-06	Biotic stress	P-loop containing nucleotide triphosphate hydrolases superfamily protein	6.00E-82
T2-L2	yellow	Csa5G146260	3.213	0.02	Protein degradation	RING/U-box superfamily protein	8.00E-40
T2-L2	yellow	Csa5G240140	-4.312	2.12E-03	Cell wall	Glycosyl hydrolase superfamily protein	0
T2-L2	yellow	Csa5G651640	1.436	3.38E-03	Transcription factor	MYB domain protein 6 (MYB6)	4.00E-80
T2-L2	yellow	Csa6G147460	-2.678	1.24E-03	Secondary metabolism	Phenylalanine lyase 4 (PAL4)	0
T2-L2	yellow	Csa6G365170	1.397	2.67E-03	Protein synthesis	Ribosomal protein L22p/L17e family protein	2.00E-93
T2-L2	yellow	Csa6G398200	1.382	7.92E-04	Not assigned	Unknown	9.00E-102
T2-L2	yellow	Csa6G445170	1.436	0.012	Protein synthesis	Ribosomal protein L11 family protein	5.00E-85
T2-L2	yellow	Csa6G493850	1.86	1.50E-05	Not assigned	Unknown	9.00E-136

Supplementary Table 1 (Continued)
